# Supplementary material for: Do digital innovations for HIV and sexually transmitted infections work? Results from a systematic review (1996-2017)
Source: BMJ Open. 2017 Nov 3;7(11):e017604. doi: 10.1136/bmjopen-2017-017604 (PMC5695353; doi:10.1136/bmjopen-2017-017604)
Supplement: Supplementary file 1 [file bmjopen-2017-017604supp001.pdf]

Appendix 1: Search Strategy.

Search #1	"HIV Infections"[Mesh] OR "HIV" [MeSH] OR “human immunodeficiency virus”[tiab] OR “human immuno deficiency virus”[tiab] OR “human immune deficiency virus”[tiab] OR “human immunedeficiency virus”[tiab] OR “aids”[tiab] OR “acquired immunodeficiency syndrome”[tiab] OR “acquired immunodeficiency syndromes”[tiab] OR “acquired immuno deficiency syndrome”[tiab] OR “acquired immuno deficiency syndromes”[tiab] OR “acquired immune deficiency syndrome”[tiab] OR “acquired immune deficiency syndromes”[tiab] OR “acquired immunedeficiency syndrome”[tiab] OR “acquired immunedeficiency syndromes”[tiab]
Search #2	"mHealth" [tiab] OR "telemedicine"[MeSH] OR telemedicine[tiab] OR eHealth[MeSH] OR ehealth[tiab] OR "mobile health" [tiab] OR “mobile technology”[tiab] OR “app”[tiab] OR “apps”[tiab] OR “mobile applications” OR social medi*[tiab] OR cell phone* [tiab] OR cellphone*[tiab] OR “cellular phone”[mesh] OR cellular phone*[tiab] OR smartphone*[tiab] OR smart phone*[tiab] OR mobile phone[tiab] OR mobile device*[tiab] OR cellular telephone*[tiab] OR mobile telephone*[tiab] OR text messag*[tiab] OR texting[tiab] OR texted[tiab] OR SMS[tiab] OR MMS[tiab] OR multimedia messag*[tiab] OR short messag*[tiab] OR “computers, handheld”[mesh] OR personal digital assistant*[tiab]
Search #3 [1,2]<br><br>References<br><br>1.Ferreira A, Young T, Mathews C, Zunza M, Low N. Strategies for partner notification for sexually transmitted infections, including HIV. Cochrane Database of Systematic Reviews 2013, Issue 10. Art. No.: CD002843. DOI: 10.1002/14651858.CD002843.pub2<br><br>2.Obiero J, Mwethera PG, Wiysonge CS. Topical microbicides for prevention of sexually transmitted infections. Cochrane Database of Systematic Reviews 2012, Issue 6. Art. No.: CD007961. DOI: 10.1002/14651858.CD007961.pub2	sexually transmitted infections[mh] OR sexually transmitted disease*[tiab] OR sexually transmissible disease*[tiab] OR sexually transmitted infection*[tiab] OR sexually transmissible infection*[tiab] OR sexually transmitted infectious disease*[tiab] OR sexually transmissible infectious disease*[tiab] OR sexually transmitted disorder*[tiab] OR sexually transmissible disorder*[tiab] OR STI[tiab] OR STIs[tiab] OR STD[tiab] OR STIs[tiab] OR venereal disease*[tiab] OR venereal infection*[tiab] OR venereal disorder*[tiab] OR genital herpes[tiab] OR herpes genitalis[mh] OR herpes genitalis[tiab] OR genital infection*[tiab] OR genital disorder*[tiab] OR herpes simplex[tiab] OR herpes virus[tiab] OR HSV-1[tiab] OR HSV-2[tiab] OR chancroid[mh] OR chancroid* [tiab] OR haemophilus ducreyi[tiab] OR chlamydia infection*[tiab] OR chlamydia trachomatis[mh] OR chlamydia trachomatis[tiab] OR gonorrhea[mh] OR gonorrhoea*[tiab] OR gonorrhea*[tiab] OR syphilis[mh] OR syphilis[tiab] OR cuminat[tiab] OR condylomata lata[tiab] OR chancre*[tiab] OR lymphogranuloma venereum[mh] OR lymphogranuloma venereum[tiab] OR granuloma Inguinale[mh] OR granuloma inguinale[tiab] OR donovania[tiab] OR donovanosis[tiab] OR calymmatobacterium[mh] OR calymmatobacterium granulomatis[tiab] OR klebsiella granulomatis[tiab] OR klebsiella granulomatis[tiab] OR treponema pallidum[mh] OR treponema pallidum[tiab] OR genital wart*[tiab] OR venereal wart*[tiab] OR condylomata cuminate[mh] OR human papillomavirus 6[mh] OR hpv-6[tiab] OR hpv-11[tiab] OR hpv6[tiab] OR human papillomavirus[tiab] OR hepatitis b[mh] OR hepatitis b[tiab] OR trichomonas vaginitis[mh] OR trichomonas vaginitis[tiab] OR genital ulcer*[tiab] OR anogenital ulcer*[tiab] OR anorectal ulcer*[tiab] OR anorectal ulcer*[tiab] OR penile ulcer*[tiab] OR blood-born pathogen*[tiab] OR blood-borne infection*[tiab] OR blood-borne virus*[tiab]
Search #4	#1 OR #3
Search #5	#2 AND #4
