# Supplementary material for: Do digital innovations for HIV and sexually transmitted infections work? Results from a systematic review (1996-2017)
Source: BMJ Open. 2017 Nov 3;7(11):e017604. doi: 10.1136/bmjopen-2017-017604 (PMC5695353; doi:10.1136/bmjopen-2017-017604)
Supplement: Supplementary file 2 [file bmjopen-2017-017604supp002.pdf]

Appendix 2: **Abstraction table.**

Combined Innovations	Author	Study Design	Participants/ Country	STBBI	Intervention	Measure/Metric	Results (MD=Mean difference, RR=risk ratio, IRR=incidence rate ratio, HR=hazard ratio, SD= standard deviation, 95% CI when presented. M=months, W=weeks)
**Online campaign + SMS + TV.**	Friedman 2014	Quasi-experimental: HxCtrl w/ population data.	≤25 yrs, USA. n=N/A	HIV, CT, GC	GetYourselfTested: TV campaign w/ website & SMS service for STI info & clinic locator.	ATT testing: Attendance rate.	41.2% more CT tests in 2010 vs. 2008, 90.5% more GC tests, and 190.3% more HIV tests.
						Acceptability: Number of followers.	4477 FB followers and 1994 Twitter followers at yr 2.
						Feasibility: Referral rate.	83,404 referrals using clinic locator in yr1. 61,119 in yr2.
**Interactive website + SMS + cash incentives.**	Horvath 2013	RCT	HIV+ Gay/Bi-sexual men 18+ yrs, USA. n=67(Int) n=57(Ctrl)	HIV	Online self-monitoring system w/ interactive interface + optional SMS reminders +$25 gift card draw.	ART in PVLA: Self-report. [Difference scores: DS = FU-baseline]	No difference. (DS=0.54, SD=25.2 vs. DS=-3.2, SD=24.5; t(107)=1.79, p=0.43) / No impact.
						ART in PVLA: Self-report.	Increased adherence in drug users (DS= 7.1, SD= 22.1 vs. DS= -24, SD= 30.5; t(17)=2.52, p=0.02) / Effective.
						ART in PVLA: Self-report.	Trend to taking meds within 2hrs of scheduled dose. DS=6.6, SD=29.3 vs. DS=-3, SD=29.6; t(105)=1.68, p=0.1 / No impact.
						Acceptability: Self-report.	Mean score = 5.7 on 7-point Likert Scale for satisfaction / Highly acceptable.
						Feasibility: Completion rate.	Completion rate 88% vs. 93% in Ctrl / Highly feasible.
**Website + SMS**	Gotz 2014	Cross-sectional study.	STI index patients at clinic, NLD. n=988	HIV, CT, GC, syph	Suggestatest.nl: online partner notification via SMS/email.	PN: % partners notified.	14% notifications via SAT. 505 notifications sent (84% by SMS, 15% by email). 56% read notification. 20% visited one of 2 STI clinics.
**Social media + SMS.**	Hightow-Weidman 2014	Quasi-experimental: HxCtrl.	HIV+ or syphilis+ patients, USA. n=362(Int) n=133(HxCtrl)	HIV, syphilis	Notification on social networking sites + SMS	PN: % partners notified.	63.5% of contacts notified via internet in 2011 vs. 26% in 2010.
**PC/SMS/MMS + WhatsApp messages**	John 2016	UnCtrlled trial.	HIV+ non-disclosed, 15-29 yrs, NGA. n=19	HIV	Weekly counselling, educational & motivational calls, SMS/MMS and WhatsApp messages over 3M.	Self-care: Self-report.	Significant increase in self-care performance at 6MI (p=0.002)/ Effective.
**Interactive website + SMS**	Hightow-Weidman 2015	Feasibility study.	Black MSM & transwamen 18-30 yrs, USA. n=15	HIV	HealthMpowerment.org: online community networking Int to reduce STI risk + health promotion messages.	Acceptability: Self-report.	86.7%-100% strongly agreed w/ acceptability questions / Highly acceptable.
						Feasibility: Retention rate.	100% retention rate. 7/15 participants used the site 1W after study ended / Highly feasible.
**Mobile app + SMS**	Hirsch-Moverman 2017	Feasibility study.	≥18yrs, HIV+/TB, LSO. n=171	HIV/TB	CommCare application used to automatically send SMS medication reminders over 29M	Acceptability: Self-report.	41.9% think SMS facilitated adherence to TB /ART medication / Less acceptable.

**Mobile app + SMS**	Aronson 2016	Feasibility study	18-24 yrs, USA.<br>n=100	HIV	App assessing risk and sending SMS to encourage re-testing of HIV negatives.	Feasibility: Completion rate	98/100 completed the app process/ Highly feasible<br>30/100 accepted to receive HIV test<br>21/30 accepted to receive SMS<br>1/21 re-tested after 90 days window period.
**Website + SMS**	Dokkum 2012	UnCtrlled trial.	16-29 yrs, NLD.<br>n=52600(Rd 1)<br>n=41700(Rd 2)	CT	At-home CT test + SMS/email to return test for analysis.	Feasibility: Completion rate.	Higher retesting rates (From 10% w/o reminders to 14% in round 1; from 7% to 10% in round 2) / Less feasible.

Note: Int= intervention; Ctrl= control; HxCtrl= historical control; PB= preventative behaviors (i.e. risk reduction); PN= partner notification; TAT= turnaround time; ATT= attendance rate; ART= ART adherence; NAPs= non-adherent patients; AP= adherent patients; PVLA= Patients with various levels of adherence; TNPs= Treatment naive patients; VL= viral load; CD4= CD4 cell count; PC= phone call; FB= Facebook.

Internet-based eHealth Innovation	Author	Study Design	Participants/ Country	STBBI	Intervention	Measure/Metric	Results (MD=Mean difference, RR=risk ratio, IRR=incidence rate ratio, HR=hazard ratio, SD= standard deviation, 95% CI when presented. M=months, W=weeks)
**Online campaign**	Downshen 2015	Quasi-experimental: HxCtrl w/ population data.	13-17 yrs, USA. n=1500	HIV, CT, GC, syphilis	IknowUshould2: social-media campaign w/ website for STI info & clinic locator.	ATT testing: Attendance rate.	More syphilis tests (18.8% vs. 5.4%; p<0.01) and HIV tests (19.0% vs. 5.4%; p<0.01). No change for CT & GC / Effective.
						Acceptability: Number of followers.	1500+ unique website interactions. 128 FB likes; 46 Twitter followers; 390 Youtube views; 42 Instagram followers.
**Social media campaign**	Elliot 2016	Cross-sectional study.	MSM, GBR. n=17361	HIV	Promotion through Gaydar, Grindr, Recon and FB pages to order free postal HIV home sampling kits	ATT testing: Participation rate.	10 323/11 127 (93%) ordered HIV sample kit. 5696/10 323 (55%) returned sample kit within 24M. 82/5696 (1.4%) confirmed new diagnosis and in care.
						Acceptability: Self-report.	59.7% would recommend to someone expected to test positive (93.8% if expected to negative). 64% clicked for more info on test. / Moderately acceptable.
**Social media campaign**	Huang 2016	Cross-sectional	≥18yrs, Black/African American or Hispanic/Latino MSM, USA. n=122	HIV	Promoting of HIV self-testing for 6W on GrindR + study website to order self-test kit	ATT testing: Participation rate.	122 requested tests; 55/57 HIV-, 2/57 HIV+.
						Acceptability: Number of followers	11 939 unique website visitors; 2.8% click-through rate 334 tests requested.
						Feasibility: Completion rate.	122/334 visitors were eligible and completed baseline survey, 81/122 confirmed receiving self test kit, 57/122 completed follow-up survey / Less feasible.
**Social media campaign**	Jones 2015	Cross-sectional study.	MSM, GBR. n=305	HIV	Health promotion and offer of rapid at-home testing via FB, Grindr, and Squirt.	ATT testing: Participation rate.	5/5 high risk sexual behavior but tested HIV negative; 1/5 never tested before; 3/5 not tested in many yrs.
						Acceptability: Number of followers.	103 clicked FB survey; 152 approached on Grindr; 50 Squirt contacts.
						Feasibility: Completion rate.	FB: 6/103 completed survey; 3/6 requested HIV test; 2/3 made appointment. Grindr: 20/152 engaged; 6/20 requests for at home test; 3/6 made appointment. Squirt: 3/50 engaged and 0/3 test requests / Less feasible.
**Social media campaign**	Rhodes 2016	Quasi-experimental.	MSM & transgender, USA n=339 (Int) n=286 (Ctrl)	HIV	Posting info and answering questions on HIV testing on social media sites (Adam4Adam, BlackGayChat, Craigslist, and Gay.com).	ATT testing: Self-report.	63.7% of intervention participants reported past 12M HIV testing compared with 42.0% of control. Adjusted OR= 2.9 (1.8-4.7)/ Effective.
**Social media campaign + website**	Rosengren 2016	Cross-sectional	Black or Hispanic MSM 18+ yrs, USA n=56	HIV	Promotion of free rapid HIV self-testing kits on Grindr and offer of delivery via study website (kit, voucher or pin for smart vending machine)	ATT testing: Self-report.	All 56 reported testing completion (100%); 2/56 reported positive result and linkage to care (confirmatory testing and ART initiation)
						Feasibility: Completion rate.	4389 visited the website; 333 requested test (i.e. 1 in 13 visitors); 56 completed survey 2W after request/ Less feasible.
						ART in TNPs: Self-report.	Higher adherence at 3M & 6M (71.2% vs. 63.9%, d=0.17; 70.3% vs. 66.6%, d=0.09)
**Mobile phone application**	Himelhoch 2016	RCT	18-64yrs, history of drug/alcohol use, HIV+, USA. n=19(Int) n=9(Ctrl)	HIV	Heart2HAART mobile application for ART adherence	ART in NAPs: Pill count	No significant difference in adherence between intervention and control group (p=0.29), but adherence was 100% in both at 3M / No impact
						Acceptability: Self-report.	94.3% strongly agreed/agreed Heart2HAART helped them take their medication / Highly acceptable.
						Feasibility: Response rate.	App was used on avg 21.4, 19.1 and 16.4 times in months 1, 2 and 3. Participants responded to medication prompts on avg 18, 16 and 14 times during months 1,2 and 3 respectively.

**Avatar-guided computer software**	Kurth 2014	RCT	HIV+ 18+ yrs, USA.<br>n=120(Int)<br>n=120(Ctrl)	HIV	Audio narrated risk assessment, skill building videos, tailored feedback and printouts vs. computer risk assessment only.	ART in PVLA: VL.	Non-significant change. (log10VL= -0.06(-0.4 to -0.3), p=0.74). Significant in subgroup w/ detectable VL at baseline (-0.73(-1.42 to -0.03), p=0.041) / No impact.
						ART in PVLA: Self-report.	Increased adherence. (4.71(0.95- 8.48) increase vs. 1.39(6.03 to 3.24) decrease; p=0.046) / Effective.
						PB: Self-report.	Lower odds of HIV transmission (OR=0.46 (0.25-0.84), p=0.012) / Effective.
						Acceptability: Self-report.	97% reported ease of use and high privacy; 99% satisfied w/ session length; 75% preferred it over human counsellor / Highly acceptable.
						Feasibility: Retention rate.	87.1% retention / Highly feasible.
**Avatar-guided computer program**	Naar-King 2012	RCT	HIV+ 16-24 yrs, USA.<br>n=36(Int) n=40(Ctrl)	HIV	2-D animated character delivering personalized health feedback vs. character giving nutrition info.	ART in TNPs: VL.	Larger suppression rate. (Cohen's d=0.09 at 3M; d= 0.28 at 6M). Larger drop in VL from baseline (d=0.39 at 3M & d=0.19 at 6M).
						ART in TNPs: Self-report.	Higher adherence at 3M & 6M<br>(71.2% vs. 63.9%, d=0.17; 70.3% vs. 66.6%, d=0.09)
						Acceptability: Self-report.	Mean satisfaction ratings 3.7 out of 4 / Highly acceptable.
**Mobile phone application**	Perera 2014	RCT	HIV+, NZ. n=17(Int)<br>n=11(Ctrl)	HIV	ART adherence app w/ medication clock & graphs on disease-state vs. standard app (medication clock only)	ART in PVLA: Self-report.	Increased adherence (F(1,23)=5.37, p=0.03) / Effective.
						ART in PVLA: Pharmacy refills.	No difference. (F(1,25)=1.88, p=0.18) / No impact.
						ART in PVLA: VL.	Lower VL at 3M (F(1,23)=5.62, p=0.023) / Effective.
						ART in PVLA: Composite score (refills, VL, & self-report).	Increased adherence (53% to 13%, X2(1,15)=6, p=0.03). No change in Ctrl (27% to 27%, X2(1,11)=0.00, p>0.99) / Effective.
						Acceptability: Self-report.	More satisfying (on 11 point-scale: 5.88 vs. 3.27, p=0.017) and informative (6 vs. 3, p=0.034) at 3M than standard app / Highly acceptable.
**Mobile app + cash incentive**	Brayboy 2017	UnCtrlled trial.	12-17yrs, USA.<br>n=17	STI	GirlTalk mobile phone app to assess knowledge increase	PB: Self-report.	75.6% to 79% increase in knowledge pre and post app use at 2W. / No impact.
						Acceptability: Self-report.	94.1% would use the app again/recommend it / Highly acceptable
**Social media**	Jones 2012	Quasi-experimental: HxCtrl.	15–24 yrs, USA.<br>n=70/896 FB friends	CT	Educational FB site addressing safe sexual health.	PB: Self-report.	Condom from 57% to 80%. 54% reduction in CT in ages 15-17 from previous yrs (but 42% less tests done).
**Videos vs. SMS**	Jones 2013	RCT	High-risk urban African-American women 18-29 yrs, USA.<br>n=117(Soap opera)<br>n=121(SMS)	HIV	Weekly soap opera episodes (Love, Sex & Choices) vs. HIV prevention SMS.	PB: Self-report.	18% greater reduction in Int. group, p=0.23 / No impact.<br>78% reduction in risky acts from baseline in Int. group (p<0.001); 72% reduction from baseline in Ctrl (p<0.001)/ Effective
						Acceptability: Self-report.	97.4% liked the videos / Highly acceptable.
**Social media + video chat**	Lelutiu-Weinberger 2014	UnCtrlled trial.	MSM 18-29 yrs, high risk for STI, USA.<br>n=31	HIV	miCHAT: FB chat Int. 8 motivational interviews to reduce HIV risk + CBT training.	PB: Self-report.	Decrease in unprotected anal sex acts (3.11 vs. 8.96; p=0.042). Increased knowledge of sexual risk (p=0.01) / Effective.
						Acceptability: Self-report.	All felt privacy was ensured / Highly acceptable.
						Feasibility: Completion rate.	46% completed baseline assessment + minimum 5 sessions / Less feasible.

**Social media campaign + website + cash incentive**	Solorio 2016	Feasibility study.	Hispanic MSM, 18-30 yrs, USA<br>n=50	HIV	Radio & social media-based campaign for 16W to encourage testing & condome use + website w/clinic locator to provide free HIV home testing kits and linkage to care	PB: self-report.	No significant change in condom use at 16W (26.1% vs. 15.65, OR=1.9 (0.6-5.9))/ No impact.
						Feasibility: Self-report.	32/50 (64%) requested HIV home testing kit, 28/32 (88%) completed the test/ Moderately feasible.
**Mobile app**	Jeon 2016	RCT.	Chronic HBV+, 19-60 yrs, KOR<br>n=26 (Int)<br>n=27 (Ctrl)	HBV	App to increase disease knowledge, set alarm medication reminders, record lab nutrition & physical activity data, and chat with other users.	Self-care: Self-report.	Significantly higher self-care performance in intervention vs. control (t=3.597, p=0.001)/ Effective.
						Feasibility: Utilisation rate.	Average monthly utilisation rate was 75.1%/ Highly feasible.
**Social media**	Henwood 2016	Feasibility study.	12-25 yrs, HIV+, ZAF<br>n=90	HIV	Use of MXit as support group for HIV+ youth	Acceptability: Self-report.	84% would like chat-room to continue / Highly acceptable.
						Feasibility: Participation rate	33% ever visited MXit chat-room / Less feasible.
**Mobile app + cash incentive**	Przybyla 2016	Feasibility study.	HIV + on ART, 18+ yrs, USA<br>n=27	HIV	DRUM app to report daily on ART adherence and substance abuse.	Acceptability: Self-report.	84% reported the app was easy to use; 96% were satisfied; 92% would use it in the future/ Highly acceptable.
						Feasibility: Completion rate.	Overall completion rate of daily reports after 2W= 95.3%/ Highly feasible.
**Telemedicine**	Talal 2016	Feasibility study.	Individuals on opioid agonist tx, USA<br>n=54	HCV	Telemedicine-based medical tx with hepatologist	Acceptability: Self-report.	88.9% prefer medical tx using telemedicine vs. clinic visit; 100% would recommend it to a friend/ Highly acceptable.
						Feasibility: Completion rate.	54 tested HCV+ over 14M; 81.5% started evaluation/tx; 75% of those given tx have completed it/ Highly feasible.
**Social media**	Garett 2016	Feasibility study.	18+yrs, MSM, PER<br>n=102(Int)<br>n=109(Ctrl)	HIV	12W FB based peer-led intervention to increase HIV testing and prevention behaviour.	Acceptability: Self-report.	Intervention group felt they learned more about; where to receive sexual health services (p-value=0.0061), more likely to have safe sex (p-value=0.034) and more likely to get tested for HIV regularly (p-value=0.021) compared to control group / Highly acceptable.
**Website**	Polilli 2016	Feasibility study.	Residents of Abruzzo Region, ITA<br>n=3500	HIV, syphilis, HBV, HCV	Website with STI info, risk calculator, and appointments booking at testing sites.	Feasibility: Completion rate.	3500 booked an appointment; 3046 (87%) presented for testing within 15M study period/ Highly feasible.

Note: Int= intervention; Ctrl= control; HxCtrl= historical control; PB= preventative behaviors (i.e. risk reduction); PN= partner notification; TAT= turnaround time; ATT= attendance rate; ART= ART adherence; NAPs= non-adherent patients; AP= adherent patients; PVLA= Patients with various levels of adherence; TNPs= Treatment naive patients; VL= viral load; CD4= CD4 cell count; PC= phone call; FB= Facebook.

Basic mHealth Innovation	Author	Study Design	Participants/ Country	STBBI	Intervention	Measure/Metric	Results (MD=Mean difference, RR=risk ratio, IRR=incidence rate ratio, HR=hazard ratio, SD= standard deviation, 95% CI when presented. M=months, W=weeks)
**SMS**	Bailey 2014	UnCtrlled trial.	CT+ at clinic, AUS. n=64	CT	SMS reminders to recall for treatment.	ATT treatment: Attendance rate.	100% treated for CT infection. 72% treated within 1 day of SMS.
						Feasibility: Response rate.	94% replied to SMS, 84% the same day / Highly feasible.
**SMS + PC**	Bassett 2016	RCT	≥18yrs, ZAF. n=543(Int) n=471(Ctrl)	HIV/TB	5 scheduled PC) and 4 SMS, reminders to retrieve test results and attend appointments, over 4M.	ATT treatment: Attendance rate.	No significant difference in reaching outcome at 9M (3M ART treatment or 3+6M of TB treatment) between intervention and control (39% vs 42%, RR=0.93, 95%CI 0.80-1.08) / No Impact
**SMS + PC**	Bigna 2014	RCT	Caregivers of HIV + children 18+ yrs, CMR. n=61(SMS+PC) n=60(PC) n=60(SMS) n=61(Ctrl)	HIV	SMS+PC, SMS, or PC appointment reminders.	ATT FU appointment: Attendance rate.	Improved attendance. (OR=2.9 (1.3-6.3), p=0.012) / Effective.
**SMS**	Brook 2013	Quasi-experimental: HxCtrl.	Sexual health clinic, GBR. n=207(Int) n=169(HxCtrl)	HIV, syphilis, HBV	SMS reminders.	ATT testing: Attendance rate.	Higher retesting rate (41% vs. 28%; p<0.001) / Effective.
**SMS**	Brook 2013	Quasi-experimental: HxCtrl.	Sexual health clinic, GBR.n=699(Int) n=768(HxCtrl)	HIV	SMS reminders 2 days before appointment.	ATT FU appointment: LTFU rate.	35% improvement in overall LTFU rate (26% to 17%; p<0.0001) / Effective.
**SMS**	Burton 2013	Quasi-experimental: HxCtrl.	High risk for STI at clinic, GBR. n=273(Int) n=266(Ctrl)	CT, GC	SMS STI testing reminders.	ATT: testing: Attendance rate.	No change in retesting rates for those w/ recent CT or GC. (CT: 36% vs.33%; p=0.79) (GC: 19% vs. 33%; p=0.48) / No impact.
**SMS**	Coleman 2017	Retrospective Quasi-experimental	>=18 yrs, HIV+ pregnant women, ZAF. n=192(Int) n=447(Ctrl)	HIV	Bi-weekly maternal health info sent throughout pregnancy and for one year after birth to increase HIV PCR testing postpartum and increase ANC visits	ATT testing: Attendance rate.	81.3% vs 75.4% in intervention vs control group likely to attend first PCR 6W postpartum. 40% increase in the likelihood of attending the recommended four ANC visits among individuals within the intervention group (RR: 1.41, CI: 1.15–1.72) / Effective.
						PB: Infection rate	3 infants born with HIV in control group
**SMS**	Desai 2014	Quasi-experimental: Conc. + HxCtrl.	High risk MSM at clinic, GBR. n=31(Int) n=656(Conc. Ctrl) n=745(HxCtrl)	HIV	SMS HIV/STI testing reminders.	ATT testing: Attendance rate.	No significant change in re-testing odds. (32% in SMS vs.30% in Conc. Ctrl; OR=1.1(0.5-2.4) and 17% in HxCtrl; OR=2.3(1.0-4.9) / No impact.
**SMS + cash incentive**	Downing 2013	RCT	CT + or suspected at clinic 16+ yrs, AUS. n=30(Int) n=32(Ctrl)	CT	SMS appointment reminders + $10 if attended.	ATT testing: Attendance rate.	Increased re-testing rate at 10-12W post CT treatment (without cash 26.7% vs. 6.3% in Ctrl; p=0.04); (with cash 28.1% vs. 6.3% in Ctrl; p=0.043) / Effective.
**SMS**	Evans 2015	UnCtrlled trial.	African community, GBR. n=172	HIV	2 weekly Health Belief Model SMS to reduce risky sexual behaviours.	ATT testing: Self-report.	10.5% reported being tested for HIV during/after the 12W Int.
						PB: Self-report.	Non-significant increase in HIV knowledge & attitudes / No impact.
						Acceptability: Self-report.	Acceptable & useful. Majority shared w/ others and want to get tested in future.

**SMS**	Farmer 2014	Quasi-experimental: HxCtrl.	HIV clinic attendees, GBR. n=951(Int) n=822(HxCtrl)	HIV	SMS reminder 2 days before appointment.	ATT FU appointment: LTFU & cancellation rate.	No difference in LTFU (25% vs.28%) or cancellation (62% vs.64%) / No impact.
**SMS**	Finocchario-Kessler 2014	Quasi-experimental: HxCtrl.	HIV+ mother-infant pairs, KEN. n=523(Int) n=320(HxCtrl)	HIV	SMS notification of available test results and appointment reminder.	ATT treatment: Attendance rate.	More infants initiated on ART (Urban: 11/11 vs. 1/7, p<0.001; Peri-urban: 14/14 vs. 9/14, p<0.05) / Effective.
						TAT: Time from test to diagnosis & test to treat.	Shorter median time to diagnosis (5 vs. 6.3W (urban) & 3.4 vs. 8.1W (peri-urban); both p<0.001). Shorter median time to treat (13 vs. 40 days (urban) & 1 vs. 36 days (peri-urban); p<0.001) / Effective.
						Feasibility: Retention rate.	Retention rate double at 9M post-natal (45.1% vs. 93% (urban) and 43.2% vs. 94.1% (peri-urban); p<0.001) / Highly feasible.
**SMS**	Guy 2012	Quasi-experimental: HxCtrl.	STI clinic, AUS. n=141(Int) n=338(HxCtrl)	CT	SMS re-testing reminder 3M after initial infection.	ATT testing: Attendance rate.	Higher retesting rate (30% 1-4M post-infection vs. 21%; p=0.04); AOR= 1.57(1.01-2.46) / Effective.
**SMS**	Joseph Davey 2016	RCT.	HIV+ adults on ART, MOZ n=416 (Int) n=414 (Ctrl)	HIV	SMS reminders 2 and 7 days of appointment and ART drug-pick up + educational SMS every 2M.	ATT treatment: Attendance rate.	Nonsignificant difference in overall retention in care at 12 M (93.8% vs 91%, p=0.139)/ No impact.
**SMS**	Kapman 2016	Quasi-experimental: HxCtrl.	Heterosexual clinic attendees dx & tx for CT, 16-23 yrs, NLD n=828 (Int) n=1530 (Ctrl)	CT	2 SMS reminders at 5.5M & 6M after initial dx with CT for retesting appointment scheduling & attendance.	ATT testing: Attendance rate.	Higher attendance rate between 5-8M after initial dx (30.6% vs. 9.2%).
**SMS**	Kharbanda 2011	Quasi-experimental: Conc. + HxCtrl.	Parents of girls 9-20 yrs at clinics, USA. n=124(Int) n=308(Conc. Ctrl) n=1080(HxCtrl)	HPV	Up to 3 weekly SMS vaccination reminders.	ATT vaccination: Attendance rate.	More likely to get vaccine on time after controlling for insurance and site of care (AOR=1.83(1.23-2.71)) / Effective.
**SMS**	Kliner 2013	Quasi-experimental: HxCtrl.	HIV+ at hospital, SWZ. n=162(Int) n=297(HxCtrl)	HIV	SMS reminders one day before appointment.	ATT FU appointment: Attendance rate.	No difference. SMS 83.3% vs. Ctrl 80.1%; p=0.401. AOR=1.13, p=0.662 / No impact.
**SMS**	Matheson 2014	Quasi-experimental.	11-22 yrs at clinic, USA. n=37(Int) n=232(Ctrl)	HPV	SMS vaccination reminders (3 SMS per dose).	ATT vaccination: Attendance rate.	Higher attendance rate. HPV2 vaccine complete: 73% vs.34%, (p=0.000); on-time HPV2 38% vs. 25%, (p=0.035). HPV3 complete 16% vs.6%, (p=0.018); on-time HPV3 14% vs.3%, (p=0.007) / Effective.
**SMS**	McIver 2016	Quasi-experimental: HxCtrl.	Clinic attendees susceptible to HBV (HIV+, bisexual, CSW, IDUs, Aboriginals), AUS n=241 (Int) n=463 (Ctrl)	HBV	SMS reminders 1 day before appointment for HBV vaccine doses 2&3 reattendance.	ATT vaccination: Attendance rate.	Nonsignificant decrease in attendance rate within 12 M (54% vs 56% for 2 doses, p=0.65/ 24% vs 30% for 3 doses, p=0.07)/ No impact Nonsignificant difference in completion of 3 doses in 12M. aOR= 0.7 (0.48-1.01)/No impact.
**SMS**	Njuguna 2016	RCT.	Rural women, 18-24 yrs, KEN n=300 (Int) n=300 (Ctrl)	HIV	Weekly SMS on HIV and reproductive health.	ATT testing: Self-report.	Significant increase in reported testing at 6M (67% vs 51%, aHR=1.54(1.25-1.90)/ Effective.
**SMS vs. PC**	Norton 2014	RCT	HIV+, 17+ yrs, USA. n=25(Int) n=27(Ctrl)	HIV	SMS appointment reminder vs. message to home phone.	ATT FU appointment: Attendance rate.	No difference (72% vs. 81%, p=0.42) but patients already had high attendance rate / No impact.

**SMS**	Nyatsanza 2016	Quasi-experimental: HxCtrl.	MSM & CSW at high-risk of STI, GBR n=266 (Int) n=273 (Ctrl)	HIV/STI	Personalised SMS reminders for reattendance.	ATT testing: Attendance rate.	Significantly higher reattendance rate at 6M (56% vs. 33%, p<0.001)/ Effective.
**SMS**	Odeny 2012	RCT	Males circumcised at clinic 18+ yrs, KEN. n=600(Int) n=600(Ctrl)	HIV	Daily SMS for 1W.	ATT FU appointment: Attendance rate.	Improved attendance within 3 days of post-operative clinic appointment: 65.4% vs.59.7% (RR=1.09(1.00–1.20); p=0.04) / Effective.
						PB: Self-report.	Abstention of sexual activity before FU: 28.3% vs. 25.2% (RR=1.13(0.91-1.38), p=0.3) / No impact.
**SMS**	Rand 2015	RCT	11-16 yrs at clinic, USA. n=1893(Int) n=1919(Ctrl)	HPV	SMS appointment reminders.	ATT vaccination: Attendance rate.	Higher HPV1 vaccination rate (16% vs. 13%; HR= 1.3(1.0-1.6); p=0.04) / Effective.
**SMS/PC**	Rand 2016	RCT.	Clinic attendees Parents of youth 11-17 yrs who received 1st HPV vaccine, USA. n=191 (SMS) n=200 (Ctrl); n=178 (PC) n=180 (Ctrl)	HPV	SMS appointment reminders to receive 3 doses of HPV vaccine over 2 yrs.	ATT vaccination: Attendance rate.	SMS: Significant difference in vaccination rates compared to control (49% vs 30%, p=0.001)/ Effective.<br>PC: No difference in vaccination rates compared to control (48% vs 40%, p=0.34)/ No impact.
						TAT: Time from enroll to completion of 3 vaccines.	SMS: Significant difference in time taken to complete 3 HPV doses (71 days earlier than control, p<0.001)/ Effective.<br>PC: No difference in time taken to complete 3 HPV doses compared to control (p=0.08)/ No impact.
**SMS + PC**	Schwartz 2015	Quasi-experimental: HxCtrl.	HIV+ pregnant women on ART, ZAF. n=50	HIV	SMS messages and PCs from a case manager (CM) through 6W postpartum.	ATT testing: Attendance rate.	More infant testing (90.0% vs. 63.3% at 10W; p<0.01) / Effective.
						Acceptability: Self-report.	Helpful to have CM support during pregnancy and postpartum (98%) / Highly acceptable.
						Feasibility: Completion rate.	96% completed postpartum questionnaire / Highly feasible.
**SMS + PC**	Segaren 2012	UnCtrlled trial.	Mothers of HIV+ infants, HTI. n=108	HIV	Cell phones + regular PC for monitoring of mother & child.	ATT treatment: Attendance rate.	All 76 w/ active phones were adherent to treatment (attended 6/6 monthly hospital appointments).
						Acceptability: Self-report.	70% phones active after Int.; good for med reminders (63%) / Moderately acceptable.
**SMS + PC**	Smillie 2014	UnCtrlled trial.	HIV+ in clinic 14+ yrs, CAN. n=20	HIV	Weekly PC or SMS for 6M.	ATT FU appointment: Self-report.	65% said SMS had no effect on attendance.
						Acceptability: Self-report.	Beneficial for appointment scheduling (80%) & reminder (75%). All would recommend to a friend / Highly acceptable.
						Feasibility: Self-report.	75% had no difficulty in receiving and responding to SMS / Highly feasible.
**SMS**	Tolly 2012	RCT	Randomly sampled adults (existing database), ZAF. n=438(in each of 4 Int.) n=801(Ctrl)	HIV	3 or 10 motivational or informational SMS.	ATT testing: Self-report.	Improved attendance in group receiving 10 motivational SMS at 3W: (69% vs. 57%; OR=1.7(1.10–2.390), p=0.0036) / Effective.
						Feasibility: Self-report.	SMS motivated HIV counseling and testing uptake in 89% / Highly feasible.
**SMS**	Vilella 2004	Quasi-experimental: Conc. + HxCtrl.	18+ yrs at travel clinic, ESP. n=738(Int) n=1610(Conc. Ctrl) n=2247(HxCtrl)	HAV/ HBV	SMS reminders for vaccination appointments.	ATT vaccination: Attendance rate.	Improved adherence for 3rd HepA+B dose. (47.1% vs. 26.9%, RR=1.75(1.41–2.17) in Conc. Ctrl and 23.6%(20.1–27.4), RR=2.00(1.63–2.45) in HxCtrl) / Effective.

**SMS**	Ammassari 2010	UnCtrlled trial.	HIV+, ITA. n=71	HIV	SMS reminders.	ART in NAPs: Self-report.	Increased adherence over 9M. (93.2% vs.79.6%, p=0.003) / Effective.
**SMS**	Ammassari 2011	UnCtrlled trial.	HIV+, 18+ yrs, ITA. n=145	HIV	SMS reminders.	ART in NAPs: Self-report.	Increased adherence at 9M (94.9% vs.78.8%, p<0.001) / Effective.
						ART in NAPs: VL.	More w/ undetectable VL at 9M (76.2% vs. 42.3%, p<0.001) / Effective.
						Acceptability: Self-report.	>90% reporting SMS helpful / Highly acceptable.
**PC + cash incentives**	Belzer 2014	RCT	HIV+ 12-29 yrs, USA. n=19(Int) n=18(Ctrl)	HIV	Daily PC reminders and referrals if necessary+ free phone & plan.	ART in NAPs: Self-report.	Increased adherence for 1M &3 M (OR=3.09(1.20-7.98); OR=2.85(1.02-7.97)) / Effective.
						ART in NAPs: VL.	Lower VL at wk 24 and 48 (2.82 vs. 4.52, p=0.002; 3.23 vs. 4.23, p=0.043) / Effective.
**SMS**	Cantudo-Cuenca 2016	Retrospective quasi-exprimental.	HIV + on ART, ESP n=120 (Int&Ctrl)	HIV	SMS on ART adherence.	ART in PVLA: Pharmacy refills.	Statistically sign relationship bt no SMS and ART adherence(OR= 0.35 (0.14-0.8), p=0.025) [multivariate analysis]/ Effective.
**SMS**	da Costa 2012	RCT	HIV+ women, BRA. n=8(Int) n=13(Ctrl)	HIV	Daily SMS reminders.	ART in APs: Pill count.	Increased adherence over 4M (50% vs. 38.5%; p=0.604) / No impact.
						ART in APs: MEM.	Increased adherence over 4M (75% vs. 46%; p=0.195) / No impact.
						ART in APs: Self-report.	Increased adherence (100% vs. 84.6% in Ctrls; p=0.244) / No impact.
						Acceptability: Self-report.	82% believed SMS were helpful, 77% wanted to keep receiving SMS / Highly acceptable.
**SMS**	Downshen 2011	UnCtrlled trial.	HIV+ 14-29 yrs, AUS. n=25	HIV	Daily SMS ART reminder + FU SMS 1hr later.	ART in NAPs: Self-report.	Increased adherence (Baseline Mean=74.7; 12W Mean=93.3; 24WMean=93.1; p<0.001) / Effective.
						ART in NAPs: VL + CD4 count.	Insignificant change in CD4 cell count & VL (mean VL= 2750, CD4= 502 to VL= 29, CD4= 545 at 24W, p=0.12) / No impact.
						Acceptability: Self-report.	81% want SMS after study end. Helped decrease missed doses in 95% / Highly acceptable.
**SMS**	Downshen 2011	UnCtrlled trial.	HIV+ 14-29 yrs, AUS. n=25	HIV	Daily SMS ART reminder + FU SMS 1hr later.	ART in NAPs: Self-report.	Decreased adherence (58.3% for 0-12W vs. 55.2% for 13-24W, p=0.53) / No impact.
						Feasibility: Completion & response rate.	84% completed all study visits. 61.4% response rate / Highly feasible.
**SMS + cash incentive**	Garofalo 2016	RCT	16-29yrs, HIV+ on ART for ≥1M , USA. n=51(Int) n=54(Ctrl)	HIV	Daily personlised SMS over 6M to remind participants take medications	ART in NAPs: Self-report.	Significant difference in adherence compared to control at 3M OR=2.57 (1.01-6.54). Not significant at 6M OR=1.68 (0.69-4.09). Significant difference from baseline to 6M OR=2.12 (95% CI 1.01-4.45). / Effective.
						ART in NAPs: VL.	No difference in log viral load or viral suppression compared to control at 3 and 6M / No impact.
						Acceptability: Self-report.	100% would recommend intervention to those in need, 81 % wanted to continue getting the text messages after conclusion of the study, 95 % satisfied with the intervention overall / Highly acceptable
						Feasibility: Response rate.	58% average response rate to SMS / Moderately feasible.

**SMS +PC**	Haberer 2016	RCT	≥18yrs, HIV+ on ART, UGA. n=21(Scheduled SMS) n=20 (Triggered SMS) n=21(Ctrl)	HIV	Scheduled SMS: 1M daily SMS, 2M weekly SMS, 6M SMS sent to patient and support if needed. Triggered SMS; SMS sent to patient and support if no signal received from monitor.	ART: MEM	Significant difference in scheduled SMS intervention compared to control (11.1% increase in adherence, 48-h and more than 96-h lapses were less frequent (IRR=0.6, p value=0.02 and IRR 0.3, P<0.001, respectively). Similar adherence in triggered SMS vs control group. / Effective.
						ART: VL	No significant differences in HIV RNA suppression among study arms (p value = 0.14). 47/62 participants virally suppressed at 3 and 9M / No impact.
**SMS**	Hardy 2011	RCT	HIV+ 18+ yrs, USA. n=12(SMS) n=14(Beeper)	HIV	SMS vs. beeper reminders.	ART in APs: Composite score (MEM+ pill count + self-report).	Higher adherence at 6W. (MD=27.1(7.6-46.6), p =0.009) / Effective.
						ART in APs: MEM.	Increased adherence. (MD=33.4(14.1-52.6), p = 0.002) / Effective.
						ART in APs: Pill count.	No difference. (MD=13.7(-6.7-34.1), p = 0.153) / No impact.
						ART in APs: Self-report.	No difference. (MD=20.2 (-1.8-42.1), p = 0.069) / No impact.
**SMS**	Jeffries 2016	RCT	15-24yrs, HIV+, USA. n=91(Int) n=45(Ctrl)	HIV	UCARE4LIFE daily moblie text messageing intervenetion over 3M to improve HIV care among youth	ART: VL	Significant difference in ART adherence in intervention vs control among non-adherent/new to ART at baseline (6M p=0.03). / Effective. No sig difference in those on ART at baseline (6M p=0.119) /No impact.
						Acceptability: Self-report.	Mean score = 8.44 (SD=2.45) on 10 point Likert Scale for appointment reminder SMS./ Highly acceptable
**PC**	Kalichman 2011	RCT	HIV+ 18+ yrs, USA. n=21(Int) n=19(Ctrl)	HIV	PC counselling.	ART in NAPs: Pill count.	No difference at 4M (F(1,36)=3.32, p<0.07) / No impact.
						Feasibility: Completion rate.	99% completion rate / Highly feasible.
**SMS**	Kassaye 2016	RCT.	HIV+ pregnant women, KEN n=280 (Int) n=270 (Ctrl)	HIV	3 to 6 weekly SMS (ART reminders, motivational, PMTCT, child health & nutrition).	ART in TNPs: Self-report.	Nonsignificant difference in adherence to ART at 34-36W gestation between the 2 groups (97.3% vs 99.6%, aRR= 1.25 (0.43-3.60)./No impact. Nonsignificant difference in adherence to ART at delivery between the 2 groups (94.7% vs 100%, aRR=1.01 (0.88-1.16))./ No impact.
**PC**	Kebaya 2014	RCT	HIV+ mothers in PMTCT, KEN. n=75(Int) n=75(Ctrl)	HIV	Bi-weekly PC.	ART in TNPs: Self-report.	Increased adherence (90.7% vs. 72%, p=0.005) / Effective.
						Feasibility: Retention rate.	More likely to remain in treatment at 10W (69.3% vs 37.3%, p<0.001) / Moderately feasible.
**SMS**	Lester 2010	RCT	HIV+ 18+ yrs, KEN. n=273(Int) n=265(Ctrl)	HIV	Weekly SMS.	ART in TNPs: Self-report.	Improved adherence at 6M and 12M: RR=0.81(0.69-0.94) p=0.006 / Effective.
						ART in TNPs: VL.	Lower virological failure (RR=0.84(0.71-0.99) p=0.04) and improved viral suppression (OR=0.71(0.5-1.01) p=0.058) / Effective.
**SMS + PC + cash incentives**	Maduka 2013	RCT	HIV+ at hospital 20+ yrs, NGA. n=52(Int) n=52(Ctrl)	HIV	2 monthly counselling PCs + 2 weekly SMS+ cash incentives	ART in NAPs: Self-report.	Increased adherence (76.9% vs. 55.8%, X2=5.211,p=0.022; RR=0.725(0.55-0.96)) / Effective.
						ART in NAPs: CD4 count.	Improved CD4+ count (193-->575 cells/mL vs. 131-->361.5 cells/mL; p=0.007) / Effective.
**SMS + PC**	Mbuagbaw 2012	RCT	HIV+ 21+ yrs, CMR. n=101(Int) n=99(Ctrl)	HIV	Weekly motivational SMS. Phone number to call for support.	ART in PVLA: Self-report.	No difference. (RR=1.06(0.89-1.29); p=0.542) / No impact.
						ART in PVLA: Pharmacy Refills.	No difference at 6 months (MD=0.1(-0.23-0.43); p=0.617) / No impact.
						Acceptability: Self-report.	91.1% believed SMS reminders helped; 65% were satisfied; 81.2% would recommend to a friend / Highly acceptable.

**SMS**	Moore 2015	RCT	HIV+ bipolar 18+ yrs, USA. n=25(Int) n=25(Ctrl)	HIV	SMS reminders.	ART in PVLA: MEM.	No difference. (86.2% (SD= 12.7) vs. 84.8% (SD= 18.1); p=0.95; d=0.01) / No impact.
**SMS**	Nsagha 2016	RCT.	HIV+ on ART, 18+yrs, CMR n=45 (Int) n=45 (Ctrl)	HIV	4 weekly educative SMS over 1M.	ART in PVLA: Self-report.	Nonsignificant difference in adherence to ART at 1M between the 2 groups (64.4% vs 44.2%, p=0.056)/ No impact.
						Acceptability: Self-report.	57.8% wished the SMS to continue/ Moderately acceptable
**SMS**	Pop-Eleches 2010	RCT	HIV+ 18+ yrs, rural KEN. n=142(Daily SMS) n=147(Weekly SMS) n=139(Ctrl)	HIV	Daily or weekly SMS.	ART in PVLA: MEM.	Increased adherence in weekly SMS group over 48W (53% vs. 40% p=0.03) / Effective.
						ART in PVLA: MEM.	No difference between daily SMS group and Ctrl (41% vs. 40% p=0.92) / No impact.
**SMS + cash incentive**	Rana 2016	UnCtrlled trial.	HIV+, 18+yrs, USA. n=32	HIV	Bi-directional weekly SMS appointment reminders, daily ART reminder & supportive messages.	ART in PVLA: Undetectable VL	Significant increase in the number of participants with undetectable VL at 6M (25 vs. 18, p=0.002)/ Effective.
						ATT treatment: Attendance rate.	20/32 completed all visits within 6M study period.
**SMS**	Sabin 2015	RCT	HIV+ 18+ yrs, in CHN. n=63(Int) n=56(Ctrl)	HIV	SMS reminders via MEM + adherence counselling.	ART in PVLA: MEM.	Increased adherence over 6M (82% vs. 51.8%; RR=1.59(1.21- 2.10), p<0.001) / Effective.
						ART in PVLA: VL.	No difference in undetectable VL (93.6% vs. 98.2%, p=0.218) / No impact.
						ART in PVLA: CD4 count.	Higher mean change in CD4 count (52 vs 28 cell/μL, p=0.297) / No impact.
**PC + MMS.**	Shet 2014	RCT	HIV+ 18-60 yrs, IND. n=315(Int) n=316(Ctrl)	HIV	Weekly automated motivational voice call, followed by weekly MMS.	ART in TNPs: VL.	No difference. (Number of virological failures: 15.6% vs. 15.5%. Time to virological failure: aHR= 0.96(0.65-1.43), p= 0.85) / No impact.
						ART in TNPs: Pill count.	No difference. (27% vs. 21.7%; aIRR=1.24(0.94-1.63), p=0.13) / No impact.
						Feasibility: PC received.	86% of calls received by patients / Highly feasible.
**SMS**	Walsh 2012	UnCtrlled trial.	HIV+ Adults on ART, GBR. n=14	HIV	Pill-box w/ MEM + weekly SMS wrt med taking + up to 3 late dose SMS reminders.	ART in APs: Self-report + MEM.	99.5% baseline adherence, 98% at 24W. No difference in missed doses (4.8% in 0-12W; 6.3% in 13-24W)
						Acceptability: Self-report.	64% satisfied, 50% found SMS & system useful. 55% found reminders irritating / Moderately acceptable.
**SMS**	Lim 2008	Quasi-experimental: HxCtrl.	STI clinic, NZL. n=293(Int) n=303(HxCtrl)	CT	SMS to contact clinic for CT test result.	TAT: Time from test to treat.	No change in median time to treat (3 days vs. 4 days, t = - 1.3, p<0.1) / No impact.
**SMS**	Menon-Johansson 2006	Quasi-experimental.	At clinic w/untreated CT, GBR. n=28(Int) n=21(Ctrl)	CT	SMS to contact clinic for CT test result.	TAT: Time from test to diagnosis & test to treat.	Shorter mean time to diagnosis. (7.9 days vs. 12.5; p<0.001) Shorter median time to treat. (8.5 days vs. 15; p=0.005) / Effective.
**SMS+PC**	Barnabas 2016	RCT	16-49 yrs,, ZAF & UGA. n=284(Int) n=224(Ctrl)	HIV	SMS promoting male circumcision 3W, 6-7W after tested negative. Follow-up phone call 1M & 2M following SMS reminders.	PB: Self-report.	Significant difference in reaching outcome at 3M (Intervention vs clinic referral); 48% (RR=1.72 95% CI 1.36-2.17, p values < 0.0001) in SMS reminder group and 47% (RR=1.67, 95%CI 1.29-2.14, p value = 0.0001) in lay counsellor follow-up achieved MC at 3M / Effective
**SMS + MMS.**	Cornelius 2013	UnCtrlled trial.	African-Americans age 13-18, USA. n=40	HIV	HIV-prevention SMS + knowledge question for	PB: Self-report.	Improved condom attitudes & HIV knowledge (83% vs.78% correct answers) / No impact.

					3W.	Acceptability: Self-report.	97% satisfied w/ number of SMS. 86% reported SMS not interfering w/ daily activities/ Highly acceptable.
						Feasibility: Completion rate.	100% at pretest; 90% at 3M FU/ Highly feasible.
**PC**	DiClemente 2014	RCT	High-risk African-American women 14-20 yrs, USA. n=342(Int) n=359(Ctrl)	CT	PC w/ prevention messages every 8W.	PB: % diagnosed w/ CT or GC.	Fewer participants diagnosed w/ CT & GC (90 vs. 104; RR = 0.5 (0.28-0.88), p=0.02. 48 vs. 54; RR = 0.4 (0.15-1.02), p=0.06) / Effective.
						PB: Self-report.	Higher condom use (MD=0.08(0.06 to 0.10) p=0.04) / Effective.
**SMS + cash incentive**	Juzang 2011	Non-randomized Ctrlled trial.	African-American men 16-20 yrs, USA. n=30/group	HIV	3 weekly SMS HIV prevention messages + $40 for completion.	PB: Self-report.	No statistical difference in % of protected sex. Higher awareness of sexual health / No impact.
						Feasibility: Retention rate.	20 (67%) retained in Ctrl & 19 (63%) in SMS group after 2nd FU / Moderately feasible.
**SMS**	Odeny 2014	RCT	Circumcised male at clinic, 18+ yrs, KE. n=600(Int) n=600(Ctrl)	HIV	Daily SMS for 1wk + SMS on days 8, 14, 21, 28, 35, 41, and 42 post-procedure.	PB: Self-report.	Abstention of sexual activity before 42-day follow up: 139/491(28.3%) vs. 124/493(25.2%) in control group (RR=1.13(0.91-1.38), p=0.3)/ No impact.
**SMS**	Reback 2015	UnCtrlled trial.	MSM drug users 18-65 yrs, USA. n=52	HIV	Daily SMS for 2W to reduce risky sexual behaviours.	PB: Self-report.	Reduction in anal sex (6.9 vs. 2.6, t97=2.82, p<0.05) and unprotected anal sex (1.8 vs. 0.5, t97=2.19, p<0.05) in past 2M/ Effective.
**PC**	Belzer 2015	RCT	HIV+ 12-29 yrs, USA. n=19(Int) n=18(Ctrl)	HIV	PC 1hr from time to take medication.	Acceptability: Self-report.	94% satisfied w/ call length and 81% would continue receiving calls / Highly acceptable.
						Feasibility: Retention rate.	63% retention rate / Moderately feasible.
**SMS**	Dean 2012	Feasibility study.	HIV+ at antenatal clinics, ZAF. n=7	HIV	SMS support group+ inquiries answered by physicians.	Acceptability: Self-report.	Overall satisfaction.
						Feasibility: Self-report.	SMS easily kept confidential.
**SMS**	Roth 2014	Feasibility study.	Sex workers 18+ yrs, USA. n=26	HIV	Cell phone diaries to collect info about sexual events.	Acceptability: Self-report.	Cell-phone electronic dairies to collect sensitive information acceptable (84.6%)/ Highly acceptable.
						Feasibility: Completion rate.	90.3% surveys completed / Highly feasible.
**SMS**	Georgette 2016	Feasibility study.	≥18yrs, HIV+, ZAF. n=88	HIV	Weekly SMS reminders to increase ART adherence and appointment reminders	Acceptability: Self-report.	92% would recommend SMS program to a friend, 90.9% said frequency of SMS was just right, 2/88 felt the SMS program slightly violated their privacy. 97.7% reported it helped them remember to take medication. 77.3% agreed that it helped them remember appointments. / Highly acceptable
**SMS**	Reid 2014	Cross-sectional study.	HIV+, BWA. n=42(Int) n=41(Ctrl)	HIV	SMS ARV pick-up reminder.	Acceptability: Self-report.	SMS helpful 93% (Int) vs. 58% (Ctrl) (p<0.001). SMS may lead to serostatus disclosure 10% vs. 56% (p<0.001). 95% satisfied w/ appointment scheduling. 90% would continue receiving SMS / Highly acceptable.
**PC**	Bauermeister 2014	Feasibility study.	MSM 18-30, USA. n=124	HIV	IVRS: microbicide use.	Feasibility: Self-report.	75.5% reported no problems using IVRS / Highly feasible.
**SMS + MMS.**	Cornelius 2011	Feasibility study.	African-Americans age 13-18, USA. n=12	HIV	HIV-prevention SMS+knowledge question for 3W.	Feasibility: Response rate.	80% response rate/ Highly feasible.

Note: Int= intervention; Ctrl= control; HxCtrl= historical control; PB= preventative behaviors (i.e. risk reduction); PN= partner notification; TAT= turnaround time; ATT= attendance rate; ART= ART adherence; NAPs= non-adherent patients; AP= adherent patients; PVLA= Patients with various levels of adherence; TNPs= Treatment naive patients; VL= viral load; CD4= CD4 cell count; PC= phone call; FB= Facebook.
