# Supplementary material for: Do digital innovations for HIV and sexually transmitted infections work? Results from a systematic review (1996-2017)
Source: BMJ Open. 2017 Nov 3;7(11):e017604. doi: 10.1136/bmjopen-2017-017604 (PMC5695353; doi:10.1136/bmjopen-2017-017604)
Supplement: Supplementary file 3 [file bmjopen-2017-017604supp003.pdf]

Appendix 3. Table of studies by innovation (in rows) and by outcomes (in columns)

	Outcome* / *Digital Innovation	***Attendance rate***	***ART adherence***	***Risk reduction***	***Partner notification***	***Turnaround time***	***Self-care***	***Feasibility*†**	***Acceptability*†**
Number of Studies by Type of Digital Innovation	***mHealth Innovations (SMS/phone call only)***	*30**	*24*	*6*	*0*	*2**	*0*	*5*	*2*
	Internet-based m/eHealth Innovations	*6*	*4*	*5*	*0*	*0*	*1*	*4*	*1*
	Combined innovations	*1*	*1*	*0*	*2*	*0*	*1*	*3*	*1*

*Note: *1 study evaluated both attendance rate and turnaround time and was counted as part of the 30 studies on attendance rate. † studies reporting feasibility and acceptability as secondary outcomes are counted elsewhere in the table depending on primary outcome.*
