# Supplementary figures and images for: Do digital innovations for HIV and sexually transmitted infections work? Results from a systematic review (1996-2017)
Source: BMJ Open. 2017 Nov 3;7(11):e017604. doi: 10.1136/bmjopen-2017-017604 (PMC5695353; doi:10.1136/bmjopen-2017-017604)

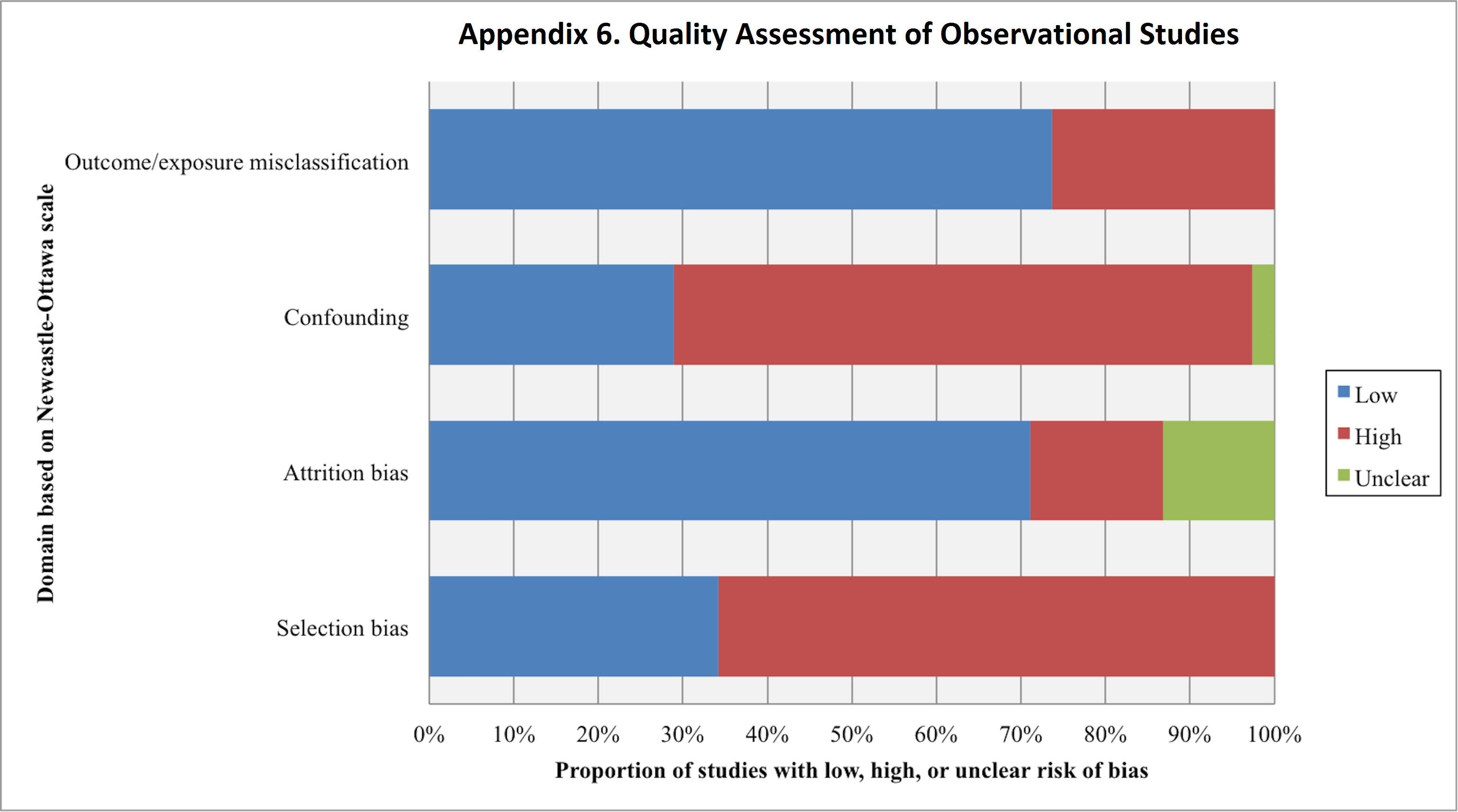

Supplement: Supplementary file 6 [file bmjopen-2017-017604supp006.jpg]

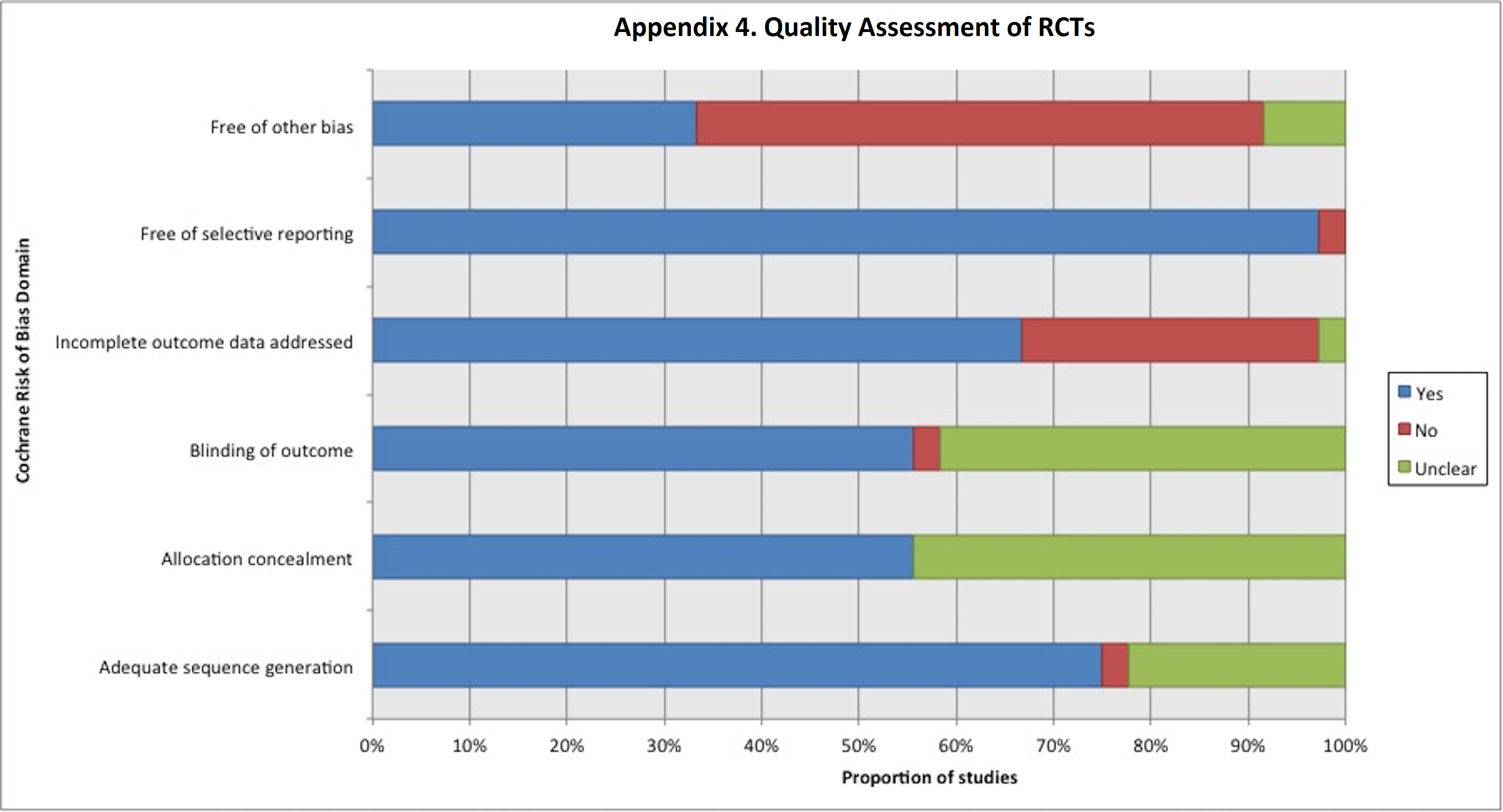

Supplement: Supplementary file 4 [file bmjopen-2017-017604supp004.jpg]

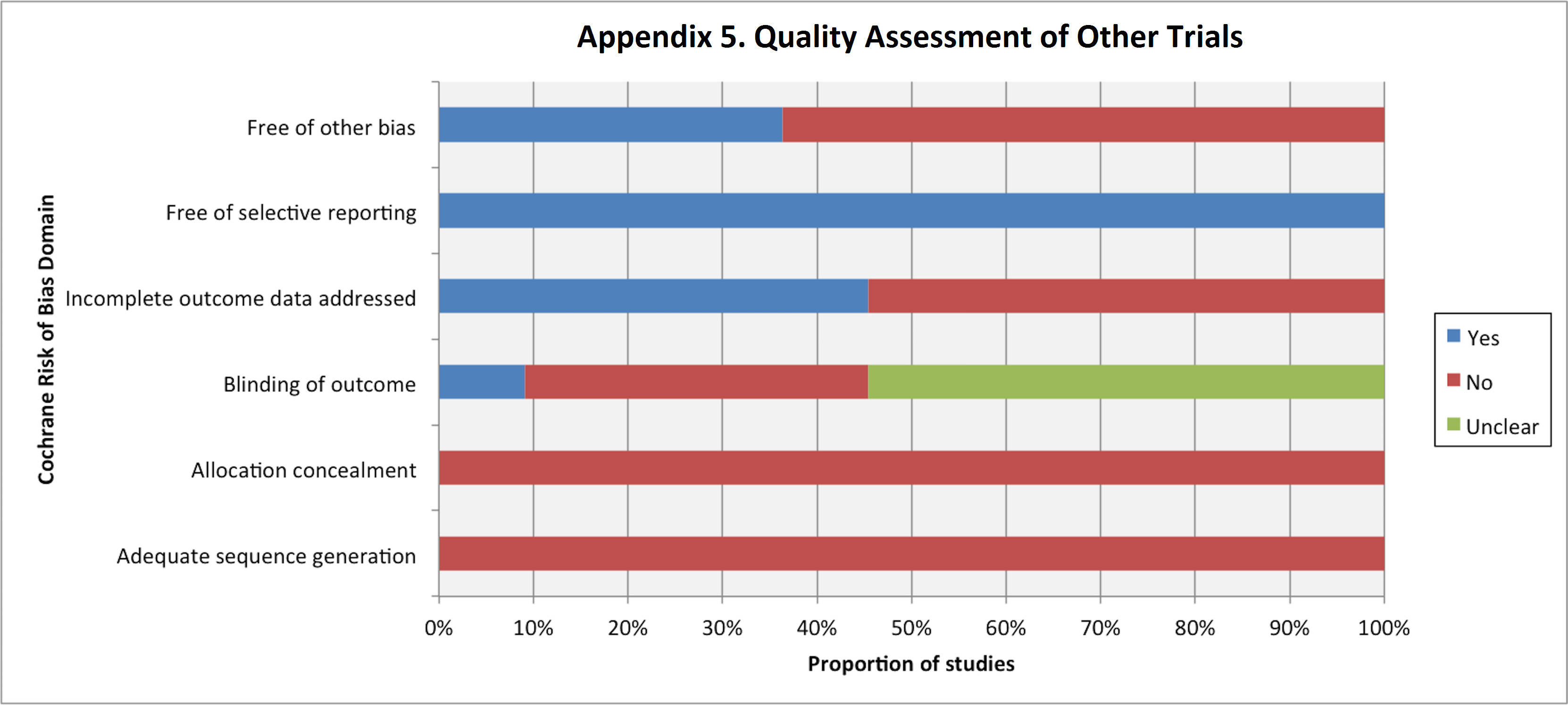

Supplement: Supplementary file 5 [file bmjopen-2017-017604supp005.jpg]
