# Supplementary material for: Do digital innovations for HIV and sexually transmitted infections work? Results from a systematic review (1996-2017)
Source: BMJ Open. 2017 Nov 3;7(11):e017604. doi: 10.1136/bmjopen-2017-017604 (PMC5695353; doi:10.1136/bmjopen-2017-017604)
Supplement: Author's manuscript [file bmjopen-2017-017604.draft_revisions.pdf]

BMJ Open is committed to open peer review. As part of this commitment we make the peer review history of every article we publish publicly available.

When an article is published we post the peer reviewers' comments and the authors' responses online. We also post the versions of the paper that were used during peer review. These are the versions that the peer review comments apply to.

The versions of the paper that follow are the versions that were submitted during the peer review process. They are not the versions of record or the final published versions. They should not be cited or distributed as the published version of this manuscript.

BMJ Open is an open access journal and the full, final, typeset and author-corrected version of record of the manuscript is available on our site with no access controls, subscription charges or pay-per-view fees (http://bmjopen.bmj.com).

If you have any questions on BMJ Open's open peer review process please email editorial.bmjopen@bmj.com

BMJ Open

Do Digital Innovations for HIV and Sexually Transmitted Infections work? Results from a Comprehensive Systematic Review (1996-2017).

Journal:	*BMJ Open*
Manuscript ID	bmjopen-2017-017604
Article Type:	Research
Date Submitted by the Author:	11-May-2017
Complete List of Authors:	Daher, Jana; Research Institute of the McGill University Health Centre<br>Vijh, Rohit; Research Institute of the McGill University Health Centre<br>Linthwaite , Blake; Research Institute of the McGill University Health Centre<br>Dave, Sailly; Research Institute of the McGill University Health Centre<br>Kim, John; National HIV/AIDS Labs, Public EHalth Agency of Canda<br>Dheda, Keertan; University of Cape Town<br>Peter, Trevor; Clinton Health Access Initiative (CHAI)<br>Pai, Nitika; McGill University, Medicine
**Primary Subject Heading**:	HIV/AIDS
Secondary Subject Heading:	Health services research, Global health
Keywords:	mHealth/eHealth, Innovations, HIV, Sexually transmitted Infections, systematic reviews

SCHOLARONE™
Manuscripts

For peer review only

Do Digital Innovations for HIV and Sexually Transmitted Infections work? Results from a Comprehensive Systematic Review (1996-2017).

Jana Daher[1], MSc (PH), Rohit Vijh[1], BSc, Blake Linthwaite[1], BSc, Sailly Dave[1], BSc,

John Kim[2], PhD, Keertan Dheda[3], MBBCh, PhD, Trevor Peter[4], PhD,

Nitika Pant Pai[1,5], MD PhD

Affiliations:

[1] Division of Clinical Epidemiology, Research Institute of the McGill University Health Centre, Montreal, Canada.

[2] Chief, National HIV/AIDS Labs, National Labs, Winnipeg, Canada.

[3] Professor, Department of Pulmonology, UCT Lung Institute, University of Cape Town, South Africa.

[4] Scientific Director, Diagnostics, Clinton Health Access Initiative (CHAI), Boston, USA.

[1,5] Associate Professor, Department of Medicine, McGill University, Montreal, Canada.

Corresponding author:

Nitika.pai@mcgill.ca

Address: 687 Pine Avenue West Division of Clinical Epidemiology, McGill University Health Center, Bureau V2.19, Montreal, Canada

Telephone number: 514-934-1934 x44729

Keywords: mHealth/eHealth; Innovations; HIV; Sexually transmitted Infections; systematic reviews, meta-analyses.

For peer review only

ABSTRACT

Objective

To control HIV/STI epidemics globally, in overburdened health systems, with high service delivery costs, and a lack of patient engagement, digital innovations on internet/mobile phones offer a potential solution.

To evaluate feasibility and impact of all digital innovations for all HIV/STIs.

Design: Systematic review
Setting/Participants: All settings/ all participants.

Intervention

Digital innovations were sub-classified: a) Mobile health based (mhealth: SMS (short message service) /phone calls), b) Internet based mobile and/or electronic health (m/eHealth: social media, avatar-guided computer programs websites, mobile applications, streamed soap opera videos), and c) combined innovations (SMS/phone calls and internet-based m/eHealth)

Primary and secondary outcome measures: Feasibility, Acceptability, Impact.

Methods:

Databases- MEDLINE via PubMed, Embase, Cochrane CENTRAL, and Web of Science were searched. Data were abstracted; heterogeneity was explored; random effects subgroup analysis was performed in only one group that reported consistent measures.

Results:

99 studies were reviewed- 63 (64%) from America/Europe, 36 (36%) from Africa/Asia.
Break up: Mobile health based innovations-70% (69/99); internet based innovations: 21% (21/99); combined innovations: 9% (9/99).79% (79/99) clinical trials; 84% (83/99) evaluated impact.
Majority of digital innovations were highly acceptable (26/31; 84%) feasible (20/31; 65%), and impacted measures-
Mobile health (SMS) significantly improved ART adherence (pooled OR=2.15 [95%CI: 1·18, 3·91]), clinic attendance (pooled OR=1.76 [95%CI: 1·28, 2·42]).
Internet-based innovations improved clinic attendance (6/6; 100%), ART adherence (4/4; 100%), reduced risk (5/5; 100%) and improved self-care (1/1; 100%).
Combined innovations increased clinic attendance, ART adherence, partner notification, and self-care.

Conclusion

Overall, digital innovations were acceptable, feasible, and favorably impacted measures.

A recent trend towards a greater use of internet based and combined (internet and mobile) innovations was noted. Cost data were limited.

Findings will appeal to stakeholders aiming to integrate digital innovations in their HIV/STI strategies/programs globally.

For peer review only - http://bmjopen.bmj.com/site/about/guidelines.xhtml

Funding: Grand Challenges Canada, FRSQ.

Strengths of the review

- A Comprehensive and up-to-date systematic review/meta-analysis.
- All digital innovations for HIV/STIs and all health outcomes were reviewed.
- Cochrane methodology and PRISMA guidelines followed.
- Critique of study quality conducted.
- A subgroup analyses performed when similar outcomes were reported.

Limitations of the review

- Cost-effectiveness data from the high HIV/STIs burden regions (i.e., Sub-Saharan Africa and Southeast Asia) were limited.
- Limited data from Sub-Saharan Africa and Southeast Asia (29%, 29/99).
- Limited evidence on other STIs (other than HIV) (18/99, 18%).
- A lack of integrated online impact metrics to evaluate internet-based eHealth innovations.
- Studies with small sample sizes, low power, insufficient follow-up time (e.g. 3 weeks or less) sometimes provided contradictory results when objective and subjective metrics evaluated the same outcome.

For peer review only

INTRODUCTION

HIV/STI infections remain a public health concern worldwide. A million new HIV/STI infections are acquired every day, and their cumulative disease burden exceeds 500 million infections.[1-5] Regarding HIV, many countries are working to meet the UNAIDS 90-90-90 targets;[6] however, structural and societal barriers such as stigma, low socio-economic status, and geographical isolation, impede access to quality care for marginalized populations that are disproportionately impacted by the HIV/AIDS epidemic.[7-8] A lack of timely testing and poor retention in care impairs efforts to control HIV/STIs.[7 9-10] To improve early testing, linkage and retention in care, health care systems around the world are seeking solutions for population engagement, awareness, and education. Providing efficient care to hard-to-reach populations, while plugging gaps in health care service delivery, is urgently needed.[11-12]

The World Bank estimates that globally, 96% of the world's population and 70% of the world's poorest have access to a mobile phone.[13] Of seven billion, two billion (30%) individuals own a smartphone and approximately 50% of mobile phone users access the internet through their phones.[14-15] Technological access has created a portal for social media and other internet-based health interventions.[16] The rapid diffusion of mobile phones and internet technologies are prime drivers of this disruption in health care service delivery, through a phenomenon aptly titled, the creative destruction of medicine.[17]

Digital innovations such as electronic health (eHealth), mobile health (mhealth), and combined innovations offer promising solutions to improve health service delivery. Ehealth encompasses non-internet and internet-enabled mHealth as well as other internet-based health interventions. These innovations, together with expanded mobile and internet networks, global connectivity, and affordability, present opportunities to change the future landscape of health care service delivery. In recent years, visionary foundations (*Grameen*, *Bill and Melinda Gates Foundation*, *UNAIDS*, *Vodafone)* have increased funding and created opportunities for innovative thinking in health, as demonstrated by ninety-five countries which have evaluated digital innovations to date.[11] This is most evident in under-resourced settings where low-cost, sustainable solutions to solve complex global health challenges are much in demand.[18]

The early use of digital innovations was evident in non-communicable diseases, which gained popularity in infectious disease.[19] In the field of HIV/STIs, a study published in the Lancet was the first to demonstrate the effectiveness of mHealth-based SMS innovations on adherence to antiretroviral therapy (ART).[20] As novel digital innovations, strategies and programs continue to be developed and tested, many smaller reviews and systematic reviews were published. However, a vast majority only evaluated a single innovation (e.g. SMS, social media), focused on one or two outcomes, and restricted their populations to select groups (people living with HIV (PLHIV), pregnant women, adolescents, men who have sex with men (MSM)).[21-27] These reviews failed to provide a comprehensive summary of all innovations in one place.

Due to the rapid expansion of the field of digital innovations, and with the increased popularity of combined innovations in recent years (beginning 2013), a need for a comprehensive up-to-date synthesis on all innovations for HIV/STIs was felt. Our objective was therefore to generate a high quality overview/systematic review, following Cochrane methods and guidelines, to

For peer review only - http://bmjopen.bmj.com/site/about/guidelines.xhtml

summarize all digital innovations across all populations and outcomes. This review compiles and evaluates all existing data, tailored to inform researchers, policy makers and key stakeholders in the field of HIV/STI on decisions regarding implementation and scale-up.[11]

METHODS

PRISMA (Preferred Reporting Items for Systematic Reviews and Meta-Analyses) guidelines were followed for the review.[28]

Data Sources and Searches

We searched MEDLINE via PubMed, Embase, Cochrane CENTRAL, and Web of Science for a 21-year period from Feb 1996 up to March 2017, with no language restrictions.

Search Strategy

For MEDLINE: (#1 ("HIV" [MeSH] OR "acquired immunodeficiency syndrome" [tiab]), OR #2 (sexually transmitted infections [mh] OR sexually transmitted disease* [tiab]), AND #3 ("mHealth" [tiab] OR "mobile health" [tiab] OR short messag* [tiab] OR "eHealth" [MeSH] OR "telemedicine" [MeSH] OR social medi* [tiab] OR "mobile applications" [tiab]) (Refer to Appendix 1).

Study Selection

Two reviewers independently screened and evaluated citations for eligibility (JD & RV) and two others (BL & SD) independently assessed quality. A senior reviewer was consulted (NPP) for discordance.

Eligibility Criteria

Any clinical trials or observational study designs that evaluated any digital (e/mHealth) technology with any reported outcomes (Refer Figure 1) were included.

Data Abstraction

Two reviewers (RV, JD) independently abstracted all the data. A pre-piloted data abstraction form, was used to abstract the following items: study design, study population, sample size, digital innovation type, HIV/STIs, outcome measures (e.g. impact, acceptability and feasibility), and metrics (e.g. attendance rate, completion rate, satisfaction) (Refer to Appendix 2). We referred to a previously published framework to define and further classify the following metrics for impact, acceptability, and feasibility.[29]

Subgroup Pooled Analyses

We classified study designs and then classified digital innovations into three groups:[30]

a) mHealth (SMS and phone calls only; i.e. non-internet based);
b) Internet-enabled mHealth and other internet-based eHealth (mobile application, website, online campaign, streamed soap opera videos, avatar-guided computer programs);
c) Combined innovations (innovations that combined both mHealth (SMS/phone calls) with internet enabled m/eHealth).

Only one subgroup reported similar outcomes which could be pooled, SMS and phone calls, for the following outcomes: a) clinic attendance with SMS; and b) ART adherence via Medication

For peer review only - http://bmjopen.bmj.com/site/about/guidelines.xhtml

Event Monitoring System (MEMS) caps, with SMS. We pooled these outcomes using a random effects subgroup analysis. Given the diversity in the sample populations between studies, we used the Dersimonian and Laird random effects frequentist model, weighted by study sample to calculate a pooled effect. We generated forest plots for visual representation of heterogeneity and pooled odds ratios (OR) with 95% confidence intervals (95% CI). We performed all statistical analyses using Stata/IC, version 13 (StataCorp, College Station, Texas USA).[31]

Narrative Analysis

We narratively described all other data using as follows:

Digital innovations were classified into the following groups based on the strength of evidence: high/strong evidence (metrics at 75-100%), moderate evidence (51-74%), and low/weak evidence (50% or less).

Acceptability: Acceptability was defined as the receptivity in using digital innovations.

Feasibility: Feasibility was defined as the perceived convenience in using digital innovations. It was reported with various metrics: completion, retention, response and referral rates.

Impact: Impact was defined as a statistically significant improvement in measured outcomes compared to a comparator group (i.e. control group or baseline observations). The metrics used to evaluated impact were: A) attendance rate, B) ART adherence, C) risk reduction, D) self-care, E) partner notification. Impact measures were evaluated on two criteria: effect size and precision. Effect size was assessed when data on a comparator group was made available. Precision of the effect estimate was assessed whenever reported, as it reflects the variance or spread of results.

Quality Assessment

We assessed study quality for both clinical trials and observational studies. We used the Cochrane Risk of Bias Tool for trials, and Newcastle-Ottawa quality assessment scale for observational studies.

RESULTS

Of 4252 citations identified through our extensive search, 792 were selected for full-text screening, and 99 citations met our inclusion criteria and were included in this review for evidence synthesis (Refer: Figure 1).

Study characteristics

By geographical location, 37% (37/99) of studies were conducted in North America, 26% (26/99) in Sub-Saharan Africa, 24% (24/99) in Europe, 7% (7/99) in Oceania, 3% (3/99) in Southeast Asia, and 2% (2/99) in South America.

By study design, the majority were trials: 38% (38/99) were RCTs, 16% (16/99) uncontrolled trials, and 1% (1/99) non-randomised controlled trials. Others included quasi-experimental studies, of which many used historical controls (24%, 24/99), and observational studies (i.e. cross-sectional and feasibility studies) (20%, 20/99).

For peer review only

For peer review only - http://bmjopen.bmj.com/site/about/guidelines.xhtml

HIV was the most frequently reported infection (74%, 73/99 studies), followed by chlamydia/gonorrhea (CT/GC) (10%, 10/99). Combinations of HIV with STIs (e.g., syphilis) (8%, 8/99), human papillomavirus (HPV) (4%, 4/99) and hepatitis A/B/C (HBV) (4%, 4/99) were also reported.

In terms of study populations, people living with HIV were prominent across studies (42%, 42/99) followed by other high-risk groups (i.e. MSM/bisexual men, drug users, pregnant women/mother-infant pairs, African-Americans, sex workers, and visible minorities) (28%, 28/99), general clinic attendees (16%, 16/99), CT/ HBV infected individuals (4%, 4/99), and residents of a specific area (9%, 9/99).

Innovations

Digital innovations were documented across the spectrum.

MHealth innovations (SMS/phone calls only) were evaluated in 70% (69/99) of studies.[20 32-99] 72% (50/69) were SMS-based and 28% (19/69) used phone calls or a combination of both (Refer to Figure 2).

Internet-enabled mHealth and other internet-based eHealth innovations were evaluated in 21% (21/99) of studies.[100-120] These innovations consisted of many different forms: social media and online campaigns (9/21), avatar-guided computer programs (2/21), mobile applications (5/21), combination of social media and websites (2/21), websites (1/21), telemedicine services (1/21) and streamed soap opera videos (1/21) (Refer to Figure 2).

Combined innovations were evaluated in 9% (9/99) of studies.[121-129] Innovations consisted of: SMS + websites/ interactive websites (4/9), SMS + mobile application (3/9) and SMS + social media (including online campaigns) (2/9). (Refer to Figure 2).

Measures and Metrics

A vast majority (84%, 83/99) of studies focused on impact measure and metrics, while about 12% (12/99) focused only on feasibility, and the remaining 4% (4/99) on acceptability. Within impact measures, metrics such as clinic attendance rates were reported in 45% (37/83) of studies, followed by ART adherence at 35% (29/83), HIV/STIs risk reduction behaviors at 13% (11/83), turnaround time from testing to treatment at 2% (2/83), partner notification at 2% (2/83), and self-care at 2% (2/83).

Subgroup Pooled Analyses

It was possible to perform subgroup analyses on outcomes that were consistently documented: clinic attendance in 14 quasi-experimental studies that used SMS reminders and MEMS-based ART adherence in 4 RCTs evaluating SMS. The pooled estimate for the impact of SMS reminders on attendance rates was 1.76 [95%CI: 1·28, 2·42] (Refer to Figure 3 A). The pooled estimate for the impact of SMS on ART adherence tracked via MEMS caps was also significant, OR= 2.15 [95%CI: 1·18, 3·91] (Refer to Figure 3 B).[32 47-48]

For peer review only - http://bmjopen.bmj.com/site/about/guidelines.xhtml

Narrative Analysis

Impact

Non-internet based mHealth (SMS/PC only)

Of 69 studies, positive results were reported for the following outcomes: clinic attendance (63%, 19/30 studies, of which 84% reached statistical significance); ART adherence (63%, 15/24 studies, of which 93% reached statistical significance); turnaround time from testing to treatment (67%, 2/3 studies). However, SMS reported a limited effect on risk reduction behaviors (3/7, 43%).

Internet-based m/eHealth:

Studies evaluating internet-based eHealth innovations (21/99) reported results that were largely in favor of the following innovations: social media-based interventions for clinic attendance; avatar-guided and mobile applications for ART adherence; social media, avatar, and soap opera videos for risk reduction behaviors; mobile app for self-care.

Social media contributed to higher testing uptake rates in all studies (6/6, 100%). A social media-based campaign increased HIV testing by 252% (n= 1500; 19% from baseline 5.4%, p<0·01) and Syphilis testing by 248% (18·8% from baseline 5·4%, p<0·01), while another campaign increased HIV testing by 52% compared to control (n=625; 63.7% vs. 42% in controls, OR=2.9 [95%CI: 1.8-4.7]).[100 115] Four campaigns guaranteed rapid in-home HIV testing for all those who requested it online.[100-101 108 111 116]

Avatar-guided programs and mobile applications improved ART adherence in all studies (4/4, 100%). Statistically significant outcomes were reported in 2/4 programs (50%). These were: a) A personalized avatar-guided computer program improved adherence (n=240; p=0·046); b) a mobile application with immunosuppression graphs and medication reminders lowered viral load (n= 28; p=0·023) and improved adherence (p=0·03) as well.[102 104] In the other two studies, an avatar-guided program improved viral suppression and a mobile application allowed for 100% adherence, but these were underpowered to detect a significant effect (n=76 and n=28, respectively).[107 110]

Social media, avatar and soap opera videos were successful at reducing risky sexual behavior in 100% of studies (5/5). However, significant results were reported in only 3/5 studies: a) Social media-based interventions decreased unprotected sex acts by 65% (n=31; 3·11 vs. baseline 8·96, p=0·042); b) soap opera videos on HIV prevention reduced condomless sex by 78% (n=117; 78% reduction from baseline, p<0·001);[103 106]c) An avatar-guided computer program also lowered the odds of HIV transmission (n=240; OR= 0·46, p=0·012).[102-103 106] Even in two underpowered studies, social media-based interventions led to 40% and 67% higher condom uptake (n=70 and n=50, respectively).[105 117]

A mobile app increased self-care in the sole study in this category (1/1; 100%). A significantly higher self-care performance among chronic HBV-infected individuals was reported compared to controls (n=53; p=0.001).[112]

For peer review only - http://bmjopen.bmj.com/site/about/guidelines.xhtml

Combined innovations:

Studies evaluating combined innovations (9/99) showed success of social media + SMS in increasing clinic attendance and partner notification; interactive websites + SMS in improving ART adherence; and mobile app + SMS in increasing self-care. Among the five impact studies, 80% (4/5) reported a favorable outcome. An online campaign with SMS services increased CT, GC, and HIV tests uptake by 41%, 91%, and 190%, respectively;[123] an interactive website with SMS reminders improved ART adherence in drug-users (n=20; p=0·02);[121] a social media-based partner notification with SMS increased notified contacts by 144% (63.5% in 2011 from baseline 26% in 2010);[126] and a mobile app with SMS significantly improved self-care performance in HIV-infected individuals compared to baseline (n=19; p=0.002).[129]

Acceptability and Feasibility

Overall, across studies that assessed acceptability/feasibility, digital innovations were found to be highly acceptable and feasible (75%-100%) in 26/31 and 20/31 studies, respectively. mHealth innovations (SMS/PC only) were highly acceptable and feasible in 81% (13/16) and 75% (12/16) of studies, respectively.

Internet-based m/eHealth innovations were highly acceptable and feasible in 92% (11/12) and 45% (5/11) of studies, respectively. All included innovations (i.e. avatar, mobile app, social media and streamed videos) were highly acceptable.[102-104 106-107] While avatar-guided programs were rated high on feasibility, social media-based strategies were found to be less feasible[101-103]

Combined innovations were highly acceptable and feasible in 67% (2/3) and 75% (3/4) of studies, respectively.[121 124] The innovations that were rated high involved a combination of SMS and interactive websites.

Quality

Studies were individually evaluated on quality criteria and biases were noted. Across trials, losses to follow-up were reported in 31% of RCTs and 55% of quasi-trials. Additionally, biases (i.e. misclassification, desirability/recall bias) were of concern in 58% of the RCTs and 64% of quasi randomized trials (Refer to Appendix 3 & 4).

In observational studies, potential biases observed were confounding (68%) and selection bias (66%) were observed. (Refer to Appendix 5).

DISCUSSION

Summary of findings

Overall, digital innovations reported positive effects on key metrics. We noted a strong positive effect of digital innovations on clinic attendance rates (70%; 26/37), ART adherence (69%; 20/29), risk reduction behaviors (67%; 8/12) and self-care (100%; 2/2). SMS/phone calls were not able to reduce risky sexual behaviours; however social-media based interventions, particularly interactive social media, were effective in reducing risky sexual behaviors. Acceptability was found to be high for all innovations. Feasibility estimates also remained high

For peer review only - http://bmjopen.bmj.com/site/about/guidelines.xhtml

For peer review only

for all innovations, except for social media-based interventions, possibly due to a perceived lack of privacy and confidentiality. Combined innovations may thus offer promise in plugging this feasibility gap, with internet-based innovations compensating for limitations in SMS-only strategies and vice versa.

While mHealth (SMS/phone calls only) innovations were highly effective in improving clinic attendance, ART adherence, and turnaround time from testing to treatment, they did not report on other outcomes. It should be noted that SMS and phone calls alone failed to reduce risky sexual behaviors, which was not surprising as it is challenging to reduce risky behaviors with a prescriptive SMS alone. Population engagement is essential for risk reduction through qualitative research.

While internet-based m/eHealth innovations (social media, avatar-guided computer programs, mobile apps, and soap opera videos) demonstrated positive evidence on impact metrics, not all studies reached statistical significance. Those that failed to report a statistically significant improvement in ART adherence had small sample sizes and were underpowered to detect these outcomes (n=76 vs. n=240), and had less frequent sessions over a shorter evaluation period (2 sessions over 6 months vs. 4 sessions over 9 months).[102 107] For mobile applications, studies which reported significant effects recruited participants with varying level of adherence,[104 110] compared with studies which had high adherence at baseline (≥ 95%) and did not show significance (due to smaller changes in effect). For social media-based campaigns, the two studies that did not reach statistical significance in reducing risky sexual behaviors lacked an interactive component and simply displayed educational material, while the study that showed significant effect engaged the participants by allowing them to contact professional cognitive behavioral therapists via live chat sessions.[103 105 117]

In terms of quality, confounding and selection bias were noted in observational and quasi-experimental studies, and loss to follow-up in some trials. Nevertheless, the overall validity of the findings from this review was not threatened by biases, as a large proportion of our data was derived from trials. Consistent reporting of metrics was lacking, which prevented a comprehensive meta-analysis. While clinical trials were generally high quality, observational studies were medium to low quality. Objectives, end points, metrics, and measures, are equally important in feasibility studies and must well designed to generate high quality evidence.

Our review is an exhaustive assessment of the role of digital innovations in improving prevention and care for HIV/STIs. Our findings resonate with many smaller systematic reviews, which have separately evaluated individual components of digital innovation, such as SMS-based mHealth.[22-23 130-137] Other systematic reviews evaluating social media-based interventions reported similar findings to ours, in improved testing uptake or improvements in sexual health.[25-27 138-139]

Our review makes a valuable addition to the growing body of evidence by highlighting the success of other interactive and engaging innovations such as avatar-guided computer programs, mobile apps, streamed soap opera videos, and combined innovations. These are becoming popular, with their power to engage audiences at many levels. Designing combined innovations offers complementarity with media, methods, platforms, and messaging. This complementarity can encourage participant engagement, and improve prevention and care metrics and measures

For peer review only - http://bmjopen.bmj.com/site/about/guidelines.xhtml

sustainably over time. This is more challenging when only one innovation (e.g. mHealth SMS/phone calls only) is the sole focus.

Caveats and implications for future research

There are some caveats to consider while designing and evaluating digital innovations. Innovations aiming to reduce risky sexual behaviors need to be interactive and tailored to the setting and population, with a deep understanding of patients' needs and preferences.[137 140-141] Any communication with patients should be customized for timing to avoid uptake fatigue. For example, patients may be more responsive to weekly versus daily SMS ART reminders.[32 142] Future research needs to be focussed on tailoring innovations to the context and population, and program objectives.

Study quality is essential to generating meaningful results. Large and representative samples of the underlying population and sound statistical techniques during data analysis can prevent or address selection bias. Exploring reasons for differential loss to follow-up would inform future studies. Wherever possible, a control group should be included to differentiate Hawthorne effect from the effect of the intervention.[143] Trials and impact designs can prevent or reduce confounding. Following checklists, such as the report recently published by the WHO mHealth Technical Evidence Review Group on reporting of mHealth innovations, is suggested and encouraged.[144]

Objective measures (e.g. HIV/STIs diagnosis, VL load) are desired in reporting of quantitative outcomes, over subjective self-reported data (e.g. condom use, self-reported adherence). This could potentially reduce some biases (misclassification/desirability bias/recall bias). Qualitative data are rich and complement the understanding of all the contextual and population needs, and capture the dynamics of sustainability and change. They need to be urgently integrated with quantitative data to provide a holistic picture of innovation.

The quality of digital data requires improvement. Across studies, a lack of integrated online impact metrics in evaluating the success of innovations was evident. With continuously evolving digital media, inventing new ways to evaluate acceptability and feasibility becomes necessary. For example, some studies tracked online metrics via Google analytics.[74 100-101 121-124] Synergy with industry powered metrics could be a new wave to measure success.

To scale up proven innovations, a multi-stakeholder engagement is necessary. For that, data and metrics that appeal to all sections of stakeholders are needed. In addition to improving the quality of randomized controlled trials and quasi-experimental impact studies, qualitative studies, cost effectiveness studies, usability studies, are needed.

Implications for policy and practice

In consonance with other systematic reviews, evidence at-scale was scarce.[138] This limits the projection of the long-term effectiveness of digital innovations. More evidence on scale-up, cost savings and cost-effectiveness from Sub Saharan Africa and Asia is needed. Future investments that incentivize both: the development and evaluation of combined innovations by government and industry alike, and focus on sustainability of digital innovations with public and private partnerships, are urgently needed.

For peer review only - http://bmjopen.bmj.com/site/about/guidelines.xhtml

CONCLUSION

To control HIV/STIs globally, we need novel and disruptive innovations that will uniquely impact health outcomes across the spectrum of access, engagement, treatment and retention so as to impact health service delivery. On one hand, mHealth (SMS/phone calls only) and internet-based m/eHealth were found acceptable, feasible and offered complementarity in improving prevention and care of HIV/STIs. On the other hand, when combined, they provided customized and contextualized solutions for hard-to-reach populations. Integrating these innovations across various levels of healthcare with clear evaluation, monitoring, and documentation of metrics will help enhance existing health service delivery models to impact health outcomes over time. Findings from this comprehensive review will be informative to all stakeholders – innovators, researchers, healthcare practitioners, policy makers and funders – worldwide seeking evidence on innovations across the spectrum of HIV/STI care.

ACKNOWLEDGEMENT

The authors would like to acknowledge Ms. Megan Smallwood for her assistance in editing the manuscript.

FOOTNOTES

Contributors:

NPP and JD: concept, design.

NPP: data critiquing, write-up, critique, and overall responsibility of the data.

JD: data synthesis, write-up, critiquing.

RV, BL and SD: data synthesis, write-up and critique.

JK, TP and KD: write-up and critique.

Final version approved by all authors

Competing Interests:

There are no conflicts of interest

Data Sharing:

No additional data are available. This is a systematic review/syntheses of existing studies, therefore all data are reported in the tables.

Funding:

Grand Challenges Canada Transition to Scale; Grant number 0710-05.
FRSQ salary award Chercheur Junior 1.

For peer review only - http://bmjopen.bmj.com/site/about/guidelines.xhtml

REFERENCES

1. The World Health Organization. Sexually transmitted infections (STIs): Fact sheet N°110: WHO Media Centre; 2015 [updated December 2015. Available from: http://www.who.int/mediacentre/factsheets/fs110/en/ accessed 27 January 2016.
2. The World Health Organization. Hepatitis B: Fact sheet N°204: WHO Media Centre; 2015 [updated July 2015. Available from: http://www.who.int/mediacentre/factsheets/fs204/en/ accessed 27 January 2016.
3. The World health Organization. Hepatitis C: Fact sheet N°164 2015 [updated July 2015. Available from: http://www.who.int/mediacentre/factsheets/fs164/en/ accessed 27 January 2016.
4. The Joint United Nations Programme on HIV/AIDS. Fact Sheet 2015 2015 [Available from: http://www.unaids.org/en/resources/campaigns/HowAIDSchangedeverything/factsheet accessed 17 Decembre 2016.
5. Newman L, Rowley J, Vander Hoorn S, et al. Global Estimates of the Prevalence and Incidence of Four Curable Sexually Transmitted Infections in 2012 Based on Systematic Review and Global Reporting. *PLoS One* 2015;10(12):e0143304. doi: 10.1371/journal.pone.0143304
PONE-D-15-31044 [pii] [published Online First: 2015/12/10]
6. The Joint United Nations Programme on HIV/AIDS. The Gap Report. Geneva: UNAIDS, 2014.
7. Kagee A, Remien RH, Berkman A, et al. Structural barriers to ART adherence in Southern Africa: Challenges and potential ways forward. *Glob Public Health* 2011;6(1):83-97. doi: 922541258 [pii]
10.1080/17441691003796387 [published Online First: 2010/05/29]
8. Pai NP. Multiplexed point-of-care assays for HIV and co-infections for resource constrained settings: a perspective. *Future Microbiol* 2015;10(9):1393-6. doi: 10.2217/fmb.15.77 [published Online First: 2015/09/09]
9. Pai NP, Vadnais C, Denkinger C, et al. Point-of-care testing for infectious diseases: diversity, complexity, and barriers in low- and middle-income countries. *PLoS Med* 2012;9(9):e1001306. doi: 10.1371/journal.pmed.1001306
PMEDICINE-D-12-01227 [pii] [published Online First: 2012/09/14]
10. Rosen S, Fox MP. Retention in HIV care between testing and treatment in sub-Saharan Africa: a systematic review. *PLoS Med* 2011;8(7):e1001056. doi: 10.1371/journal.pmed.1001056
PMEDICINE-D-11-00096 [pii] [published Online First: 2011/08/04]
11. Ryu S. Book Review: mHealth: New Horizons for Health through Mobile Technologies: Based on the Findings of the Second Global Survey on eHealth (Global Observatory for eHealth Series, Volume 3). *Healthcare Informatics research* 2012;18(3):231-33. doi: doi:10.4258/hir.2012.18.3.231
12. Free C, Phillips G, Watson L, et al. The effectiveness of mobile-health technologies to improve health care service delivery processes: a systematic review and meta-analysis. *PLoS Med* 2013;10(1):e1001363. doi: 10.1371/journal.pmed.1001363
PMEDICINE-D-12-00641 [pii] [published Online First: 2013/03/06]
13. The World Bank. Maximizing Mobile: Information and Communication Technologies for Development. . Washington DC: International Bank for Reconstruction and Development The World Bank 2012.
14. eMarketer. Smartphone user penetration as percentage of total global population from 2011 to 2018. : Statistica- The Statistics Portal; 2014 [Available from: http://www.statista.com/statistics/203734/global-smartphone-penetration-per-capita-since-2005/ accessed 19 February 2016.

For peer review only - http://bmjopen.bmj.com/site/about/guidelines.xhtml

15. eMarketer. Mobile phone internet user penetration worldwide from 2014 to 2019. : Statistica- The Statistics Potal; 2014 [Available from: http://www.statista.com/statistics/284202/mobile-phone-internet-user-penetration-worldwide/ accessed 19 February 2016.
16. Oulasvirta A, Rattenbury T, Ma L, et al. Habits make smartphone use more pervasive. *Personal and Ubiquitous Computing* 2012;16(1):105-14.
17. Topol EJ. The creative destruction of medicine: How the digital revolution will create better health care. . New York: Basic Books 2012.
18. Akter S, Ray P. mHealth - an Ultimate Platform to Serve the Unserved. *Yearb Med Inform* 2010:94-100. doi: me10010094 [pii] [published Online First: 2010/10/13]
19. Ali EE, Chew L, Yap KYL. Evolution and current status of mhealth research: A systematic review. *BMJ Innov* 2016;2(1):33-40. doi: 10.1136/bmjinnov-2015-000096
20. Lester RT, Ritvo P, Mills EJ, et al. Effects of a mobile phone short message service on antiretroviral treatment adherence in Kenya (WelTel Kenya1): a randomised trial. *Lancet* 2010;376(9755):1838-45. doi: S0140-6736(10)61997-6 [pii]
10.1016/S0140-6736(10)61997-6 [published Online First: 2010/11/13]
21. Ambia J, Mandala J. A systematic review of interventions to improve prevention of mother-to-child HIV transmission service delivery and promote retention. *Journal of the International AIDS Society* 2016;19(1):20309. doi: 10.7448/ias.19.1.20309 [published Online First: 2016/04/09]
22. Conserve DF, Jennings L, Aguiar C, et al. Systematic review of mobile health behavioural interventions to improve uptake of HIV testing for vulnerable and key populations. *J Telemed Telecare* 2017;23(2):347-59. doi: 10.1177/1357633x16639186 [published Online First: 2016/04/09]
23. L'Engle KL, Mangone ER, Parcesepe AM, et al. Mobile Phone Interventions for Adolescent Sexual and Reproductive Health: A Systematic Review. *Pediatrics* 2016;138(3) doi: 10.1542/peds.2016-0884 [published Online First: 2016/08/25]
24. Lima IC, Galvao MT, Alexandre Hde O, et al. Information and communication technologies for adherence to antiretroviral treatment in adults with HIV/AIDS. *Int J Med Inform* 2016;92:54-61. doi: 10.1016/j.ijmedinf.2016.04.013 [published Online First: 2016/06/19]
25. Dunne A, McIntosh J, Mallory D. Adolescents, Sexually Transmitted Infections, and Education Using Social Media: A Review of the Literature. *JNP-J Nurse Pract* 2014;10(6):409-16. doi: 10.1016/j.nurpra.2014.03.020
26. Jones K, Eathington P, Baldwin K, et al. The Impact of Health Education Transmitted Via Social Media or Text Messaging on Adolescent and Young Adult Risky Sexual Behavior: A Systematic Review of the Literature. *Sex Transm Dis* 2014;41(7):413-19. doi: 10.1097/olq.0000000000000146
27. Schnall R, Travers J, Rojas M, et al. eHealth Interventions for HIV Prevention in High-Risk Men Who Have Sex With Men: A Systematic Review. *J Med Internet Res* 2014;16(5):228-37. doi: 10.2196/jmir.3393
28. Moher D, Liberati A, Tetzlaff J, et al. Preferred reporting items for systematic reviews and meta-analyses: the PRISMA statement. *Ann Intern Med* 2009;151(4):264-9, W64. doi: 0000605-200908180-00135 [pii] [published Online First: 2009/07/23]
29. Chiavegatti T, Vijh R, Pant Pai N. Metrics beyond accuracy for HIV point-of-care tests: a systematic review of evidence. *Canadian Journal of Infectious Diseases & Medical Microbiology* 2014;25(Suppl A):90A.
30. Pai M, Filion K. Classification of study designs (Version 8) Montreal: McGill University; 2014 [Available from: http://www.teachepi.org/documents/courses/Classification%20Design.pdf.
31. Harris R, Bradburn M, Deeks J, et al. metan: fixed- and random-effects meta-analysis. *Stata Journal* 2008;8(1):3-28.
32. Pop-Eleches C, Thirumurthy H, Habyarimana JP, et al. Mobile phone technologies improve adherence to antiretroviral treatment in a resource-limited setting: a randomized controlled trial of text

For peer review only - http://bmjopen.bmj.com/site/about/guidelines.xhtml

message reminders. *AIDS* 2011;25(6):825-34. doi: 10.1097/QAD.0b013e32834380c1 [published Online First: 2011/01/22]

33. Dowshen N, Kuhns LM, Johnson A, et al. Improving adherence to antiretroviral therapy for youth living with HIV/AIDS: a pilot study using personalized, interactive, daily text message reminders. *J Med Internet Res* 2012;14(2):e51. doi: v14i2e51 [pii] 10.2196/jmir.2015 [published Online First: 2012/04/07]
34. Menon-Johansson AS, McNaught F, Mandalia S, et al. Texting decreases the time to treatment for genital Chlamydia trachomatis infection. *Sex Transm Infect* 2006;82(1):49-51. doi: 82/1/49 [pii] 10.1136/sti.2004.014381 [published Online First: 2006/02/08]
35. Finocchario-Kessler S, Gautney BJ, Khamadi S, et al. If you text them, they will come: using the HIV infant tracking system to improve early infant diagnosis quality and retention in Kenya. *AIDS* 2014;28 Suppl 3:S313-21. doi: 10.1097/QAD.0000000000000332 00002030-201407001-00011 [pii] [published Online First: 2014/07/06]
36. Lim EJ, Haar J, Morgan J. Can text messaging results reduce time to treatment of Chlamydia trachomatis? *Sex Transm Infect* 2008;84(7):563-4. doi: sti.2008.032441 [pii] 10.1136/sti.2008.032441 [published Online First: 2008/08/30]
37. Bailey S, Scalley B, Gilles M. Text 2 treat - using SMS to recall clients for treatment. *Int J STD AIDS* 2014;25(14):1038-40. doi: 0956462414530108 [pii] 10.1177/0956462414530108 [published Online First: 2014/04/04]
38. Smillie K, Van Borek N, Abaki J, et al. A qualitative study investigating the use of a mobile phone short message service designed to improve HIV adherence and retention in care in Canada (WelTel BC1). *J Assoc Nurses AIDS Care* 2014;25(6):614-25. doi: S1055-3290(14)00040-5 [pii] 10.1016/j.jana.2014.01.008 [published Online First: 2014/04/29]
39. Reback CJ, Fletcher JB, Shoptaw S, et al. Exposure to Theory-Driven Text Messages is Associated with HIV Risk Reduction Among Methamphetamine-Using Men Who have Sex with Men. *AIDS Behav* 2015;19 Suppl 2:130-41. doi: 10.1007/s10461-014-0985-7 [published Online First: 2015/01/08]
40. Evans C, Turner K, Blake H, et al. Text messaging to encourage uptake of HIV testing amongst African communities: Findings from a theory based feasibility study. *HIV Medicine* 2015;16(Suppl 2):54.
41. Dowshen N, Kuhns LM, Gray C, et al. Feasibility of interactive text message response (ITR) as a novel, real-time measure of adherence to antiretroviral therapy for HIV+ youth. *AIDS Behav* 2013;17(6):2237-43. doi: 10.1007/s10461-013-0464-6 [published Online First: 2013/04/03]
42. Cornelius JB, Dmochowski J, Boyer C, et al. Text-messaging-enhanced HIV intervention for African American adolescents: a feasibility study. *J Assoc Nurses AIDS Care* 2013;24(3):256-67. doi: S1055-3290(12)00140-9 [pii] 10.1016/j.jana.2012.06.005 [published Online First: 2012/11/06]
43. Ammassari A, Trotta MP, Mussini C, et al. Mobile phone intervention based on short message system (SMS) improve adherence behavior in HIV-infected non-adherent to antiretroviral therapy. The HI-Vision Study. *Infection* 2010;38(Suppl 52):52.
44. Ammassari A, Trotta MP, Shalev N, et al. Timed short messaging service improves adherence and virological outcomes in HIV-1-infected patients with suboptimal adherence to antiretroviral therapy. *J Acquir Immune Defic Syndr* 2011;58(4):e113-5. doi: 10.1097/QAI.0b013e3182359d2a 00126334-201112010-00015 [pii] [published Online First: 2011/10/29]
45. Segaren N, Lewis T, Desinor O, et al. The distribution and use of cell phones to mothers of HIV-positive infants identified by the Haiti National Early Infant Diagnosis of HIV program (EID): a model for increasing adherence? XIX International AIDS Conference Washington DC, 2012.
46. Sheppard Z, Walsh J, Weston R. The TRxCare™ adherence support system: a pilot study of its acceptability to patients on virologically successful HAART. 18th Annual Conference of British HIV Association Birmingham, 2012.

For peer review only - http://bmjopen.bmj.com/site/about/guidelines.xhtml

47. da Costa TM, Barbosa BJ, Gomes e Costa DA, et al. Results of a randomized controlled trial to assess the effects of a mobile SMS-based intervention on treatment adherence in HIV/AIDS-infected Brazilian women and impressions and satisfaction with respect to incoming messages. *Int J Med Inform* 2012;81(4):257-69. doi: S1386-5056(11)00204-8 [pii]
10.1016/j.ijmedinf.2011.10.002 [published Online First: 2012/02/03]
48. Sabin LL, Bachman DeSilva M, Gill CJ, et al. Improving Adherence to Antiretroviral Therapy With Triggered Real-time Text Message Reminders: The China Adherence Through Technology Study. *J Acquir Immune Defic Syndr* 2015;69(5):551-9. doi: 10.1097/QAI.0000000000000651 [published Online First: 2015/04/19]
49. Brook G, Farmer T, Murphy S, et al. Using SMS text reminders to reduce 'did not attend' (DNA) rates in sexual health and HIV appointment clinics. *Sex Transm Infect* 2013;89(Suppl 1):A1-A428.
50. Kliner M, Knight A, Mamvura C, et al. Using no-cost mobile phone reminders to improve attendance for HIV test results: a pilot study in rural Swaziland. *Infect Dis Poverty* 2013;2(1):12. doi: 2049-9957-2-12 [pii]
10.1186/2049-9957-2-12 [published Online First: 2013/07/16]
51. Farmer T, Brook G, McSorley J, et al. Using short message service text reminders to reduce 'did not attend' rates in sexual health and HIV appointment clinics. *Int J STD AIDS* 2014;25(4):289-93. doi: 0956462413502325 [pii]
10.1177/0956462413502325 [published Online First: 2013/09/04]
52. Brook G, Burton J, McSorley J, et al. The effectiveness of SMS texts for reminding patietns at high risk of STIs and HIV to return for testing. *Sex Transm Infect* 2013;89(Suppl 1):A1-A428.
53. Guy R, Wand H, Knight V, et al. SMS reminders improve re-screening in women and heterosexual men with chlamydia infection at Sydney Sexual Health Centre: a before-and-after study. *Sex Transm Infect* 2013;89(1):11-5. doi: sextrans-2011-050370 [pii]
10.1136/sextrans-2011-050370 [published Online First: 2012/04/21]
54. Burton J, Brook G, McSorley J, et al. The utility of short message service (SMS) texts to remind patients at higher risk of STIs and HIV to reattend for testing: a controlled before and after study. *Sex Transm Infect* 2014;90(1):11-3. doi: sextrans-2013-051228 [pii]
10.1136/sextrans-2013-051228 [published Online First: 2013/09/26]
55. Desai M, Burns F, Mercey D, et al. Active recall of men who have sex with men (MSM) for an HIV/STI testing: a feasible and effective strategy? *HIV Medicine* 2014;15(Suppl 3):17-159.
56. Kharbanda EO, Stockwell MS, Fox HW, et al. Text message reminders to promote human papillomavirus vaccination. *Vaccine* 2011;29(14):2537-41. doi: S0264-410X(11)00125-3 [pii]
10.1016/j.vaccine.2011.01.065 [published Online First: 2011/02/09]
57. Vilella A, Bayas JM, Diaz MT, et al. The role of mobile phones in improving vaccination rates in travelers. *Prev Med* 2004;38(4):503-9. doi: 10.1016/j.ypmed.2003.12.005
S0091743503003475 [pii] [published Online First: 2004/03/17]
58. Downing SG, Cashman C, McNamee H, et al. Increasing chlamydia test of re-infection rates using SMS reminders and incentives. *Sex Transm Infect* 2013;89(1):16-9. doi: sextrans-2011-050454 [pii]
10.1136/sextrans-2011-050454 [published Online First: 2012/06/26]
59. Odeny TA, Bailey RC, Bukusi EA, et al. Text messaging to improve attendance at post-operative clinic visits after adult male circumcision for HIV prevention: a randomized controlled trial. *PLoS One* 2012;7(9):e43832. doi: 10.1371/journal.pone.0043832
PONE-D-12-11992 [pii] [published Online First: 2012/09/08]
60. de Tolly K, Skinner D, Nembaware V, et al. Investigation into the use of short message services to expand uptake of human immunodeficiency virus testing, and whether content and dosage have impact. *Telemed J E Health* 2012;18(1):18-23. doi: 10.1089/tmj.2011.0058 [published Online First: 2011/12/14]

For peer review only - http://bmjopen.bmj.com/site/about/guidelines.xhtml

61. Bigna JJ, Noubiap JJ, Kouanfack C, et al. Effect of mobile phone reminders on follow-up medical care of children exposed to or infected with HIV in Cameroon (MORE CARE): a multicentre, single-blind, factorial, randomised controlled trial. *Lancet Infect Dis* 2014;14(7):600-8. doi: S1473-3099(14)70741-8 [pii]
10.1016/S1473-3099(14)70741-8 [published Online First: 2014/06/17]
62. Rand CM, Brill H, Albertin C, et al. Effectiveness of centralized text message reminders on human papillomavirus immunization coverage for publicly insured adolescents. *J Adolesc Health* 2015;56(5 Suppl):S17-20. doi: S1054-139X(14)00707-1 [pii]
10.1016/j.jadohealth.2014.10.273 [published Online First: 2015/04/13]
63. Matheson EC, Derouin A, Gagliano M, et al. Increasing HPV vaccination series completion rates via text message reminders. *J Pediatr Health Care* 2014;28(4):e35-9. doi: S0891-5245(13)00293-9 [pii]
10.1016/j.pedhc.2013.09.001 [published Online First: 2013/11/10]
64. Schwartz SR, Clouse K, Yende N, et al. Acceptability and Feasibility of a Mobile Phone-Based Case Management Intervention to Retain Mothers and Infants from an Option B+ Program in Postpartum HIV Care. *Matern Child Health J* 2015;19(9):2029-37. doi: 10.1007/s10995-015-1715-0 [published Online First: 2015/02/07]
65. Belzer ME, Naar-King S, Olson J, et al. The use of cell phone support for non-adherent HIV-infected youth and young adults: an initial randomized and controlled intervention trial. *AIDS Behav* 2014;18(4):686-96. doi: 10.1007/s10461-013-0661-3 [published Online First: 2013/11/26]
66. Maduka O, Tobin-West CI. Adherence counseling and reminder text messages improve uptake of antiretroviral therapy in a tertiary hospital in Nigeria. *Niger J Clin Pract* 2013;16(3):302-8. doi: NigerJClinPract_2013_16_3_302_113451 [pii]
10.4103/1119-3077.113451 [published Online First: 2013/06/19]
67. Kebaya L, Nduati R, Bashir A. Efficacy of mobile phone use on adherence to nevirapine prophylaxis and retention in care among the HIV-exposed infants in PMTCT: a randomized controlled trial. *Arch Dis Child* 2014;99(Suppl 2):A1-A620.
68. Hardy H, Kumar V, Doros G, et al. Randomized controlled trial of a personalized cellular phone reminder system to enhance adherence to antiretroviral therapy. *AIDS Patient Care STDS* 2011;25(3):153-61. doi: 10.1089/apc.2010.0006 [published Online First: 2011/02/18]
69. DiClemente RJ, Wingood GM, Sales JM, et al. Efficacy of a telephone-delivered sexually transmitted infection/human immunodeficiency virus prevention maintenance intervention for adolescents: a randomized clinical trial. *JAMA Pediatr* 2014;168(10):938-46. doi: 1899236 [pii]
10.1001/jamapediatrics.2014.1436 [published Online First: 2014/08/27]
70. Cornelius JB, Cato M, Lawrence JS, et al. Development and pretesting multimedia HIV-prevention text messages for mobile cell phone delivery. *J Assoc Nurses AIDS Care* 2011;22(5):407-13. doi: S1055-3290(10)00224-4 [pii]
10.1016/j.jana.2010.11.007 [published Online First: 2011/01/25]
71. Dean AL, Makin JD, Kydd AS, et al. A pilot study using interactive SMS support groups to prevent mother-to-child HIV transmission in South Africa. *J Telemed Telecare* 2012;18(7):399-403. doi: jtt.2012.120118 [pii]
10.1258/jtt.2012.120118 [published Online First: 2012/10/05]
72. Juzang I, Fortune T, Black S, et al. A pilot programme using mobile phones for HIV prevention. *J Telemed Telecare* 2011;17(3):150-3. doi: jtt.2010.091107 [pii]
10.1258/jtt.2010.091107 [published Online First: 2011/01/29]
73. Kalichman SC, Kalichman MO, Cherry C, et al. Brief behavioral self-regulation counseling for HIV treatment adherence delivered by cell phone: an initial test of concept trial. *AIDS Patient Care STDS* 2011;25(5):303-10. doi: 10.1089/apc.2010.0367 [published Online First: 2011/04/05]

For peer review only - http://bmjopen.bmj.com/site/about/guidelines.xhtml

74. Mbuagbaw L, Thabane L, Ongolo-Zogo P, et al. The Cameroon Mobile Phone SMS (CAMPS) trial: a randomized trial of text messaging versus usual care for adherence to antiretroviral therapy. *PLoS One* 2012;7(12):e46909. doi: 10.1371/journal.pone.0046909
PONE-D-12-24092 [pii] [published Online First: 2012/12/14]
75. Moore DJ, Poquette A, Casaletto KB, et al. Individualized texting for adherence building (iTAB): improving antiretroviral dose timing among HIV-infected persons with co-occurring bipolar disorder. *AIDS Behav* 2015;19(3):459-71. doi: 10.1007/s10461-014-0971-0 [published Online First: 2014/12/17]
76. Norton BL, Person AK, Castillo C, et al. Barriers to using text message appointment reminders in an HIV clinic. *Telemed J E Health* 2014;20(1):86-9. doi: 10.1089/tmj.2012.0275 [published Online First: 2013/10/29]
77. Odeny TA, Bailey RC, Bukusi EA, et al. Effect of text messaging to deter early resumption of sexual activity after male circumcision for HIV prevention: a randomized controlled trial. *J Acquir Immune Defic Syndr* 2014;65(2):e50-7. doi: 10.1097/QAI.0b013e3182a0a050 [published Online First: 2013/07/13]
78. Bauermeister J, Carballo-Dieguez A, Giguere R, et al. Interactive voice response system (IVRS): data quality considerations and lessons learned during a microbicide placebo adherence trial with young men who have sex with men. *Journal of Adolescent Health* 2014;54(2):S57-S58.
79. Belzer ME, Kolmodin MacDonell K, Clark LF, et al. Acceptability and Feasibility of a Cell Phone Support Intervention for Youth Living with HIV with Nonadherence to Antiretroviral Therapy. *AIDS Patient Care STDS* 2015;29(6):338-45. doi: 10.1089/apc.2014.0282 [published Online First: 2015/05/01]
80. Shet A, De Costa A, Kumarasamy N, et al. Effect of mobile telephone reminders on treatment outcome in HIV: evidence from a randomised controlled trial in India. *BMJ* 2014;349:g5978. [published Online First: 2014/01/01]
81. Reid MJ, Dhar SI, Cary M, et al. Opinions and attitudes of participants in a randomized controlled trial examining the efficacy of SMS reminders to enhance antiretroviral adherence: a cross-sectional survey. *J Acquir Immune Defic Syndr* 2014;65(2):e86-8. doi: 10.1097/QAI.0b013e3182a9c72b
00126334-201402010-00027 [pii] [published Online First: 2014/01/21]
82. Roth AM, Hensel DJ, Fortenberry JD, et al. Feasibility and acceptability of cell phone diaries to measure HIV risk behavior among female sex workers. *AIDS Behav* 2014;18(12):2314-24. doi: 10.1007/s10461-014-0718-y [published Online First: 2014/03/20]
83. Barnabas RV, van Rooyen H, Tumwesigye E, et al. Uptake of antiretroviral therapy and male circumcision after community-based HIV testing and strategies for linkage to care versus standard clinic referral: a multisite, open-label, randomised controlled trial in South Africa and Uganda. *The lancet HIV* 2016;3(5):e212-20. doi: 10.1016/s2352-3018(16)00020-5 [published Online First: 2016/04/30]
84. Bassett IV, Coleman SM, Giddy J, et al. Sizanani: A Randomized Trial of Health System Navigators to Improve Linkage to HIV and TB Care in South Africa. *J Acquir Immune Defic Syndr* 2016;73(2):154-60. doi: 10.1097/qai.0000000000001025 [published Online First: 2016/09/16]
85. Cantudo-Cuenca MR, Manzano-Garcia M, Jimenez-Galan R, et al. Effect of mobile phone short messages on antiretroviral treatment adherence among HIV infected patients. *International Journal of Clinical Pharmacy* 2016;38 (6):517. doi: http://dx.doi.org/10.1007/s11096-015-0240-y
86. Haberer JE, Musiimenta A, Atukunda EC, et al. Short message service (SMS) reminders and real-time adherence monitoring improve antiretroviral therapy adherence in rural Uganda. *AIDS* 2016;30(8):1295-300. doi: 10.1097/qad.0000000000001021 [published Online First: 2016/01/14]
87. Jeffries C, Ross P, Matoff-Stepp S, et al. Ucare4life: Mobile texting to improve HIV care continuum outcomes for minority youth. *Topics in Antiviral Medicine* 2016;24 (E-1):427.

For peer review only - http://bmjopen.bmj.com/site/about/guidelines.xhtml

88. Joseph Davey D, Nhavoto JA, Augusto O, et al. SMSaude: Evaluating Mobile Phone Text Reminders to Improve Retention in HIV Care for Patients on Antiretroviral Therapy in Mozambique. *J Acquir Immune Defic Syndr* 2016;73(2):e23-30. doi: 10.1097/qai.0000000000001115 [published Online First: 2016/09/16]
89. Kampman C, Koedijk F, Driessen-Hulshof H, et al. Retesting young STI clinic visitors with urogenital Chlamydia trachomatis infection in the Netherlands; response to a text message reminder and reinfection rates: a prospective study with historical controls. *Sex Transm Infect* 2016;92(2):124-9. doi: 10.1136/sextrans-2015-052115 [published Online First: 2015/09/26]
90. Kassaye SG, Ong'ech J, Sirengo M, et al. Cluster-Randomized Controlled Study of SMS Text Messages for Prevention of Mother-to-Child Transmission of HIV in Rural Kenya. *AIDS research and treatment* 2016;2016:1289328. doi: 10.1155/2016/1289328 [published Online First: 2017/01/06]
91. McIver R, Dyda A, McNulty AM, et al. Text message reminders do not improve hepatitis B vaccination rates in an Australian sexual health setting. *Journal of the American Medical Informatics Association : JAMIA* 2016;23(e1):e88-92. doi: 10.1093/jamia/ocv145 [published Online First: 2015/10/27]
92. Njuguna N, Ngure K, Mugo N, et al. The Effect of Human Immunodeficiency Virus Prevention and Reproductive Health Text Messages on Human Immunodeficiency Virus Testing Among Young Women in Rural Kenya: A Pilot Study. *Sex Transm Dis* 2016;43(6):353-9. doi: 10.1097/olq.0000000000000450 [published Online First: 2016/05/21]
93. Nsagha DS, Lange I, Fon PN, et al. A Randomized Controlled Trial on the Usefulness of Mobile Text Phone Messages to Improve the Quality of Care of HIV and AIDS Patients in Cameroon. *Open AIDS J* 2016;10:93-103. doi: 10.2174/1874613601610010093 [published Online First: 2016/09/02]
94. Nyatsanza F, McSorley J, Murphy S, et al. 'It's all in the message': the utility of personalised short message service (SMS) texts to remind patients at higher risk of STIs and HIV to reattend for testing-a repeat before and after study. *Sex Transm Infect* 2016;92(5):393-5. doi: 10.1136/sextrans-2015-052216 [published Online First: 2015/12/17]
95. Rana AI, van den Berg JJ, Lamy E, et al. Using a Mobile Health Intervention to Support HIV Treatment Adherence and Retention Among Patients at Risk for Disengaging with Care. *AIDS Patient Care STDS* 2016;30(4):178-84. doi: 10.1089/apc.2016.0025 [published Online First: 2016/03/31]
96. Rand CM, Vincelli P, Goldstein NP, et al. Effects of Phone and Text Message Reminders on Completion of the Human Papillomavirus Vaccine Series. *J Adolesc Health* 2017;60(1):113-19. doi: 10.1016/j.jadohealth.2016.09.011 [published Online First: 2016/11/12]
97. Coleman J, Bohlin KC, Thorson A, et al. Effectiveness of an SMS-based maternal mHealth intervention to improve clinical outcomes of HIV-positive pregnant women. *AIDS care* 2017:1-8. doi: 10.1080/09540121.2017.1280126 [published Online First: 2017/01/22]
98. Garofalo R, Kuhns LM, Hotton A, et al. A Randomized Controlled Trial of Personalized Text Message Reminders to Promote Medication Adherence Among HIV-Positive Adolescents and Young Adults. *AIDS Behav* 2016;20(5):1049-59. doi: 10.1007/s10461-015-1192-x [published Online First: 2015/09/13]
99. Georgette N, Siedner MJ, Zanoni B, et al. The Acceptability and Perceived Usefulness of a Weekly Clinical SMS Program to Promote HIV Antiretroviral Medication Adherence in KwaZulu-Natal, South Africa. *AIDS Behav* 2016;20(11):2629-38. doi: 10.1007/s10461-016-1287-z [published Online First: 2016/10/21]
100. Dowshen N, Lee S, Matty Lehman B, et al. IknowUshould2: Feasibility of a Youth-Driven Social Media Campaign to Promote STI and HIV Testing Among Adolescents in Philadelphia. *AIDS Behav* 2015;19 Suppl 2:106-11. doi: 10.1007/s10461-014-0991-9 [published Online First: 2015/01/08]

For peer review only - http://bmjopen.bmj.com/site/about/guidelines.xhtml

101. Jones M, Aderogba K, Drunis L. Providing rapid HIV testing in their homes for men who have sex with men, recruited via social media. *HIV Medicine* 2015;16(Suppl 2):50-51.
102. Kurth AE, Spielberg F, Cleland CM, et al. Computerized counseling reduces HIV-1 viral load and sexual transmission risk: findings from a randomized controlled trial. *J Acquir Immune Defic Syndr* 2014;65(5):611-20. doi: 10.1097/QAI.0000000000000100 [published Online First: 2014/01/05]
103. Lelutiu-Weinberger C, Pachankis JE, Gamarel KE, et al. Feasibility, Acceptability, and Preliminary Efficacy of a Live-Chat Social Media Intervention to Reduce HIV Risk Among Young Men Who Have Sex With Men. *AIDS Behav* 2015;19(7):1214-27. doi: 10.1007/s10461-014-0911-z [published Online First: 2014/09/27]
104. Perera AI, Thomas MG, Moore JO, et al. Effect of a smartphone application incorporating personalized health-related imagery on adherence to antiretroviral therapy: a randomized clinical trial. *AIDS Patient Care STDS* 2014;28(11):579-86. doi: 10.1089/apc.2014.0156 [published Online First: 2014/10/08]
105. Jones K, Baldwin KA, Lewis PR. The potential influence of a social media intervention on risky sexual behavior and Chlamydia incidence. *J Community Health Nurs* 2012;29(2):106-20. doi: 10.1080/07370016.2012.670579 [published Online First: 2012/04/28]
106. Jones R, Hoover DR, Lacroix LJ. A randomized controlled trial of soap opera videos streamed to smartphones to reduce risk of sexually transmitted human immunodeficiency virus (HIV) in young urban African American women. *Nurs Outlook* 2013;61(4):205-15 e3. doi: S0029-6554(13)00061-4 [pii]
10.1016/j.outlook.2013.03.006 [published Online First: 2013/06/08]
107. Naar-King S, Outlaw AY, Sarr M, et al. Motivational Enhancement System for Adherence (MESA): pilot randomized trial of a brief computer-delivered prevention intervention for youth initiating antiretroviral treatment. *J Pediatr Psychol* 2013;38(6):638-48. doi: jss132 [pii]
10.1093/jpepsy/jss132 [published Online First: 2013/01/30]
108. Elliot E, Rossi M, McCormack S, et al. Identifying undiagnosed HIV in men who have sex with men (MSM) by offering HIV home sampling via online gay social media: a service evaluation. *Sex Transm Infect* 2016;92(6):470-3. doi: 10.1136/sextrans-2015-052090 [published Online First: 2016/05/26]
109. Garett R, Menacho L, Young SD. Ethical Issues in Using Social Media to Deliver an HIV Prevention Intervention: Results from the HOPE Peru Study. *Prevention science : the official journal of the Society for Prevention Research* 2017;18(2):225-32. doi: 10.1007/s11121-016-0739-z [published Online First: 2016/12/10]
110. Himelhoch S, Kreyenbuhl J, Palmer-Bacon J, et al. Pilot feasibility study of Heart2HAART: a smartphone application to assist with adherence among substance users living with HIV. *AIDS care* 2017:1-7. doi: 10.1080/09540121.2016.1259454 [published Online First: 2017/01/04]
111. Huang E, Marlin RW, Young SD, et al. Using Grindr, a Smartphone Social-Networking Application, to Increase HIV Self-Testing Among Black and Latino Men Who Have Sex With Men in Los Angeles, 2014. *AIDS education and prevention : official publication of the International Society for AIDS Education* 2016;28(4):341-50. doi: 10.1521/aeap.2016.28.4.341 [published Online First: 2016/07/20]
112. Jeon JH. Evaluation of a smartphone application for self-care performance of patients with chronic hepatitis B: A randomized controlled trial. *Applied nursing research : ANR* 2016;32:182-89. doi: 10.1016/j.apnr.2016.07.011 [published Online First: 2016/12/15]
113. Polilli E, Sozio F, Di Stefano P, et al. Web-Based HIV Testing in Abruzzo, Italy: Analysis of 15-Month Activity Results. *AIDS Patient Care STDS* 2016;30(10):471-75. doi: 10.1089/apc.2016.0082
114. Przybyla SM, Eliseo-Arras RK, Krawiec G, et al. Feasibility and Acceptability of a Smartphone App for Daily Reports of Substance Use and Antiretroviral Therapy Adherence among HIV-Infected

For peer review only - http://bmjopen.bmj.com/site/about/guidelines.xhtml

Adults. *AIDS research and treatment* 2016;2016:9510172. doi: 10.1155/2016/9510172 [published Online First: 2016/09/10]

115. Rhodes SD, McCoy TP, Tanner AE, et al. Using Social Media to Increase HIV Testing Among Gay and Bisexual Men, Other Men Who Have Sex With Men, and Transgender Persons: Outcomes From a Randomized Community Trial. *Clinical infectious diseases : an official publication of the Infectious Diseases Society of America* 2016;62(11):1450-3. doi: 10.1093/cid/ciw127 [published Online First: 2016/03/17]
116. Rosengren AL, Huang E, Daniels J, et al. Feasibility of using GrindrTM to distribute HIV self-test kits to men who have sex with men in Los Angeles, California. *Sexual health* 2016 doi: 10.1071/sh15236 [published Online First: 2016/05/23]
117. Solorio R, Norton-Shelpuk P, Forehand M, et al. Tu Amigo Pepe: Evaluation of a Multi-media Marketing Campaign that Targets Young Latino Immigrant MSM with HIV Testing Messages. *AIDS Behav* 2016;20(9):1973-88. doi: 10.1007/s10461-015-1277-6 [published Online First: 2016/02/07]
118. Talal AH, Andrews P, McLeod A, et al. Telemedicine-based Hepatitis C Virus (HCV) management for individuals on opioid agonist treatment (OAT). *Hepatology* 2016;63 (1 Supplement 1):475A.
119. Henwood R, Patten G, Barnett W, et al. Acceptability and use of a virtual support group for HIV-positive youth in Khayelitsha, Cape Town using the MXit social networking platform. *AIDS care* 2016;28(7):898-903. doi: 10.1080/09540121.2016.1173638 [published Online First: 2016/04/22]
120. Brayboy LM, Sepolen A, Mezoian T, et al. Girl Talk: A Smartphone Application to Teach Sexual Health Education to Adolescent Girls. *J Pediatr Adolesc Gynecol* 2017;30(1):23-28. doi: 10.1016/j.jpag.2016.06.011 [published Online First: 2016/07/10]
121. Horvath KJ, Oakes JM, Rosser BR, et al. Feasibility, acceptability and preliminary efficacy of an online peer-to-peer social support ART adherence intervention. *AIDS Behav* 2013;17(6):2031-44. doi: 10.1007/s10461-013-0469-1 [published Online First: 2013/04/05]
122. Gotz HM, van Rooijen MS, Vriens P, et al. Initial evaluation of use of an online partner notification tool for STI, called 'suggest a test': a cross sectional pilot study. *Sex Transm Infect* 2014;90(3):195-200. doi: sextrans-2013-051254 [pii]
10.1136/sextrans-2013-051254 [published Online First: 2014/01/07]
123. Friedman AL, Brookmeyer KA, Kachur RE, et al. An assessment of the GYT: Get Yourself Tested campaign: an integrated approach to sexually transmitted disease prevention communication. *Sex Transm Dis* 2014;41(3):151-7. doi: 10.1097/OLQ.0000000000000100
00007435-201403000-00001 [pii] [published Online First: 2014/02/14]
124. Hightow-Weidman LB, Muessig KE, Pike EC, et al. HealthMpowerment.org: Building Community Through a Mobile-Optimized, Online Health Promotion Intervention. *Health Educ Behav* 2015;42(4):493-9. doi: 1090198114562043 [pii]
10.1177/1090198114562043 [published Online First: 2015/01/16]
125. Dokkum NF, Koekenbier RH, van den Broek IV, et al. Keeping participants on board: increasing uptake by automated respondent reminders in an Internet-based chlamydia screening in the Netherlands. *BMC Public Health* 2012;12:176. doi: 1471-2458-12-176 [pii]
10.1186/1471-2458-12-176 [published Online First: 2012/03/13]
126. Hightow-Weidman L, Beagle S, Pike E, et al. "No one's at home and they won't pick up the phone": using the Internet and text messaging to enhance partner services in North Carolina. *Sex Transm Dis* 2014;41(2):143-8. doi: 10.1097/OLQ.0000000000000087
00007435-201402000-00014 [pii] [published Online First: 2014/01/15]
127. Aronson ID, Cleland CM, Perlman DC, et al. MOBILE SCREENING TO IDENTIFY AND FOLLOW-UP WITH HIGH RISK, HIV NEGATIVE YOUTH. *Journal of mobile technology in medicine* 2016;5(1):9-18. doi: 10.7309/jmtm.5.1.3 [published Online First: 2016/04/26]
128. Hirsch-Moverman Y, Daftary A, Yuengling KA, et al. Using mHealth for HIV/TB Treatment Support in Lesotho: Enhancing Patient-Provider Communication in the START Study. *J Acquir*

For peer review only - http://bmjopen.bmj.com/site/about/guidelines.xhtml

Immune Defic Syndr 2017;74 Suppl 1:S37-S43. doi: 10.1097/qai.0000000000001202 [published Online First: 2016/12/09]

129. John ME, Samson-Akpan PE, Etowa JB, et al. Enhancing self-care, adjustment and engagement through mobile phones in youth with HIV. *International nursing review* 2016;63(4):555-61. doi: 10.1111/inr.12313 [published Online First: 2016/09/23]
130. Guy R, Hocking J, Wand H, et al. How effective are short message service reminders at increasing clinic attendance? A meta-analysis and systematic review. *Health Serv Res* 2012;47(2):614-32. doi: 10.1111/j.1475-6773.2011.01342.x [published Online First: 2011/11/19]
131. Free C, Phillips G, Galli L, et al. The effectiveness of mobile-health technology-based health behaviour change or disease management interventions for health care consumers: a systematic review. *PLoS Med* 2013;10(1):e1001362. doi: 10.1371/journal.pmed.1001362
PMEDICINE-D-12-00520 [pii] [published Online First: 2013/01/26]
132. Ershad Sarabi R, Sadoughi F, Jamshidi Orak R, et al. The Effectiveness of Mobile Phone Text Messaging in Improving Medication Adherence for Patients with Chronic Diseases: A Systematic Review. *Iranian Red Crescent medical journal* 2016;18(5):e25183. doi: 10.5812/ircmj.25183 [published Online First: 2016/07/21]
133. Finitsis DJ, Pellowski JA, Huedo-Medina TB, et al. Visual analogue scale (VAS) measurement of antiretroviral adherence in people living with HIV (PLWH): a meta-analysis. *Journal of behavioral medicine* 2016;39(6):1043-55. doi: 10.1007/s10865-016-9770-6 [published Online First: 2016/08/03]
134. Kanters S, Park JJ, Chan K, et al. Interventions to improve adherence to antiretroviral therapy: a systematic review and network meta-analysis. *The lancet HIV* 2017;4(1):e31-e40. doi: 10.1016/s2352-3018(16)30206-5 [published Online First: 2016/11/20]
135. Mayer JE, Fontelo P. Meta-analysis on the effect of text message reminders for HIV-related compliance. *AIDS care* 2016:1-9. doi: 10.1080/09540121.2016.1214674 [published Online First: 2016/08/02]
136. Thakkar J, Kurup R, Laba TL, et al. Mobile telephone text messaging for medication adherence in chronic disease a meta-analysis. *JAMA Intern Med* 2016;176(3):340-49. doi: 10.1001/jamainternmed.2015.7667
137. Mbuagbaw L, Mursleen S, Lytvyn L, et al. Mobile phone text messaging interventions for HIV and other chronic diseases: an overview of systematic reviews and framework for evidence transfer. *BMC Health Serv Res* 2015;15:33. doi: 10.1186/s12913-014-0654-6
s12913-014-0654-6 [pii] [published Online First: 2015/01/23]
138. Muessig KE, Nekkanti M, Bauermeister J, et al. A Systematic Review of Recent Smartphone, Internet and Web 2.0 Interventions to Address the HIV Continuum of Care. *Curr Hiv/Aids Rep* 2015;12(1):173-90. doi: 10.1007/s11904-014-0239-3
139. Gabarron E, Wynn R. Use of social media for sexual health promotion: a scoping review. *Global health action* 2016;9:32193. doi: 10.3402/gha.v9.32193 [published Online First: 2016/09/22]
140. Chavez NR, Shearer LS, Rosenthal SL. Use of digital media technology for primary prevention of STIs/HIV in youth. *J Pediatr Adolesc Gynecol* 2014;27(5):244-57. doi: 10.1016/j.jpag.2013.07.008 [published Online First: 2013/12/18]
141. Barello S, Triberti S, Graffigna G, et al. eHealth for Patient Engagement: A Systematic Review. *Front Psychol* 2015;6:2013. doi: 10.3389/fpsyg.2015.02013 [published Online First: 2016/01/19]
142. Finitsis DJ, Pellowski JA, Johnson BT. Text Message Intervention Designs to Promote Adherence to Antiretroviral Therapy (ART): A Meta-Analysis of Randomized Controlled Trials. *PLoS ONE* 2014;9(2) doi: e88166
10.1371/journal.pone.0088166
143. Fletcher WR, Fletcher WS. Chapter 8: Treatment. Clinical Epidemiology: The Essentials. 4th ed. Baltimore: Lippincott Williams & Wilkins 2005:141.

For peer review only - http://bmjopen.bmj.com/site/about/guidelines.xhtml

144. Agarwal S, LeFevre AE, Lee J, et al. Guidelines for reporting of health interventions using mobile phones: mobile health (mHealth) evidence reporting and assessment (mERA) checklist. *BMJ* 2016;352:i1174. [published Online First: 2016/03/19]

For peer review only

For peer review only - http://bmjopen.bmj.com/site/about/guidelines.xhtml

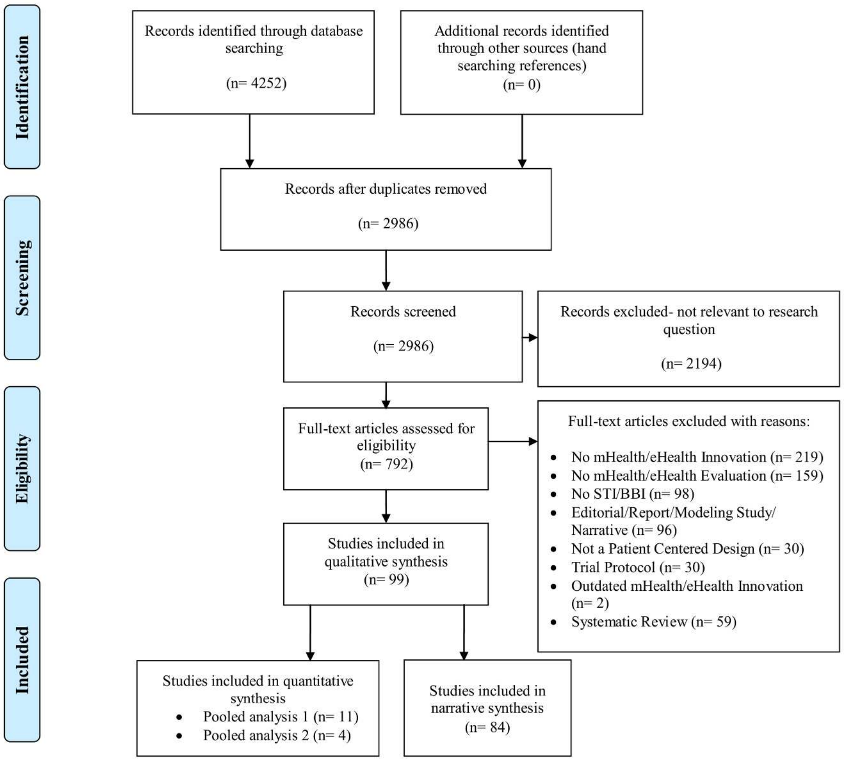

Figure 1: PRISMA flow diagram

215x279mm (300 x 300 DPI)

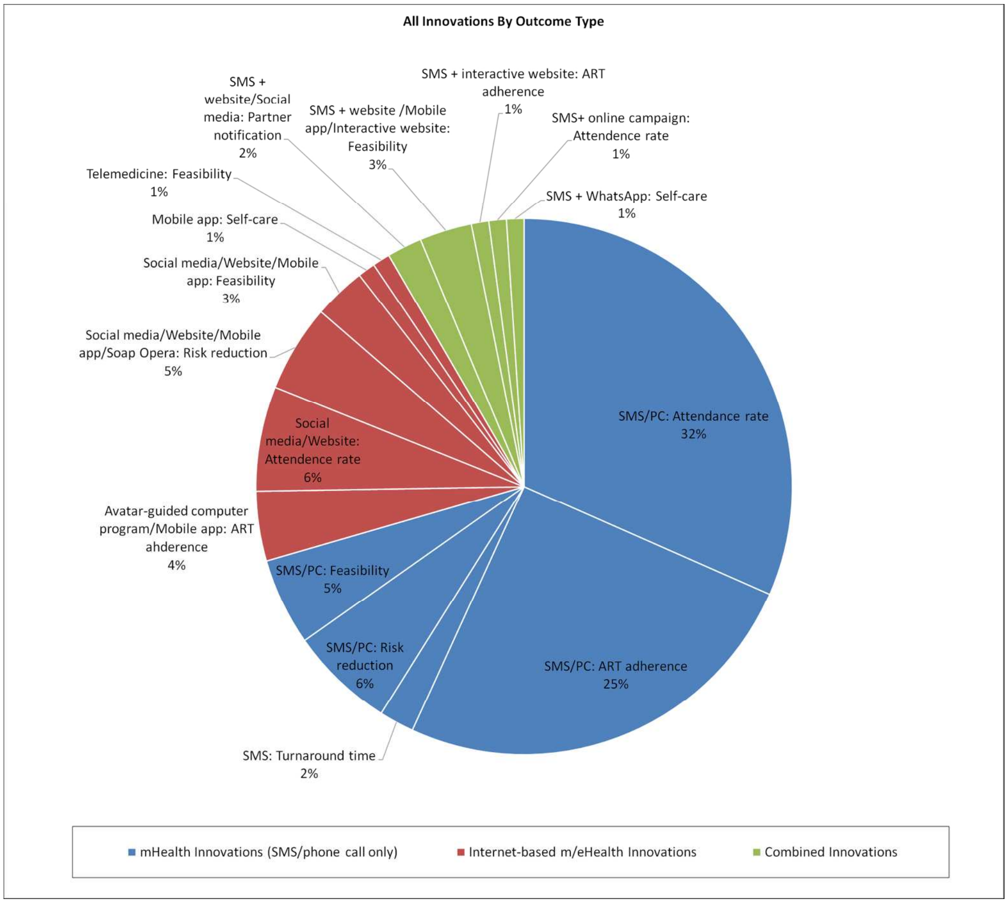

Figure 2: All innovations by outcome

170x152mm (300 x 300 DPI)

For peer review only

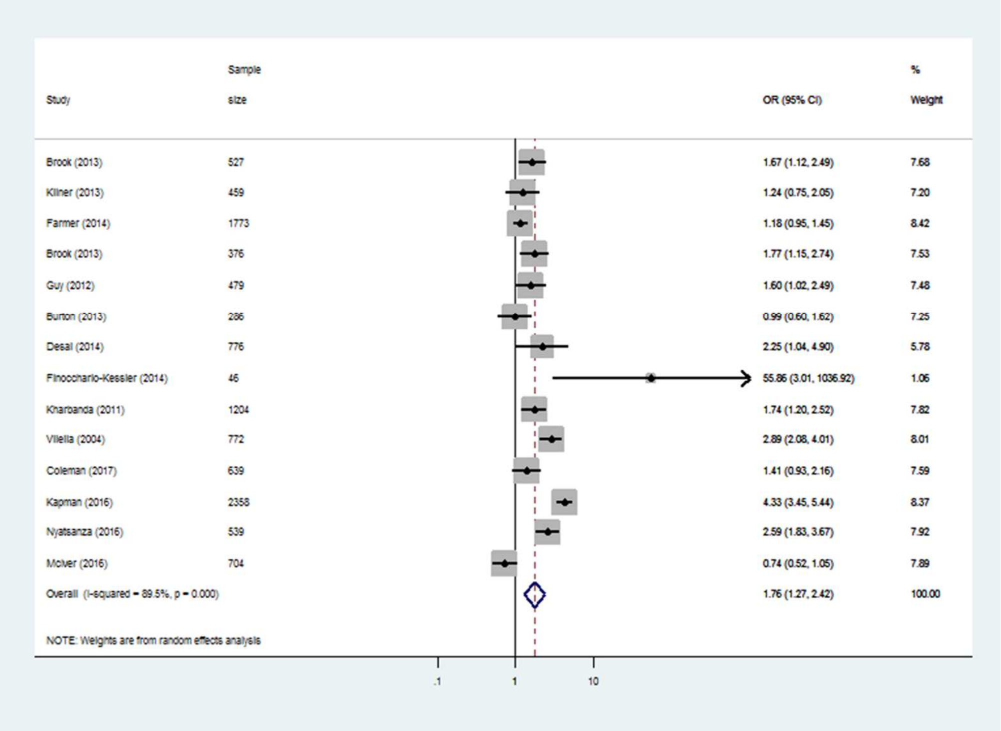

Figure 3A: Sub-group analysis pooled OR for attendance

101x74mm (300 x 300 DPI)

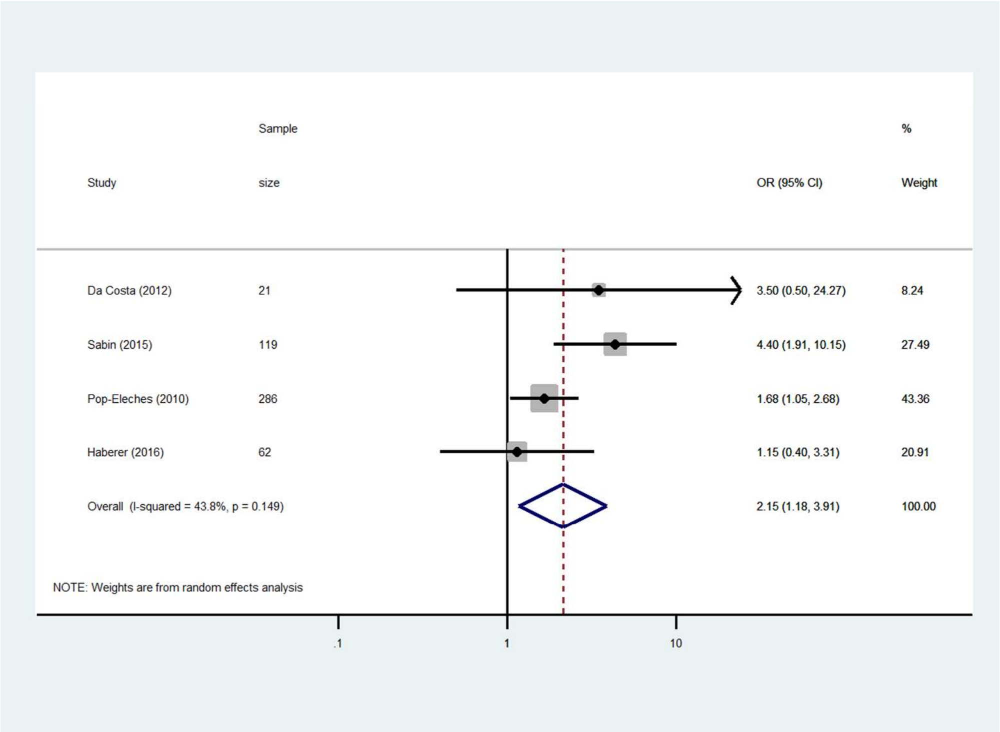

Figure 3B: Sub-group analysis pooled OR for adherence

108x79mm (300 x 300 DPI)

Appendix 1: Search Strategy.

Search #1	"HIV Infections"[Mesh] OR "HIV" [MeSH] OR “human immunodeficiency virus”[tiab] OR “human immuno deficiency virus”[tiab] OR “human immune deficiency virus”[tiab] OR “human immunedeficiency virus”[tiab] OR “aids”[tiab] OR “acquired immunodeficiency syndrome”[tiab] OR “acquired immunodeficiency syndromes”[tiab] OR “acquired immuno deficiency syndrome”[tiab] OR “acquired immuno deficiency syndromes”[tiab] OR “acquired immune deficiency syndrome”[tiab] OR “acquired immune deficiency syndromes”[tiab] OR “acquired immunedeficiency syndrome”[tiab] OR “acquired immunedeficiency syndromes”[tiab]
Search #2	"mHealth" [tiab] OR "telemedicine"[MeSH] OR telemedicine[tiab] OR eHealth[MeSH] OR ehealth[tiab] OR "mobile health" [tiab] OR “mobile technology”[tiab] OR “app”[tiab] OR “apps”[tiab] OR “mobile applications” OR social medi*[tiab] OR cell phone* [tiab] OR cellphone*[tiab] OR “cellular phone”[mesh] OR cellular phone*[tiab] OR smartphone*[tiab] OR smart phone*[tiab] OR mobile phone[tiab] OR mobile device*[tiab] OR cellular telephone*[tiab] OR mobile telephone*[tiab] OR text messag*[tiab] OR texting[tiab] OR texted[tiab] OR SMS[tiab] OR MMS[tiab] OR multimedia messag*[tiab] OR short messag*[tiab] OR “computers, handheld”[mesh] OR personal digital assistant*[tiab]
Search #3 [1,2]<br><br>References<br><br>1.Ferreira A, Young T, Mathews C, Zunza M, Low N. Strategies for partner notification for sexually transmitted infections, including HIV. Cochrane Database of Systematic Reviews 2013, Issue 10. Art. No.: CD002843. DOI: 10.1002/14651858.CD002843.pub2<br><br>2.Obiero J, Mwethera PG, Wiysonge CS. Topical microbicides for prevention of sexually transmitted infections. Cochrane Database of Systematic Reviews 2012, Issue 6. Art. No.: CD007961. DOI: 10.1002/14651858.CD007961.pub2	sexually transmitted infections[mh] OR sexually transmitted disease*[tiab] OR sexually transmissible disease*[tiab] OR sexually transmitted infection*[tiab] OR sexually transmissible infection*[tiab] OR sexually transmitted infectious disease*[tiab] OR sexually transmissible infectious disease*[tiab] OR sexually transmitted disorder*[tiab] OR sexually transmissible disorder*[tiab] OR STI[tiab] OR STIs[tiab] OR STD[tiab] OR STIs[tiab] OR venereal disease*[tiab] OR venereal infection*[tiab] OR venereal disorder*[tiab] OR genital herpes[tiab] OR herpes genitalis[mh] OR herpes genitalis[tiab] OR genital infection*[tiab] OR genital disorder*[tiab] OR herpes simplex[tiab] OR herpes virus[tiab] OR HSV-1[tiab] OR HSV-2[tiab] OR chancroid[mh] OR chancroid* [tiab] OR haemophilus ducreyi[tiab] OR chlamydia infection*[tiab] OR chlamydia trachomatis[mh] OR chlamydia trachomatis[tiab] OR gonorrhea[mh] OR gonorrhoea*[tiab] OR gonorrhea*[tiab] OR syphilis[mh] OR syphilis[tiab] OR cuminat[tiab] OR condylomata lata[tiab] OR chancre*[tiab] OR lymphogranuloma venereum[mh] OR lymphogranuloma venereum[tiab] OR granuloma Inguinale[mh] OR granuloma inguinale[tiab] OR donovania[tiab] OR donovanosis[tiab] OR calymmatobacterium[mh] OR calymmatobacterium granulomatis[tiab] OR klebsiella granulomatis[tiab] OR klebsiella granulomatis[tiab] OR treponema pallidum[mh] OR treponema pallidum[tiab] OR genital wart*[tiab] OR venereal wart*[tiab] OR condylomata cuminate[mh] OR human papillomavirus 6[mh] OR hpv-6[tiab] OR hpv-11[tiab] OR hpv6[tiab] OR human papillomavirus[tiab] OR hepatitis b[mh] OR hepatitis b[tiab] OR trichomonas vaginitis[mh] OR trichomonas vaginitis[tiab] OR genital ulcer*[tiab] OR anogenital ulcer*[tiab] OR anorectal ulcer*[tiab] OR anorectal ulcer*[tiab] OR penile ulcer*[tiab] OR blood-born pathogen*[tiab] OR blood-borne infection*[tiab] OR blood-borne virus*[tiab]
Search #4	#1 OR #3
Search #5	#2 AND #4

For peer review only

Appendix 2: **Abstraction table.**

Combined Innovations	Author	Study Design	Participants/ Country	STBBI	Intervention	Measure/Metric	Results (MD=Mean difference, RR=risk ratio, IRR=incidence rate ratio, HR=hazard ratio, SD= standard deviation, 95% CI when presented. M=months, W=weeks)
**Online campaign + SMS + TV.**	Friedman 2014	Quasi-experimental: HxCtrl w/ population data.	≤25 yrs, USA. n=N/A	HIV, CT, GC	GetYourselfTested: TV campaign w/ website & SMS service for STI info & clinic locator.	ATT testing: Attendance rate.	41.2% more CT tests in 2010 vs. 2008, 90.5% more GC tests, and 190.3% more HIV tests.
						Acceptability: Number of followers.	4477 FB followers and 1994 Twitter followers at yr 2.
						Feasibility: Referral rate.	83,404 referrals using clinic locator in yr1. 61,119 in yr2.
**Interactive website + SMS + cash incentives.**	Horvath 2013	RCT	HIV+ Gay/Bi-sexual men 18+ yrs, USA. n=67(Int) n=57(Ctrl)	HIV	Online self-monitoring system w/ interactive interface + optional SMS reminders +$25 gift card draw.	ART in PVLA: Self-report. [Difference scores: DS = FU-baseline]	No difference. (DS=0.54, SD=25.2 vs. DS=-3.2, SD=24.5; t(107)=1.79, p=0.43) / No impact.
						ART in PVLA: Self-report.	Increased adherence in drug users (DS= 7.1, SD= 22.1 vs. DS= -24, SD= 30.5; t(17)=2.52, p=0.02) / Effective.
						ART in PVLA: Self-report.	Trend to taking meds within 2hrs of scheduled dose. DS=6.6, SD=29.3 vs. DS=-3, SD=29.6; t(105)=1.68, p=0.1 / No impact.
						Acceptability: Self-report.	Mean score = 5.7 on 7-point Likert Scale for satisfaction / Highly acceptable.
						Feasibility: Completion rate.	Completion rate 88% vs. 93% in Ctrl / Highly feasible.
**Website + SMS**	Gotz 2014	Cross-sectional study.	STI index patients at clinic, NLD. n=988	HIV, CT, GC, syph	Suggestatest.nl: online partner notification via SMS/email.	PN: % partners notified.	14% notifications via SAT. 505 notifications sent (84% by SMS, 15% by email). 56% read notification. 20% visited one of 2 STI clinics.
**Social media + SMS.**	Hightow-Weidman 2014	Quasi-experimental: HxCtrl.	HIV+ or syphilis+ patients, USA. n=362(Int) n=133(HxCtrl)	HIV, syphilis	Notification on social networking sites + SMS	PN: % partners notified.	63.5% of contacts notified via internet in 2011 vs. 26% in 2010.
**PC/SMS/MMS + WhatsApp messages**	John 2016	UnCtrlled trial.	HIV+ non-disclosed, 15-29 yrs, NGA. n=19	HIV	Weekly counselling, educational & motivational calls, SMS/MMS and WhatsApp messages over 3M.	Self-care: Self-report.	Significant increase in self-care performance at 6MI (p=0.002)/ Effective.
**Interactive website + SMS**	Hightow-Weidman 2015	Feasibility study.	Black MSM & transwamen 18-30 yrs, USA. n=15	HIV	HealthMpowerment.org: online community networking Int to reduce STI risk + health promotion messages.	Acceptability: Self-report.	86.7%-100% strongly agreed w/ acceptability questions / Highly acceptable.
						Feasibility: Retention rate.	100% retention rate. 7/15 participants used the site 1W after study ended / Highly feasible.
**Mobile app + SMS**	Hirsch-Moverman 2017	Feasibility study.	≥18yrs, HIV+/TB, LSO. n=171	HIV/TB	CommCare application used to automatically send SMS medication reminders over 29M	Acceptability: Self-report.	41.9% think SMS facilitated adherence to TB /ART medication / Less acceptable.

For peer review only

**Mobile app + SMS**	Aronson 2016	Feasibility study	18-24 yrs, USA. n=100	HIV	App assessing risk and sending SMS to encourage re-testing of HIV negatives.	Feasibility: Completion rate	98/100 completed the app process/ Highly feasible<br>30/100 accepted to receive HIV test<br>21/30 accepted to receive SMS<br>1/21 re-tested after 90 days window period.
**Website + SMS**	Dokkum 2012	UnCtrlled trial.	16-29 yrs, NLD. n=52600(Rd 1) n=41700(Rd 2)	CT	At-home CT test + SMS/email to return test for analysis.	Feasibility: Completion rate.	Higher retesting rates (From 10% w/o reminders to 14% in round 1; from 7% to 10% in round 2) / Less feasible.

Note: Int= intervention; Ctrl= control; HxCtrl= historical control; PB= preventative behaviors (i.e. risk reduction); PN= partner notification; TAT= turnaround time; ATT= attendance rate; ART= ART adherence; NAPs= non-adherent patients; AP= adherent patients; PVLA= Patients with various levels of adherence; TNPs= Treatment naive patients; VL= viral load; CD4= CD4 cell count; PC= phone call; FB= Facebook.

For peer review only

Internet-based eHealth Innovation	Author	Study Design	Participants/ Country	STBBI	Intervention	Measure/Metric	Results (MD=Mean difference, RR=risk ratio, IRR=incidence rate ratio, HR=hazard ratio, SD= standard deviation, 95% CI when presented. M=months, W=weeks)
**Online campaign**	Downshen 2015	Quasi-experimental: HxCtrl w/ population data.	13-17 yrs, USA. n=1500	HIV, CT, GC, syphilis	IknowUshould2: social-media campaign w/ website for STI info & clinic locator.	ATT testing: Attendance rate.	More syphilis tests (18.8% vs. 5.4%; p<0.01) and HIV tests (19.0% vs. 5.4%; p<0.01). No change for CT & GC / Effective.
						Acceptability: Number of followers.	1500+ unique website interactions. 128 FB likes; 46 Twitter followers; 390 Youtube views; 42 Instagram followers.
**Social media campaign**	Elliot 2016	Cross-sectional study.	MSM, GBR. n=17361	HIV	Promotion through Gaydar, Grindr, Recon and FB pages to order free postal HIV home sampling kits	ATT testing: Participation rate.	10 323/11 127 (93%) ordered HIV sample kit. 5696/10 323 (55%) returned sample kit within 24M. 82/5696 (1.4%) confirmed new diagnosis and in care.
						Acceptability: Self-report.	59.7% would recommend to someone expected to test positive (93.8% if expected to negative). 64% clicked for more info on test. / Moderately acceptable.
**Social media campaign**	Huang 2016	Cross-sectional	≥18yrs, Black/African American or Hispanic/Latino MSM, USA. n=122	HIV	Promoting of HIV self-testing for 6W on GrindR + study website to order self-test kit	ATT testing: Participation rate.	122 requested tests; 55/57 HIV-, 2/57 HIV+.
						Acceptability: Number of followers	11 939 unique website visitors; 2.8% click-through rate 334 tests requested.
						Feasibility: Completion rate.	122/334 visitors were eligible and completed baseline survey, 81/122 confirmed receiving self test kit, 57/122 completed follow-up survey / Less feasible.
**Social media campaign**	Jones 2015	Cross-sectional study.	MSM, GBR. n=305	HIV	Health promotion and offer of rapid at-home testing via FB, Grindr, and Squirt.	ATT testing: Participation rate.	5/5 high risk sexual behavior but tested HIV negative; 1/5 never tested before; 3/5 not tested in many yrs.
						Acceptability: Number of followers.	103 clicked FB survey; 152 approached on Grindr; 50 Squirt contacts.
						Feasibility: Completion rate.	FB: 6/103 completed survey; 3/6 requested HIV test; 2/3 made appointment. Grindr: 20/152 engaged; 6/20 requests for at home test; 3/6 made appointment. Squirt: 3/50 engaged and 0/3 test requests / Less feasible.
**Social media campaign**	Rhodes 2016	Quasi-experimental.	MSM & transgender, USA n=339 (Int) n=286 (Ctrl)	HIV	Posting info and answering questions on HIV testing on social media sites (Adam4Adam, BlackGayChat, Craigslist, and Gay.com).	ATT testing: Self-report.	63.7% of intervention participants reported past 12M HIV testing compared with 42.0% of control. Adjusted OR= 2.9 (1.8-4.7)/ Effective.
**Social media campaign + website**	Rosengren 2016	Cross-sectional	Black or Hispanic MSM 18+ yrs, USA n=56	HIV	Promotion of free rapid HIV self-testing kits on Grindr and offer of delivery via study website (kit, voucher or pin for smart vending machine)	ATT testing: Self-report.	All 56 reported testing completion (100%); 2/56 reported positive result and linkage to care (confirmatory testing and ART initiation)
						Feasibility: Completion rate.	4389 visited the website; 333 requested test (i.e. 1 in 13 visitors); 56 completed survey 2W after request/ Less feasible.
						ART in TNPs: Self-report.	Higher adherence at 3M & 6M (71.2% vs. 63.9%, d=0.17; 70.3% vs. 66.6%, d=0.09)
**Mobile phone application**	Himelhoch 2016	RCT	18-64yrs, history of drug/alcohol use, HIV+, USA. n=19(Int) n=9(Ctrl)	HIV	Heart2HAART mobile application for ART adherence	ART in NAPs: Pill count	No significant difference in adherence between intervention and control group (p=0.29), but adherence was 100% in both at 3M / No impact
						Acceptability: Self-report.	94.3% strongly agreed/agreed Heart2HAART helped them take their medication / Highly acceptable.
						Feasibility: Response rate.	App was used on avg 21.4, 19.1 and 16.4 times in months 1, 2 and 3. Participants responded to medication prompts on avg 18, 16 and 14 times during months 1,2 and 3 respectively.

For peer review only
For peer review only - http://bmjopen.bmj.com/site/about/guidelines.xhtml

**Avatar-guided computer software**	Kurth 2014	RCT	HIV+ 18+ yrs, USA. n=120(Int) n=120(Ctrl)	HIV	Audio narrated risk assessment, skill building videos, tailored feedback and printouts vs. computer risk assessment only.	ART in PVLA: VL.	Non-significant change. (log10VL= -0.06(-0.4 to -0.3), p=0.74). Significant in subgroup w/ detectable VL at baseline (-0.73(-1.42 to -0.03), p=0.041) / No impact.
						ART in PVLA: Self-report.	Increased adherence. (4.71(0.95- 8.48) increase vs. 1.39(6.03 to 3.24) decrease; p=0.046) / Effective.
						PB: Self-report.	Lower odds of HIV transmission (OR=0.46 (0.25-0.84), p=0.012) / Effective.
						Acceptability: Self-report.	97% reported ease of use and high privacy; 99% satisfied w/ session length; 75% preferred it over human counsellor / Highly acceptable.
						Feasibility: Retention rate.	87.1% retention / Highly feasible.
**Avatar-guided computer program**	Naar-King 2012	RCT	HIV+ 16-24 yrs, USA. n=36(Int) n=40(Ctrl)	HIV	2-D animated character delivering personalized health feedback vs. character giving nutrition info.	ART in TNPs: VL.	Larger suppression rate. (Cohen's d=0.09 at 3M; d= 0.28 at 6M). Larger drop in VL from baseline (d=0.39 at 3M & d=0.19 at 6M).
						ART in TNPs: Self-report.	Higher adherence at 3M & 6M (71.2% vs. 63.9%, d=0.17; 70.3% vs. 66.6%, d=0.09)
						Acceptability: Self-report.	Mean satisfaction ratings 3.7 out of 4 / Highly acceptable.
**Mobile phone application**	Perera 2014	RCT	HIV+, NZ. n=17(Int) n=11(Ctrl)	HIV	ART adherence app w/ medication clock & graphs on disease-state vs. standard app (medication clock only)	ART in PVLA: Self-report.	Increased adherence (F(1,23)=5.37, p=0.03) / Effective.
						ART in PVLA: Pharmacy refills.	No difference. (F(1,25)=1.88, p=0.18) / No impact.
						ART in PVLA: VL.	Lower VL at 3M (F(1,23)=5.62, p=0.023) / Effective.
						ART in PVLA: Composite score (refills, VL, & self-report).	Increased adherence (53% to 13%, X2(1,15)=6, p=0.03). No change in Ctrl (27% to 27%, X2(1,11)=0.00, p>0.99) / Effective.
						Acceptability: Self-report.	More satisfying (on 11 point-scale: 5.88 vs. 3.27, p=0.017) and informative (6 vs. 3, p=0.034) at 3M than standard app / Highly acceptable.
**Mobile app + cash incentive**	Brayboy 2017	UnCtrlled trial.	12-17yrs, USA. n=17	STI	GirlTalk mobile phone app to assess knowledge increase	PB: Self-report.	75.6% to 79% increase in knowledge pre and post app use at 2W. / No impact.
						Acceptability: Self-report.	94.1% would use the app again/recommend it / Highly acceptable
**Social media**	Jones 2012	Quasi-experimental: HxCtrl.	15–24 yrs, USA. n=70/896 FB friends	CT	Educational FB site addressing safe sexual health.	PB: Self-report.	Condom from 57% to 80%. 54% reduction in CT in ages 15-17 from previous yrs (but 42% less tests done).
**Videos vs. SMS**	Jones 2013	RCT	High-risk urban African-American women 18-29 yrs, USA. n=117(Soap opera) n=121(SMS)	HIV	Weekly soap opera episodes (Love, Sex & Choices) vs. HIV prevention SMS.	PB: Self-report.	18% greater reduction in Int. group, p=0.23 / No impact. 78% reduction in risky acts from baseline in Int. group (p<0.001); 72% reduction from baseline in Ctrl (p<0.001)/ Effective
						Acceptability: Self-report.	97.4% liked the videos / Highly acceptable.
**Social media + video chat**	Lelutiu-Weinberger 2014	UnCtrlled trial.	MSM 18-29 yrs, high risk for STI, USA. n=31	HIV	miCHAT: FB chat Int. 8 motivational interviews to reduce HIV risk + CBT training.	PB: Self-report.	Decrease in unprotected anal sex acts (3.11 vs. 8.96; p=0.042). Increased knowledge of sexual risk (p=0.01) / Effective.
						Acceptability: Self-report.	All felt privacy was ensured / Highly acceptable.
						Feasibility: Completion rate.	46% completed baseline assessment + minimum 5 sessions / Less feasible.

For peer review only

**Social media campaign + website + cash incentive**	Solorio 2016	Feasibility study.	Hispanic MSM, 18-30 yrs, USA<br>n=50	HIV	Radio & social media-based campaign for 16W to encourage testing & condome use + website w/clinic locator to provide free HIV home testing kits and linkage to care	PB: self-report.	No significant change in condom use at 16W (26.1% vs. 15.65, OR=1.9 (0.6-5.9))/ No impact.
						Feasibility: Self-report.	32/50 (64%) requested HIV home testing kit, 28/32 (88%) completed the test/ Moderately feasible.
**Mobile app**	Jeon 2016	RCT.	Chronic HBV+, 19-60 yrs, KOR<br>n=26 (Int)<br>n=27 (Ctrl)	HBV	App to increase disease knowledge, set alarm medication reminders, record lab nutrition & physical activity data, and chat with other users.	Self-care: Self-report.	Significantly higher self-care performance in intervention vs. control (t=3.597, p=0.001)/ Effective.
						Feasibility: Utilisation rate.	Average monthly utilisation rate was 75.1%/ Highly feasible.
**Social media**	Henwood 2016	Feasibility study.	12-25 yrs, HIV+, ZAF<br>n=90	HIV	Use of MXit as support group for HIV+ youth	Acceptability: Self-report.	84% would like chat-room to continue / Highly acceptable.
						Feasibility: Participation rate	33% ever visited MXit chat-room / Less feasible.
**Mobile app + cash incentive**	Przybyla 2016	Feasibility study.	HIV + on ART, 18+ yrs, USA<br>n=27	HIV	DRUM app to report daily on ART adherence and substance abuse.	Acceptability: Self-report.	84% reported the app was easy to use; 96% were satisfied; 92% would use it in the future/ Highly acceptable.
						Feasibility: Completion rate.	Overall completion rate of daily reports after 2W= 95.3%/ Highly feasible.
**Telemedicine**	Talal 2016	Feasibility study.	Individuals on opioid agonist tx, USA<br>n=54	HCV	Telemedicine-based medical tx with hepatologist	Acceptability: Self-report.	88.9% prefer medical tx using telemedicine vs. clinic visit; 100% would recommend it to a friend/ Highly acceptable.
						Feasibility: Completion rate.	54 tested HCV+ over 14M; 81.5% started evaluation/tx; 75% of those given tx have completed it/ Highly feasible.
**Social media**	Garett 2016	Feasibility study.	18+yrs, MSM, PER<br>n=102(Int)<br>n=109(Ctrl)	HIV	12W FB based peer-led intervention to increase HIV testing and prevention behaviour.	Acceptability: Self-report.	Intervention group felt they learned more about; where to receive sexual health services (p-value=0.0061), more likely to have safe sex (p-value=0.034) and more likely to get tested for HIV regularly (p-value=0.021) compared to control group / Highly acceptable.
**Website**	Polilli 2016	Feasibility study.	Residents of Abruzzo Region, ITA<br>n=3500	HIV, syphilis, HBV, HCV	Website with STI info, risk calculator, and appointments booking at testing sites.	Feasibility: Completion rate.	3500 booked an appointment; 3046 (87%) presented for testing within 15M study period/ Highly feasible.

Note: Int= intervention; Ctrl= control; HxCtrl= historical control; PB= preventative behaviors (i.e. risk reduction); PN= partner notification; TAT= turnaround time; ATT= attendance rate; ART= ART adherence; NAPs= non-adherent patients; AP= adherent patients; PVLA= Patients with various levels of adherence; TNPs= Treatment naive patients; VL= viral load; CD4= CD4 cell count; PC= phone call; FB= Facebook.

For peer review only

Basic mHealth Innovation	Author	Study Design	Participants/ Country	STBBI	Intervention	Measure/Metric	Results (MD=Mean difference, RR=risk ratio, IRR=incidence rate ratio, HR=hazard ratio, SD= standard deviation, 95% CI when presented. M=months, W=weeks)
**SMS**	Bailey 2014	UnCtrlled trial.	CT+ at clinic, AUS. n=64	CT	SMS reminders to recall for treatment.	ATT treatment: Attendance rate.	100% treated for CT infection. 72% treated within 1 day of SMS.
						Feasibility: Response rate.	94% replied to SMS, 84% the same day / Highly feasible.
**SMS + PC**	Bassett 2016	RCT	≥18yrs, ZAF. n=543(Int) n=471(Ctrl)	HIV/TB	5 scheduled PC) and 4 SMS, reminders to retrieve test results and attend appointments, over 4M.	ATT treatment: Attendance rate.	No significant difference in reaching outcome at 9M (3M ART treatment or 3+6M of TB treatment) between intervention and control (39% vs 42%, RR=0.93, 95%CI 0.80-1.08) / No Impact
**SMS + PC**	Bigna 2014	RCT	Caregivers of HIV + children 18+ yrs, CMR. n=61(SMS+PC) n=60(PC) n=60(SMS) n=61(Ctrl)	HIV	SMS+PC, SMS, or PC appointment reminders.	ATT FU appointment: Attendance rate.	Improved attendance. (OR=2.9 (1.3-6.3), p=0.012) / Effective.
**SMS**	Brook 2013	Quasi-experimental: HxCtrl.	Sexual health clinic, GBR. n=207(Int) n=169(HxCtrl)	HIV, syphilis, HBV	SMS reminders.	ATT testing: Attendance rate.	Higher retesting rate (41% vs. 28%; p<0.001) / Effective.
**SMS**	Brook 2013	Quasi-experimental: HxCtrl.	Sexual health clinic, GBR.n=699(Int) n=768(HxCtrl)	HIV	SMS reminders 2 days before appointment.	ATT FU appointment: LTFU rate.	35% improvement in overall LTFU rate (26% to 17%; p<0.0001) / Effective.
**SMS**	Burton 2013	Quasi-experimental: HxCtrl.	High risk for STI at clinic, GBR. n=273(Int) n=266(Ctrl)	CT, GC	SMS STI testing reminders.	ATT: testing: Attendance rate.	No change in retesting rates for those w/ recent CT or GC. (CT: 36% vs.33%; p=0.79) (GC: 19% vs. 33%; p=0.48) / No impact.
**SMS**	Coleman 2017	Retrospective Quasi-experimental	>=18 yrs, HIV+ pregnant women, ZAF. n=192(Int) n=447(Ctrl)	HIV	Bi-weekly maternal health info sent throughout pregnancy and for one year after birth to increase HIV PCR testing postpartum and increase ANC visits	ATT testing: Attendance rate.	81.3% vs 75.4% in intervention vs control group likely to attend first PCR 6W postpartum. 40% increase in the likelihood of attending the recommended four ANC visits among individuals within the intervention group (RR: 1.41, CI: 1.15–1.72) / Effective.
						PB: Infection rate	3 infants born with HIV in control group
**SMS**	Desai 2014	Quasi-experimental: Conc. + HxCtrl.	High risk MSM at clinic, GBR. n=31(Int) n=656(Conc. Ctrl) n=745(HxCtrl)	HIV	SMS HIV/STI testing reminders.	ATT testing: Attendance rate.	No significant change in re-testing odds. (32% in SMS vs.30% in Conc. Ctrl; OR=1.1(0.5-2.4) and 17% in HxCtrl; OR=2.3(1.0-4.9) / No impact.
**SMS + cash incentive**	Downing 2013	RCT	CT + or suspected at clinic 16+ yrs, AUS. n=30(Int) n=32(Ctrl)	CT	SMS appointment reminders + $10 if attended.	ATT testing: Attendance rate.	Increased re-testing rate at 10-12W post CT treatment (without cash 26.7% vs. 6.3% in Ctrl; p=0.04); (with cash 28.1% vs. 6.3% in Ctrl; p=0.043) / Effective.
**SMS**	Evans 2015	UnCtrlled trial.	African community, GBR. n=172	HIV	2 weekly Health Belief Model SMS to reduce risky sexual behaviours.	ATT testing: Self-report.	10.5% reported being tested for HIV during/after the 12W Int.
						PB: Self-report.	Non-significant increase in HIV knowledge & attitudes / No impact.
						Acceptability: Self-report.	Acceptable & useful. Majority shared w/ others and want to get tested in future.

For peer review only - http://bmjopen.bmj.com/site/about/guidelines.xhtml

**SMS**	Farmer 2014	Quasi-experimental: HxCtrl.	HIV clinic attendees, GBR. n=951(Int) n=822(HxCtrl)	HIV	SMS reminder 2 days before appointment.	ATT FU appointment: LTFU & cancellation rate.	No difference in LTFU (25% vs.28%) or cancellation (62% vs.64%) / No impact.
**SMS**	Finocchario-Kessler 2014	Quasi-experimental: HxCtrl.	HIV+ mother-infant pairs, KEN. n=523(Int) n=320(HxCtrl)	HIV	SMS notification of available test results and appointment reminder.	ATT treatment: Attendance rate.	More infants initiated on ART (Urban: 11/11 vs. 1/7, p<0.001; Peri-urban: 14/14 vs. 9/14, p<0.05) / Effective.
						TAT: Time from test to diagnosis & test to treat.	Shorter median time to diagnosis (5 vs. 6.3W (urban) & 3.4 vs. 8.1W (peri-urban); both p<0.001). Shorter median time to treat (13 vs. 40 days (urban) & 1 vs. 36 days (peri-urban); p<0.001) / Effective.
						Feasibility: Retention rate.	Retention rate double at 9M post-natal (45.1% vs. 93% (urban) and 43.2% vs. 94.1% (peri-urban); p<0.001) / Highly feasible.
**SMS**	Guy 2012	Quasi-experimental: HxCtrl.	STI clinic, AUS. n=141(Int) n=338(HxCtrl)	CT	SMS re-testing reminder 3M after initial infection.	ATT testing: Attendance rate.	Higher retesting rate (30% 1-4M post-infection vs. 21%; p=0.04); AOR= 1.57(1.01-2.46) / Effective.
**SMS**	Joseph Davey 2016	RCT.	HIV+ adults on ART, MOZ n=416 (Int) n=414 (Ctrl)	HIV	SMS reminders 2 and 7 days of appointment and ART drug-pick up + educational SMS every 2M.	ATT treatment: Attendance rate.	Nonsignificant difference in overall retention in care at 12 M (93.8% vs 91%, p=0.139)/ No impact.
**SMS**	Kapman 2016	Quasi-experimental: HxCtrl.	Heterosexual clinic attendees dx & tx for CT, 16-23 yrs, NLD n=828 (Int) n=1530 (Ctrl)	CT	2 SMS reminders at 5.5M & 6M after initial dx with CT for retesting appointment scheduling & attendance.	ATT testing: Attendance rate.	Higher attendance rate between 5-8M after initial dx (30.6% vs. 9.2%).
**SMS**	Kharbanda 2011	Quasi-experimental: Conc. + HxCtrl.	Parents of girls 9-20 yrs at clinics, USA. n=124(Int) n=308(Conc. Ctrl) n=1080(HxCtrl)	HPV	Up to 3 weekly SMS vaccination reminders.	ATT vaccination: Attendance rate.	More likely to get vaccine on time after controlling for insurance and site of care (AOR=1.83(1.23-2.71)) / Effective.
**SMS**	Kliner 2013	Quasi-experimental: HxCtrl.	HIV+ at hospital, SWZ. n=162(Int) n=297(HxCtrl)	HIV	SMS reminders one day before appointment.	ATT FU appointment: Attendance rate.	No difference. SMS 83.3% vs. Ctrl 80.1%; p=0.401. AOR=1.13, p=0.662 / No impact.
**SMS**	Matheson 2014	Quasi-experimental.	11-22 yrs at clinic, USA. n=37(Int) n=232(Ctrl)	HPV	SMS vaccination reminders (3 SMS per dose).	ATT vaccination: Attendance rate.	Higher attendance rate. HPV2 vaccine complete: 73% vs.34%, (p=0.000); on-time HPV2 38% vs. 25%, (p=0.035). HPV3 complete 16% vs.6%, (p=0.018); on-time HPV3 14% vs.3%, (p=0.007) / Effective.
**SMS**	McIver 2016	Quasi-experimental: HxCtrl.	Clinic attendees susceptible to HBV (HIV+, bisexual, CSW, IDUs, Aboriginals), AUS n=241 (Int) n=463 (Ctrl)	HBV	SMS reminders 1 day before appointment for HBV vaccine doses 2&3 reattendance.	ATT vaccination: Attendance rate.	Nonsignificant decrease in attendance rate within 12 M (54% vs 56% for 2 doses, p=0.65/ 24% vs 30% for 3 doses, p=0.07)/ No impact Nonsignificant difference in completion of 3 doses in 12M. aOR= 0.7 (0.48-1.01)/No impact.
**SMS**	Njuguna 2016	RCT.	Rural women, 18-24 yrs, KEN n=300 (Int) n=300 (Ctrl)	HIV	Weekly SMS on HIV and reproductive health.	ATT testing: Self-report.	Significant increase in reported testing at 6M (67% vs 51%, aHR=1.54(1.25-1.90)/ Effective.
**SMS vs. PC**	Norton 2014	RCT	HIV+, 17+ yrs, USA. n=25(Int) n=27(Ctrl)	HIV	SMS appointment reminder vs. message to home phone.	ATT FU appointment: Attendance rate.	No difference (72% vs. 81%, p=0.42) but patients already had high attendance rate / No impact.

For peer review only

**SMS**	Nyatsanza 2016	Quasi-experimental: HxCtrl.	MSM & CSW at high-risk of STI, GBR n=266 (Int) n=273 (Ctrl)	HIV/STI	Personalised SMS reminders for reattendance.	ATT testing: Attendance rate.	Significantly higher reattendance rate at 6M (56% vs. 33%, p<0.001)/ Effective.
**SMS**	Odeny 2012	RCT	Males circumcised at clinic 18+ yrs, KEN. n=600(Int) n=600(Ctrl)	HIV	Daily SMS for 1W.	ATT FU appointment: Attendance rate.	Improved attendance within 3 days of post-operative clinic appointment: 65.4% vs.59.7% (RR=1.09(1.00–1.20); p=0.04) / Effective.
						PB: Self-report.	Abstention of sexual activity before FU: 28.3% vs. 25.2% (RR=1.13(0.91-1.38), p=0.3) / No impact.
**SMS**	Rand 2015	RCT	11-16 yrs at clinic, USA. n=1893(Int) n=1919(Ctrl)	HPV	SMS appointment reminders.	ATT vaccination: Attendance rate.	Higher HPV1 vaccination rate (16% vs. 13%; HR= 1.3(1.0-1.6); p=0.04) / Effective.
**SMS/PC**	Rand 2016	RCT.	Clinic attendees Parents of youth 11-17 yrs who received 1st HPV vaccine, USA. n=191 (SMS) n=200 (Ctrl); n=178 (PC) n=180 (Ctrl)	HPV	SMS appointment reminders to receive 3 doses of HPV vaccine over 2 yrs.	ATT vaccination: Attendance rate.	SMS: Significant difference in vaccination rates compared to control (49% vs 30%, p=0.001)/ Effective.<br>PC: No difference in vaccination rates compared to control (48% vs 40%, p=0.34)/ No impact.
						TAT: Time from enroll to completion of 3 vaccines.	SMS: Significant difference in time taken to complete 3 HPV doses (71 days earlier than control, p<0.001)/ Effective.<br>PC: No difference in time taken to complete 3 HPV doses compared to control (p=0.08)/ No impact.
**SMS + PC**	Schwartz 2015	Quasi-experimental: HxCtrl.	HIV+ pregnant women on ART, ZAF. n=50	HIV	SMS messages and PCs from a case manager (CM) through 6W postpartum.	ATT testing: Attendance rate.	More infant testing (90.0% vs. 63.3% at 10W; p<0.01) / Effective.
						Acceptability: Self-report.	Helpful to have CM support during pregnancy and postpartum (98%) / Highly acceptable.
						Feasibility: Completion rate.	96% completed postpartum questionnaire / Highly feasible.
**SMS + PC**	Segaren 2012	UnCtrlled trial.	Mothers of HIV+ infants, HTI. n=108	HIV	Cell phones + regular PC for monitoring of mother & child.	ATT treatment: Attendance rate.	All 76 w/ active phones were adherent to treatment (attended 6/6 monthly hospital appointments).
						Acceptability: Self-report.	70% phones active after Int.; good for med reminders (63%) / Moderately acceptable.
**SMS + PC**	Smillie 2014	UnCtrlled trial.	HIV+ in clinic 14+ yrs, CAN. n=20	HIV	Weekly PC or SMS for 6M.	ATT FU appointment: Self-report.	65% said SMS had no effect on attendance.
						Acceptability: Self-report.	Beneficial for appointment scheduling (80%) & reminder (75%). All would recommend to a friend / Highly acceptable.
						Feasibility: Self-report.	75% had no difficulty in receiving and responding to SMS / Highly feasible.
**SMS**	Tolly 2012	RCT	Randomly sampled adults (existing database), ZAF. n=438(in each of 4 Int.) n=801(Ctrl)	HIV	3 or 10 motivational or informational SMS.	ATT testing: Self-report.	Improved attendance in group receiving 10 motivational SMS at 3W: (69% vs. 57%; OR=1.7(1.10–2.390), p=0.0036) / Effective.
						Feasibility: Self-report.	SMS motivated HIV counseling and testing uptake in 89% / Highly feasible.
**SMS**	Vilella 2004	Quasi-experimental: Conc. + HxCtrl.	18+ yrs at travel clinic, ESP. n=738(Int) n=1610(Conc. Ctrl) n=2247(HxCtrl)	HAV/ HBV	SMS reminders for vaccination appointments.	ATT vaccination: Attendance rate.	Improved adherence for 3rd HepA+B dose. (47.1% vs. 26.9%, RR=1.75(1.41–2.17) in Conc. Ctrl and 23.6%(20.1–27.4), RR=2.00(1.63–2.45) in HxCtrl) / Effective.

For peer review only

**SMS**	Ammassari 2010	UnCtrlled trial.	HIV+, ITA. n=71	HIV	SMS reminders.	ART in NAPs: Self-report.	Increased adherence over 9M. (93.2% vs.79.6%, p=0.003) / Effective.
**SMS**	Ammassari 2011	UnCtrlled trial.	HIV+, 18+ yrs, ITA. n=145	HIV	SMS reminders.	ART in NAPs: Self-report.	Increased adherence at 9M (94.9% vs.78.8%, p<0.001) / Effective.
						ART in NAPs: VL.	More w/ undetectable VL at 9M (76.2% vs. 42.3%, p<0.001) / Effective.
						Acceptability: Self-report.	>90% reporting SMS helpful / Highly acceptable.
**PC + cash incentives**	Belzer 2014	RCT	HIV+ 12-29 yrs, USA. n=19(Int) n=18(Ctrl)	HIV	Daily PC reminders and referrals if necessary+ free phone & plan.	ART in NAPs: Self-report.	Increased adherence for 1M &3 M (OR=3.09(1.20-7.98); OR=2.85(1.02-7.97)) / Effective.
						ART in NAPs: VL.	Lower VL at wk 24 and 48 (2.82 vs. 4.52, p=0.002; 3.23 vs. 4.23, p=0.043) / Effective.
**SMS**	Cantudo-Cuenca 2016	Retrospective quasi-exprimental.	HIV + on ART, ESP n=120 (Int&Ctrl)	HIV	SMS on ART adherence.	ART in PVLA: Pharmacy refills.	Statistically sign relationship bt no SMS and ART adherence(OR= 0.35 (0.14-0.8), p=0.025) [multivariate analysis]/ Effective.
**SMS**	da Costa 2012	RCT	HIV+ women, BRA. n=8(Int) n=13(Ctrl)	HIV	Daily SMS reminders.	ART in APs: Pill count.	Increased adherence over 4M (50% vs. 38.5%; p=0.604) / No impact.
						ART in APs: MEM.	Increased adherence over 4M (75% vs. 46%; p=0.195) / No impact.
						ART in APs: Self-report.	Increased adherence (100% vs. 84.6% in Ctrls; p=0.244) / No impact.
						Acceptability: Self-report.	82% believed SMS were helpful, 77% wanted to keep receiving SMS / Highly acceptable.
**SMS**	Downshen 2011	UnCtrlled trial.	HIV+ 14-29 yrs, AUS. n=25	HIV	Daily SMS ART reminder + FU SMS 1hr later.	ART in NAPs: Self-report.	Increased adherence (Baseline Mean=74.7; 12W Mean=93.3; 24WMean=93.1; p<0.001) / Effective.
						ART in NAPs: VL + CD4 count.	Insignificant change in CD4 cell count & VL (mean VL= 2750, CD4= 502 to VL= 29, CD4= 545 at 24W, p=0.12) / No impact.
						Acceptability: Self-report.	81% want SMS after study end. Helped decrease missed doses in 95% / Highly acceptable.
**SMS**	Downshen 2011	UnCtrlled trial.	HIV+ 14-29 yrs, AUS. n=25	HIV	Daily SMS ART reminder + FU SMS 1hr later.	ART in NAPs: Self-report.	Decreased adherence (58.3% for 0-12W vs. 55.2% for 13-24W, p=0.53) / No impact.
						Feasibility: Completion & response rate.	84% completed all study visits. 61.4% response rate / Highly feasible.
**SMS + cash incentive**	Garofalo 2016	RCT	16-29yrs, HIV+ on ART for ≥1M , USA. n=51(Int) n=54(Ctrl)	HIV	Daily personlised SMS over 6M to remind participants take medications	ART in NAPs: Self-report.	Significant difference in adherence compared to control at 3M OR=2.57 (1.01-6.54). Not significant at 6M OR=1.68 (0.69-4.09). Significant difference from baseline to 6M OR=2.12 (95% CI 1.01-4.45). / Effective.
						ART in NAPs: VL.	No difference in log viral load or viral suppression compared to control at 3 and 6M / No impact.
						Acceptability: Self-report.	100% would recommend intervention to those in need, 81 % wanted to continue getting the text messages after conclusion of the study, 95 % satisfied with the intervention overall / Highly acceptable
						Feasibility: Response rate.	58% average response rate to SMS / Moderately feasible.

For peer review only

**SMS +PC**	Haberer 2016	RCT	≥18yrs, HIV+ on ART, UGA. n=21(Scheduled SMS) n=20 (Triggered SMS) n=21(Ctrl)	HIV	Scheduled SMS: 1M daily SMS, 2M weekly SMS, 6M SMS sent to patient and support if needed. Triggered SMS; SMS sent to patient and support if no signal received from monitor.	ART: MEM	Significant difference in scheduled SMS intervention compared to control (11.1% increase in adherence, 48-h and more than 96-h lapses were less frequent (IRR=0.6, p value=0.02 and IRR 0.3, P<0.001, respectively). Similar adherence in triggered SMS vs control group. / Effective.
						ART: VL	No significant differences in HIV RNA suppression among study arms (p value = 0.14). 47/62 participants virally suppressed at 3 and 9M / No impact.
**SMS**	Hardy 2011	RCT	HIV+ 18+ yrs, USA. n=12(SMS) n=14(Beeper)	HIV	SMS vs. beeper reminders.	ART in APs: Composite score (MEM+ pill count + self-report).	Higher adherence at 6W. (MD=27.1(7.6-46.6), p =0.009) / Effective.
						ART in APs: MEM.	Increased adherence. (MD=33.4(14.1-52.6), p = 0.002) / Effective.
						ART in APs: Pill count.	No difference. (MD=13.7(-6.7-34.1), p = 0.153) / No impact.
						ART in APs: Self-report.	No difference. (MD=20.2 (-1.8-42.1), p = 0.069) / No impact.
**SMS**	Jeffries 2016	RCT	15-24yrs, HIV+, USA. n=91(Int) n=45(Ctrl)	HIV	UCARE4LIFE daily moblie text messageing intervenetion over 3M to improve HIV care among youth	ART: VL	Significant difference in ART adherence in intervention vs control among non-adherent/new to ART at baseline (6M p=0.03). / Effective. No sig difference in those on ART at baseline (6M p=0.119) /No impact.
						Acceptability: Self-report.	Mean score = 8.44 (SD=2.45) on 10 point Likert Scale for appointment reminder SMS./ Highly acceptable
**PC**	Kalichman 2011	RCT	HIV+ 18+ yrs, USA. n=21(Int) n=19(Ctrl)	HIV	PC counselling.	ART in NAPs: Pill count.	No difference at 4M (F(1,36)=3.32, p<0.07) / No impact.
						Feasibility: Completion rate.	99% completion rate / Highly feasible.
**SMS**	Kassaye 2016	RCT.	HIV+ pregnant women, KEN n=280 (Int) n=270 (Ctrl)	HIV	3 to 6 weekly SMS (ART reminders, motivational, PMTCT, child health & nutrition).	ART in TNPs: Self-report.	Nonsignificant difference in adherence to ART at 34-36W gestation between the 2 groups (97.3% vs 99.6%, aRR= 1.25 (0.43-3.60)./No impact. Nonsignificant difference in adherence to ART at delivery between the 2 groups (94.7% vs 100%, aRR=1.01 (0.88-1.16))./ No impact.
**PC**	Kebaya 2014	RCT	HIV+ mothers in PMTCT, KEN. n=75(Int) n=75(Ctrl)	HIV	Bi-weekly PC.	ART in TNPs: Self-report.	Increased adherence (90.7% vs. 72%, p=0.005) / Effective.
						Feasibility: Retention rate.	More likely to remain in treatment at 10W (69.3% vs 37.3%, p<0.001) / Moderately feasible.
**SMS**	Lester 2010	RCT	HIV+ 18+ yrs, KEN. n=273(Int) n=265(Ctrl)	HIV	Weekly SMS.	ART in TNPs: Self-report.	Improved adherence at 6M and 12M: RR=0.81(0.69-0.94) p=0.006 / Effective.
						ART in TNPs: VL.	Lower virological failure (RR=0.84(0.71-0.99) p=0.04) and improved viral suppression (OR=0.71(0.5-1.01) p=0.058) / Effective.
**SMS + PC + cash incentives**	Maduka 2013	RCT	HIV+ at hospital 20+ yrs, NGA. n=52(Int) n=52(Ctrl)	HIV	2 monthly counselling PCs + 2 weekly SMS+ cash incentives	ART in NAPs: Self-report.	Increased adherence (76.9% vs. 55.8%, X2=5.211,p=0.022; RR=0.725(0.55-0.96)) / Effective.
						ART in NAPs: CD4 count.	Improved CD4+ count (193-->575 cells/mL vs. 131-->361.5 cells/mL; p=0.007) / Effective.
**SMS + PC**	Mbuagbaw 2012	RCT	HIV+ 21+ yrs, CMR. n=101(Int) n=99(Ctrl)	HIV	Weekly motivational SMS. Phone number to call for support.	ART in PVLA: Self-report.	No difference. (RR=1.06(0.89-1.29); p=0.542) / No impact.
						ART in PVLA: Pharmacy Refills.	No difference at 6 months (MD=0.1(-0.23-0.43); p=0.617) / No impact.
						Acceptability: Self-report.	91.1% believed SMS reminders helped; 65% were satisfied; 81.2% would recommend to a friend / Highly acceptable.

For peer review only

**SMS**	Moore 2015	RCT	HIV+ bipolar 18+ yrs, USA. n=25(Int) n=25(Ctrl)	HIV	SMS reminders.	ART in PVLA: MEM.	No difference. (86.2% (SD= 12.7) vs. 84.8% (SD= 18.1); p=0.95; d=0.01) / No impact.
**SMS**	Nsagha 2016	RCT.	HIV+ on ART, 18+yrs, CMR n=45 (Int) n=45 (Ctrl)	HIV	4 weekly educative SMS over 1M.	ART in PVLA: Self-report.	Nonsignificant difference in adherence to ART at 1M between the 2 groups (64.4% vs 44.2%, p=0.056)/ No impact.
						Acceptability: Self-report.	57.8% wished the SMS to continue/ Moderately acceptable
**SMS**	Pop-Eleches 2010	RCT	HIV+ 18+ yrs, rural KEN. n=142(Daily SMS) n=147(Weekly SMS) n=139(Ctrl)	HIV	Daily or weekly SMS.	ART in PVLA: MEM.	Increased adherence in weekly SMS group over 48W (53% vs. 40% p=0.03) / Effective.
						ART in PVLA: MEM.	No difference between daily SMS group and Ctrl (41% vs. 40% p=0.92) / No impact.
**SMS + cash incentive**	Rana 2016	UnCtrlled trial.	HIV+, 18+yrs, USA. n=32	HIV	Bi-directional weekly SMS appointment reminders, daily ART reminder & supportive messages.	ART in PVLA: Undetectable VL	Significant increase in the number of participants with undetectable VL at 6M (25 vs. 18, p=0.002)/ Effective.
						ATT treatment: Attendance rate.	20/32 completed all visits within 6M study period.
**SMS**	Sabin 2015	RCT	HIV+ 18+ yrs, in CHN. n=63(Int) n=56(Ctrl)	HIV	SMS reminders via MEM + adherence counselling.	ART in PVLA: MEM.	Increased adherence over 6M (82% vs. 51.8%; RR=1.59(1.21- 2.10), p<0.001) / Effective.
						ART in PVLA: VL.	No difference in undetectable VL (93.6% vs. 98.2%, p=0.218) / No impact.
						ART in PVLA: CD4 count.	Higher mean change in CD4 count (52 vs 28 cell/μL, p=0.297) / No impact.
**PC + MMS.**	Shet 2014	RCT	HIV+ 18-60 yrs, IND. n=315(Int) n=316(Ctrl)	HIV	Weekly automated motivational voice call, followed by weekly MMS.	ART in TNPs: VL.	No difference. (Number of virological failures: 15.6% vs. 15.5%. Time to virological failure: aHR= 0.96(0.65-1.43), p= 0.85) / No impact.
						ART in TNPs: Pill count.	No difference. (27% vs. 21.7%; aIRR=1.24(0.94-1.63), p=0.13) / No impact.
						Feasibility: PC received.	86% of calls received by patients / Highly feasible.
**SMS**	Walsh 2012	UnCtrlled trial.	HIV+ Adults on ART, GBR. n=14	HIV	Pill-box w/ MEM + weekly SMS wrt med taking + up to 3 late dose SMS reminders.	ART in APs: Self-report + MEM.	99.5% baseline adherence, 98% at 24W. No difference in missed doses (4.8% in 0-12W; 6.3% in 13-24W)
						Acceptability: Self-report.	64% satisfied, 50% found SMS & system useful. 55% found reminders irritating / Moderately acceptable.
**SMS**	Lim 2008	Quasi-experimental: HxCtrl.	STI clinic, NZL. n=293(Int) n=303(HxCtrl)	CT	SMS to contact clinic for CT test result.	TAT: Time from test to treat.	No change in median time to treat (3 days vs. 4 days, t = - 1.3, p<0.1) / No impact.
**SMS**	Menon-Johansson 2006	Quasi-experimental.	At clinic w/untreated CT, GBR. n=28(Int) n=21(Ctrl)	CT	SMS to contact clinic for CT test result.	TAT: Time from test to diagnosis & test to treat.	Shorter mean time to diagnosis. (7.9 days vs. 12.5; p<0.001) Shorter median time to treat. (8.5 days vs. 15; p=0.005) / Effective.
**SMS+PC**	Barnabas 2016	RCT	16-49 yrs,, ZAF & UGA. n=284(Int) n=224(Ctrl)	HIV	SMS promoting male circumcision 3W, 6-7W after tested negative. Follow-up phone call 1M & 2M following SMS reminders.	PB: Self-report.	Significant difference in reaching outcome at 3M (Intervention vs clinic referral); 48% (RR=1.72 95% CI 1.36-2.17, p values < 0.0001) in SMS reminder group and 47% (RR=1.67, 95%CI 1.29-2.14, p value = 0.0001) in lay counsellor follow-up achieved MC at 3M / Effective
**SMS + MMS.**	Cornelius 2013	UnCtrlled trial.	African-Americans age 13-18, USA, n=40	HIV	HIV-prevention SMS + knowledge question for	PB: Self-report.	Improved condom attitudes & HIV knowledge (83% vs.78% correct answers) / No impact.

For peer review only

					3W.	Acceptability: Self-report.	97% satisfied w/ number of SMS. 86% reported SMS not interfering w/ daily activities/ Highly acceptable.
						Feasibility: Completion rate.	100% at pretest; 90% at 3M FU/ Highly feasible.
**PC**	DiClemente 2014	RCT	High-risk African-American women 14-20 yrs, USA. n=342(Int) n=359(Ctrl)	CT	PC w/ prevention messages every 8W.	PB: % diagnosed w/ CT or GC.	Fewer participants diagnosed w/ CT & GC (90 vs. 104; RR = 0.5 (0.28-0.88), p=0.02. 48 vs. 54; RR = 0.4 (0.15-1.02), p=0.06) / Effective.
						PB: Self-report.	Higher condom use (MD=0.08(0.06 to 0.10) p=0.04) / Effective.
**SMS + cash incentive**	Juzang 2011	Non-randomized Ctrlled trial.	African-American men 16-20 yrs, USA. n=30/group	HIV	3 weekly SMS HIV prevention messages + $40 for completion.	PB: Self-report.	No statistical difference in % of protected sex. Higher awareness of sexual health / No impact.
						Feasibility: Retention rate.	20 (67%) retained in Ctrl & 19 (63%) in SMS group after 2nd FU / Moderately feasible.
**SMS**	Odeny 2014	RCT	Circumcised male at clinic, 18+ yrs, KE. n=600(Int) n=600(Ctrl)	HIV	Daily SMS for 1wk + SMS on days 8, 14, 21, 28, 35, 41, and 42 post-procedure.	PB: Self-report.	Abstention of sexual activity before 42-day follow up: 139/491(28.3%) vs. 124/493(25.2%) in control group (RR=1.13(0.91-1.38), p=0.3)/ No impact.
**SMS**	Reback 2015	UnCtrlled trial.	MSM drug users 18-65 yrs, USA. n=52	HIV	Daily SMS for 2W to reduce risky sexual behaviours.	PB: Self-report.	Reduction in anal sex (6.9 vs. 2.6, t97=2.82, p<0.05) and unprotected anal sex (1.8 vs. 0.5, t97=2.19, p<0.05) in past 2M/ Effective.
**PC**	Belzer 2015	RCT	HIV+ 12-29 yrs, USA. n=19(Int) n=18(Ctrl)	HIV	PC 1hr from time to take medication.	Acceptability: Self-report.	94% satisfied w/ call length and 81% would continue receiving calls / Highly acceptable.
						Feasibility: Retention rate.	63% retention rate / Moderately feasible.
**SMS**	Dean 2012	Feasibility study.	HIV+ at antenatal clinics, ZAF. n=7	HIV	SMS support group+ inquiries answered by physicians.	Acceptability: Self-report.	Overall satisfaction.
						Feasibility: Self-report.	SMS easily kept confidential.
**SMS**	Roth 2014	Feasibility study.	Sex workers 18+ yrs, USA. n=26	HIV	Cell phone diaries to collect info about sexual events.	Acceptability: Self-report.	Cell-phone electronic dairies to collect sensitive information acceptable (84.6%)/ Highly acceptable.
						Feasibility: Completion rate.	90.3% surveys completed / Highly feasible.
**SMS**	Georgette 2016	Feasibility study.	≥18yrs, HIV+, ZAF. n=88	HIV	Weekly SMS reminders to increase ART adherence and appointment reminders	Acceptability: Self-report.	92% would recommend SMS program to a friend, 90.9% said frequency of SMS was just right, 2/88 felt the SMS program slightly violated their privacy. 97.7% reported it helped them remember to take medication. 77.3% agreed that it helped them remember appointments. / Highly acceptable
**SMS**	Reid 2014	Cross-sectional study.	HIV+, BWA. n=42(Int) n=41(Ctrl)	HIV	SMS ARV pick-up reminder.	Acceptability: Self-report.	SMS helpful 93% (Int) vs. 58% (Ctrl) (p<0.001). SMS may lead to serostatus disclosure 10% vs. 56% (p<0.001). 95% satisfied w/ appointment scheduling. 90% would continue receiving SMS / Highly acceptable.
**PC**	Bauermeister 2014	Feasibility study.	MSM 18-30, USA. n=124	HIV	IVRS: microbicide use.	Feasibility: Self-report.	75.5% reported no problems using IVRS / Highly feasible.
**SMS + MMS.**	Cornelius 2011	Feasibility study.	African-Americans age 13-18, USA. n=12	HIV	HIV-prevention SMS+knowledge question for 3W.	Feasibility: Response rate.	80% response rate/ Highly feasible.

Note: Int= intervention; Ctrl= control; HxCtrl= historical control; PB= preventative behaviors (i.e. risk reduction); PN= partner notification; TAT= turnaround time; ATT= attendance rate; ART= ART adherence; NAPs= non-adherent patients; AP= adherent patients; PVLA= Patients with various levels of adherence; TNPs= Treatment naive patients; VL= viral load; CD4= CD4 cell count; PC= phone call; FB= Facebook.

For peer review only - http://bmjopen.bmj.com/site/about/guidelines.xhtml

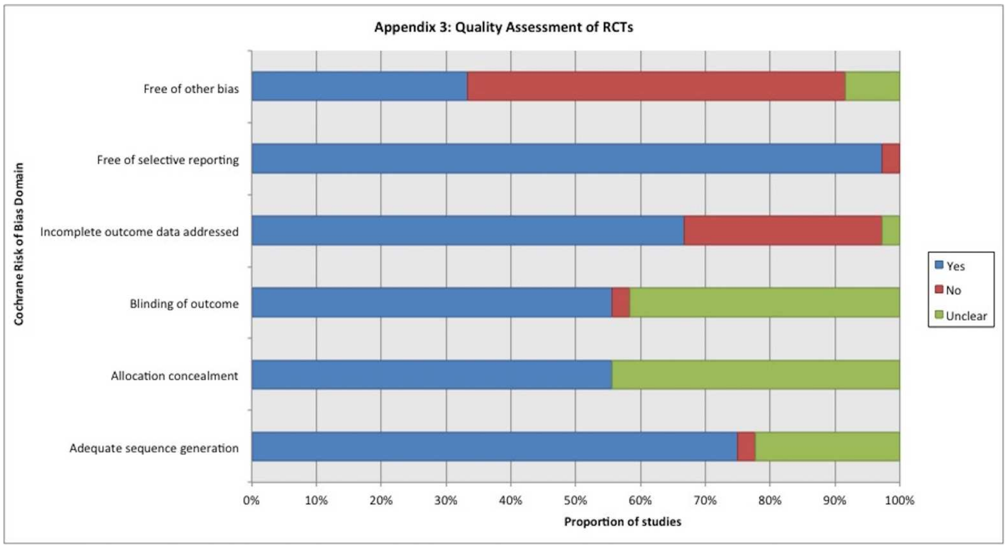

317x171mm (300 x 300 DPI)

review only

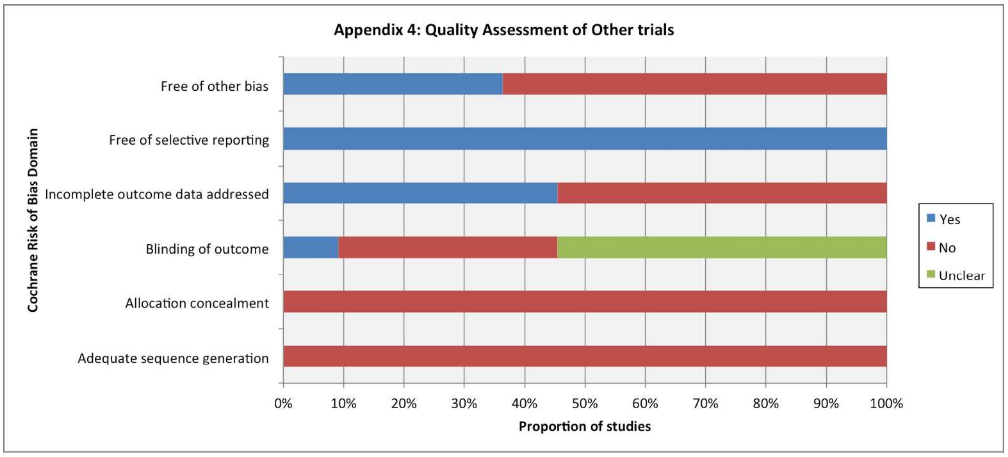

281x126mm (300 x 300 DPI)

For peer review only - http://bmjopen.bmj.com/site/about/guidelines.xhtml

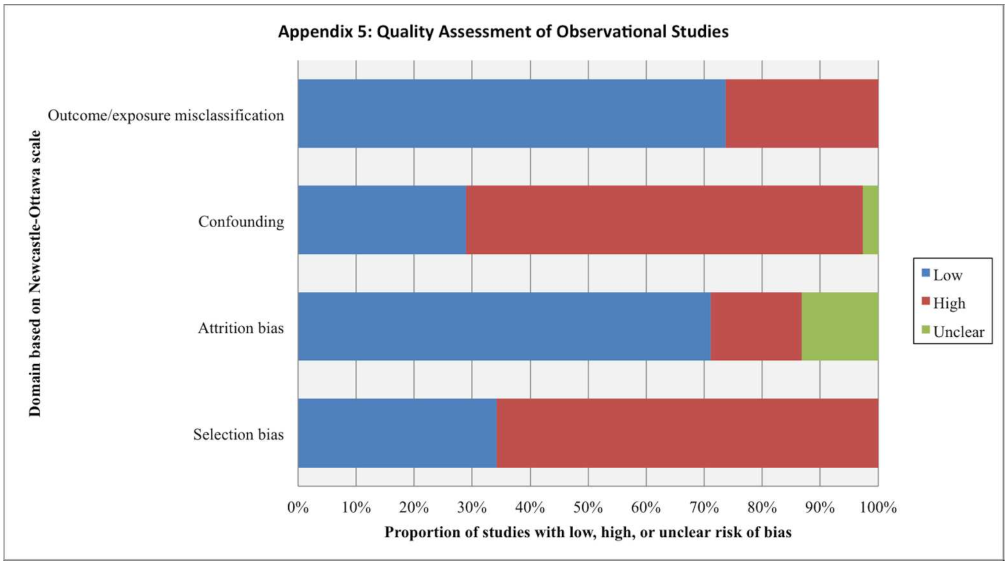

260x145mm (300 x 300 DPI)

review only

PRISMA 2009 Checklist

Section/topic	#	Checklist item	Reported on page #
**TITLE**			
Title	1	Identify the report as a systematic review, meta-analysis, or both.	1
**ABSTRACT**			
Structured summary	2	Provide a structured summary including, as applicable: background; objectives; data sources; study eligibility criteria, participants, and interventions; study appraisal and synthesis methods; results; limitations; conclusions and implications of key findings; systematic review registration number.	2
**INTRODUCTION**			
Rationale	3	Describe the rationale for the review in the context of what is already known.	3
Objectives	4	Provide an explicit statement of questions being addressed with reference to participants, interventions, comparisons, outcomes, and study design (PICOS).	3
**METHODS**			
Protocol and registration	5	Indicate if a review protocol exists, if and where it can be accessed (e.g., Web address), and, if available, provide registration information including registration number.	N/A
Eligibility criteria	6	Specify study characteristics (e.g., PICOS, length of follow-up) and report characteristics (e.g., years considered, language, publication status) used as criteria for eligibility, giving rationale.	4
Information sources	7	Describe all information sources (e.g., databases with dates of coverage, contact with study authors to identify additional studies) in the search and date last searched.	4
Search	8	Present full electronic search strategy for at least one database, including any limits used, such that it could be repeated.	4
Study selection	9	State the process for selecting studies (i.e., screening, eligibility, included in systematic review, and, if applicable, included in the meta-analysis).	4
Data collection process	10	Describe method of data extraction from reports (e.g., piloted forms, independently, in duplicate) and any processes for obtaining and confirming data from investigators.	4
Data items	11	List and define all variables for which data were sought (e.g., PICOS, funding sources) and any assumptions and simplifications made.	4
Risk of bias in individual studies	12	Describe methods used for assessing risk of bias of individual studies (including specification of whether this was done at the study or outcome level), and how this information is to be used in any data synthesis.	5
Summary measures	13	State the principal summary measures (e.g., risk ratio, difference in means).	5
Synthesis of results	14	Describe the methods of handling data and combining results of studies, if done, including measures of consistency (e.g., I^2) for each meta-analysis.	4

For peer review only

PRISMA 2009 Checklist

Section/topic	#	Checklist item	Reported on page #
Risk of bias across studies	15	Specify any assessment of risk of bias that may affect the cumulative evidence (e.g., publication bias, selective reporting within studies).	5
Additional analyses	16	Describe methods of additional analyses (e.g., sensitivity or subgroup analyses, meta-regression), if done, indicating which were pre-specified.	4
**RESULTS**			
Study selection	17	Give numbers of studies screened, assessed for eligibility, and included in the review, with reasons for exclusions at each stage, ideally with a flow diagram.	5
Study characteristics	18	For each study, present characteristics for which data were extracted (e.g., study size, PICOS, follow-up period) and provide the citations.	Abstraction Table
Risk of bias within studies	19	Present data on risk of bias of each study and, if available, any outcome level assessment (see item 12).	8
Results of individual studies	20	For all outcomes considered (benefits or harms), present, for each study: (a) simple summary data for each intervention group (b) effect estimates and confidence intervals, ideally with a forest plot.	6
Synthesis of results	21	Present results of each meta-analysis done, including confidence intervals and measures of consistency.	6
Risk of bias across studies	22	Present results of any assessment of risk of bias across studies (see Item 15).	8, Supplementary
Additional analysis	23	Give results of additional analyses, if done (e.g., sensitivity or subgroup analyses, meta-regression [see Item 16]).	6
**DISCUSSION**			
Summary of evidence	24	Summarize the main findings including the strength of evidence for each main outcome; consider their relevance to key groups (e.g., healthcare providers, users, and policy makers).	8
Limitations	25	Discuss limitations at study and outcome level (e.g., risk of bias), and at review-level (e.g., incomplete retrieval of identified research, reporting bias).	10
Conclusions	26	Provide a general interpretation of the results in the context of other evidence, and implications for future research.	10
**FUNDING**			
Funding	27	Describe sources of funding for the systematic review and other support (e.g., supply of data); role of funders for the systematic review.	2

From: Moher D, Liberati A, Tetzlaff J, Altman DG, The PRISMA Group (2009). Preferred Reporting Items for Systematic Reviews and Meta-Analyses: The PRISMA Statement. PLoS Med 6(7): e1000097. doi:10.1371/journal.pmed1000097

For more information, visit: www.prisma-statement.org.

For peer review only - http://bmjopen.bmj.com/site/about/guidelines.xhtml

PRISMA 2009 Checklist

For peer review only

Do Digital Innovations for HIV and Sexually Transmitted Infections work? Results from a Systematic Review (1996-2017).

Journal:	*BMJ Open*
Manuscript ID	bmjopen-2017-017604.R1
Article Type:	Research
Date Submitted by the Author:	03-Aug-2017
Complete List of Authors:	Daher, Jana; Research Institute of the McGill University Health Centre<br>Vijh, Rohit; Research Institute of the McGill University Health Centre<br>Linthwaite , Blake; Research Institute of the McGill University Health Centre<br>Dave, Sailly; Research Institute of the McGill University Health Centre<br>Kim, John; National HIV/AIDS Labs, Public EHalth Agency of Canda<br>Dheda, Keertan; University of Cape Town<br>Peter, Trevor; Clinton Health Access Initiative (CHAI)<br>Pai, Nitika; McGill University, Medicine
**Primary Subject Heading**:	HIV/AIDS
Secondary Subject Heading:	Health services research, Global health
Keywords:	mHealth/eHealth, Innovations, HIV, Sexually transmitted Infections, systematic reviews

SCHOLARONE™
Manuscripts

For peer review only

Do Digital Innovations for HIV and Sexually Transmitted Infections work? Results from a Systematic Review (1996-2017).

Jana Daher[1], MSc (PH), Rohit Vijh[1], BSc, Blake Linthwaite[1], BSc, Sailly Dave[1], BSc,

John Kim[2], PhD, Keertan Dheda[3], MBBCh, PhD, Trevor Peter[4], PhD,

Nitika Pant Pai[1,5], MD PhD

Affiliations:

[1] Division of Clinical Epidemiology, Research Institute of the McGill University Health Centre, Montreal, Canada.

[2] Chief, National HIV/AIDS Labs, National Labs, Winnipeg, Canada.

[3] Professor, Department of Pulmonology, UCT Lung Institute, University of Cape Town, South Africa.

[4] Scientific Director, Diagnostics, Clinton Health Access Initiative (CHAI), Boston, USA.

[1,5] Associate Professor, Department of Medicine, McGill University, Montreal, Canada.

Corresponding author:

nitika.pai@mcgill.ca

Address: 5252 boul de Maisonneuve West, Division of Clinical Epidemiology, McGill University Health Center, Montreal, Quebec, H4A 3S5

Telephone number: 514-934-1934 x44729

Keywords: mHealth/eHealth; Innovations; HIV; Sexually transmitted Infections; systematic reviews, meta-analyses.

For peer review only

ABSTRACT

Objective: Digital innovations with internet/mobile phones offer a potential cost saving solution for overburdened health systems with high service delivery costs to improve efficiency of HIV/STI control initiatives. However, their overall evidence has not yet been appraised. We evaluated the feasibility and impact of all digital innovations for all HIV/STIs.

Design: Systematic review.

Setting/Participants: All settings/all participants.

Intervention: We classified digital innovations into: a) Mobile health-based (mHealth: SMS (short message service)/phone calls), b) Internet-based mobile and/or electronic health (m/eHealth: social media, avatar-guided computer programs, websites, mobile applications, streamed soap opera videos), and c) combined innovations (included both SMS/phone calls and internet-based m/eHealth).

Primary and secondary outcome measures: Feasibility, Acceptability, Impact.

Methods: We searched Databases- MEDLINE via PubMed, Embase, Cochrane CENTRAL, and Web of Science, abstracted data, explored heterogeneity, performed a random effects subgroup analysis.

Results: We reviewed 99 studies, 63 (64%) were from America/Europe, 36 (36%) from Africa/Asia; 79% (79/99) were clinical trials; 84% (83/99) evaluated impact. Of innovations, mHealth-based: 70% (69/99); internet-based: 21% (21/99); combined: 9% (9/99).
All digital innovations were highly accepted (26/31; 84%), feasible (20/31; 65%). Regarding impacted measures: mHealth-based innovations (SMS) significantly improved ART adherence (pooled OR=2.15 [95%CI: 1·18, 3·91]), and clinic attendance rates (pooled OR=1.76 [95%CI: 1·28, 2·42]); Internet-based innovations improved clinic attendance (6/6), ART adherence (4/4), self-care (1/1), while reducing risk (5/5); combined innovations increased clinic attendance, ART adherence, partner notifications, and self-care. Confounding (68%) and selection bias (66%) were observed in observational studies and attrition bias in 31% of clinical trials.

Conclusion: Digital innovations were acceptable, feasible, and generated impact. A trend towards use of internet-based and combined (internet and mobile) innovations was noted. Large scale up studies of high quality, with new integrated impact metrics, and cost effectiveness are needed. Findings will appeal to all stakeholders in the HIV/STI global initiatives space.

For peer review only - http://bmjopen.bmj.com/site/about/guidelines.xhtml

Strengths of the review

- An updated and comprehensive systematic review/meta-analysis of all innovations in HIV/STI.
- Evaluation of study quality with biases, subgroup analyses and sensitivity analyses.
- Evaluation of metrics and measures for objective and subjective data.

Limitations of the review

- Limited data were reported from Sub-Saharan Africa and Southeast Asia (29%, 29/99).
- Limited evidence (18/99, 18%) was available for STIs (other than HIV).
- Limited data on cost effectiveness from high burden settings.
- A lack of integrated online impact metrics to evaluate internet-based eHealth innovations.

For peer review only

For peer review only - http://bmjopen.bmj.com/site/about/guidelines.xhtml

INTRODUCTION

HIV/STI infections remain a public health concern worldwide - a million new HIV/STI infections are acquired every day, with cumulative disease burden exceeding 500 million infections.[1-5] Regarding HIV, countries are working hard to achieve the new UNAIDS 90-90-90 treatment targets;[6] however, structural and societal barriers such as stigma, low socio-economic status, and geographical isolation, impede access to quality care for marginalized populations who are disproportionately impacted by the HIV/AIDS epidemic.[7-8] Likewise, a lack of timely testing and poor retention in care impairs efforts to control HIV/STIs.[7 9-10] To improve early testing, linkage and retention in care, health care systems globally are seeking solutions to improve population engagement, awareness, and education, and efficient care for their hard-to-reach populations. It is imperative to plug gaps in health care service delivery.[11-12] Digital innovations such as electronic health (eHealth), mobile health (mHealth), and combined innovations offer promising solutions to improve health service delivery. eHealth encompasses non-internet and internet-enabled mHealth as well as other internet-based health interventions. These innovations, together with expanded mobile and internet networks, global connectivity, and affordability, present opportunities to change the future landscape of health care service delivery.

The World Bank estimates that globally, 96% of the world's population and 70% of the world's poorest have access to a mobile phone.[13] Of seven billion, two billion (30%) individuals own a smartphone; approximately 50% of mobile phone users access the internet through their phones.[14-15] Technological access has created a portal for social media and other internet-based health interventions.[16] A rapid diffusion of mobile phones and internet technologies are prime drivers of this disruptive phenomenon in health, aptly titled, the creative destruction of medicine.[17] In recent years, visionary foundations (*Grameen*, *Bill and Melinda Gates Foundation*, *UNAIDS*, *Vodafone)* have, with funding, created opportunities for innovative thinking in health. To date, ninety-five countries have evaluated some digital health innovations.[11] This is most evident in under-resourced settings where low-cost and sustainable solutions are needed to solve complex global health challenges.[18]

Digital innovations were first used in non-communicable diseases and later became popular in infectious disease.[19] In the field of HIV/STIs, a *Lancet* study demonstrated the effectiveness of mHealth-based SMS innovations on adherence to antiretroviral therapy (ART).[20] As novel digital innovations and strategies continue to be developed and tested, many smaller reviews and systematic reviews were published. However, a vast majority of these reviews only evaluated a single innovation (e.g. SMS, social media), one or two outcomes, and restricted exploration in select sub-groups (people living with HIV (PLHIV), pregnant women, adolescents, men who have sex with men (MSM)).[21-27] These reviews failed to provide a comprehensive summary of all innovations for program planning and research. Due to a rapid expansion of digital innovations, and an increased popularity of combined innovations (2013-), a need for a comprehensive up-to-date synthesis on all innovations for HIV/STIs was felt.

Our primary objective was to generate a high quality overview/systematic review that summarizes all digital innovations across all populations and outcomes in HIV/STIs. Our

For peer review only - http://bmjopen.bmj.com/site/about/guidelines.xhtml

secondary objective was to inform researchers, policy makers, funders with evidence for their decisions on implementation and scale-up.[11]

METHODS

PRISMA (Preferred Reporting Items for Systematic Reviews and Meta-Analyses) and Cochrane guidelines were followed.[28]

Data Sources and Searches
We searched MEDLINE via PubMed, Embase, Cochrane CENTRAL, and Web of Science for a 21-year period from Feb 1996 up to March 2017, with no language restrictions.

Search Strategy
Keywords used were HIV, AIDS, STI, mhealth, mobile health, ehealth, telemedicine, mobile applications and social media. For a full search strategy, please refer to Appendix 1. (#1 ("HIV" [MeSH] OR "acquired immunodeficiency syndrome" [tiab]), OR #2 (sexually transmitted infections [mh] OR sexually transmitted disease* [tiab]), AND #3 ("mHealth" [tiab] OR "mobile health" [tiab] OR short messag* [tiab] OR "eHealth" [MeSH] OR "telemedicine" [MeSH] OR social medi* [tiab] OR "mobile applications" [tiab]).

Study Selection
Two reviewers independently screened and evaluated citations for eligibility (JD & RV) and two others (BL & SD) independently assessed quality. A senior reviewer was consulted (NPP) for discordance.

Eligibility Criteria
Any clinical trials or observational study designs that evaluated any digital (m/eHealth) technology with any reported outcomes (Refer Figure 1) were included.

Data Abstraction
Two reviewers (RV, JD) independently abstracted all the data. A pre-piloted data abstraction form, was used to abstract the following items: study design, study population, sample size, digital innovation type, HIV/STIs, outcome measures (e.g. impact, acceptability and feasibility), and metrics (e.g. attendance rate, completion rate, satisfaction) (Refer to Appendix 2). We referred to a previously published framework to define and further classify the following metrics for impact, acceptability, and feasibility.[29]

Subgroup Pooled Analyses
We classified study designs and then classified digital innovations into three groups:[30]

a) mHealth (SMS and phone calls only; i.e. non-internet based);
b) Internet-enabled mHealth and other internet-based eHealth (mobile application, website, online campaign, streamed soap opera videos, avatar-guided computer programs);
c) Combined innovations (innovations that combined both mHealth (SMS/phone calls) with internet enabled m/eHealth).

For peer review only - http://bmjopen.bmj.com/site/about/guidelines.xhtml

Only one subgroup reported similar outcomes which could be pooled, SMS and phone calls, for the following outcomes: a) clinic attendance with SMS; and b) ART adherence via Medication Event Monitoring System (MEMS) caps, with SMS. We pooled these outcomes using a random effects subgroup analysis. Given the diversity in the sample populations between studies, we used the Dersimonian and Laird random effects frequentist model, weighted by study sample to calculate a pooled effect. We generated forest plots for visual representation of heterogeneity and pooled odds ratios (OR) with 95% confidence intervals (CI). We performed all statistical analyses using Stata/IC, version 13 (StataCorp, College Station, Texas USA).[31]

Narrative Analysis

We narratively described all other data using as follows:

Digital innovations were classified into the following groups based on the strength of evidence: high/strong evidence (metrics at 75-100%), moderate evidence (51-74%), and low/weak evidence (50% or less).

Acceptability: Acceptability was defined as the receptivity in using digital innovations.

Feasibility: Feasibility was defined as the perceived convenience in using digital innovations. It was reported with various metrics: completion, retention, response and referral rates.

Impact: Impact was defined as a statistically significant improvement in measured outcomes compared to a comparator group (i.e. control group or baseline observations). The metrics used to evaluated impact were: A) attendance rate, B) ART adherence, C) risk reduction, D) self-care and E) partner notification. Impact measures were evaluated on two criteria: effect size and precision. Effect size was assessed when data on a comparator group was made available. Precision of the effect estimate was assessed whenever reported, as it reflects the variance or spread of results.

Quality Assessment

We assessed study quality for both clinical trials and observational studies. We used the Cochrane Risk of Bias Tool for trials, and Newcastle-Ottawa quality assessment scale for observational studies.

RESULTS

Of 4252 citations identified through our extensive search, 792 were selected for full-text screening, and 99 citations met our inclusion criteria and were included in this review for evidence synthesis (Refer: Figure 1).

Study characteristics

By geographical location, 37% (37/99) of studies were conducted in North America, 26% (26/99) in Sub-Saharan Africa, 24% (24/99) in Europe, 7% (7/99) in Oceania, 3% (3/99) in Southeast Asia, and 2% (2/99) in South America.

By study design, the majority were trials: 38% (38/99) were RCTs, 16% (16/99) uncontrolled trials, and 1% (1/99) non-randomised controlled trials. Others included quasi-experimental

For peer review only - http://bmjopen.bmj.com/site/about/guidelines.xhtml

studies, of which many used historical controls (24%, 24/99), and observational studies (i.e. cross-sectional and feasibility studies) (20%, 20/99).

HIV was the most frequently reported infection (74%, 73/99 studies), followed by chlamydia/gonorrhea (CT/GC) (10%, 10/99). Combinations of HIV with STIs (e.g., syphilis) (8%, 8/99), human papillomavirus (HPV) (4%, 4/99) and hepatitis A/B/C (HBV) (4%, 4/99) were also reported.

In terms of study populations, people living with HIV were prominent across studies (42%, 42/99) followed by other high-risk groups (i.e. MSM/bisexual men, drug users, pregnant women/mother-infant pairs, African-Americans, sex workers, and visible minorities) (28%, 28/99), general clinic attendees (16%, 16/99), CT/ HBV infected individuals (4%, 4/99), and residents of a specific area (9%, 9/99).

Innovations

Digital innovations were documented across the spectrum.

mHealth innovations (SMS/phone calls only) were evaluated in 70% (69/99) of studies.[20 32-99] 72% (50/69) were SMS-based and 28% (19/69) used phone calls or a combination of both (Refer to Figure 2 and Appendix 3).

Internet-enabled mHealth and other internet-based eHealth innovations were evaluated in 21% (21/99) of studies.[100-120] These innovations consisted of many different forms: social media and online campaigns (9/21), avatar-guided computer programs (2/21), mobile applications (5/21), combination of social media and websites (2/21), websites (1/21), telemedicine services (1/21) and streamed soap opera videos (1/21) (Refer to Figure 2 and Appendix 3).

Combined innovations were evaluated in 9% (9/99) of studies.[121-129] Innovations consisted of: SMS + websites/ interactive websites (4/9), SMS + mobile application (3/9) and SMS + social media (including online campaigns) (2/9). (Refer to Figure 2 and Appendix 3).

Measures and Metrics

A vast majority (84%, 83/99) of studies focused on impact measure and metrics, while about 12% (12/99) focused only on feasibility, and the remaining 4% (4/99) on acceptability. Within impact measures, metrics such as clinic attendance rates were reported in 45% (37/83) of studies, followed by ART adherence at 35% (29/83), HIV/STIs risk reduction behaviors at 13% (11/83), turnaround time from testing to treatment at 2% (2/83), partner notification at 2% (2/83), and self-care at 2% (2/83).

Analyses:

Subgroup Pooled Analyses

It was possible to perform subgroup analyses on outcomes that were consistently documented: clinic attendance in 14 quasi-experimental studies that used SMS reminders and MEMS-based ART adherence in 4 RCTs evaluating SMS. The pooled estimate for the impact of SMS reminders on attendance rates was 1.76 [95%CI: 1·28, 2·42] (Refer to Figure 3A). The pooled estimate for the impact of SMS on ART adherence tracked via MEMS caps was also significant, OR= 2.15 [95%CI: 1·18, 3·91] (Refer to Figure 3B).[32,47-48]

For peer review only - http://bmjopen.bmj.com/site/about/guidelines.xhtml

Narrative Analysis

Impact

Non-internet based mHealth (SMS/PC only)

Of 69 studies, positive results were reported for the following outcomes: clinic attendance (63%, 19/30 studies, of which 84% reached statistical significance); ART adherence (63%, 15/24 studies, of which 93% reached statistical significance); turnaround time from testing to treatment (67%, 2/3 studies). However, SMS reported a limited effect on risk reduction behaviors (3/7, 43%).

Internet-based m/eHealth:

Studies evaluating internet-based eHealth innovations (21/99) reported results that were largely in favor of the following innovations: social media-based interventions for clinic attendance; avatar-guided and mobile applications for ART adherence; social media, avatar, and soap opera videos for risk reduction behaviors; mobile app for self-care.

Social media contributed to higher testing uptake rates in all studies (6/6, 100%). A social media-based campaign increased HIV testing by 252% (n= 1500; 19% from baseline 5.4%, p<0·01) and Syphilis testing by 248% (18·8% from baseline 5·4%, p<0·01), while another campaign increased HIV testing by 52% compared to control (n=625; 63.7% vs. 42% in controls, OR=2.9 [95%CI: 1.8-4.7]).[100,115] Four campaigns guaranteed rapid in-home HIV testing for all those who requested it online.[100-101, 108, 111, 116]

Avatar-guided programs and mobile applications improved ART adherence in all studies (4/4). Statistically significant outcomes were reported in 2/4 programs (50%). These were: a) A personalized avatar-guided computer program improved adherence (n=240; p=0·046); b) a mobile application with immunosuppression graphs and medication reminders lowered viral load (n= 28; p=0·023) and improved adherence (p=0·03) as well.[102,104] In the other two studies, an avatar-guided program improved viral suppression and a mobile application allowed for 100% adherence, but these were underpowered to detect a significant effect (n=76 and n=28, respectively).[107,110]

Social media, avatar and soap opera videos were successful at reducing risky sexual behavior in all the reported studies (5/5). However, significant results were reported in only 3/5 studies: a) Social media-based interventions decreased unprotected sex acts by 65% (n=31; 3·11 vs. baseline 8·96, p=0·042); b) soap opera videos on HIV prevention reduced condomless sex by 78% (n=117; 78% reduction from baseline, p<0·001);[103,106]c) An avatar-guided computer program also lowered the odds of HIV transmission (n=240; OR= 0·46, p=0·012).[102-103,106] Even in two underpowered studies, social media-based interventions led to 40% and 67% higher condom uptake (n=70 and n=50, respectively).[105,117]

A mobile application increased self-care in the sole study in this category (1/1). A significantly higher self-care performance among chronic HBV-infected individuals was reported compared to controls (n=53; p=0.001).[112]

For peer review only - http://bmjopen.bmj.com/site/about/guidelines.xhtml

Combined innovations:

Studies evaluating combined innovations (9/99) showed success of social media + SMS in increasing clinic attendance and partner notification; interactive websites + SMS in improving ART adherence; and mobile app + SMS in increasing self-care. Among the five impact studies, 80% reported a favorable outcome. An online campaign with SMS services increased CT, GC, and HIV tests uptake by 41%, 91%, and 190%, respectively;[123] an interactive website with SMS reminders improved ART adherence in drug-users (n=20; p=0·02);[121] a social media-based partner notification with SMS increased notified contacts by 144% (63.5% in 2011 from baseline 26% in 2010);[126] and a mobile app with SMS significantly improved self-care performance in HIV-infected individuals compared to baseline (n=19; p=0.002).[129]

Acceptability and Feasibility

Overall, across studies that assessed acceptability/feasibility, digital innovations were found to be highly acceptable and feasible (75%-100%) in 26/31 and 20/31 studies, respectively. mHealth innovations (SMS/PC only) were highly acceptable and feasible in 81% (13/16) and 75% (12/16) of studies, respectively.

Internet-based m/eHealth innovations were highly acceptable and feasible in 92% (11/12) and 45% (5/11) of studies, respectively. All included innovations (i.e. avatar, mobile app, social media and streamed videos) were highly acceptable.[102-104,-106-107] While avatar-guided programs were rated high on feasibility, social media-based strategies were found to be less feasible[101-103]

Combined innovations were highly acceptable and feasible in 67% (2/3) and 75% (3/4) of studies, respectively.[121,124] The innovations that were rated high involved a combination of SMS and interactive websites.

Quality

Studies were individually evaluated on quality criteria and biases were noted. Across trials, losses to follow-up were reported in 31% of RCTs and 55% of quasi-trials. Additionally, biases (i.e. misclassification, recall bias) were of concern in 58% of the RCTs and 64% of quasi randomized trials (Refer to Appendix 4 & 5).

In observational studies, confounding (68%) and selection bias (66%) were observed. (Refer to Appendix 6). Studies with small sample sizes, low power or insufficient follow-up time (e.g. 3 weeks or less) sometimes provided contradictory results when objective and subjective metrics evaluated the same outcome.

DISCUSSION

Summary of findings

Overall, digital innovations reported positive effects on key metrics. We noted a strong positive effect of digital innovations on clinic attendance rates (70%; 26/37), ART adherence (69%; 20/29), risk reduction behaviors (67%; 8/12) and self-care (100%; 2/2). SMS/phone calls were not able to reduce risky sexual behaviours; however social-media based interventions,

For peer review only - http://bmjopen.bmj.com/site/about/guidelines.xhtml

particularly interactive social media, were effective in reducing risky sexual behaviors. Acceptability was found to be high for all innovations. Feasibility estimates also remained high for all innovations, except for social media-based interventions, possibly due to a perceived lack of privacy and confidentiality. Combined innovations may thus offer promise in plugging this feasibility gap, with internet-based innovations compensating for limitations in SMS-only strategies and vice versa.

While mHealth (SMS/phone calls only) innovations were highly effective in improving clinic attendance, ART adherence, and turnaround time from testing to treatment, they did not report on other outcomes. It should be noted that SMS and phone calls alone failed to reduce risky sexual behaviors, which was not surprising as it is challenging to reduce risky behaviors with a prescriptive SMS alone. Population engagement is essential for risk reduction through qualitative research.

While internet-based m/eHealth innovations (social media, avatar-guided computer programs, mobile apps, and soap opera videos) demonstrated positive evidence on impact metrics, not all studies reached statistical significance. Those that failed to report a statistically significant improvement in ART adherence had small sample sizes and were underpowered to detect these outcomes (n=76 vs. n=240), and had less frequent sessions over a shorter evaluation period (2 sessions over 6 months vs. 4 sessions over 9 months).[102 107] For mobile applications, studies which reported significant effects recruited participants with varying level of adherence,[104 110] compared with studies which had high adherence at baseline (≥ 95%) and did not show significance (due to smaller changes in effect). For social media-based campaigns, the two studies that did not reach statistical significance in reducing risky sexual behaviors lacked an interactive component and simply displayed educational material, while the study that showed significant effect engaged the participants by allowing them to contact professional cognitive behavioral therapists via live chat sessions.[103 105 117]

In terms of quality, confounding and selection bias were noted in observational and quasi-experimental studies, and loss to follow-up in some trials. Nevertheless, the overall validity of the findings from this review was not threatened by biases, as a large proportion of our data were derived from trials. While clinical trials were generally high quality, observational studies were medium to low quality.

Consistent reporting of metrics was lacking, which prevented a comprehensive meta-analysis. Objectives, end points, metrics, and measures, are equally important in feasibility studies and must well designed to generate high quality evidence.

Our review is an exhaustive assessment of the role of digital innovations in improving prevention and care for HIV/STIs. Our findings resonate with many smaller systematic reviews, which have separately evaluated individual components of digital innovation, such as SMS-based mHealth.[22-23 130-137] Other systematic reviews evaluating social media-based interventions reported similar findings to ours, in improved testing uptake or improvements in sexual health.[25-27 138-139]

Our review makes a valuable addition to the growing body of evidence by highlighting the success of other interactive and engaging innovations such as avatar-guided computer programs, mobile apps, streamed soap opera videos, and combined innovations. These integrated

For peer review only - http://bmjopen.bmj.com/site/about/guidelines.xhtml

innovations and programs are gaining in popularity, because of their power to engage rural and urban audiences at many levels.

Designing combined innovations that complementarity of various media, methods, platforms, and messaging may delivery best results. This complementarity may also encourage participant engagement, to improve prevention and care metrics and measures sustainably over time. Engagement is challenging when only one innovation (e.g. mHealth SMS/phone calls only) is the sole focus, where boredom is likely.

Caveats and implications for future research

There are some caveats to considering design and evaluation of innovations. Future research needs to be focussed on tailoring innovations to the context and population, and program objectives. Innovations aiming to reduce risky sexual behaviors could be interactive and tailored to the setting and population, with a deep understanding of patients' needs and preferences.[137 140-141] Any communication with patients could be customized for timing to avoid fatigue with its uptake. For example, patients may be more responsive to weekly versus daily SMS ART reminders.[32 142]

Study quality is essential to generating meaningful results. Large and representative samples of the underlying population and sound statistical techniques during data analysis or sampling methodology, can minimize selection bias. Exploring reasons for differential losses to follow-up could inform future studies. Wherever possible, a control group should be included to differentiate Hawthorne effect from the effect of the intervention.[143] Trials and impact designs can prevent or reduce confounding. Following checklists, like the one by the WHO mHealth Technical Evidence Review Group on mHealth innovations, is suggested and encouraged.[144]

Objective measures (e.g. HIV/STIs diagnosis, VL load) are desired in reporting of quantitative outcomes, over subjective self-reported data (e.g. condom use, self-reported adherence). This could potentially reduce some biases (misclassification biases/ or, desirability/recall biases) that are observed with subjective reporting.

Qualitative data are rich and complement the understanding of all the contextual and population needs, and capture the dynamics of sustainability and change. They need to be integrated with quantitative data to provide a holistic picture of uptake of any digital innovation.

Quality of digital data will merit from an improvement. Across studies, a lack of integrated online impact metrics in evaluating the success of innovations was evident. With continuously evolving digital media, inventing new ways to evaluate acceptability and feasibility becomes necessary. For example, some studies tracked online metrics via Google analytics.[74 100-101 121-124] Synergy with industry powered metrics could be a new wave to measure success of digital innovations.

To scale up proven innovations, a multi-stakeholder engagement is necessary. For that, data and metrics that appeal to all sections of stakeholders will be needed. In addition to improving the quality of randomized controlled trials and quasi-experimental impact studies, qualitative studies, cost effectiveness studies, usability studies, are also needed.

For peer review only - http://bmjopen.bmj.com/site/about/guidelines.xhtml

Implications for policy and practice

In consonance with other systematic reviews, evidence at-scale and over time was scarce.[138] This limits the projection of the long-term sustainability and cost effectiveness of digital innovations. More evidence on scale-up, cost savings and cost-effectiveness from Sub Saharan Africa and Asia is needed. Future investments that incentivize both: the development and evaluation of combined innovations by government and industry alike, and focus on sustainability of digital innovations with public and private partnerships, are urgently needed.

CONCLUSION

To control HIV/STIs globally, we need novel and disruptive innovations that will uniquely impact health outcomes across the spectrum of access, engagement, treatment and retention so as to impact health service delivery. On one hand, mHealth (SMS/phone calls only) and internet-based m/eHealth were found acceptable, feasible and offered complementarity in improving prevention and care of HIV/STIs. On the other hand, when combined, they provided customized and contextualized solutions for hard-to-reach populations.

Innovations need to be proven for impact and cost effectiveness, using a combination of clinical trials, quasi-randomized studies, observational studies, qualitative research studies. Integrating these innovations across various levels of healthcare with clear evaluation, monitoring, and documentation of metrics will facilitate their integration within existing health service delivery models so as to efficiently impact health outcomes over time.

Findings from this comprehensive review will be informative to all stakeholders – innovators, researchers, healthcare practitioners, policy makers and funders – worldwide seeking evidence on integrating and funding innovations, to make the entire spectrum of HIV/STI care.

ACKNOWLEDGEMENT

The authors would like to acknowledge Ms. Megan Smallwood for her assistance in editing the manuscript.

FOOTNOTES

Contributors:

NPP, JD: concept, design.

NPP: data critiquing, write-up, critique, and overall responsibility of the data.

JD: data synthesis, write-up, critiquing.

RV, BL and SD: data synthesis, write-up and critique.

JK, TP and KD: write-up and critique.

Final version approved by all authors

For peer review only

Competing Interests:

There are no conflicts of interest

Data Sharing:

No additional data are available. This is a systematic review/syntheses of existing studies, therefore all data are reported in the tables.

Funding:

Grand Challenges Canada Transition to Scale; Grant number 0710-05.
FRSQ Salary Award Chercheur-Boursier Junior 2.

Figure Legends

Figure 1. PRISMA Flow Diagram

Figure 2. All Innovations by Outcome Type

Figure 3. Sub-Group Analyses

3A. Sub-Group Analysis Pooled OR for Attendance

3B. Sub-Group Analysis Pooled OR for Adherence

Appendices

Appendix 1. Search Strategy

Appendix 2. Abstraction Table

Appendix 3. Table of Studies by Innovation and by Outcomes

Appendix 4. Quality Assessment of RCTs

Appendix 5. Quality Assessment of Other Trials

Appendix 6. Quality Assessment of Observational Studies

For peer review only

REFERENCES

1. The World Health Organization. Sexually transmitted infections (STIs): Fact sheet N°110: WHO Media Centre; 2015 [updated December 2015. Available from: http://www.who.int/mediacentre/factsheets/fs110/en/ accessed 27 January 2016.
2. The World Health Organization. Hepatitis B: Fact sheet N°204: WHO Media Centre; 2015 [updated July 2015. Available from: http://www.who.int/mediacentre/factsheets/fs204/en/ accessed 27 January 2016.
3. The World health Organization. Hepatitis C: Fact sheet N°164 2015 [updated July 2015. Available from: http://www.who.int/mediacentre/factsheets/fs164/en/ accessed 27 January 2016.
4. The Joint United Nations Programme on HIV/AIDS. Fact Sheet 2015 2015 [Available from: http://www.unaids.org/en/resources/campaigns/HowAIDSchangedeverything/factsheet accessed 17 Decembre 2016.
5. Newman L, Rowley J, Vander Hoorn S, et al. Global Estimates of the Prevalence and Incidence of Four Curable Sexually Transmitted Infections in 2012 Based on Systematic Review and Global Reporting. *PLoS One* 2015;10(12):e0143304. doi: 10.1371/journal.pone.0143304

PONE-D-15-31044 [pii] [published Online First: 2015/12/10]

6. The Joint United Nations Programme on HIV/AIDS. The Gap Report. Geneva: UNAIDS, 2014.
7. Kagee A, Remien RH, Berkman A, et al. Structural barriers to ART adherence in Southern Africa: Challenges and potential ways forward. *Glob Public Health* 2011;6(1):83-97. doi: 922541258 [pii]

10.1080/17441691003796387 [published Online First: 2010/05/29]

8. Pai NP. Multiplexed point-of-care assays for HIV and co-infections for resource constrained settings: a perspective. *Future Microbiol* 2015;10(9):1393-6. doi: 10.2217/fmb.15.77 [published Online First: 2015/09/09]
9. Pai NP, Vadnais C, Denkinger C, et al. Point-of-care testing for infectious diseases: diversity, complexity, and barriers in low- and middle-income countries. *PLoS Med* 2012;9(9):e1001306. doi: 10.1371/journal.pmed.1001306

PMEDICINE-D-12-01227 [pii] [published Online First: 2012/09/14]

10. Rosen S, Fox MP. Retention in HIV care between testing and treatment in sub-Saharan Africa: a systematic review. *PLoS Med* 2011;8(7):e1001056. doi: 10.1371/journal.pmed.1001056

PMEDICINE-D-11-00096 [pii] [published Online First: 2011/08/04]

11. Ryu S. Book Review: mHealth: New Horizons for Health through Mobile Technologies: Based on the Findings of the Second Global Survey on eHealth (Global Observatory for eHealth Series, Volume 3). *Healthcare Informatics research* 2012;18(3):231-33. doi: doi:10.4258/hir.2012.18.3.231
12. Free C, Phillips G, Watson L, et al. The effectiveness of mobile-health technologies to improve health care service delivery processes: a systematic review and meta-analysis. *PLoS Med* 2013;10(1):e1001363. doi: 10.1371/journal.pmed.1001363

PMEDICINE-D-12-00641 [pii] [published Online First: 2013/03/06]

13. The World Bank. Maximizing Mobile: Information and Communication Technologies for Development. . Washington DC: International Bank for Reconstruction and Development The World Bank 2012.
14. eMarketer. Smartphone user penetration as percentage of total global population from 2011 to 2018. : Statistica- The Statistics Portal; 2014 [Available from: http://www.statista.com/statistics/203734/global-smartphone-penetration-per-capita-since-2005/ accessed 19 February 2016.

For peer review only - http://bmjopen.bmj.com/site/about/guidelines.xhtml

15. eMarketer. Mobile phone internet user penetration worldwide from 2014 to 2019. : Statistica- The Statistics Potal; 2014 [Available from: http://www.statista.com/statistics/284202/mobile-phone-internet-user-penetration-worldwide/ accessed 19 February 2016.
16. Oulasvirta A, Rattenbury T, Ma L, et al. Habits make smartphone use more pervasive. *Personal and Ubiquitous Computing* 2012;16(1):105-14.
17. Topol EJ. The creative destruction of medicine: How the digital revolution will create better health care. . New York: Basic Books 2012.
18. Akter S, Ray P. mHealth - an Ultimate Platform to Serve the Unserved. *Yearb Med Inform* 2010:94-100. doi: me10010094 [pii] [published Online First: 2010/10/13]
19. Ali EE, Chew L, Yap KYL. Evolution and current status of mhealth research: A systematic review. *BMJ Innov* 2016;2(1):33-40. doi: 10.1136/bmjinnov-2015-000096
20. Lester RT, Ritvo P, Mills EJ, et al. Effects of a mobile phone short message service on antiretroviral treatment adherence in Kenya (WelTel Kenya1): a randomised trial. *Lancet* 2010;376(9755):1838-45. doi: S0140-6736(10)61997-6 [pii]
10.1016/S0140-6736(10)61997-6 [published Online First: 2010/11/13]
21. Ambia J, Mandala J. A systematic review of interventions to improve prevention of mother-to-child HIV transmission service delivery and promote retention. *Journal of the International AIDS Society* 2016;19(1):20309. doi: 10.7448/ias.19.1.20309 [published Online First: 2016/04/09]
22. Conserve DF, Jennings L, Aguiar C, et al. Systematic review of mobile health behavioural interventions to improve uptake of HIV testing for vulnerable and key populations. *J Telemed Telecare* 2017;23(2):347-59. doi: 10.1177/1357633x16639186 [published Online First: 2016/04/09]
23. L'Engle KL, Mangone ER, Parcesepe AM, et al. Mobile Phone Interventions for Adolescent Sexual and Reproductive Health: A Systematic Review. *Pediatrics* 2016;138(3) doi: 10.1542/peds.2016-0884 [published Online First: 2016/08/25]
24. Lima IC, Galvao MT, Alexandre Hde O, et al. Information and communication technologies for adherence to antiretroviral treatment in adults with HIV/AIDS. *Int J Med Inform* 2016;92:54-61. doi: 10.1016/j.ijmedinf.2016.04.013 [published Online First: 2016/06/19]
25. Dunne A, McIntosh J, Mallory D. Adolescents, Sexually Transmitted Infections, and Education Using Social Media: A Review of the Literature. *JNP-J Nurse Pract* 2014;10(6):409-16. doi: 10.1016/j.nurpra.2014.03.020
26. Jones K, Eathington P, Baldwin K, et al. The Impact of Health Education Transmitted Via Social Media or Text Messaging on Adolescent and Young Adult Risky Sexual Behavior: A Systematic Review of the Literature. *Sex Transm Dis* 2014;41(7):413-19. doi: 10.1097/olq.0000000000000146
27. Schnall R, Travers J, Rojas M, et al. eHealth Interventions for HIV Prevention in High-Risk Men Who Have Sex With Men: A Systematic Review. *J Med Internet Res* 2014;16(5):228-37. doi: 10.2196/jmir.3393
28. Moher D, Liberati A, Tetzlaff J, et al. Preferred reporting items for systematic reviews and meta-analyses: the PRISMA statement. *Ann Intern Med* 2009;151(4):264-9, W64. doi: 0000605-200908180-00135 [pii] [published Online First: 2009/07/23]
29. Chiavegatti T, Vijh R, Pant Pai N. Metrics beyond accuracy for HIV point-of-care tests: a systematic review of evidence. *Canadian Journal of Infectious Diseases & Medical Microbiology* 2014;25(Suppl A):90A.
30. Pai M, Filion K. Classification of study designs (Version 8) Montreal: McGill University; 2014 [Available from: http://www.teachepi.org/documents/courses/Classification%20Design.pdf.
31. Harris R, Bradburn M, Deeks J, et al. metan: fixed- and random-effects meta-analysis. *Stata Journal* 2008;8(1):3-28.
32. Pop-Eleches C, Thirumurthy H, Habyarimana JP, et al. Mobile phone technologies improve adherence to antiretroviral treatment in a resource-limited setting: a randomized controlled trial of text

For peer review only - http://bmjopen.bmj.com/site/about/guidelines.xhtml

message reminders. *AIDS* 2011;25(6):825-34. doi: 10.1097/QAD.0b013e32834380c1 [published Online First: 2011/01/22]
33. Dowshen N, Kuhns LM, Johnson A, et al. Improving adherence to antiretroviral therapy for youth living with HIV/AIDS: a pilot study using personalized, interactive, daily text message reminders. *J Med Internet Res* 2012;14(2):e51. doi: v14i2e51 [pii]
10.2196/jmir.2015 [published Online First: 2012/04/07]
34. Menon-Johansson AS, McNaught F, Mandalia S, et al. Texting decreases the time to treatment for genital Chlamydia trachomatis infection. *Sex Transm Infect* 2006;82(1):49-51. doi: 82/1/49 [pii]
10.1136/sti.2004.014381 [published Online First: 2006/02/08]
35. Finocchario-Kessler S, Gautney BJ, Khamadi S, et al. If you text them, they will come: using the HIV infant tracking system to improve early infant diagnosis quality and retention in Kenya. *AIDS* 2014;28 Suppl 3:S313-21. doi: 10.1097/QAD.0000000000000332
00002030-201407001-00011 [pii] [published Online First: 2014/07/06]
36. Lim EJ, Haar J, Morgan J. Can text messaging results reduce time to treatment of Chlamydia trachomatis? *Sex Transm Infect* 2008;84(7):563-4. doi: sti.2008.032441 [pii]
10.1136/sti.2008.032441 [published Online First: 2008/08/30]
37. Bailey S, Scalley B, Gilles M. Text 2 treat - using SMS to recall clients for treatment. *Int J STD AIDS* 2014;25(14):1038-40. doi: 0956462414530108 [pii]
10.1177/0956462414530108 [published Online First: 2014/04/04]
38. Smillie K, Van Borek N, Abaki J, et al. A qualitative study investigating the use of a mobile phone short message service designed to improve HIV adherence and retention in care in Canada (WelTel BC1). *J Assoc Nurses AIDS Care* 2014;25(6):614-25. doi: S1055-3290(14)00040-5 [pii]
10.1016/j.jana.2014.01.008 [published Online First: 2014/04/29]
39. Reback CJ, Fletcher JB, Shoptaw S, et al. Exposure to Theory-Driven Text Messages is Associated with HIV Risk Reduction Among Methamphetamine-Using Men Who have Sex with Men. *AIDS Behav* 2015;19 Suppl 2:130-41. doi: 10.1007/s10461-014-0985-7 [published Online First: 2015/01/08]
40. Evans C, Turner K, Blake H, et al. Text messaging to encourage uptake of HIV testing amongst African communities: Findings from a theory based feasibility study. *HIV Medicine* 2015;16(Suppl 2):54.
41. Dowshen N, Kuhns LM, Gray C, et al. Feasibility of interactive text message response (ITR) as a novel, real-time measure of adherence to antiretroviral therapy for HIV+ youth. *AIDS Behav* 2013;17(6):2237-43. doi: 10.1007/s10461-013-0464-6 [published Online First: 2013/04/03]
42. Cornelius JB, Dmochowski J, Boyer C, et al. Text-messaging-enhanced HIV intervention for African American adolescents: a feasibility study. *J Assoc Nurses AIDS Care* 2013;24(3):256-67. doi: S1055-3290(12)00140-9 [pii]
10.1016/j.jana.2012.06.005 [published Online First: 2012/11/06]
43. Ammassari A, Trotta MP, Mussini C, et al. Mobile phone intervention based on short message system (SMS) improve adherence behavior in HIV-infected non-adherent to antiretroviral therapy. The HI-Vision Study. *Infection* 2010;38(Suppl 52):52.
44. Ammassari A, Trotta MP, Shalev N, et al. Timed short messaging service improves adherence and virological outcomes in HIV-1-infected patients with suboptimal adherence to antiretroviral therapy. *J Acquir Immune Defic Syndr* 2011;58(4):e113-5. doi: 10.1097/QAI.0b013e3182359d2a
00126334-201112010-00015 [pii] [published Online First: 2011/10/29]
45. Segaren N, Lewis T, Desinor O, et al. The distribution and use of cell phones to mothers of HIV-positive infants identified by the Haiti National Early Infant Diagnosis of HIV program (EID): a model for increasing adherence? XIX International AIDS Conference Washington DC, 2012.
46. Sheppard Z, Walsh J, Weston R. The TRxCare™ adherence support system: a pilot study of its acceptability to patients on virologically successful HAART. 18th Annual Conference of British HIV Association Birmingham, 2012.

For peer review only - http://bmjopen.bmj.com/site/about/guidelines.xhtml

47. da Costa TM, Barbosa BJ, Gomes e Costa DA, et al. Results of a randomized controlled trial to assess the effects of a mobile SMS-based intervention on treatment adherence in HIV/AIDS-infected Brazilian women and impressions and satisfaction with respect to incoming messages. *Int J Med Inform* 2012;81(4):257-69. doi: S1386-5056(11)00204-8 [pii]
10.1016/j.ijmedinf.2011.10.002 [published Online First: 2012/02/03]
48. Sabin LL, Bachman DeSilva M, Gill CJ, et al. Improving Adherence to Antiretroviral Therapy With Triggered Real-time Text Message Reminders: The China Adherence Through Technology Study. *J Acquir Immune Defic Syndr* 2015;69(5):551-9. doi: 10.1097/QAI.0000000000000651 [published Online First: 2015/04/19]
49. Brook G, Farmer T, Murphy S, et al. Using SMS text reminders to reduce 'did not attend' (DNA) rates in sexual health and HIV appointment clinics. *Sex Transm Infect* 2013;89(Suppl 1):A1-A428.
50. Kliner M, Knight A, Mamvura C, et al. Using no-cost mobile phone reminders to improve attendance for HIV test results: a pilot study in rural Swaziland. *Infect Dis Poverty* 2013;2(1):12. doi: 2049-9957-2-12 [pii]
10.1186/2049-9957-2-12 [published Online First: 2013/07/16]
51. Farmer T, Brook G, McSorley J, et al. Using short message service text reminders to reduce 'did not attend' rates in sexual health and HIV appointment clinics. *Int J STD AIDS* 2014;25(4):289-93. doi: 0956462413502325 [pii]
10.1177/0956462413502325 [published Online First: 2013/09/04]
52. Brook G, Burton J, McSorley J, et al. The effectiveness of SMS texts for reminding patietns at high risk of STIs and HIV to return for testing. *Sex Transm Infect* 2013;89(Suppl 1):A1-A428.
53. Guy R, Wand H, Knight V, et al. SMS reminders improve re-screening in women and heterosexual men with chlamydia infection at Sydney Sexual Health Centre: a before-and-after study. *Sex Transm Infect* 2013;89(1):11-5. doi: sextrans-2011-050370 [pii]
10.1136/sextrans-2011-050370 [published Online First: 2012/04/21]
54. Burton J, Brook G, McSorley J, et al. The utility of short message service (SMS) texts to remind patients at higher risk of STIs and HIV to reattend for testing: a controlled before and after study. *Sex Transm Infect* 2014;90(1):11-3. doi: sextrans-2013-051228 [pii]
10.1136/sextrans-2013-051228 [published Online First: 2013/09/26]
55. Desai M, Burns F, Mercey D, et al. Active recall of men who have sex with men (MSM) for an HIV/STI testing: a feasible and effective strategy? *HIV Medicine* 2014;15(Suppl 3):17-159.
56. Kharbanda EO, Stockwell MS, Fox HW, et al. Text message reminders to promote human papillomavirus vaccination. *Vaccine* 2011;29(14):2537-41. doi: S0264-410X(11)00125-3 [pii]
10.1016/j.vaccine.2011.01.065 [published Online First: 2011/02/09]
57. Vilella A, Bayas JM, Diaz MT, et al. The role of mobile phones in improving vaccination rates in travelers. *Prev Med* 2004;38(4):503-9. doi: 10.1016/j.ypmed.2003.12.005
S0091743503003475 [pii] [published Online First: 2004/03/17]
58. Downing SG, Cashman C, McNamee H, et al. Increasing chlamydia test of re-infection rates using SMS reminders and incentives. *Sex Transm Infect* 2013;89(1):16-9. doi: sextrans-2011-050454 [pii]
10.1136/sextrans-2011-050454 [published Online First: 2012/06/26]
59. Odeny TA, Bailey RC, Bukusi EA, et al. Text messaging to improve attendance at post-operative clinic visits after adult male circumcision for HIV prevention: a randomized controlled trial. *PLoS One* 2012;7(9):e43832. doi: 10.1371/journal.pone.0043832
PONE-D-12-11992 [pii] [published Online First: 2012/09/08]
60. de Tolly K, Skinner D, Nembaware V, et al. Investigation into the use of short message services to expand uptake of human immunodeficiency virus testing, and whether content and dosage have impact. *Telemed J E Health* 2012;18(1):18-23. doi: 10.1089/tmj.2011.0058 [published Online First: 2011/12/14]

For peer review only - http://bmjopen.bmj.com/site/about/guidelines.xhtml

61. Bigna JJ, Noubiap JJ, Kouanfack C, et al. Effect of mobile phone reminders on follow-up medical care of children exposed to or infected with HIV in Cameroon (MORE CARE): a multicentre, single-blind, factorial, randomised controlled trial. *Lancet Infect Dis* 2014;14(7):600-8. doi: S1473-3099(14)70741-8 [pii]
10.1016/S1473-3099(14)70741-8 [published Online First: 2014/06/17]
62. Rand CM, Brill H, Albertin C, et al. Effectiveness of centralized text message reminders on human papillomavirus immunization coverage for publicly insured adolescents. *J Adolesc Health* 2015;56(5 Suppl):S17-20. doi: S1054-139X(14)00707-1 [pii]
10.1016/j.jadohealth.2014.10.273 [published Online First: 2015/04/13]
63. Matheson EC, Derouin A, Gagliano M, et al. Increasing HPV vaccination series completion rates via text message reminders. *J Pediatr Health Care* 2014;28(4):e35-9. doi: S0891-5245(13)00293-9 [pii]
10.1016/j.pedhc.2013.09.001 [published Online First: 2013/11/10]
64. Schwartz SR, Clouse K, Yende N, et al. Acceptability and Feasibility of a Mobile Phone-Based Case Management Intervention to Retain Mothers and Infants from an Option B+ Program in Postpartum HIV Care. *Matern Child Health J* 2015;19(9):2029-37. doi: 10.1007/s10995-015-1715-0 [published Online First: 2015/02/07]
65. Belzer ME, Naar-King S, Olson J, et al. The use of cell phone support for non-adherent HIV-infected youth and young adults: an initial randomized and controlled intervention trial. *AIDS Behav* 2014;18(4):686-96. doi: 10.1007/s10461-013-0661-3 [published Online First: 2013/11/26]
66. Maduka O, Tobin-West CI. Adherence counseling and reminder text messages improve uptake of antiretroviral therapy in a tertiary hospital in Nigeria. *Niger J Clin Pract* 2013;16(3):302-8. doi: NigerJClinPract_2013_16_3_302_113451 [pii]
10.4103/1119-3077.113451 [published Online First: 2013/06/19]
67. Kebaya L, Nduati R, Bashir A. Efficacy of mobile phone use on adherence to nevirapine prophylaxis and retention in care among the HIV-exposed infants in PMTCT: a randomized controlled trial. *Arch Dis Child* 2014;99(Suppl 2):A1-A620.
68. Hardy H, Kumar V, Doros G, et al. Randomized controlled trial of a personalized cellular phone reminder system to enhance adherence to antiretroviral therapy. *AIDS Patient Care STDS* 2011;25(3):153-61. doi: 10.1089/apc.2010.0006 [published Online First: 2011/02/18]
69. DiClemente RJ, Wingood GM, Sales JM, et al. Efficacy of a telephone-delivered sexually transmitted infection/human immunodeficiency virus prevention maintenance intervention for adolescents: a randomized clinical trial. *JAMA Pediatr* 2014;168(10):938-46. doi: 1899236 [pii]
10.1001/jamapediatrics.2014.1436 [published Online First: 2014/08/27]
70. Cornelius JB, Cato M, Lawrence JS, et al. Development and pretesting multimedia HIV-prevention text messages for mobile cell phone delivery. *J Assoc Nurses AIDS Care* 2011;22(5):407-13. doi: S1055-3290(10)00224-4 [pii]
10.1016/j.jana.2010.11.007 [published Online First: 2011/01/25]
71. Dean AL, Makin JD, Kydd AS, et al. A pilot study using interactive SMS support groups to prevent mother-to-child HIV transmission in South Africa. *J Telemed Telecare* 2012;18(7):399-403. doi: jtt.2012.120118 [pii]
10.1258/jtt.2012.120118 [published Online First: 2012/10/05]
72. Juzang I, Fortune T, Black S, et al. A pilot programme using mobile phones for HIV prevention. *J Telemed Telecare* 2011;17(3):150-3. doi: jtt.2010.091107 [pii]
10.1258/jtt.2010.091107 [published Online First: 2011/01/29]
73. Kalichman SC, Kalichman MO, Cherry C, et al. Brief behavioral self-regulation counseling for HIV treatment adherence delivered by cell phone: an initial test of concept trial. *AIDS Patient Care STDS* 2011;25(5):303-10. doi: 10.1089/apc.2010.0367 [published Online First: 2011/04/05]

For peer review only - http://bmjopen.bmj.com/site/about/guidelines.xhtml

74. Mbuagbaw L, Thabane L, Ongolo-Zogo P, et al. The Cameroon Mobile Phone SMS (CAMPS) trial: a randomized trial of text messaging versus usual care for adherence to antiretroviral therapy. *PLoS One* 2012;7(12):e46909. doi: 10.1371/journal.pone.0046909
PONE-D-12-24092 [pii] [published Online First: 2012/12/14]
75. Moore DJ, Poquette A, Casaletto KB, et al. Individualized texting for adherence building (iTAB): improving antiretroviral dose timing among HIV-infected persons with co-occurring bipolar disorder. *AIDS Behav* 2015;19(3):459-71. doi: 10.1007/s10461-014-0971-0 [published Online First: 2014/12/17]
76. Norton BL, Person AK, Castillo C, et al. Barriers to using text message appointment reminders in an HIV clinic. *Telemed J E Health* 2014;20(1):86-9. doi: 10.1089/tmj.2012.0275 [published Online First: 2013/10/29]
77. Odeny TA, Bailey RC, Bukusi EA, et al. Effect of text messaging to deter early resumption of sexual activity after male circumcision for HIV prevention: a randomized controlled trial. *J Acquir Immune Defic Syndr* 2014;65(2):e50-7. doi: 10.1097/QAI.0b013e3182a0a050 [published Online First: 2013/07/13]
78. Bauermeister J, Carballo-Dieguez A, Giguere R, et al. Interactive voice response system (IVRS): data quality considerations and lessons learned during a microbicide placebo adherence trial with young men who have sex with men. *Journal of Adolescent Health* 2014;54(2):S57-S58.
79. Belzer ME, Kolmodin MacDonell K, Clark LF, et al. Acceptability and Feasibility of a Cell Phone Support Intervention for Youth Living with HIV with Nonadherence to Antiretroviral Therapy. *AIDS Patient Care STDS* 2015;29(6):338-45. doi: 10.1089/apc.2014.0282 [published Online First: 2015/05/01]
80. Shet A, De Costa A, Kumarasamy N, et al. Effect of mobile telephone reminders on treatment outcome in HIV: evidence from a randomised controlled trial in India. *BMJ* 2014;349:g5978. [published Online First: 2014/01/01]
81. Reid MJ, Dhar SI, Cary M, et al. Opinions and attitudes of participants in a randomized controlled trial examining the efficacy of SMS reminders to enhance antiretroviral adherence: a cross-sectional survey. *J Acquir Immune Defic Syndr* 2014;65(2):e86-8. doi: 10.1097/QAI.0b013e3182a9c72b
00126334-201402010-00027 [pii] [published Online First: 2014/01/21]
82. Roth AM, Hensel DJ, Fortenberry JD, et al. Feasibility and acceptability of cell phone diaries to measure HIV risk behavior among female sex workers. *AIDS Behav* 2014;18(12):2314-24. doi: 10.1007/s10461-014-0718-y [published Online First: 2014/03/20]
83. Barnabas RV, van Rooyen H, Tumwesigye E, et al. Uptake of antiretroviral therapy and male circumcision after community-based HIV testing and strategies for linkage to care versus standard clinic referral: a multisite, open-label, randomised controlled trial in South Africa and Uganda. *The lancet HIV* 2016;3(5):e212-20. doi: 10.1016/s2352-3018(16)00020-5 [published Online First: 2016/04/30]
84. Bassett IV, Coleman SM, Giddy J, et al. Sizanani: A Randomized Trial of Health System Navigators to Improve Linkage to HIV and TB Care in South Africa. *J Acquir Immune Defic Syndr* 2016;73(2):154-60. doi: 10.1097/qai.0000000000001025 [published Online First: 2016/09/16]
85. Cantudo-Cuenca MR, Manzano-Garcia M, Jimenez-Galan R, et al. Effect of mobile phone short messages on antiretroviral treatment adherence among HIV infected patients. *International Journal of Clinical Pharmacy* 2016;38 (6):517. doi: http://dx.doi.org/10.1007/s11096-015-0240-y
86. Haberer JE, Musiimenta A, Atukunda EC, et al. Short message service (SMS) reminders and real-time adherence monitoring improve antiretroviral therapy adherence in rural Uganda. *AIDS* 2016;30(8):1295-300. doi: 10.1097/qad.0000000000001021 [published Online First: 2016/01/14]
87. Jeffries C, Ross P, Matoff-Stepp S, et al. Ucare4life: Mobile texting to improve HIV care continuum outcomes for minority youth. *Topics in Antiviral Medicine* 2016;24 (E-1):427.

For peer review only - http://bmjopen.bmj.com/site/about/guidelines.xhtml

88. Joseph Davey D, Nhavoto JA, Augusto O, et al. SMSaude: Evaluating Mobile Phone Text Reminders to Improve Retention in HIV Care for Patients on Antiretroviral Therapy in Mozambique. *J Acquir Immune Defic Syndr* 2016;73(2):e23-30. doi: 10.1097/qai.0000000000001115 [published Online First: 2016/09/16]
89. Kampman C, Koedijk F, Driessen-Hulshof H, et al. Retesting young STI clinic visitors with urogenital Chlamydia trachomatis infection in the Netherlands; response to a text message reminder and reinfection rates: a prospective study with historical controls. *Sex Transm Infect* 2016;92(2):124-9. doi: 10.1136/sextrans-2015-052115 [published Online First: 2015/09/26]
90. Kassaye SG, Ong'ech J, Sirengo M, et al. Cluster-Randomized Controlled Study of SMS Text Messages for Prevention of Mother-to-Child Transmission of HIV in Rural Kenya. *AIDS research and treatment* 2016;2016:1289328. doi: 10.1155/2016/1289328 [published Online First: 2017/01/06]
91. McIver R, Dyda A, McNulty AM, et al. Text message reminders do not improve hepatitis B vaccination rates in an Australian sexual health setting. *Journal of the American Medical Informatics Association : JAMIA* 2016;23(e1):e88-92. doi: 10.1093/jamia/ocv145 [published Online First: 2015/10/27]
92. Njuguna N, Ngure K, Mugo N, et al. The Effect of Human Immunodeficiency Virus Prevention and Reproductive Health Text Messages on Human Immunodeficiency Virus Testing Among Young Women in Rural Kenya: A Pilot Study. *Sex Transm Dis* 2016;43(6):353-9. doi: 10.1097/olq.0000000000000450 [published Online First: 2016/05/21]
93. Nsagha DS, Lange I, Fon PN, et al. A Randomized Controlled Trial on the Usefulness of Mobile Text Phone Messages to Improve the Quality of Care of HIV and AIDS Patients in Cameroon. *Open AIDS J* 2016;10:93-103. doi: 10.2174/1874613601610010093 [published Online First: 2016/09/02]
94. Nyatsanza F, McSorley J, Murphy S, et al. 'It's all in the message': the utility of personalised short message service (SMS) texts to remind patients at higher risk of STIs and HIV to reattend for testing-a repeat before and after study. *Sex Transm Infect* 2016;92(5):393-5. doi: 10.1136/sextrans-2015-052216 [published Online First: 2015/12/17]
95. Rana AI, van den Berg JJ, Lamy E, et al. Using a Mobile Health Intervention to Support HIV Treatment Adherence and Retention Among Patients at Risk for Disengaging with Care. *AIDS Patient Care STDS* 2016;30(4):178-84. doi: 10.1089/apc.2016.0025 [published Online First: 2016/03/31]
96. Rand CM, Vincelli P, Goldstein NP, et al. Effects of Phone and Text Message Reminders on Completion of the Human Papillomavirus Vaccine Series. *J Adolesc Health* 2017;60(1):113-19. doi: 10.1016/j.jadohealth.2016.09.011 [published Online First: 2016/11/12]
97. Coleman J, Bohlin KC, Thorson A, et al. Effectiveness of an SMS-based maternal mHealth intervention to improve clinical outcomes of HIV-positive pregnant women. *AIDS care* 2017:1-8. doi: 10.1080/09540121.2017.1280126 [published Online First: 2017/01/22]
98. Garofalo R, Kuhns LM, Hotton A, et al. A Randomized Controlled Trial of Personalized Text Message Reminders to Promote Medication Adherence Among HIV-Positive Adolescents and Young Adults. *AIDS Behav* 2016;20(5):1049-59. doi: 10.1007/s10461-015-1192-x [published Online First: 2015/09/13]
99. Georgette N, Siedner MJ, Zanoni B, et al. The Acceptability and Perceived Usefulness of a Weekly Clinical SMS Program to Promote HIV Antiretroviral Medication Adherence in KwaZulu-Natal, South Africa. *AIDS Behav* 2016;20(11):2629-38. doi: 10.1007/s10461-016-1287-z [published Online First: 2016/10/21]
100. Dowshen N, Lee S, Matty Lehman B, et al. IknowUshould2: Feasibility of a Youth-Driven Social Media Campaign to Promote STI and HIV Testing Among Adolescents in Philadelphia. *AIDS Behav* 2015;19 Suppl 2:106-11. doi: 10.1007/s10461-014-0991-9 [published Online First: 2015/01/08]

For peer review only - http://bmjopen.bmj.com/site/about/guidelines.xhtml

101. Jones M, Aderogba K, Drunis L. Providing rapid HIV testing in their homes for men who have sex with men, recruited via social media. *HIV Medicine* 2015;16(Suppl 2):50-51.
102. Kurth AE, Spielberg F, Cleland CM, et al. Computerized counseling reduces HIV-1 viral load and sexual transmission risk: findings from a randomized controlled trial. *J Acquir Immune Defic Syndr* 2014;65(5):611-20. doi: 10.1097/QAI.0000000000000100 [published Online First: 2014/01/05]
103. Lelutiu-Weinberger C, Pachankis JE, Gamarel KE, et al. Feasibility, Acceptability, and Preliminary Efficacy of a Live-Chat Social Media Intervention to Reduce HIV Risk Among Young Men Who Have Sex With Men. *AIDS Behav* 2015;19(7):1214-27. doi: 10.1007/s10461-014-0911-z [published Online First: 2014/09/27]
104. Perera AI, Thomas MG, Moore JO, et al. Effect of a smartphone application incorporating personalized health-related imagery on adherence to antiretroviral therapy: a randomized clinical trial. *AIDS Patient Care STDS* 2014;28(11):579-86. doi: 10.1089/apc.2014.0156 [published Online First: 2014/10/08]
105. Jones K, Baldwin KA, Lewis PR. The potential influence of a social media intervention on risky sexual behavior and Chlamydia incidence. *J Community Health Nurs* 2012;29(2):106-20. doi: 10.1080/07370016.2012.670579 [published Online First: 2012/04/28]
106. Jones R, Hoover DR, Lacroix LJ. A randomized controlled trial of soap opera videos streamed to smartphones to reduce risk of sexually transmitted human immunodeficiency virus (HIV) in young urban African American women. *Nurs Outlook* 2013;61(4):205-15 e3. doi: S0029-6554(13)00061-4 [pii]
10.1016/j.outlook.2013.03.006 [published Online First: 2013/06/08]
107. Naar-King S, Outlaw AY, Sarr M, et al. Motivational Enhancement System for Adherence (MESA): pilot randomized trial of a brief computer-delivered prevention intervention for youth initiating antiretroviral treatment. *J Pediatr Psychol* 2013;38(6):638-48. doi: jss132 [pii]
10.1093/jpepsy/jss132 [published Online First: 2013/01/30]
108. Elliot E, Rossi M, McCormack S, et al. Identifying undiagnosed HIV in men who have sex with men (MSM) by offering HIV home sampling via online gay social media: a service evaluation. *Sex Transm Infect* 2016;92(6):470-3. doi: 10.1136/sextrans-2015-052090 [published Online First: 2016/05/26]
109. Garett R, Menacho L, Young SD. Ethical Issues in Using Social Media to Deliver an HIV Prevention Intervention: Results from the HOPE Peru Study. *Prevention science : the official journal of the Society for Prevention Research* 2017;18(2):225-32. doi: 10.1007/s11121-016-0739-z [published Online First: 2016/12/10]
110. Himelhoch S, Kreyenbuhl J, Palmer-Bacon J, et al. Pilot feasibility study of Heart2HAART: a smartphone application to assist with adherence among substance users living with HIV. *AIDS care* 2017:1-7. doi: 10.1080/09540121.2016.1259454 [published Online First: 2017/01/04]
111. Huang E, Marlin RW, Young SD, et al. Using Grindr, a Smartphone Social-Networking Application, to Increase HIV Self-Testing Among Black and Latino Men Who Have Sex With Men in Los Angeles, 2014. *AIDS education and prevention : official publication of the International Society for AIDS Education* 2016;28(4):341-50. doi: 10.1521/aeap.2016.28.4.341 [published Online First: 2016/07/20]
112. Jeon JH. Evaluation of a smartphone application for self-care performance of patients with chronic hepatitis B: A randomized controlled trial. *Applied nursing research : ANR* 2016;32:182-89. doi: 10.1016/j.apnr.2016.07.011 [published Online First: 2016/12/15]
113. Polilli E, Sozio F, Di Stefano P, et al. Web-Based HIV Testing in Abruzzo, Italy: Analysis of 15-Month Activity Results. *AIDS Patient Care STDS* 2016;30(10):471-75. doi: 10.1089/apc.2016.0082
114. Przybyla SM, Eliseo-Arras RK, Krawiec G, et al. Feasibility and Acceptability of a Smartphone App for Daily Reports of Substance Use and Antiretroviral Therapy Adherence among HIV-Infected

For peer review only - http://bmjopen.bmj.com/site/about/guidelines.xhtml

Adults. *AIDS research and treatment* 2016;2016:9510172. doi: 10.1155/2016/9510172 [published Online First: 2016/09/10]
115. Rhodes SD, McCoy TP, Tanner AE, et al. Using Social Media to Increase HIV Testing Among Gay and Bisexual Men, Other Men Who Have Sex With Men, and Transgender Persons: Outcomes From a Randomized Community Trial. *Clinical infectious diseases : an official publication of the Infectious Diseases Society of America* 2016;62(11):1450-3. doi: 10.1093/cid/ciw127 [published Online First: 2016/03/17]
116. Rosengren AL, Huang E, Daniels J, et al. Feasibility of using GrindrTM to distribute HIV self-test kits to men who have sex with men in Los Angeles, California. *Sexual health* 2016 doi: 10.1071/sh15236 [published Online First: 2016/05/23]
117. Solorio R, Norton-Shelpuk P, Forehand M, et al. Tu Amigo Pepe: Evaluation of a Multi-media Marketing Campaign that Targets Young Latino Immigrant MSM with HIV Testing Messages. *AIDS Behav* 2016;20(9):1973-88. doi: 10.1007/s10461-015-1277-6 [published Online First: 2016/02/07]
118. Talal AH, Andrews P, McLeod A, et al. Telemedicine-based Hepatitis C Virus (HCV) management for individuals on opioid agonist treatment (OAT). *Hepatology* 2016;63 (1 Supplement 1):475A.
119. Henwood R, Patten G, Barnett W, et al. Acceptability and use of a virtual support group for HIV-positive youth in Khayelitsha, Cape Town using the MXit social networking platform. *AIDS care* 2016;28(7):898-903. doi: 10.1080/09540121.2016.1173638 [published Online First: 2016/04/22]
120. Brayboy LM, Sepolen A, Mezoian T, et al. Girl Talk: A Smartphone Application to Teach Sexual Health Education to Adolescent Girls. *J Pediatr Adolesc Gynecol* 2017;30(1):23-28. doi: 10.1016/j.jpag.2016.06.011 [published Online First: 2016/07/10]
121. Horvath KJ, Oakes JM, Rosser BR, et al. Feasibility, acceptability and preliminary efficacy of an online peer-to-peer social support ART adherence intervention. *AIDS Behav* 2013;17(6):2031-44. doi: 10.1007/s10461-013-0469-1 [published Online First: 2013/04/05]
122. Gotz HM, van Rooijen MS, Vriens P, et al. Initial evaluation of use of an online partner notification tool for STI, called 'suggest a test': a cross sectional pilot study. *Sex Transm Infect* 2014;90(3):195-200. doi: sextrans-2013-051254 [pii]
10.1136/sextrans-2013-051254 [published Online First: 2014/01/07]
123. Friedman AL, Brookmeyer KA, Kachur RE, et al. An assessment of the GYT: Get Yourself Tested campaign: an integrated approach to sexually transmitted disease prevention communication. *Sex Transm Dis* 2014;41(3):151-7. doi: 10.1097/OLQ.0000000000000100
00007435-201403000-00001 [pii] [published Online First: 2014/02/14]
124. Hightow-Weidman LB, Muessig KE, Pike EC, et al. HealthMpowerment.org: Building Community Through a Mobile-Optimized, Online Health Promotion Intervention. *Health Educ Behav* 2015;42(4):493-9. doi: 1090198114562043 [pii]
10.1177/1090198114562043 [published Online First: 2015/01/16]
125. Dokkum NF, Koekenbier RH, van den Broek IV, et al. Keeping participants on board: increasing uptake by automated respondent reminders in an Internet-based chlamydia screening in the Netherlands. *BMC Public Health* 2012;12:176. doi: 1471-2458-12-176 [pii]
10.1186/1471-2458-12-176 [published Online First: 2012/03/13]
126. Hightow-Weidman L, Beagle S, Pike E, et al. "No one's at home and they won't pick up the phone": using the Internet and text messaging to enhance partner services in North Carolina. *Sex Transm Dis* 2014;41(2):143-8. doi: 10.1097/OLQ.0000000000000087
00007435-201402000-00014 [pii] [published Online First: 2014/01/15]
127. Aronson ID, Cleland CM, Perlman DC, et al. MOBILE SCREENING TO IDENTIFY AND FOLLOW-UP WITH HIGH RISK, HIV NEGATIVE YOUTH. *Journal of mobile technology in medicine* 2016;5(1):9-18. doi: 10.7309/jmtm.5.1.3 [published Online First: 2016/04/26]
128. Hirsch-Moverman Y, Daftary A, Yuengling KA, et al. Using mHealth for HIV/TB Treatment Support in Lesotho: Enhancing Patient-Provider Communication in the START Study. *J Acquir*

For peer review only - http://bmjopen.bmj.com/site/about/guidelines.xhtml

Immune Defic Syndr 2017;74 Suppl 1:S37-S43. doi: 10.1097/qai.0000000000001202 [published Online First: 2016/12/09]
129. John ME, Samson-Akpan PE, Etowa JB, et al. Enhancing self-care, adjustment and engagement through mobile phones in youth with HIV. *International nursing review* 2016;63(4):555-61. doi: 10.1111/inr.12313 [published Online First: 2016/09/23]
130. Guy R, Hocking J, Wand H, et al. How effective are short message service reminders at increasing clinic attendance? A meta-analysis and systematic review. *Health Serv Res* 2012;47(2):614-32. doi: 10.1111/j.1475-6773.2011.01342.x [published Online First: 2011/11/19]
131. Free C, Phillips G, Galli L, et al. The effectiveness of mobile-health technology-based health behaviour change or disease management interventions for health care consumers: a systematic review. *PLoS Med* 2013;10(1):e1001362. doi: 10.1371/journal.pmed.1001362
PMEDICINE-D-12-00520 [pii] [published Online First: 2013/01/26]
132. Ershad Sarabi R, Sadoughi F, Jamshidi Orak R, et al. The Effectiveness of Mobile Phone Text Messaging in Improving Medication Adherence for Patients with Chronic Diseases: A Systematic Review. *Iranian Red Crescent medical journal* 2016;18(5):e25183. doi: 10.5812/ircmj.25183 [published Online First: 2016/07/21]
133. Finitsis DJ, Pellowski JA, Huedo-Medina TB, et al. Visual analogue scale (VAS) measurement of antiretroviral adherence in people living with HIV (PLWH): a meta-analysis. *Journal of behavioral medicine* 2016;39(6):1043-55. doi: 10.1007/s10865-016-9770-6 [published Online First: 2016/08/03]
134. Kanters S, Park JJ, Chan K, et al. Interventions to improve adherence to antiretroviral therapy: a systematic review and network meta-analysis. *The lancet HIV* 2017;4(1):e31-e40. doi: 10.1016/s2352-3018(16)30206-5 [published Online First: 2016/11/20]
135. Mayer JE, Fontelo P. Meta-analysis on the effect of text message reminders for HIV-related compliance. *AIDS care* 2016:1-9. doi: 10.1080/09540121.2016.1214674 [published Online First: 2016/08/02]
136. Thakkar J, Kurup R, Laba TL, et al. Mobile telephone text messaging for medication adherence in chronic disease a meta-analysis. *JAMA Intern Med* 2016;176(3):340-49. doi: 10.1001/jamainternmed.2015.7667
137. Mbuagbaw L, Mursleen S, Lytvyn L, et al. Mobile phone text messaging interventions for HIV and other chronic diseases: an overview of systematic reviews and framework for evidence transfer. *BMC Health Serv Res* 2015;15:33. doi: 10.1186/s12913-014-0654-6
s12913-014-0654-6 [pii] [published Online First: 2015/01/23]
138. Muessig KE, Nekkanti M, Bauermeister J, et al. A Systematic Review of Recent Smartphone, Internet and Web 2.0 Interventions to Address the HIV Continuum of Care. *Curr Hiv/Aids Rep* 2015;12(1):173-90. doi: 10.1007/s11904-014-0239-3
139. Gabarron E, Wynn R. Use of social media for sexual health promotion: a scoping review. *Global health action* 2016;9:32193. doi: 10.3402/gha.v9.32193 [published Online First: 2016/09/22]
140. Chavez NR, Shearer LS, Rosenthal SL. Use of digital media technology for primary prevention of STIs/HIV in youth. *J Pediatr Adolesc Gynecol* 2014;27(5):244-57. doi: 10.1016/j.jpag.2013.07.008 [published Online First: 2013/12/18]
141. Barello S, Triberti S, Graffigna G, et al. eHealth for Patient Engagement: A Systematic Review. *Front Psychol* 2015;6:2013. doi: 10.3389/fpsyg.2015.02013 [published Online First: 2016/01/19]
142. Finitsis DJ, Pellowski JA, Johnson BT. Text Message Intervention Designs to Promote Adherence to Antiretroviral Therapy (ART): A Meta-Analysis of Randomized Controlled Trials. *PLoS ONE* 2014;9(2) doi: e88166
10.1371/journal.pone.0088166
143. Fletcher WR, Fletcher WS. Chapter 8: Treatment. Clinical Epidemiology: The Essentials. 4th ed. Baltimore: Lippincott Williams & Wilkins 2005:141.

For peer review only - http://bmjopen.bmj.com/site/about/guidelines.xhtml

144. Agarwal S, LeFevre AE, Lee J, et al. Guidelines for reporting of health interventions using mobile phones: mobile health (mHealth) evidence reporting and assessment (mERA) checklist. *BMJ* 2016;352:i1174. [published Online First: 2016/03/19]

For peer review only

For peer review only - http://bmjopen.bmj.com/site/about/guidelines.xhtml

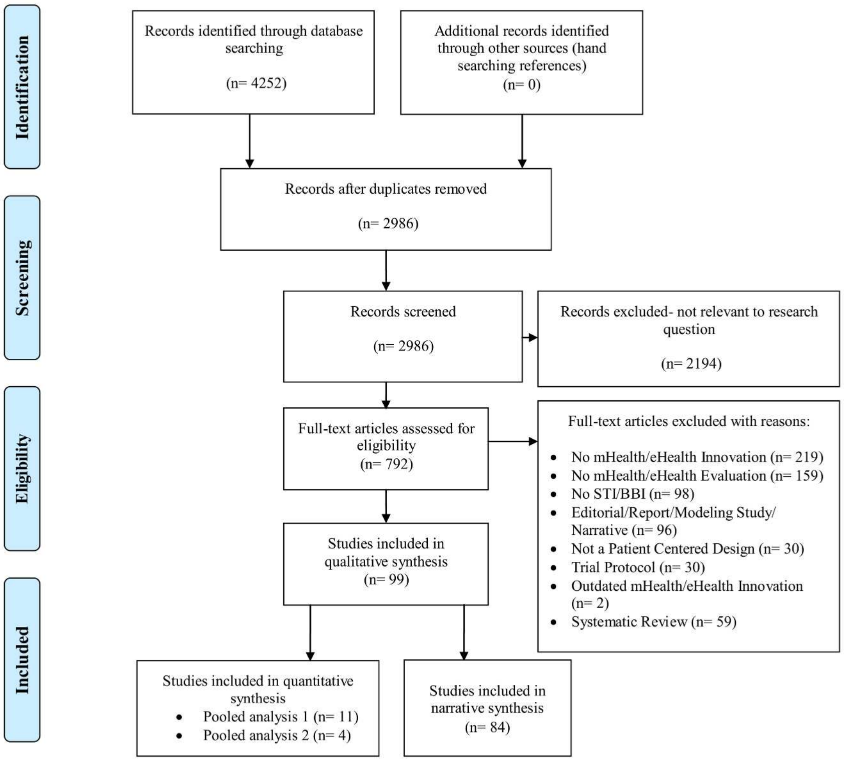

Figure 1: PRISMA flow diagram

215x279mm (300 x 300 DPI)

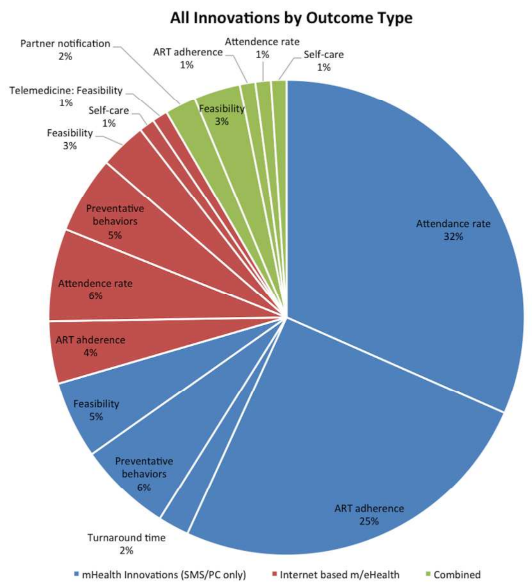

Figure 2. All Innovations by Outcome Type

279x162mm (300 x 300 DPI)

For peer review only

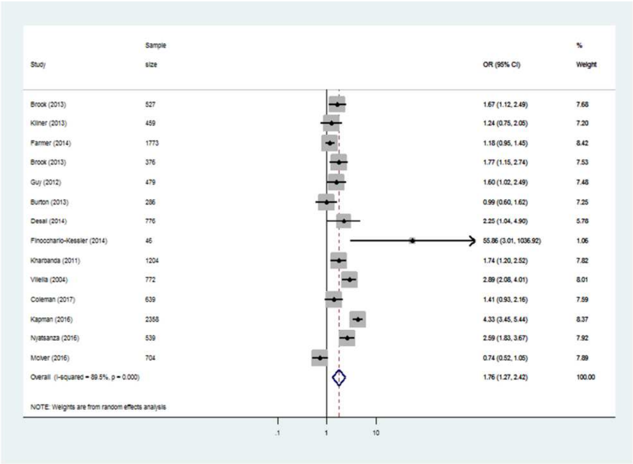

Figure 3A. Sub-Group Analysis Pooled OR for Attendance

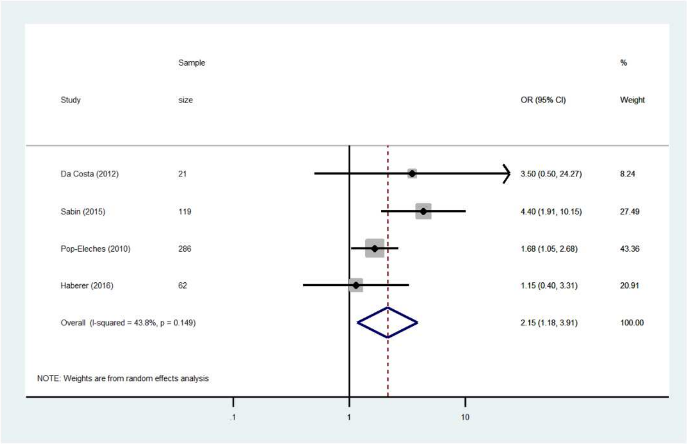

Figure 3B. Sub-Group Analysis Pooled OR for Adherence

Figure 3. Sub-Group Analyses

215x279mm (300 x 300 DPI)

Appendix 1: Search Strategy.

Search #1	"HIV Infections"[Mesh] OR "HIV" [MeSH] OR "human immunodeficiency virus"[tiab] OR "human immuno deficiency virus"[tiab] OR "human immune deficiency virus"[tiab] OR "human immunedeficiency virus"[tiab] OR "aids"[tiab] OR "acquired immunodeficiency syndrome"[tiab] OR "acquired immunodeficiency syndromes"[tiab] OR "acquired immuno deficiency syndrome"[tiab] OR "acquired immuno deficiency syndromes"[tiab] OR "acquired immune deficiency syndrome"[tiab] OR "acquired immune deficiency syndromes"[tiab] OR "acquired immunedeficiency syndrome"[tiab] OR "acquired immunedeficiency syndromes"[tiab]
Search #2	"mHealth" [tiab] OR "telemedicine"[MeSH] OR telemedicine[tiab] OR eHealth[MeSH] OR ehealth[tiab] OR "mobile health" [tiab] OR "mobile technology"[tiab] OR "app"[tiab] OR "apps"[tiab] OR "mobile applications" OR social medi*[tiab] OR cell phone* [tiab] OR cellphone*[tiab] OR "cellular phone"[mesh] OR cellular phone*[tiab] OR smartphone*[tiab] OR smart phone*[tiab] OR mobile phone[tiab] OR mobile device*[tiab] OR cellular telephone*[tiab] OR mobile telephone*[tiab] OR text messag*[tiab] OR texting[tiab] OR texted[tiab] OR SMS[tiab] OR MMS[tiab] OR multimedia messag*[tiab] OR short messag*[tiab] OR "computers, handheld"[mesh] OR personal digital assistant*[tiab]
Search #3 [1,2]<br><br>References<br><br>1.Ferreira A, Young T, Mathews C, Zunza M, Low N. Strategies for partner notification for sexually transmitted infections, including HIV. Cochrane Database of Systematic Reviews 2013, Issue 10. Art. No.: CD002843. DOI: 10.1002/14651858.CD002843.pub2<br><br>2.Obiero J, Mwethera PG, Wiysonge CS. Topical microbicides for prevention of sexually transmitted infections. Cochrane Database of Systematic Reviews 2012, Issue 6. Art. No.: CD007961. DOI: 10.1002/14651858.CD007961.pub2	sexually transmitted infections[mh] OR sexually transmitted disease*[tiab] OR sexually transmissible disease*[tiab] OR sexually transmitted infection*[tiab] OR sexually transmissible infection*[tiab] OR sexually transmitted infectious disease*[tiab] OR sexually transmissible infectious disease*[tiab] OR sexually transmitted disorder*[tiab] OR sexually transmissible disorder*[tiab] OR STI[tiab] OR STIs[tiab] OR STD[tiab] OR STIs[tiab] OR venereal disease*[tiab] OR venereal infection*[tiab] OR venereal disorder*[tiab] OR genital herpes[tiab] OR herpes genitalis[mh] OR herpes genitalis[tiab] OR genital infection*[tiab] OR genital disorder*[tiab] OR herpes simplex[tiab] OR herpes virus[tiab] OR HSV-1[tiab] OR HSV-2[tiab] OR chancroid[mh] OR chancroid* [tiab] OR haemophilus ducreyi[tiab] OR chlamydia infection*[tiab] OR chlamydia trachomatis[mh] OR chlamydia trachomatis[tiab] OR gonorrhea[mh] OR gonorrhoea*[tiab] OR gonorrhea*[tiab] OR syphilis[mh] OR syphilis[tiab] OR cuminat[tiab] OR condylomata lata[tiab] OR chancre*[tiab] OR lymphogranuloma venereum[mh] OR lymphogranuloma venereum[tiab] OR granuloma Inguinale[mh] OR granuloma inguinale[tiab] OR donovania[tiab] OR donovanosis[tiab] OR calymmatobacterium[mh] OR calymmatobacterium granulomatis[tiab] OR klebsiella granulomatis[tiab] OR klebsiella granulomatis[tiab] OR treponema pallidum[mh] OR treponema pallidum[tiab] OR genital wart*[tiab] OR venereal wart*[tiab] OR condylomata cuminate[mh] OR human papillomavirus 6[mh] OR hpv-6[tiab] OR hpv-11[tiab] OR hpv6[tiab] OR human papillomavirus[tiab] OR hepatitis b[mh] OR hepatitis b[tiab] OR trichomonas vaginitis[mh] OR trichomonas vaginitis[tiab] OR genital ulcer*[tiab] OR anogenital ulcer*[tiab] OR anorectal ulcer*[tiab] OR anorectal ulcer*[tiab] OR penile ulcer*[tiab] OR blood-born pathogen*[tiab] OR blood-borne infection*[tiab] OR blood-borne virus*[tiab]
Search #4	#1 OR #3
Search #5	#2 AND #4

For peer review only - http://bmjopen.bmj.com/site/about/guidelines.xhtml

Appendix 2: **Abstraction table.**

Combined Innovations	Author	Study Design	Participants/ Country	STBBI	Intervention	Measure/Metric	Results (MD=Mean difference, RR=risk ratio, IRR=incidence rate ratio, HR=hazard ratio, SD= standard deviation, 95% CI when presented. M=months, W=weeks)
**Online campaign + SMS + TV.**	Friedman 2014	Quasi-experimental: HxCtrl w/ population data.	≤25 yrs, USA. n=N/A	HIV, CT, GC	GetYourselfTested: TV campaign w/ website & SMS service for STI info & clinic locator.	ATT testing: Attendance rate.	41.2% more CT tests in 2010 vs. 2008, 90.5% more GC tests, and 190.3% more HIV tests.
						Acceptability: Number of followers.	4477 FB followers and 1994 Twitter followers at yr 2.
						Feasibility: Referral rate.	83,404 referrals using clinic locator in yr1. 61,119 in yr2.
**Interactive website + SMS + cash incentives.**	Horvath 2013	RCT	HIV+ Gay/Bi-sexual men 18+ yrs, USA. n=67(Int) n=57(Ctrl)	HIV	Online self-monitoring system w/ interactive interface + optional SMS reminders +$25 gift card draw.	ART in PVLA: Self-report. [Difference scores: DS = FU-baseline]	No difference. (DS=0.54, SD=25.2 vs. DS=-3.2, SD=24.5; t(107)=1.79, p=0.43) / No impact.
						ART in PVLA: Self-report.	Increased adherence in drug users (DS= 7.1, SD= 22.1 vs. DS= -24, SD= 30.5; t(17)=2.52, p=0.02) / Effective.
						ART in PVLA: Self-report.	Trend to taking meds within 2hrs of scheduled dose. DS=6.6, SD=29.3 vs. DS=-3, SD=29.6; t(105)=1.68, p=0.1 / No impact.
						Acceptability: Self-report.	Mean score = 5.7 on 7-point Likert Scale for satisfaction / Highly acceptable.
						Feasibility: Completion rate.	Completion rate 88% vs. 93% in Ctrl / Highly feasible.
**Website + SMS**	Gotz 2014	Cross-sectional study.	STI index patients at clinic, NLD. n=988	HIV, CT, GC, syph	Suggestatest.nl: online partner notification via SMS/email.	PN: % partners notified.	14% notifications via SAT. 505 notifications sent (84% by SMS, 15% by email). 56% read notification. 20% visited one of 2 STI clinics.
**Social media + SMS.**	Hightow-Weidman 2014	Quasi-experimental: HxCtrl.	HIV+ or syphilis+ patients, USA. n=362(Int) n=133(HxCtrl)	HIV, syphilis	Notification on social networking sites + SMS	PN: % partners notified.	63.5% of contacts notified via internet in 2011 vs. 26% in 2010.
**PC/SMS/MMS + WhatsApp messages**	John 2016	UnCtrlled trial.	HIV+ non-disclosed, 15-29 yrs, NGA. n=19	HIV	Weekly counselling, educational & motivational calls, SMS/MMS and WhatsApp messages over 3M.	Self-care: Self-report.	Significant increase in self-care performance at 6Ml (p=0.002)/ Effective.
**Interactive website + SMS**	Hightow-Weidman 2015	Feasibility study.	Black MSM & transwamen 18-30 yrs, USA. n=15	HIV	HealthMpowerment.org: online community networking Int to reduce STI risk + health promotion messages.	Acceptability: Self-report.	86.7%-100% strongly agreed w/ acceptability questions / Highly acceptable.
						Feasibility: Retention rate.	100% retention rate. 7/15 participants used the site 1W after study ended / Highly feasible.
**Mobile app + SMS**	Hirsch-Moverman 2017	Feasibility study.	≥18yrs, HIV+/TB, LSO. n=171	HIV/TB	CommCare application used to automatically send SMS medication reminders over 29M	Acceptability: Self-report.	41.9% think SMS facilitated adherence to TB /ART medication / Less acceptable.

For peer review only

For peer review only - http://bmjopen.bmj.com/site/about/guidelines.xhtml

**Mobile app + SMS**	Aronson 2016	Feasibility study	18-24 yrs, USA. n=100	HIV	App assessing risk and sending SMS to encourage re-testing of HIV negatives.	Feasibility: Completion rate	98/100 completed the app process/ Highly feasible<br>30/100 accepted to receive HIV test<br>21/30 accepted to receive SMS<br>1/21 re-tested after 90 days window period.
**Website + SMS**	Dokkum 2012	UnCtrlled trial.	16-29 yrs, NLD. n=52600(Rd 1) n=41700(Rd 2)	CT	At-home CT test + SMS/email to return test for analysis.	Feasibility: Completion rate.	Higher retesting rates (From 10% w/o reminders to 14% in round 1; from 7% to 10% in round 2) / Less feasible.

Note: Int= intervention; Ctrl= control; HxCtrl= historical control; PB= preventative behaviors (i.e. risk reduction); PN= partner notification; TAT= turnaround time; ATT= attendance rate; ART= ART adherence; NAPs= non-adherent patients; AP= adherent patients; PVLA= Patients with various levels of adherence; TNPs= Treatment naive patients; VL= viral load; CD4= CD4 cell count; PC= phone call; FB= Facebook.

For peer review only

Internet-based eHealth Innovation	Author	Study Design	Participants/ Country	STBBI	Intervention	Measure/Metric	Results (MD=Mean difference, RR=risk ratio, IRR=incidence rate ratio, HR=hazard ratio, SD= standard deviation, 95% CI when presented. M=months, W=weeks)
**Online campaign**	Downshen 2015	Quasi-experimental: HxCtrl w/ population data.	13-17 yrs, USA. n=1500	HIV, CT, GC, syphilis	IknowUshould2: social-media campaign w/ website for STI info & clinic locator.	ATT testing: Attendance rate.	More syphilis tests (18.8% vs. 5.4%; p<0.01) and HIV tests (19.0% vs. 5.4%; p<0.01). No change for CT & GC / Effective.
						Acceptability: Number of followers.	1500+ unique website interactions. 128 FB likes; 46 Twitter followers; 390 Youtube views; 42 Instagram followers.
**Social media campaign**	Elliot 2016	Cross-sectional study.	MSM, GBR. n=17361	HIV	Promotion through Gaydar, Grindr, Recon and FB pages to order free postal HIV home sampling kits	ATT testing: Participation rate.	10 323/11 127 (93%) ordered HIV sample kit. 5696/10 323 (55%) returned sample kit within 24M. 82/5696 (1.4%) confirmed new diagnosis and in care.
						Acceptability: Self-report.	59.7% would recommend to someone expected to test positive (93.8% if expected to negative). 64% clicked for more info on test. / Moderately acceptable.
**Social media campaign**	Huang 2016	Cross-sectional	≥18yrs, Black/African American or Hispanic/Latino MSM, USA. n=122	HIV	Promoting of HIV self-testing for 6W on GrindR + study website to order self-test kit	ATT testing: Participation rate.	122 requested tests; 55/57 HIV-, 2/57 HIV+.
						Acceptability: Number of followers	11 939 unique website visitors; 2.8% click-through rate 334 tests requested.
						Feasibility: Completion rate.	122/334 visitors were eligible and completed baseline survey, 81/122 confirmed receiving self test kit, 57/122 completed follow-up survey / Less feasible.
**Social media campaign**	Jones 2015	Cross-sectional study.	MSM, GBR. n=305	HIV	Health promotion and offer of rapid at-home testing via FB, Grindr, and Squirt.	ATT testing: Participation rate.	5/5 high risk sexual behavior but tested HIV negative; 1/5 never tested before; 3/5 not tested in many yrs.
						Acceptability: Number of followers.	103 clicked FB survey; 152 approached on Grindr; 50 Squirt contacts.
						Feasibility: Completion rate.	FB: 6/103 completed survey; 3/6 requested HIV test; 2/3 made appointment. Grindr: 20/152 engaged; 6/20 requests for at home test; 3/6 made appointment. Squirt: 3/50 engaged and 0/3 test requests / Less feasible.
**Social media campaign**	Rhodes 2016	Quasi-experimental.	MSM & transgender, USA n=339 (Int) n=286 (Ctrl)	HIV	Posting info and answering questions on HIV testing on social media sites (Adam4Adam, BlackGayChat, Craigslist, and Gay.com).	ATT testing: Self-report.	63.7% of intervention participants reported past 12M HIV testing compared with 42.0% of control. Adjusted OR= 2.9 (1.8-4.7)/ Effective.
**Social media campaign + website**	Rosengren 2016	Cross-sectional	Black or Hispanic MSM 18+ yrs, USA n=56	HIV	Promotion of free rapid HIV self-testing kits on Grindr and offer of delivery via study website (kit, voucher or pin for smart vending machine)	ATT testing: Self-report.	All 56 reported testing completion (100%); 2/56 reported positive result and linkage to care (confirmatory testing and ART initiation)
						Feasibility: Completion rate.	4389 visited the website; 333 requested test (i.e. 1 in 13 visitors); 56 completed survey 2W after request/ Less feasible.
						ART in TNPs: Self-report.	Higher adherence at 3M & 6M (71.2% vs. 63.9%, d=0.17; 70.3% vs. 66.6%, d=0.09)
**Mobile phone application**	Himelhoch 2016	RCT	18-64yrs, history of drug/alcohol use, HIV+, USA. n=19(Int) n=9(Ctrl)	HIV	Heart2HAART mobile application for ART adherence	ART in NAPs: Pill count	No significant difference in adherence between intervention and control group (p=0.29), but adherence was 100% in both at 3M / No impact
						Acceptability: Self-report.	94.3% strongly agreed/agreed Heart2HAART helped them take their medication / Highly acceptable.
						Feasibility: Response rate.	App was used on avg 21.4, 19.1 and 16.4 times in months 1, 2 and 3. Participants responded to medication prompts on avg 18, 16 and 14 times during months 1,2 and 3 respectively.

For peer review only - http://bmjopen.bmj.com/site/about/guidelines.xhtml

**Avatar-guided computer software**	Kurth 2014	RCT	HIV+ 18+ yrs, USA. n=120(Int) n=120(Ctrl)	HIV	Audio narrated risk assessment, skill building videos, tailored feedback and printouts vs. computer risk assessment only.	ART in PVLA: VL.	Non-significant change. (log10VL= -0.06(-0.4 to -0.3), p=0.74). Significant in subgroup w/ detectable VL at baseline (-0.73(-1.42 to -0.03), p=0.041) / No impact.
						ART in PVLA: Self-report.	Increased adherence. (4.71(0.95- 8.48) increase vs. 1.39(6.03 to 3.24) decrease; p=0.046) / Effective.
						PB: Self-report.	Lower odds of HIV transmission (OR=0.46 (0.25-0.84), p=0.012) / Effective.
						Acceptability: Self-report.	97% reported ease of use and high privacy; 99% satisfied w/ session length; 75% preferred it over human counsellor / Highly acceptable.
						Feasibility: Retention rate.	87.1% retention / Highly feasible.
**Avatar-guided computer program**	Naar-King 2012	RCT	HIV+ 16-24 yrs, USA. n=36(Int) n=40(Ctrl)	HIV	2-D animated character delivering personalized health feedback vs. character giving nutrition info.	ART in TNPs: VL.	Larger suppression rate. (Cohen's d=0.09 at 3M; d= 0.28 at 6M). Larger drop in VL from baseline (d=0.39 at 3M & d=0.19 at 6M).
						ART in TNPs: Self-report.	Higher adherence at 3M & 6M (71.2% vs. 63.9%, d=0.17; 70.3% vs. 66.6%, d=0.09)
						Acceptability: Self-report.	Mean satisfaction ratings 3.7 out of 4 / Highly acceptable.
**Mobile phone application**	Perera 2014	RCT	HIV+, NZ. n=17(Int) n=11(Ctrl)	HIV	ART adherence app w/ medication clock & graphs on disease-state vs. standard app (medication clock only)	ART in PVLA: Self-report.	Increased adherence (F(1,23)=5.37, p=0.03) / Effective.
						ART in PVLA: Pharmacy refills.	No difference. (F(1,25)=1.88, p=0.18) / No impact.
						ART in PVLA: VL.	Lower VL at 3M (F(1,23)=5.62, p=0.023) / Effective.
						ART in PVLA: Composite score (refills, VL, & self-report).	Increased adherence (53% to 13%, X2(1,15)=6, p=0.03). No change in Ctrl (27% to 27%, X2(1,11)=0.00, p>0.99) / Effective.
						Acceptability: Self-report.	More satisfying (on 11 point-scale: 5.88 vs. 3.27, p=0.017) and informative (6 vs. 3, p=0.034) at 3M than standard app / Highly acceptable.
**Mobile app + cash incentive**	Brayboy 2017	UnCtrlled trial.	12-17yrs, USA. n=17	STI	GirlTalk mobile phone app to assess knowledge increase	PB: Self-report.	75.6% to 79% increase in knowledge pre and post app use at 2W. / No impact.
						Acceptability: Self-report.	94.1% would use the app again/recommend it / Highly acceptable
**Social media**	Jones 2012	Quasi-experimental: HxCtrl.	15–24 yrs, USA. n=70/896 FB friends	CT	Educational FB site addressing safe sexual health.	PB: Self-report.	Condom from 57% to 80%. 54% reduction in CT in ages 15-17 from previous yrs (but 42% less tests done).
**Videos vs. SMS**	Jones 2013	RCT	High-risk urban African-American women 18-29 yrs, USA. n=117(Soap opera) n=121(SMS)	HIV	Weekly soap opera episodes (Love, Sex & Choices) vs. HIV prevention SMS.	PB: Self-report.	18% greater reduction in Int. group, p=0.23 / No impact. 78% reduction in risky acts from baseline in Int. group (p<0.001); 72% reduction from baseline in Ctrl (p<0.001)/ Effective
						Acceptability: Self-report.	97.4% liked the videos / Highly acceptable.
**Social media + video chat**	Lelutiu-Weinberger 2014	UnCtrlled trial.	MSM 18-29 yrs, high risk for STI, USA. n=31	HIV	miCHAT: FB chat Int. 8 motivational interviews to reduce HIV risk + CBT training.	PB: Self-report.	Decrease in unprotected anal sex acts (3.11 vs. 8.96; p=0.042). Increased knowledge of sexual risk (p=0.01) / Effective.
						Acceptability: Self-report.	All felt privacy was ensured / Highly acceptable.
						Feasibility: Completion rate.	46% completed baseline assessment + minimum 5 sessions / Less feasible.

For peer review only

**Social media campaign + website + cash incentive**	Solorio 2016	Feasibility study.	Hispanic MSM, 18-30 yrs, USA<br>n=50	HIV	Radio & social media-based campaign for 16W to encourage testing & condome use + website w/clinic locator to provide free HIV home testing kits and linkage to care	PB: self-report.	No significant change in condom use at 16W (26.1% vs. 15.65, OR=1.9 (0.6-5.9))/ No impact.
						Feasibility: Self-report.	32/50 (64%) requested HIV home testing kit, 28/32 (88%) completed the test/ Moderately feasible.
**Mobile app**	Jeon 2016	RCT.	Chronic HBV+, 19-60 yrs, KOR<br>n=26 (Int)<br>n=27 (Ctrl)	HBV	App to increase disease knowledge, set alarm medication reminders, record lab nutrition & physical activity data, and chat with other users.	Self-care: Self-report.	Significantly higher self-care performance in intervention vs. control (t=3.597, p=0.001)/ Effective.
						Feasibility: Utilisation rate.	Average monthly utilisation rate was 75.1%/ Highly feasible.
**Social media**	Henwood 2016	Feasibility study.	12-25 yrs, HIV+, ZAF<br>n=90	HIV	Use of MXit as support group for HIV+ youth	Acceptability: Self-report.	84% would like chat-room to continue / Highly acceptable.
						Feasibility: Participation rate	33% ever visited MXit chat-room / Less feasible.
**Mobile app + cash incentive**	Przybyla 2016	Feasibility study.	HIV + on ART, 18+ yrs, USA<br>n=27	HIV	DRUM app to report daily on ART adherence and substance abuse.	Acceptability: Self-report.	84% reported the app was easy to use; 96% were satisfied; 92% would use it in the future/ Highly acceptable.
						Feasibility: Completion rate.	Overall completion rate of daily reports after 2W= 95.3%/ Highly feasible.
**Telemedicine**	Talal 2016	Feasibility study.	Individuals on opioid agonist tx, USA<br>n=54	HCV	Telemedicine-based medical tx with hepatologist	Acceptability: Self-report.	88.9% prefer medical tx using telemedicine vs. clinic visit; 100% would recommend it to a friend/ Highly acceptable.
						Feasibility: Completion rate.	54 tested HCV+ over 14M; 81.5% started evaluation/tx; 75% of those given tx have completed it/ Highly feasible.
**Social media**	Garett 2016	Feasibility study.	18+yrs, MSM, PER<br>n=102(Int)<br>n=109(Ctrl)	HIV	12W FB based peer-led intervention to increase HIV testing and prevention behaviour.	Acceptability: Self-report.	Intervention group felt they learned more about; where to receive sexual health services (p-value=0.0061), more likely to have safe sex (p-value=0.034) and more likely to get tested for HIV regularly (p-value=0.021) compared to control group / Highly acceptable.
**Website**	Polilli 2016	Feasibility study.	Residents of Abruzzo Region, ITA<br>n=3500	HIV, syphilis, HBV, HCV	Website with STI info, risk calculator, and appointments booking at testing sites.	Feasibility: Completion rate.	3500 booked an appointment; 3046 (87%) presented for testing within 15M study period/ Highly feasible.

Note: Int= intervention; Ctrl= control; HxCtrl= historical control; PB= preventative behaviors (i.e. risk reduction); PN= partner notification; TAT= turnaround time; ATT= attendance rate; ART= ART adherence; NAPs= non-adherent patients; AP= adherent patients; PVLA= Patients with various levels of adherence; TNPs= Treatment naive patients; VL= viral load; CD4= CD4 cell count; PC= phone call; FB= Facebook.

For peer review only

Basic mHealth Innovation	Author	Study Design	Participants/ Country	STBBI	Intervention	Measure/Metric	Results (MD=Mean difference, RR=risk ratio, IRR=incidence rate ratio, HR=hazard ratio, SD= standard deviation, 95% CI when presented. M=months, W=weeks)
**SMS**	Bailey 2014	UnCtrlled trial.	CT+ at clinic, AUS. n=64	CT	SMS reminders to recall for treatment.	ATT treatment: Attendance rate.	100% treated for CT infection. 72% treated within 1 day of SMS.
						Feasibility: Response rate.	94% replied to SMS, 84% the same day / Highly feasible.
**SMS + PC**	Bassett 2016	RCT	≥18yrs, ZAF. n=543(Int) n=471(Ctrl)	HIV/TB	5 scheduled PC) and 4 SMS, reminders to retrieve test results and attend appointments, over 4M.	ATT treatment: Attendance rate.	No significant difference in reaching outcome at 9M (3M ART treatment or 3+6M of TB treatment) between intervention and control (39% vs 42%, RR=0.93, 95%CI 0.80-1.08) / No Impact
**SMS + PC**	Bigna 2014	RCT	Caregivers of HIV + children 18+ yrs, CMR. n=61(SMS+PC) n=60(PC) n=60(SMS) n=61(Ctrl)	HIV	SMS+PC, SMS, or PC appointment reminders.	ATT FU appointment: Attendance rate.	Improved attendance. (OR=2.9 (1.3-6.3), p=0.012) / Effective.
**SMS**	Brook 2013	Quasi-experimental: HxCtrl.	Sexual health clinic, GBR. n=207(Int) n=169(HxCtrl)	HIV, syphilis, HBV	SMS reminders.	ATT testing: Attendance rate.	Higher retesting rate (41% vs. 28%; p<0.001) / Effective.
**SMS**	Brook 2013	Quasi-experimental: HxCtrl.	Sexual health clinic, GBR.n=699(Int) n=768(HxCtrl)	HIV	SMS reminders 2 days before appointment.	ATT FU appointment: LTFU rate.	35% improvement in overall LTFU rate (26% to 17%; p<0.0001) / Effective.
**SMS**	Burton 2013	Quasi-experimental: HxCtrl.	High risk for STI at clinic, GBR. n=273(Int) n=266(Ctrl)	CT, GC	SMS STI testing reminders.	ATT: testing: Attendance rate.	No change in retesting rates for those w/ recent CT or GC. (CT: 36% vs.33%; p=0.79) (GC: 19% vs. 33%; p=0.48) / No impact.
**SMS**	Coleman 2017	Retrospective Quasi-experimental	>=18 yrs, HIV+ pregnant women, ZAF. n=192(Int) n=447(Ctrl)	HIV	Bi-weekly maternal health info sent throughout pregnancy and for one year after birth to increase HIV PCR testing postpartum and increase ANC visits	ATT testing: Attendance rate.	81.3% vs 75.4% in intervention vs control group likely to attend first PCR 6W postpartum. 40% increase in the likelihood of attending the recommended four ANC visits among individuals within the intervention group (RR: 1.41, CI: 1.15–1.72) / Effective.
						PB: Infection rate	3 infants born with HIV in control group
**SMS**	Desai 2014	Quasi-experimental: Conc. + HxCtrl.	High risk MSM at clinic, GBR. n=31(Int) n=656(Conc. Ctrl) n=745(HxCtrl)	HIV	SMS HIV/STI testing reminders.	ATT testing: Attendance rate.	No significant change in re-testing odds. (32% in SMS vs.30% in Conc. Ctrl; OR=1.1(0.5-2.4) and 17% in HxCtrl; OR=2.3(1.0-4.9) / No impact.
**SMS + cash incentive**	Downing 2013	RCT	CT + or suspected at clinic 16+ yrs, AUS. n=30(Int) n=32(Ctrl)	CT	SMS appointment reminders + $10 if attended.	ATT testing: Attendance rate.	Increased re-testing rate at 10-12W post CT treatment (without cash 26.7% vs. 6.3% in Ctrl; p=0.04); (with cash 28.1% vs. 6.3% in Ctrl; p=0.043) / Effective.
**SMS**	Evans 2015	UnCtrlled trial.	African community, GBR. n=172	HIV	2 weekly Health Belief Model SMS to reduce risky sexual behaviours.	ATT testing: Self-report.	10.5% reported being tested for HIV during/after the 12W Int.
						PB: Self-report.	Non-significant increase in HIV knowledge & attitudes / No impact.
						Acceptability: Self-report.	Acceptable & useful. Majority shared w/ others and want to get tested in future.

For peer review only

**SMS**	Farmer 2014	Quasi-experimental: HxCtrl.	HIV clinic attendees, GBR. n=951(Int) n=822(HxCtrl)	HIV	SMS reminder 2 days before appointment.	ATT FU appointment: LTFU & cancellation rate.	No difference in LTFU (25% vs.28%) or cancellation (62% vs.64%) / No impact.
**SMS**	Finocchario-Kessler 2014	Quasi-experimental: HxCtrl.	HIV+ mother-infant pairs, KEN. n=523(Int) n=320(HxCtrl)	HIV	SMS notification of available test results and appointment reminder.	ATT treatment: Attendance rate.	More infants initiated on ART (Urban: 11/11 vs. 1/7, p<0.001; Peri-urban: 14/14 vs. 9/14, p<0.05) / Effective.
						TAT: Time from test to diagnosis & test to treat.	Shorter median time to diagnosis (5 vs. 6.3W (urban) & 3.4 vs. 8.1W (peri-urban); both p<0.001). Shorter median time to treat (13 vs. 40 days (urban) & 1 vs. 36 days (peri-urban); p<0.001) / Effective.
						Feasibility: Retention rate.	Retention rate double at 9M post-natal (45.1% vs. 93% (urban) and 43.2% vs. 94.1% (peri-urban); p<0.001) / Highly feasible.
**SMS**	Guy 2012	Quasi-experimental: HxCtrl.	STI clinic, AUS. n=141(Int) n=338(HxCtrl)	CT	SMS re-testing reminder 3M after initial infection.	ATT testing: Attendance rate.	Higher retesting rate (30% 1-4M post-infection vs. 21%; p=0.04); AOR= 1.57(1.01-2.46) / Effective.
**SMS**	Joseph Davey 2016	RCT.	HIV+ adults on ART, MOZ n=416 (Int) n=414 (Ctrl)	HIV	SMS reminders 2 and 7 days of appointment and ART drug-pick up + educational SMS every 2M.	ATT treatment: Attendance rate.	Nonsignificant difference in overall retention in care at 12 M (93.8% vs 91%, p=0.139)/ No impact.
**SMS**	Kapman 2016	Quasi-experimental: HxCtrl.	Heterosexual clinic attendees dx & tx for CT, 16-23 yrs, NLD n=828 (Int) n=1530 (Ctrl)	CT	2 SMS reminders at 5.5M & 6M after initial dx with CT for retesting appointment scheduling & attendance.	ATT testing: Attendance rate.	Higher attendance rate between 5-8M after initial dx (30.6% vs. 9.2%).
**SMS**	Kharbanda 2011	Quasi-experimental: Conc. + HxCtrl.	Parents of girls 9-20 yrs at clinics, USA. n=124(Int) n=308(Conc. Ctrl) n=1080(HxCtrl)	HPV	Up to 3 weekly SMS vaccination reminders.	ATT vaccination: Attendance rate.	More likely to get vaccine on time after controlling for insurance and site of care (AOR=1.83(1.23-2.71)) / Effective.
**SMS**	Kliner 2013	Quasi-experimental: HxCtrl.	HIV+ at hospital, SWZ. n=162(Int) n=297(HxCtrl)	HIV	SMS reminders one day before appointment.	ATT FU appointment: Attendance rate.	No difference. SMS 83.3% vs. Ctrl 80.1%; p=0.401. AOR=1.13, p=0.662 / No impact.
**SMS**	Matheson 2014	Quasi-experimental.	11-22 yrs at clinic, USA. n=37(Int) n=232(Ctrl)	HPV	SMS vaccination reminders (3 SMS per dose).	ATT vaccination: Attendance rate.	Higher attendance rate. HPV2 vaccine complete: 73% vs.34%, (p=0.000); on-time HPV2 38% vs. 25%, (p=0.035). HPV3 complete 16% vs.6%, (p=0.018); on-time HPV3 14% vs.3%, (p=0.007) / Effective.
**SMS**	McIver 2016	Quasi-experimental: HxCtrl.	Clinic attendees susceptible to HBV (HIV+, bisexual, CSW, IDUs, Aboriginals), AUS n=241 (Int) n=463 (Ctrl)	HBV	SMS reminders 1 day before appointment for HBV vaccine doses 2&3 reattendance.	ATT vaccination: Attendance rate.	Nonsignificant decrease in attendance rate within 12 M (54% vs 56% for 2 doses, p=0.65/ 24% vs 30% for 3 doses, p=0.07)/ No impact Nonsignificant difference in completion of 3 doses in 12M. aOR= 0.7 (0.48-1.01)/No impact.
**SMS**	Njuguna 2016	RCT.	Rural women, 18-24 yrs, KEN n=300 (Int) n=300 (Ctrl)	HIV	Weekly SMS on HIV and reproductive health.	ATT testing: Self-report.	Significant increase in reported testing at 6M (67% vs 51%, aHR=1.54(1.25-1.90)/ Effective.
**SMS vs. PC**	Norton 2014	RCT	HIV+, 17+ yrs, USA. n=25(Int) n=27(Ctrl)	HIV	SMS appointment reminder vs. message to home phone.	ATT FU appointment: Attendance rate.	No difference (72% vs. 81%, p=0.42) but patients already had high attendance rate / No impact.

For peer review only

**SMS**	Nyatsanza 2016	Quasi-experimental: HxCtrl.	MSM & CSW at high-risk of STI, GBR n=266 (Int) n=273 (Ctrl)	HIV/STI	Personalised SMS reminders for reattendance.	ATT testing: Attendance rate.	Significantly higher reattendance rate at 6M (56% vs. 33%, p<0.001)/ Effective.
**SMS**	Odeny 2012	RCT	Males circumcised at clinic 18+ yrs, KEN. n=600(Int) n=600(Ctrl)	HIV	Daily SMS for 1W.	ATT FU appointment: Attendance rate.	Improved attendance within 3 days of post-operative clinic appointment: 65.4% vs.59.7% (RR=1.09(1.00–1.20); p=0.04) / Effective.
						PB: Self-report.	Abstention of sexual activity before FU: 28.3% vs. 25.2% (RR=1.13(0.91-1.38), p=0.3) / No impact.
**SMS**	Rand 2015	RCT	11-16 yrs at clinic, USA. n=1893(Int) n=1919(Ctrl)	HPV	SMS appointment reminders.	ATT vaccination: Attendance rate.	Higher HPV1 vaccination rate (16% vs. 13%; HR= 1.3(1.0-1.6); p=0.04) / Effective.
**SMS/PC**	Rand 2016	RCT.	Clinic attendees Parents of youth 11-17 yrs who received 1st HPV vaccine, USA. n=191 (SMS) n=200 (Ctrl); n=178 (PC) n=180 (Ctrl)	HPV	SMS appointment reminders to receive 3 doses of HPV vaccine over 2 yrs.	ATT vaccination: Attendance rate.	SMS: Significant difference in vaccination rates compared to control (49% vs 30%, p=0.001)/ Effective. PC: No difference in vaccination rates compared to control (48% vs 40%, p=0.34)/ No impact.
						TAT: Time from enroll to completion of 3 vaccines.	SMS: Significant difference in time taken to complete 3 HPV doses (71 days earlier than control, p<0.001)/ Effective. PC: No difference in time taken to complete 3 HPV doses compared to control (p=0.08)/ No impact.
**SMS + PC**	Schwartz 2015	Quasi-experimental: HxCtrl.	HIV+ pregnant women on ART, ZAF. n=50	HIV	SMS messages and PCs from a case manager (CM) through 6W postpartum.	ATT testing: Attendance rate.	More infant testing (90.0% vs. 63.3% at 10W; p<0.01) / Effective.
						Acceptability: Self-report.	Helpful to have CM support during pregnancy and postpartum (98%) / Highly acceptable.
						Feasibility: Completion rate.	96% completed postpartum questionnaire / Highly feasible.
**SMS + PC**	Segaren 2012	UnCtrlled trial.	Mothers of HIV+ infants, HTI. n=108	HIV	Cell phones + regular PC for monitoring of mother & child.	ATT treatment: Attendance rate.	All 76 w/ active phones were adherent to treatment (attended 6/6 monthly hospital appointments).
						Acceptability: Self-report.	70% phones active after Int.; good for med reminders (63%) / Moderately acceptable.
**SMS + PC**	Smillie 2014	UnCtrlled trial.	HIV+ in clinic 14+ yrs, CAN. n=20	HIV	Weekly PC or SMS for 6M.	ATT FU appointment: Self-report.	65% said SMS had no effect on attendance.
						Acceptability: Self-report.	Beneficial for appointment scheduling (80%) & reminder (75%). All would recommend to a friend / Highly acceptable.
						Feasibility: Self-report.	75% had no difficulty in receiving and responding to SMS / Highly feasible.
**SMS**	Tolly 2012	RCT	Randomly sampled adults (existing database), ZAF. n=438(in each of 4 Int.) n=801(Ctrl)	HIV	3 or 10 motivational or informational SMS.	ATT testing: Self-report.	Improved attendance in group receiving 10 motivational SMS at 3W: (69% vs. 57%; OR=1.7(1.10–2.390), p=0.0036) / Effective.
						Feasibility: Self-report.	SMS motivated HIV counseling and testing uptake in 89% / Highly feasible.
**SMS**	Vilella 2004	Quasi-experimental: Conc. + HxCtrl.	18+ yrs at travel clinic, ESP. n=738(Int) n=1610(Conc. Ctrl) n=2247(HxCtrl)	HAV/ HBV	SMS reminders for vaccination appointments.	ATT vaccination: Attendance rate.	Improved adherence for 3rd HepA+B dose. (47.1% vs. 26.9%, RR=1.75(1.41–2.17) in Conc. Ctrl and 23.6%(20.1–27.4), RR=2.00(1.63–2.45) in HxCtrl) / Effective.

For peer review only

**SMS**	Ammassari 2010	UnCtrlled trial.	HIV+, ITA. n=71	HIV	SMS reminders.	ART in NAPs: Self-report.	Increased adherence over 9M. (93.2% vs.79.6%, p=0.003) / Effective.
**SMS**	Ammassari 2011	UnCtrlled trial.	HIV+, 18+ yrs, ITA. n=145	HIV	SMS reminders.	ART in NAPs: Self-report.	Increased adherence at 9M (94.9% vs.78.8%, p<0.001) / Effective.
						ART in NAPs: VL.	More w/ undetectable VL at 9M (76.2% vs. 42.3%, p<0.001) / Effective.
						Acceptability: Self-report.	>90% reporting SMS helpful / Highly acceptable.
**PC + cash incentives**	Belzer 2014	RCT	HIV+ 12-29 yrs, USA. n=19(Int) n=18(Ctrl)	HIV	Daily PC reminders and referrals if necessary+ free phone & plan.	ART in NAPs: Self-report.	Increased adherence for 1M &3 M (OR=3.09(1.20-7.98); OR=2.85(1.02-7.97)) / Effective.
						ART in NAPs: VL.	Lower VL at wk 24 and 48 (2.82 vs. 4.52, p=0.002; 3.23 vs. 4.23, p=0.043) / Effective.
**SMS**	Cantudo-Cuenca 2016	Retrospective quasi-exprimental.	HIV + on ART, ESP n=120 (Int&Ctrl)	HIV	SMS on ART adherence.	ART in PVLA: Pharmacy refills.	Statistically sign relationship bt no SMS and ART adherence(OR= 0.35 (0.14-0.8), p=0.025) [multivariate analysis]/ Effective.
**SMS**	da Costa 2012	RCT	HIV+ women, BRA. n=8(Int) n=13(Ctrl)	HIV	Daily SMS reminders.	ART in APs: Pill count.	Increased adherence over 4M (50% vs. 38.5%; p=0.604) / No impact.
						ART in APs: MEM.	Increased adherence over 4M (75% vs. 46%; p=0.195) / No impact.
						ART in APs: Self-report.	Increased adherence (100% vs. 84.6% in Ctrls; p=0.244) / No impact.
						Acceptability: Self-report.	82% believed SMS were helpful, 77% wanted to keep receiving SMS / Highly acceptable.
**SMS**	Downshen 2011	UnCtrlled trial.	HIV+ 14-29 yrs, AUS. n=25	HIV	Daily SMS ART reminder + FU SMS 1hr later.	ART in NAPs: Self-report.	Increased adherence (Baseline Mean=74.7; 12W Mean=93.3; 24WMean=93.1; p<0.001) / Effective.
						ART in NAPs: VL + CD4 count.	Insignificant change in CD4 cell count & VL (mean VL= 2750, CD4= 502 to VL= 29, CD4= 545 at 24W, p=0.12) / No impact.
						Acceptability: Self-report.	81% want SMS after study end. Helped decrease missed doses in 95% / Highly acceptable.
**SMS**	Downshen 2011	UnCtrlled trial.	HIV+ 14-29 yrs, AUS. n=25	HIV	Daily SMS ART reminder + FU SMS 1hr later.	ART in NAPs: Self-report.	Decreased adherence (58.3% for 0-12W vs. 55.2% for 13-24W, p=0.53) / No impact.
						Feasibility: Completion & response rate.	84% completed all study visits. 61.4% response rate / Highly feasible.
**SMS + cash incentive**	Garofalo 2016	RCT	16-29yrs, HIV+ on ART for ≥1M , USA. n=51(Int) n=54(Ctrl)	HIV	Daily personlised SMS over 6M to remind participants take medications	ART in NAPs: Self-report.	Significant difference in adherence compared to control at 3M OR=2.57 (1.01-6.54). Not significant at 6M OR=1.68 (0.69-4.09). Significant difference from baseline to 6M OR=2.12 (95% CI 1.01-4.45). / Effective.
						ART in NAPs: VL.	No difference in log viral load or viral suppression compared to control at 3 and 6M / No impact.
						Acceptability: Self-report.	100% would recommend intervention to those in need, 81 % wanted to continue getting the text messages after conclusion of the study, 95 % satisfied with the intervention overall / Highly acceptable
						Feasibility: Response rate.	58% average response rate to SMS / Moderately feasible.

For peer review only

For peer review only - http://bmjopen.bmj.com/site/about/guidelines.xhtml

**SMS +PC**	Haberer 2016	RCT	≥18yrs, HIV+ on ART, UGA. n=21(Scheduled SMS) n=20 (Triggered SMS) n=21(Ctrl)	HIV	Scheduled SMS: 1M daily SMS, 2M weekly SMS, 6M SMS sent to patient and support if needed. Triggered SMS; SMS sent to patient and support if no signal received from monitor.	ART: MEM	Significant difference in scheduled SMS intervention compared to control (11.1% increase in adherence, 48-h and more than 96-h lapses were less frequent (IRR=0.6, p value=0.02 and IRR 0.3, P<0.001, respectively). Similar adherence in triggered SMS vs control group. / Effective.
						ART: VL	No significant differences in HIV RNA suppression among study arms (p value = 0.14). 47/62 participants virally suppressed at 3 and 9M / No impact.
**SMS**	Hardy 2011	RCT	HIV+ 18+ yrs, USA. n=12(SMS) n=14(Beeper)	HIV	SMS vs. beeper reminders.	ART in APs: Composite score (MEM+ pill count + self-report).	Higher adherence at 6W. (MD=27.1(7.6-46.6), p =0.009) / Effective.
						ART in APs: MEM.	Increased adherence. (MD=33.4(14.1-52.6), p = 0.002) / Effective.
						ART in APs: Pill count.	No difference. (MD=13.7(-6.7-34.1), p = 0.153) / No impact.
						ART in APs: Self-report.	No difference. (MD=20.2 (-1.8-42.1), p = 0.069) / No impact.
**SMS**	Jeffries 2016	RCT	15-24yrs, HIV+, USA. n=91(Int) n=45(Ctrl)	HIV	UCARE4LIFE daily moblie text messageing intervenetion over 3M to improve HIV care among youth	ART: VL	Significant difference in ART adherence in intervention vs control among non-adherent/new to ART at baseline (6M p=0.03). / Effective. No sig difference in those on ART at baseline (6M p=0.119) /No impact.
						Acceptability: Self-report.	Mean score = 8.44 (SD=2.45) on 10 point Likert Scale for appointment reminder SMS./ Highly acceptable
**PC**	Kalichman 2011	RCT	HIV+ 18+ yrs, USA. n=21(Int) n=19(Ctrl)	HIV	PC counselling.	ART in NAPs: Pill count.	No difference at 4M (F(1,36)=3.32, p<0.07) / No impact.
						Feasibility: Completion rate.	99% completion rate / Highly feasible.
**SMS**	Kassaye 2016	RCT.	HIV+ pregnant women, KEN n=280 (Int) n=270 (Ctrl)	HIV	3 to 6 weekly SMS (ART reminders, motivational, PMTCT, child health & nutrition).	ART in TNPs: Self-report.	Nonsignificant difference in adherence to ART at 34-36W gestation between the 2 groups (97.3% vs 99.6%, aRR= 1.25 (0.43-3.60)./No impact. Nonsignificant difference in adherence to ART at delivery between the 2 groups (94.7% vs 100%, aRR=1.01 (0.88-1.16))./ No impact.
**PC**	Kebaya 2014	RCT	HIV+ mothers in PMTCT, KEN. n=75(Int) n=75(Ctrl)	HIV	Bi-weekly PC.	ART in TNPs: Self-report.	Increased adherence (90.7% vs. 72%, p=0.005) / Effective.
						Feasibility: Retention rate.	More likely to remain in treatment at 10W (69.3% vs 37.3%, p<0.001) / Moderately feasible.
**SMS**	Lester 2010	RCT	HIV+ 18+ yrs, KEN. n=273(Int) n=265(Ctrl)	HIV	Weekly SMS.	ART in TNPs: Self-report.	Improved adherence at 6M and 12M: RR=0.81(0.69-0.94) p=0.006 / Effective.
						ART in TNPs: VL.	Lower virological failure (RR=0.84(0.71-0.99) p=0.04) and improved viral suppression (OR=0.71(0.5-1.01) p=0.058) / Effective.
**SMS + PC + cash incentives**	Maduka 2013	RCT	HIV+ at hospital 20+ yrs, NGA. n=52(Int) n=52(Ctrl)	HIV	2 monthly counselling PCs + 2 weekly SMS+ cash incentives	ART in NAPs: Self-report.	Increased adherence (76.9% vs. 55.8%, X2=5.211,p=0.022; RR=0.725(0.55-0.96)) / Effective.
						ART in NAPs: CD4 count.	Improved CD4+ count (193-->575 cells/mL vs. 131-->361.5 cells/mL; p=0.007) / Effective.
**SMS + PC**	Mbuagbaw 2012	RCT	HIV+ 21+ yrs, CMR. n=101(Int) n=99(Ctrl)	HIV	Weekly motivational SMS. Phone number to call for support.	ART in PVLA: Self-report.	No difference. (RR=1.06(0.89-1.29); p=0.542) / No impact.
						ART in PVLA: Pharmacy Refills.	No difference at 6 months (MD=0.1(-0.23-0.43); p=0.617) / No impact.
						Acceptability: Self-report.	91.1% believed SMS reminders helped; 65% were satisfied; 81.2% would recommend to a friend / Highly acceptable.

For peer review only

For peer review only - http://bmjopen.bmj.com/site/about/guidelines.xhtml

**SMS**	Moore 2015	RCT	HIV+ bipolar 18+ yrs, USA. n=25(Int) n=25(Ctrl)	HIV	SMS reminders.	ART in PVLA: MEM.	No difference. (86.2% (SD= 12.7) vs. 84.8% (SD= 18.1); p=0.95; d=0.01) / No impact.
**SMS**	Nsagha 2016	RCT.	HIV+ on ART, 18+yrs, CMR n=45 (Int) n=45 (Ctrl)	HIV	4 weekly educative SMS over 1M.	ART in PVLA: Self-report.	Nonsignificant difference in adherence to ART at 1M between the 2 groups (64.4% vs 44.2%, p=0.056)/ No impact.
						Acceptability: Self-report.	57.8% wished the SMS to continue/ Moderately acceptable
**SMS**	Pop-Eleches 2010	RCT	HIV+ 18+ yrs, rural KEN. n=142(Daily SMS) n=147(Weekly SMS) n=139(Ctrl)	HIV	Daily or weekly SMS.	ART in PVLA: MEM.	Increased adherence in weekly SMS group over 48W (53% vs. 40% p=0.03) / Effective.
						ART in PVLA: MEM.	No difference between daily SMS group and Ctrl (41% vs. 40% p=0.92) / No impact.
**SMS + cash incentive**	Rana 2016	UnCtrlled trial.	HIV+, 18+yrs, USA. n=32	HIV	Bi-directional weekly SMS appointment reminders, daily ART reminder & supportive messages.	ART in PVLA: Undetectable VL	Significant increase in the number of participants with undetectable VL at 6M (25 vs. 18, p=0.002)/ Effective.
						ATT treatment: Attendance rate.	20/32 completed all visits within 6M study period.
**SMS**	Sabin 2015	RCT	HIV+ 18+ yrs, in CHN. n=63(Int) n=56(Ctrl)	HIV	SMS reminders via MEM + adherence counselling.	ART in PVLA: MEM.	Increased adherence over 6M (82% vs. 51.8%; RR=1.59(1.21- 2.10), p<0.001) / Effective.
						ART in PVLA: VL.	No difference in undetectable VL (93.6% vs. 98.2%, p=0.218) / No impact.
						ART in PVLA: CD4 count.	Higher mean change in CD4 count (52 vs 28 cell/µL, p=0.297) / No impact.
**PC + MMS.**	Shet 2014	RCT	HIV+ 18-60 yrs, IND. n=315(Int) n=316(Ctrl)	HIV	Weekly automated motivational voice call, followed by weekly MMS.	ART in TNPs: VL.	No difference. (Number of virological failures: 15.6% vs. 15.5%. Time to virological failure: aHR= 0.96(0.65-1.43), p= 0.85) / No impact.
						ART in TNPs: Pill count.	No difference. (27% vs. 21.7%; aIRR=1.24(0.94-1.63), p=0.13) / No impact.
						Feasibility: PC received.	86% of calls received by patients / Highly feasible.
**SMS**	Walsh 2012	UnCtrlled trial.	HIV+ Adults on ART, GBR. n=14	HIV	Pill-box w/ MEM + weekly SMS wrt med taking + up to 3 late dose SMS reminders.	ART in APs: Self-report + MEM.	99.5% baseline adherence, 98% at 24W. No difference in missed doses (4.8% in 0-12W; 6.3% in 13-24W)
						Acceptability: Self-report.	64% satisfied, 50% found SMS & system useful. 55% found reminders irritating / Moderately acceptable.
**SMS**	Lim 2008	Quasi-experimental: HxCtrl.	STI clinic, NZL. n=293(Int) n=303(HxCtrl)	CT	SMS to contact clinic for CT test result.	TAT: Time from test to treat.	No change in median time to treat (3 days vs. 4 days, t = - 1.3, p<0.1) / No impact.
**SMS**	Menon-Johansson 2006	Quasi-experimental.	At clinic w/untreated CT, GBR. n=28(Int) n=21(Ctrl)	CT	SMS to contact clinic for CT test result.	TAT: Time from test to diagnosis & test to treat.	Shorter mean time to diagnosis. (7.9 days vs. 12.5; p<0.001) Shorter median time to treat. (8.5 days vs. 15; p=0.005) / Effective.
**SMS+PC**	Barnabas 2016	RCT	16-49 yrs,, ZAF & UGA. n=284(Int) n=224(Ctrl)	HIV	SMS promoting male circumcision 3W, 6-7W after tested negative. Follow-up phone call 1M & 2M following SMS reminders.	PB: Self-report.	Significant difference in reaching outcome at 3M (Intervention vs clinic referral); 48% (RR=1.72 95% CI 1.36-2.17, p values < 0.0001) in SMS reminder group and 47% (RR=1.67, 95%CI 1.29-2.14, p value = 0.0001) in lay counsellor follow-up achieved MC at 3M / Effective
**SMS + MMS.**	Cornelius 2013	UnCtrlled trial.	African-Americans age 13-18, USA, n=40	HIV	HIV-prevention SMS + knowledge question for	PB: Self-report.	Improved condom attitudes & HIV knowledge (83% vs.78% correct answers) / No impact.

For peer review only

					3W.	Acceptability: Self-report.	97% satisfied w/ number of SMS. 86% reported SMS not interfering w/ daily activities/ Highly acceptable.
						Feasibility: Completion rate.	100% at pretest; 90% at 3M FU/ Highly feasible.
**PC**	DiClemente 2014	RCT	High-risk African-American women 14-20 yrs, USA. n=342(Int) n=359(Ctrl)	CT	PC w/ prevention messages every 8W.	PB: % diagnosed w/ CT or GC.	Fewer participants diagnosed w/ CT & GC (90 vs. 104; RR = 0.5 (0.28-0.88), p=0.02. 48 vs. 54; RR = 0.4 (0.15-1.02), p=0.06) / Effective.
						PB: Self-report.	Higher condom use (MD=0.08(0.06 to 0.10) p=0.04) / Effective.
**SMS + cash incentive**	Juzang 2011	Non-randomized Ctrlled trial.	African-American men 16-20 yrs, USA. n=30/group	HIV	3 weekly SMS HIV prevention messages + $40 for completion.	PB: Self-report.	No statistical difference in % of protected sex. Higher awareness of sexual health / No impact.
						Feasibility: Retention rate.	20 (67%) retained in Ctrl & 19 (63%) in SMS group after 2nd FU / Moderately feasible.
**SMS**	Odeny 2014	RCT	Circumcised male at clinic, 18+ yrs, KE. n=600(Int) n=600(Ctrl)	HIV	Daily SMS for 1wk + SMS on days 8, 14, 21, 28, 35, 41, and 42 post-procedure.	PB: Self-report.	Abstention of sexual activity before 42-day follow up: 139/491(28.3%) vs. 124/493(25.2%) in control group (RR=1.13(0.91-1.38), p=0.3)/ No impact.
**SMS**	Reback 2015	UnCtrlled trial.	MSM drug users 18-65 yrs, USA. n=52	HIV	Daily SMS for 2W to reduce risky sexual behaviours.	PB: Self-report.	Reduction in anal sex (6.9 vs. 2.6, t97=2.82, p<0.05) and unprotected anal sex (1.8 vs. 0.5, t97=2.19, p<0.05) in past 2M/ Effective.
**PC**	Belzer 2015	RCT	HIV+ 12-29 yrs, USA. n=19(Int) n=18(Ctrl)	HIV	PC 1hr from time to take medication.	Acceptability: Self-report.	94% satisfied w/ call length and 81% would continue receiving calls / Highly acceptable.
						Feasibility: Retention rate.	63% retention rate / Moderately feasible.
**SMS**	Dean 2012	Feasibility study.	HIV+ at antenatal clinics, ZAF. n=7	HIV	SMS support group+ inquiries answered by physicians.	Acceptability: Self-report.	Overall satisfaction.
						Feasibility: Self-report.	SMS easily kept confidential.
**SMS**	Roth 2014	Feasibility study.	Sex workers 18+ yrs, USA. n=26	HIV	Cell phone diaries to collect info about sexual events.	Acceptability: Self-report.	Cell-phone electronic dairies to collect sensitive information acceptable (84.6%)/ Highly acceptable.
						Feasibility: Completion rate.	90.3% surveys completed / Highly feasible.
**SMS**	Georgette 2016	Feasibility study.	≥18yrs, HIV+, ZAF. n=88	HIV	Weekly SMS reminders to increase ART adherence and appointment reminders	Acceptability: Self-report.	92% would recommend SMS program to a friend, 90.9% said frequency of SMS was just right, 2/88 felt the SMS program slightly violated their privacy. 97.7% reported it helped them remember to take medication. 77.3% agreed that it helped them remember appointments. / Highly acceptable
**SMS**	Reid 2014	Cross-sectional study.	HIV+, BWA. n=42(Int) n=41(Ctrl)	HIV	SMS ARV pick-up reminder.	Acceptability: Self-report.	SMS helpful 93% (Int) vs. 58% (Ctrl) (p<0.001). SMS may lead to serostatus disclosure 10% vs. 56% (p<0.001). 95% satisfied w/ appointment scheduling. 90% would continue receiving SMS / Highly acceptable.
**PC**	Bauermeister 2014	Feasibility study.	MSM 18-30, USA. n=124	HIV	IVRS: microbicide use.	Feasibility: Self-report.	75.5% reported no problems using IVRS / Highly feasible.
**SMS + MMS.**	Cornelius 2011	Feasibility study.	African-Americans age 13-18, USA. n=12	HIV	HIV-prevention SMS+knowledge question for 3W.	Feasibility: Response rate.	80% response rate/ Highly feasible.

Note: Int= intervention; Ctrl= control; HxCtrl= historical control; PB= preventative behaviors (i.e. risk reduction); PN= partner notification; TAT= turnaround time; ATT= attendance rate; ART= ART adherence; NAPs= non-adherent patients; AP= adherent patients; PVLA= Patients with various levels of adherence; TNPs= Treatment naive patients; VL= viral load; CD4= CD4 cell count; PC= phone call; FB= Facebook.

For peer review only - http://bmjopen.bmj.com/site/about/guidelines.xhtml

Appendix 3. Table of studies by innovation (in rows) and by outcomes (in columns)

	Outcome / *Digital Innovation*	*Attendance rate*	*ART adherence*	*Risk reduction*	*Partner notification*	*Turnaround time*	*Self-care*	*Feasibility*†	*Acceptability*†
Number of Studies by Type of Digital Innovation	***mHealth Innovations (SMS/phone call only)***	*30**	*24*	*6*	*0*	*2**	*0*	*5*	*2*
	Internet-based m/eHealth Innovations	*6*	*4*	*5*	*0*	*0*	*1*	*4*	*1*
	Combined innovations	*1*	*1*	*0*	*2*	*0*	*1*	*3*	*1*

*Note: *1 study evaluated both attendance rate and turnaround time and was counted as part of the 30 studies on attendance rate. † studies reporting feasibility and acceptability as secondary outcomes are counted elsewhere in the table depending on primary outcome.*

For peer review only

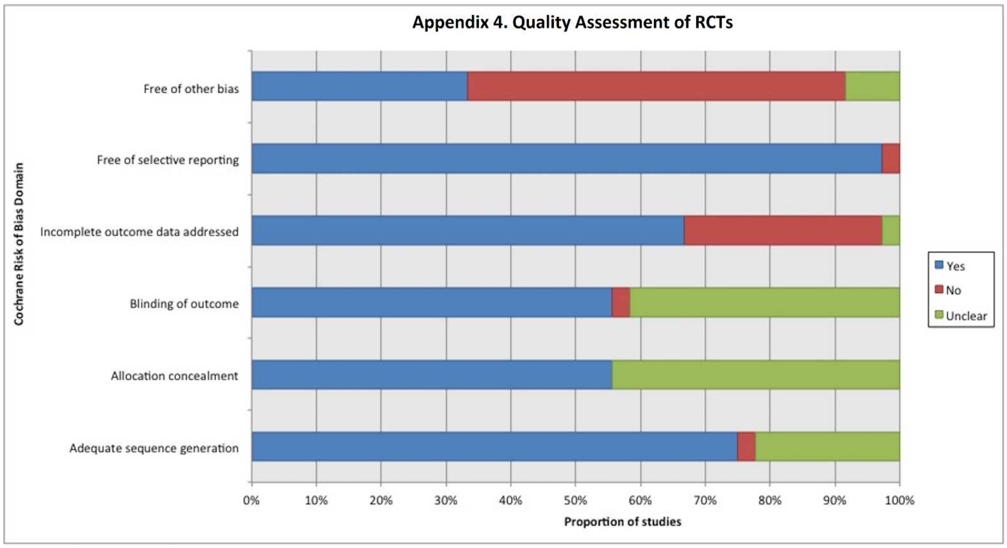

317x171mm (300 x 300 DPI)

review only

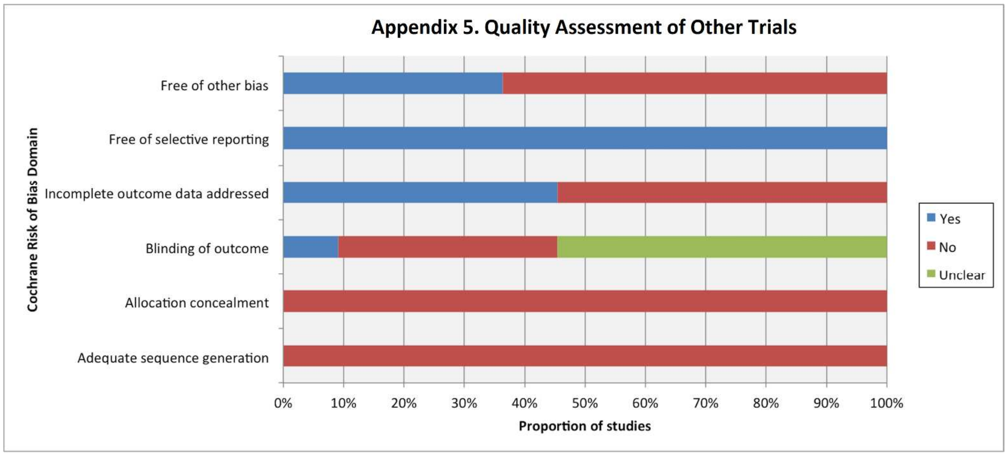

281x126mm (300 x 300 DPI)

For peer review only

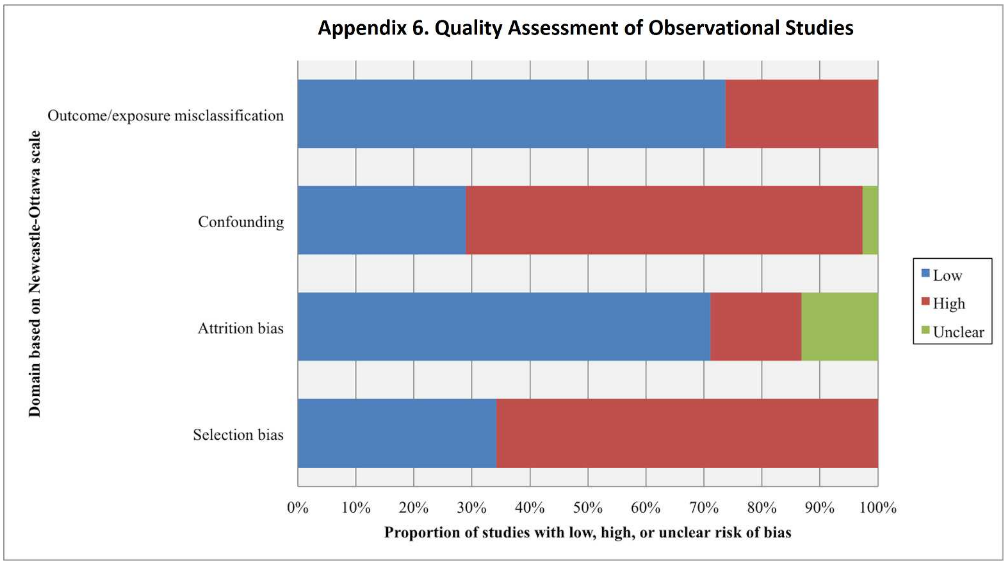

260x145mm (300 x 300 DPI)

review only

PRISMA 2009 Checklist

Section/topic	#	Checklist item	Reported on page #
**TITLE**			
Title	1	Identify the report as a systematic review, meta-analysis, or both.	1
**ABSTRACT**			
Structured summary	2	Provide a structured summary including, as applicable: background; objectives; data sources; study eligibility criteria, participants, and interventions; study appraisal and synthesis methods; results; limitations; conclusions and implications of key findings; systematic review registration number.	2
**INTRODUCTION**			
Rationale	3	Describe the rationale for the review in the context of what is already known.	3
Objectives	4	Provide an explicit statement of questions being addressed with reference to participants, interventions, comparisons, outcomes, and study design (PICOS).	3
**METHODS**			
Protocol and registration	5	Indicate if a review protocol exists, if and where it can be accessed (e.g., Web address), and, if available, provide registration information including registration number.	N/A
Eligibility criteria	6	Specify study characteristics (e.g., PICOS, length of follow-up) and report characteristics (e.g., years considered, language, publication status) used as criteria for eligibility, giving rationale.	4
Information sources	7	Describe all information sources (e.g., databases with dates of coverage, contact with study authors to identify additional studies) in the search and date last searched.	4
Search	8	Present full electronic search strategy for at least one database, including any limits used, such that it could be repeated.	4
Study selection	9	State the process for selecting studies (i.e., screening, eligibility, included in systematic review, and, if applicable, included in the meta-analysis).	4
Data collection process	10	Describe method of data extraction from reports (e.g., piloted forms, independently, in duplicate) and any processes for obtaining and confirming data from investigators.	4
Data items	11	List and define all variables for which data were sought (e.g., PICOS, funding sources) and any assumptions and simplifications made.	4
Risk of bias in individual studies	12	Describe methods used for assessing risk of bias of individual studies (including specification of whether this was done at the study or outcome level), and how this information is to be used in any data synthesis.	5
Summary measures	13	State the principal summary measures (e.g., risk ratio, difference in means).	5
Synthesis of results	14	Describe the methods of handling data and combining results of studies, if done, including measures of consistency (e.g., I^2) for each meta-analysis.	4

For peer review only - http://bmjopen.bmj.com/site/about/guidelines.xhtml

PRISMA 2009 Checklist

Section/topic	#	Checklist item	Reported on page #
Risk of bias across studies	15	Specify any assessment of risk of bias that may affect the cumulative evidence (e.g., publication bias, selective reporting within studies).	5
Additional analyses	16	Describe methods of additional analyses (e.g., sensitivity or subgroup analyses, meta-regression), if done, indicating which were pre-specified.	4
**RESULTS**			
Study selection	17	Give numbers of studies screened, assessed for eligibility, and included in the review, with reasons for exclusions at each stage, ideally with a flow diagram.	5
Study characteristics	18	For each study, present characteristics for which data were extracted (e.g., study size, PICOS, follow-up period) and provide the citations.	Abstraction Table
Risk of bias within studies	19	Present data on risk of bias of each study and, if available, any outcome level assessment (see item 12).	8
Results of individual studies	20	For all outcomes considered (benefits or harms), present, for each study: (a) simple summary data for each intervention group (b) effect estimates and confidence intervals, ideally with a forest plot.	6
Synthesis of results	21	Present results of each meta-analysis done, including confidence intervals and measures of consistency.	6
Risk of bias across studies	22	Present results of any assessment of risk of bias across studies (see Item 15).	8, Supplementary
Additional analysis	23	Give results of additional analyses, if done (e.g., sensitivity or subgroup analyses, meta-regression [see Item 16]).	6
**DISCUSSION**			
Summary of evidence	24	Summarize the main findings including the strength of evidence for each main outcome; consider their relevance to key groups (e.g., healthcare providers, users, and policy makers).	8
Limitations	25	Discuss limitations at study and outcome level (e.g., risk of bias), and at review-level (e.g., incomplete retrieval of identified research, reporting bias).	10
Conclusions	26	Provide a general interpretation of the results in the context of other evidence, and implications for future research.	10
**FUNDING**			
Funding	27	Describe sources of funding for the systematic review and other support (e.g., supply of data); role of funders for the systematic review.	13

From: Moher D, Liberati A, Tetzlaff J, Altman DG, The PRISMA Group (2009). Preferred Reporting Items for Systematic Reviews and Meta-Analyses: The PRISMA Statement. PLoS Med 6(7): e1000097. doi:10.1371/journal.pmed1000097

For more information, visit: www.prisma-statement.org.

For peer review only - http://bmjopen.bmj.com/site/about/guidelines.xhtml

PRISMA 2009 Checklist

For peer review only

BMJ Open

Do Digital Innovations for HIV and Sexually Transmitted Infections work? Results from a Systematic Review (1996-2017).

Journal:	*BMJ Open*
Manuscript ID	bmjopen-2017-017604.R2
Article Type:	Research
Date Submitted by the Author:	20-Sep-2017
Complete List of Authors:	Daher, Jana; Research Institute of the McGill University Health Centre<br>Vijh, Rohit; Research Institute of the McGill University Health Centre<br>Linthwaite , Blake; Research Institute of the McGill University Health Centre<br>Dave, Sailly; Research Institute of the McGill University Health Centre<br>Kim, John; National HIV/AIDS Labs, Public EHalth Agency of Canda<br>Dheda, Keertan; University of Cape Town<br>Peter, Trevor; Clinton Health Access Initiative (CHAI)<br>Pai, Nitika; McGill University, Medicine
**Primary Subject Heading**:	HIV/AIDS
Secondary Subject Heading:	Health services research, Global health
Keywords:	mHealth/eHealth, Innovations, HIV, Sexually transmitted Infections, systematic reviews

SCHOLARONE™
Manuscripts

For peer review only

Do Digital Innovations for HIV and Sexually Transmitted Infections work? Results from a Systematic Review (1996-2017).

Jana Daher[1], MSc (PH), Rohit Vijh[1], BSc, Blake Linthwaite[1], BSc, Sailly Dave[1], BSc,

John Kim[2], PhD, Keertan Dheda[3], MBBCh, PhD, Trevor Peter[4], PhD,

Nitika Pant Pai[1,5], MD PhD

Affiliations:

[1] Division of Clinical Epidemiology, Research Institute of the McGill University Health Centre, Montreal, Canada.

[2] Chief, National HIV/AIDS Labs, National Labs, Winnipeg, Canada.

[3] Professor, Department of Pulmonology, UCT Lung Institute, University of Cape Town, South Africa.

[4] Scientific Director, Diagnostics, Clinton Health Access Initiative (CHAI), Boston, USA.

[1,5] Associate Professor, Department of Medicine, McGill University, Montreal, Canada.

Corresponding author:

nitika.pai@mcgill.ca

Address: 5252 boul de Maisonneuve West, Division of Clinical Epidemiology, McGill University Health Center, Montreal, Quebec, H4A 3S5

Telephone number: 514-934-1934 x44729

Keywords: mHealth/eHealth; Innovations; HIV; Sexually transmitted Infections; systematic reviews, meta-analyses.

For peer review only

ABSTRACT

Objective: Digital innovations with internet/mobile phones offer a potential cost saving solution for overburdened health systems with high service delivery costs to improve efficiency of HIV/STI control initiatives. However, their overall evidence has not yet been appraised. We evaluated the feasibility and impact of all digital innovations for all HIV/STIs.

Design: Systematic review.

Setting/Participants: All settings/all participants.

Intervention: We classified digital innovations into: a) Mobile health-based (mHealth: SMS (short message service)/phone calls), b) Internet-based mobile and/or electronic health (m/eHealth: social media, avatar-guided computer programs, websites, mobile applications, streamed soap opera videos), and c) combined innovations (included both SMS/phone calls and internet-based m/eHealth).

Primary and secondary outcome measures: Feasibility, Acceptability, Impact.

Methods: We searched Databases- MEDLINE via PubMed, Embase, Cochrane CENTRAL, and Web of Science, abstracted data, explored heterogeneity, performed a random effects subgroup analysis.

Results: We reviewed 99 studies, 63 (64%) were from America/Europe, 36 (36%) from Africa/Asia; 79% (79/99) were clinical trials; 84% (83/99) evaluated impact. Of innovations, mHealth-based: 70% (69/99); internet-based: 21% (21/99); combined: 9% (9/99).
All digital innovations were highly accepted (26/31; 84%), feasible (20/31; 65%). Regarding impacted measures: mHealth-based innovations (SMS) significantly improved ART adherence (pooled OR=2.15 [95%CI: 1·18, 3·91]), and clinic attendance rates (pooled OR=1.76 [95%CI: 1·28, 2·42]); Internet-based innovations improved clinic attendance (6/6), ART adherence (4/4), self-care (1/1), while reducing risk (5/5); combined innovations increased clinic attendance, ART adherence, partner notifications, and self-care. Confounding (68%) and selection bias (66%) were observed in observational studies and attrition bias in 31% of clinical trials.

Conclusion: Digital innovations were acceptable, feasible, and generated impact. A trend towards use of internet-based and combined (internet and mobile) innovations was noted. Large scale up studies of high quality, with new integrated impact metrics, and cost effectiveness are needed. Findings will appeal to all stakeholders in the HIV/STI global initiatives space.

For peer review only - http://bmjopen.bmj.com/site/about/guidelines.xhtml

Strengths of the review

- An updated and comprehensive systematic review/meta-analysis of all innovations in HIV/STI.
- Evaluation of study quality with biases, subgroup analyses and sensitivity analyses.
- Evaluation of metrics and measures for objective and subjective data.

Limitations of the review

- Limited data were reported from Sub-Saharan Africa and Southeast Asia (29%, 29/99).
- Limited evidence (18/99, 18%) was available for STIs (other than HIV).
- Limited data on cost effectiveness from high burden settings.
- A lack of integrated online impact metrics to evaluate internet-based eHealth innovations.

For peer review only

For peer review only - http://bmjopen.bmj.com/site/about/guidelines.xhtml

INTRODUCTION

HIV/STI infections remain a public health concern worldwide - a million new HIV/STI infections are acquired every day, with cumulative disease burden exceeding 500 million infections.[1-5] Regarding HIV, countries are working hard to achieve the new UNAIDS 90-90-90 treatment targets;[6] however, structural and societal barriers such as stigma, low socio-economic status, and geographical isolation, impede access to quality care for marginalized populations who are disproportionately impacted by the HIV/AIDS epidemic.[7-8] Likewise, a lack of timely testing and poor retention in care impairs efforts to control HIV/STIs.[7 9-10] To improve early testing, linkage and retention in care, health care systems globally are seeking solutions to improve population engagement, awareness, and education, and efficient care for their hard-to-reach populations. It is imperative to plug gaps in health care service delivery.[11-12] Digital innovations such as electronic health (eHealth), mobile health (mHealth), and combined innovations offer promising solutions to improve health service delivery. eHealth encompasses non-internet and internet-enabled mHealth as well as other internet-based health interventions. These innovations, together with expanded mobile and internet networks, global connectivity, and affordability, present opportunities to change the future landscape of health care service delivery.

The World Bank estimates that globally, 96% of the world's population and 70% of the world's poorest have access to a mobile phone.[13] Of seven billion, two billion (30%) individuals own a smartphone; approximately 50% of mobile phone users access the internet through their phones.[14-15] Technological access has created a portal for social media and other internet-based health interventions.[16] A rapid diffusion of mobile phones and internet technologies are prime drivers of this disruptive phenomenon in health, aptly titled, the creative destruction of medicine.[17] In recent years, visionary foundations (*Grameen*, *Bill and Melinda Gates Foundation*, *UNAIDS*, *Vodafone)* have, with funding, created opportunities for innovative thinking in health. To date, ninety-five countries have evaluated some digital health innovations.[11] This is most evident in under-resourced settings where low-cost and sustainable solutions are needed to solve complex global health challenges.[18]

Digital innovations were first used in non-communicable diseases and later became popular in infectious disease.[19] In the field of HIV/STIs, a *Lancet* study demonstrated the effectiveness of mHealth-based SMS innovations on adherence to antiretroviral therapy (ART).[20] As novel digital innovations and strategies continue to be developed and tested, many smaller reviews and systematic reviews were published. However, a vast majority of these reviews only evaluated a single innovation (e.g. SMS, social media), one or two outcomes, and restricted exploration in select sub-groups (people living with HIV (PLHIV), pregnant women, adolescents, men who have sex with men (MSM)).[21-27] These reviews failed to provide a comprehensive summary of all innovations for program planning and research. Due to a rapid expansion of digital innovations, and an increased popularity of combined innovations (2013-), a need for a comprehensive up-to-date synthesis on all innovations for HIV/STIs was felt.

Our primary objective was to generate a high quality overview/systematic review that summarizes all digital innovations across all populations and outcomes in HIV/STIs. Our

For peer review only - http://bmjopen.bmj.com/site/about/guidelines.xhtml

secondary objective was to inform researchers, policy makers, funders with evidence for their decisions on implementation and scale-up.[11]

METHODS

PRISMA (Preferred Reporting Items for Systematic Reviews and Meta-Analyses) and Cochrane guidelines were followed.[28]

Data Sources and Searches
We searched MEDLINE via PubMed, Embase, Cochrane CENTRAL, and Web of Science for a 21-year period from Feb 1996 up to March 2017, with no language restrictions.

Search Strategy
Keywords used were HIV, AIDS, STI, mhealth, mobile health, ehealth, telemedicine, mobile applications and social media. For a full search strategy, please refer to Appendix 1. (#1 ("HIV" [MeSH] OR "acquired immunodeficiency syndrome" [tiab]), OR #2 (sexually transmitted infections [mh] OR sexually transmitted disease* [tiab]), AND #3 ("mHealth" [tiab] OR "mobile health" [tiab] OR short messag* [tiab] OR "eHealth" [MeSH] OR "telemedicine" [MeSH] OR social medi* [tiab] OR "mobile applications" [tiab]).

Study Selection
Two reviewers independently screened and evaluated citations for eligibility (JD & RV) and two others (BL & SD) independently assessed quality. A senior reviewer was consulted (NPP) for discordance.

Eligibility Criteria
Any clinical trials or observational study designs that evaluated any digital (m/eHealth) technology with any reported outcomes (Refer to Figure 1) were included.

Data Abstraction
Two reviewers (RV, JD) independently abstracted all the data. A pre-piloted data abstraction form, was used to abstract the following items: study design, study population, sample size, digital innovation type, HIV/STIs, outcome measures (e.g. impact, acceptability and feasibility), and metrics (e.g. attendance rate, completion rate, satisfaction) (Refer to Appendix 2). We referred to a previously published framework to define and further classify the following metrics for impact, acceptability, and feasibility.[29]

Subgroup Pooled Analyses
We classified study designs and then classified digital innovations into three groups:[30]

a) mHealth (SMS and phone calls only; i.e. non-internet based);
b) Internet-enabled mHealth and other internet-based eHealth (mobile application, website, online campaign, streamed soap opera videos, avatar-guided computer programs);
c) Combined innovations (innovations that combined both mHealth (SMS/phone calls) with internet enabled m/eHealth).

For peer review only - http://bmjopen.bmj.com/site/about/guidelines.xhtml

Only one subgroup reported similar outcomes which could be pooled, SMS and phone calls, for the following outcomes: a) clinic attendance with SMS; and b) ART adherence via Medication Event Monitoring System (MEMS) caps, with SMS. We pooled these outcomes using a random effects subgroup analysis. Given the diversity in the sample populations between studies, we used the random effect meta-analysis model with DerSimonian and Laird estimator (moments method) of the between-study variance to calculate the pooled effect. We generated forest plots for visual representation of heterogeneity and pooled odds ratios (OR) with 95% confidence intervals (CI). We performed all statistical analyses using Stata/IC, version 13 (StataCorp, College Station, Texas USA).[31]

Narrative Analysis
We narratively described all other data using as follows:

Digital innovations were classified into the following groups based on the strength of evidence: high/strong evidence (metrics at 75-100%), moderate evidence (51-74%), and low/weak evidence (50% or less).

Acceptability: Acceptability was defined as the receptivity in using digital innovations.

Feasibility: Feasibility was defined as the perceived convenience in using digital innovations. It was reported with various metrics: completion, retention, response and referral rates.

Impact: Impact was defined as a statistically significant improvement in measured outcomes compared to a comparator group (i.e. control group or baseline observations). The metrics used to evaluated impact were: A) attendance rate, B) ART adherence, C) risk reduction, D) self-care and E) partner notification. Impact measures were evaluated on two criteria: effect size and precision. Effect size was assessed when data on a comparator group was made available. Precision of the effect estimate was assessed whenever reported, as it reflects the variance or spread of results.

Quality Assessment
We assessed study quality for both clinical trials and observational studies. We used the Cochrane Risk of Bias Tool for trials, and Newcastle-Ottawa quality assessment scale for observational studies.

RESULTS

Of 4252 citations identified through our extensive search, 792 were selected for full-text screening, and 99 citations met our inclusion criteria and were included in this review for evidence synthesis (Refer: Figure 1).

Study characteristics
By geographical location, 37% (37/99) of studies were conducted in North America, 26% (26/99) in Sub-Saharan Africa, 24% (24/99) in Europe, 7% (7/99) in Oceania, 3% (3/99) in Southeast Asia, and 2% (2/99) in South America.

For peer review only - http://bmjopen.bmj.com/site/about/guidelines.xhtml

By study design, the majority were trials: 38% (38/99) were RCTs, 16% (16/99) uncontrolled trials, and 1% (1/99) non-randomised controlled trials. Others included quasi-experimental studies, of which many used historical controls (24%, 24/99), and observational studies (i.e. cross-sectional and feasibility studies) (20%, 20/99).

HIV was the most frequently reported infection (74%, 73/99 studies), followed by chlamydia/gonorrhea (CT/GC) (10%, 10/99). Combinations of HIV with STIs (e.g., syphilis) (8%, 8/99), human papillomavirus (HPV) (4%, 4/99) and hepatitis A/B/C (HBV) (4%, 4/99) were also reported.

In terms of study populations, people living with HIV were prominent across studies (42%, 42/99) followed by other high-risk groups (i.e. MSM/bisexual men, drug users, pregnant women/mother-infant pairs, African-Americans, sex workers, and visible minorities) (28%, 28/99), general clinic attendees (16%, 16/99), CT/ HBV infected individuals (4%, 4/99), and residents of a specific area (9%, 9/99).

Innovations

Digital innovations were documented across the spectrum.

mHealth innovations (SMS/phone calls only) were evaluated in 70% (69/99) of studies.[20 32-99] 72% (50/69) were SMS-based and 28% (19/69) used phone calls or a combination of both (Refer to Figure 2 and Appendix 3).

Internet-enabled mHealth and other internet-based eHealth innovations were evaluated in 21% (21/99) of studies.[100-120] These innovations consisted of many different forms: social media and online campaigns (9/21), avatar-guided computer programs (2/21), mobile applications (5/21), combination of social media and websites (2/21), websites (1/21), telemedicine services (1/21) and streamed soap opera videos (1/21) (Refer to Figure 2 and Appendix 3).

Combined innovations were evaluated in 9% (9/99) of studies.[121-129] Innovations consisted of: SMS + websites/ interactive websites (4/9), SMS + mobile application (3/9) and SMS + social media (including online campaigns) (2/9). (Refer to Figure 2 and Appendix 3).

Measures and Metrics

A vast majority (84%, 83/99) of studies focused on impact measure and metrics, while about 12% (12/99) focused only on feasibility, and the remaining 4% (4/99) on acceptability. Within impact measures, metrics such as clinic attendance rates were reported in 45% (37/83) of studies, followed by ART adherence at 35% (29/83), HIV/STIs risk reduction behaviors at 13% (11/83), turnaround time from testing to treatment at 2% (2/83), partner notification at 2% (2/83), and self-care at 2% (2/83).

Analyses:

Subgroup Pooled Analyses

It was possible to perform subgroup analyses on outcomes that were consistently documented: clinic attendance in 14 quasi-experimental studies that used SMS reminders and MEMS-based ART adherence in 4 RCTs evaluating SMS. The pooled estimate for the impact of SMS reminders on attendance rates was 1.76 [95%CI: 1·28, 2·42] (Refer to Figure 3A). The pooled

For peer review only - http://bmjopen.bmj.com/site/about/guidelines.xhtml

estimate for the impact of SMS on ART adherence tracked via MEMS caps was also significant, OR= 2.15 [95%CI: 1·18, 3·91] (Refer to Figure 3B).[32,47-48]

Narrative Analysis

Impact

Non-internet based mHealth (SMS/PC only)

Of 69 studies, positive results were reported for the following outcomes: clinic attendance (63%, 19/30 studies, of which 84% reached statistical significance); ART adherence (63%, 15/24 studies, of which 93% reached statistical significance); turnaround time from testing to treatment (67%, 2/3 studies). However, SMS reported a limited effect on risk reduction behaviors (3/7, 43%).

Internet-based m/eHealth:

Studies evaluating internet-based eHealth innovations (21/99) reported results that were largely in favor of the following innovations: social media-based interventions for clinic attendance; avatar-guided and mobile applications for ART adherence; social media, avatar, and soap opera videos for risk reduction behaviors; mobile app for self-care.

Social media contributed to higher testing uptake rates in all studies (6/6, 100%). A social media-based campaign increased HIV testing by 252% (n= 1500; 19% from baseline 5.4%, p<0·01) and Syphilis testing by 248% (18·8% from baseline 5·4%, p<0·01), while another campaign increased HIV testing by 52% compared to control (n=625; 63.7% vs. 42% in controls, OR=2.9 [95%CI: 1.8-4.7]).[100,115] Four campaigns guaranteed rapid in-home HIV testing for all those who requested it online.[100-101, 108, 111, 116]

Avatar-guided programs and mobile applications improved ART adherence in all studies (4/4). Statistically significant outcomes were reported in 2/4 programs (50%). These were: a) A personalized avatar-guided computer program improved adherence (n=240; p=0·046); b) a mobile application with immunosuppression graphs and medication reminders lowered viral load (n= 28; p=0·023) and improved adherence (p=0·03) as well.[102,104] In the other two studies, an avatar-guided program improved viral suppression and a mobile application allowed for 100% adherence, but these were underpowered to detect a significant effect (n=76 and n=28, respectively).[107,110]

Social media, avatar and soap opera videos were successful at reducing risky sexual behavior in all the reported studies (5/5). However, significant results were reported in only 3/5 studies: a) Social media-based interventions decreased unprotected sex acts by 65% (n=31; 3·11 vs. baseline 8·96, p=0·042); b) soap opera videos on HIV prevention reduced condomless sex by 78% (n=117; 78% reduction from baseline, p<0·001);[103,106]c) An avatar-guided computer program also lowered the odds of HIV transmission (n=240; OR= 0·46, p=0·012).[102-103,106] Even in two underpowered studies, social media-based interventions led to 40% and 67% higher condom uptake (n=70 and n=50, respectively).[105,117]

For peer review only - http://bmjopen.bmj.com/site/about/guidelines.xhtml

A mobile application increased self-care in the sole study in this category (1/1). A significantly higher self-care performance among chronic HBV-infected individuals was reported compared to controls (n=53; p=0.001).[112]

Combined innovations:

Studies evaluating combined innovations (9/99) showed success of social media + SMS in increasing clinic attendance and partner notification; interactive websites + SMS in improving ART adherence; and mobile app + SMS in increasing self-care. Among the five impact studies, 80% reported a favorable outcome. An online campaign with SMS services increased CT, GC, and HIV tests uptake by 41%, 91%, and 190%, respectively;[123] an interactive website with SMS reminders improved ART adherence in drug-users (n=20; p=0·02);[121] a social media-based partner notification with SMS increased notified contacts by 144% (63.5% in 2011 from baseline 26% in 2010);[126] and a mobile app with SMS significantly improved self-care performance in HIV-infected individuals compared to baseline (n=19; p=0.002).[129]

Acceptability and Feasibility

Overall, across studies that assessed acceptability/feasibility, digital innovations were found to be highly acceptable and feasible (75%-100%) in 26/31 and 20/31 studies, respectively. mHealth innovations (SMS/PC only) were highly acceptable and feasible in 81% (13/16) and 75% (12/16) of studies, respectively.

Internet-based m/eHealth innovations were highly acceptable and feasible in 92% (11/12) and 45% (5/11) of studies, respectively. All included innovations (i.e. avatar, mobile app, social media and streamed videos) were highly acceptable.[102-104,-106-107] While avatar-guided programs were rated high on feasibility, social media-based strategies were found to be less feasible[101-103]

Combined innovations were highly acceptable and feasible in 67% (2/3) and 75% (3/4) of studies, respectively.[121,124] The innovations that were rated high involved a combination of SMS and interactive websites.

Quality

Studies were individually evaluated on quality criteria and biases were noted. Across trials, losses to follow-up were reported in 31% of RCTs and 55% of quasi-trials. Additionally, biases (i.e. misclassification, recall bias) were of concern in 58% of the RCTs and 64% of quasi randomized trials (Refer to Appendix 4 & 5).

In observational studies, confounding (68%) and selection bias (66%) were observed. (Refer to Appendix 6). Studies with small sample sizes, low power or insufficient follow-up time (e.g. 3 weeks or less) sometimes provided contradictory results when objective and subjective metrics evaluated the same outcome.

DISCUSSION

Summary of findings

For peer review only - http://bmjopen.bmj.com/site/about/guidelines.xhtml

Overall, digital innovations reported positive effects on key metrics. We noted a strong positive effect of digital innovations on clinic attendance rates (70%; 26/37), ART adherence (69%; 20/29), risk reduction behaviors (67%; 8/12) and self-care (100%; 2/2). SMS/phone calls were not able to reduce risky sexual behaviours; however social-media based interventions, particularly interactive social media, were effective in reducing risky sexual behaviors. Acceptability was found to be high for all innovations. Feasibility estimates also remained high for all innovations, except for social media-based interventions, possibly due to a perceived lack of privacy and confidentiality. Combined innovations may thus offer promise in plugging this feasibility gap, with internet-based innovations compensating for limitations in SMS-only strategies and vice versa.

While mHealth (SMS/phone calls only) innovations were highly effective in improving clinic attendance, ART adherence, and turnaround time from testing to treatment, they did not report on other outcomes. It should be noted that SMS and phone calls alone failed to reduce risky sexual behaviors, which was not surprising as it is challenging to reduce risky behaviors with a prescriptive SMS alone. Population engagement is essential for risk reduction through qualitative research.

While internet-based m/eHealth innovations (social media, avatar-guided computer programs, mobile apps, and soap opera videos) demonstrated positive evidence on impact metrics, not all studies reached statistical significance. Those that failed to report a statistically significant improvement in ART adherence had small sample sizes and were underpowered to detect these outcomes (n=76 vs. n=240), and had less frequent sessions over a shorter evaluation period (2 sessions over 6 months vs. 4 sessions over 9 months).[102 107] For mobile applications, studies which reported significant effects recruited participants with varying level of adherence,[104 110] compared with studies which had high adherence at baseline (≥ 95%) and did not show significance (due to smaller changes in effect). For social media-based campaigns, the two studies that did not reach statistical significance in reducing risky sexual behaviors lacked an interactive component and simply displayed educational material, while the study that showed significant effect engaged the participants by allowing them to contact professional cognitive behavioral therapists via live chat sessions.[103 105 117]

In terms of quality, confounding and selection bias were noted in observational and quasi-experimental studies, and loss to follow-up in some trials. Nevertheless, the overall validity of the findings from this review was not threatened by biases, as a large proportion of our data were derived from trials. While clinical trials were generally high quality, observational studies were medium to low quality.

Consistent reporting of metrics was lacking, which prevented a comprehensive meta-analysis. Objectives, end points, metrics, and measures, are equally important in feasibility studies and must be well designed to generate high quality evidence.

Our review is an exhaustive assessment of the role of digital innovations in improving prevention and care for HIV/STIs. Our findings resonate with many smaller systematic reviews, which have separately evaluated individual components of digital innovation, such as SMS-based mHealth.[22-23 130-137] Other systematic reviews evaluating social media-based interventions reported similar findings to ours, in improved testing uptake or improvements in sexual health.[25-27 138-139]

For peer review only - http://bmjopen.bmj.com/site/about/guidelines.xhtml

Our review makes a valuable addition to the growing body of evidence by highlighting the success of other interactive and engaging innovations such as avatar-guided computer programs, mobile apps, streamed soap opera videos, and combined innovations. These integrated innovations and programs are gaining in popularity, because of their power to engage rural and urban audiences at many levels.

Designing combined innovations that complementarity of various media, methods, platforms, and messaging may delivery best results. This complementarity may also encourage participant engagement, to improve prevention and care metrics and measures sustainably over time. Engagement is challenging when only one innovation (e.g. mHealth SMS/phone calls only) is the sole focus, where boredom is likely.

Caveats and implications for future research

There are some caveats to considering design and evaluation of innovations. Future research needs to be focussed on tailoring innovations to the context and population, and program objectives. Innovations aiming to reduce risky sexual behaviors could be interactive and tailored to the setting and population, with a deep understanding of patients' needs and preferences.[137 140-141] Any communication with patients could be customized for timing to avoid fatigue with its uptake. For example, patients may be more responsive to weekly versus daily SMS ART reminders.[32 142]

Study quality is essential to generating meaningful results. Large and representative samples of the underlying population and sound statistical techniques during data analysis or sampling methodology, can minimize selection bias. Exploring reasons for differential losses to follow-up could inform future studies. Wherever possible, a control group should be included to differentiate Hawthorne effect from the effect of the intervention.[143] Trials and impact designs can prevent or reduce confounding. Following checklists, like the one by the WHO mHealth Technical Evidence Review Group on mHealth innovations, is suggested and encouraged.[144]

Objective measures (e.g. HIV/STIs diagnosis, VL load) are desired in reporting of quantitative outcomes, over subjective self-reported data (e.g. condom use, self-reported adherence). This could potentially reduce some biases (misclassification biases/ or, desirability/recall biases) that are observed with subjective reporting.

Qualitative data are rich and complement the understanding of all the contextual and population needs, and capture the dynamics of sustainability and change. They need to be integrated with quantitative data to provide a holistic picture of uptake of any digital innovation.

Quality of digital data will merit from an improvement. Across studies, a lack of integrated online impact metrics in evaluating the success of innovations was evident. With continuously evolving digital media, inventing new ways to evaluate acceptability and feasibility becomes necessary. For example, some studies tracked online metrics via Google analytics.[74 100-101 121-124] Synergy with industry powered metrics could be a new wave to measure success of digital innovations.

To scale up proven innovations, a multi-stakeholder engagement is necessary. For that, data and metrics that appeal to all sections of stakeholders will be needed. In addition to improving the

For peer review only - http://bmjopen.bmj.com/site/about/guidelines.xhtml

quality of randomized controlled trials and quasi-experimental impact studies, qualitative studies, cost effectiveness studies, usability studies, are also needed.

Implications for policy and practice

In consonance with other systematic reviews, evidence at-scale and over time was scarce.[138] This limits the projection of the long-term sustainability and cost effectiveness of digital innovations. More evidence on scale-up, cost savings and cost-effectiveness from Sub Saharan Africa and Asia is needed. Future investments that incentivize both: the development and evaluation of combined innovations by government and industry alike, and focus on sustainability of digital innovations with public and private partnerships, are urgently needed.

CONCLUSION

To control HIV/STIs globally, we need novel and disruptive innovations that will uniquely impact health outcomes across the spectrum of access, engagement, treatment and retention so as to impact health service delivery. On one hand, mHealth (SMS/phone calls only) and internet-based m/eHealth were found acceptable, feasible and offered complementarity in improving prevention and care of HIV/STIs. On the other hand, when combined, they provided customized and contextualized solutions for hard-to-reach populations.

Innovations need to be proven for impact and cost effectiveness, using a combination of clinical trials, quasi-randomized studies, observational studies, qualitative research studies. Integrating these innovations across various levels of healthcare with clear evaluation, monitoring, and documentation of metrics will facilitate their integration within existing health service delivery models so as to efficiently impact health outcomes over time.

Findings from this comprehensive review will be informative to all stakeholders – innovators, researchers, healthcare practitioners, policy makers and funders – worldwide seeking evidence on integrating and funding innovations, to make the entire spectrum of HIV/STI care.

ACKNOWLEDGEMENT

The authors would like to acknowledge Ms. Megan Smallwood for her assistance in editing the manuscript.

FOOTNOTES

Contributors:

NPP, JD: concept, design.

NPP: data critiquing, write-up, critique, and overall responsibility of the data.

JD: data synthesis, write-up, critiquing.

RV, BL and SD: data synthesis, write-up and critique.

JK, TP and KD: write-up and critique.

For peer review only - http://bmjopen.bmj.com/site/about/guidelines.xhtml

Final version approved by all authors

Competing Interests:

There are no conflicts of interest

Data Sharing:

No additional data are available. This is a systematic review/syntheses of existing studies, therefore all data are reported in the tables.

Funding:

Grand Challenges Canada Transition to Scale; Grant number 0710-05.
FRSQ Salary Award Chercheur-Boursier Junior 2.

Figure Legends

Figure 1. PRISMA Flow Diagram

Figure 2. All Innovations by Outcome Type (font size enlarged)

Figure 3. Sub-Group Analyses

3A. Sub-Group Analysis Pooled OR for Attendance

3B. Sub-Group Analysis Pooled OR for Adherence

Appendices

Appendix 1. Search Strategy

Appendix 2. Abstraction Table

Appendix 3. Table of Studies by Innovation and by Outcomes

Appendix 4. Quality Assessment of RCTs

Appendix 5. Quality Assessment of Other Trials

Appendix 6. Quality Assessment of Observational Studies

For peer review only

For peer review only - http://bmjopen.bmj.com/site/about/guidelines.xhtml

REFERENCES

1. The World Health Organization. Sexually transmitted infections (STIs): Fact sheet N°110: WHO Media Centre; 2015 [updated December 2015. Available from: http://www.who.int/mediacentre/factsheets/fs110/en/ accessed 27 January 2016.
2. The World Health Organization. Hepatitis B: Fact sheet N°204: WHO Media Centre; 2015 [updated July 2015. Available from: http://www.who.int/mediacentre/factsheets/fs204/en/ accessed 27 January 2016.
3. The World health Organization. Hepatitis C: Fact sheet N°164 2015 [updated July 2015. Available from: http://www.who.int/mediacentre/factsheets/fs164/en/ accessed 27 January 2016.
4. The Joint United Nations Programme on HIV/AIDS. Fact Sheet 2015 2015 [Available from: http://www.unaids.org/en/resources/campaigns/HowAIDSchangedeverything/factsheet accessed 17 Decembre 2016.
5. Newman L, Rowley J, Vander Hoorn S, et al. Global Estimates of the Prevalence and Incidence of Four Curable Sexually Transmitted Infections in 2012 Based on Systematic Review and Global Reporting. *PLoS One* 2015;10(12):e0143304. doi: 10.1371/journal.pone.0143304
PONE-D-15-31044 [pii] [published Online First: 2015/12/10]
6. The Joint United Nations Programme on HIV/AIDS. The Gap Report. Geneva: UNAIDS, 2014.
7. Kagee A, Remien RH, Berkman A, et al. Structural barriers to ART adherence in Southern Africa: Challenges and potential ways forward. *Glob Public Health* 2011;6(1):83-97. doi: 922541258 [pii]
10.1080/17441691003796387 [published Online First: 2010/05/29]
8. Pai NP. Multiplexed point-of-care assays for HIV and co-infections for resource constrained settings: a perspective. *Future Microbiol* 2015;10(9):1393-6. doi: 10.2217/fmb.15.77 [published Online First: 2015/09/09]
9. Pai NP, Vadnais C, Denkinger C, et al. Point-of-care testing for infectious diseases: diversity, complexity, and barriers in low- and middle-income countries. *PLoS Med* 2012;9(9):e1001306. doi: 10.1371/journal.pmed.1001306
PMEDICINE-D-12-01227 [pii] [published Online First: 2012/09/14]
10. Rosen S, Fox MP. Retention in HIV care between testing and treatment in sub-Saharan Africa: a systematic review. *PLoS Med* 2011;8(7):e1001056. doi: 10.1371/journal.pmed.1001056
PMEDICINE-D-11-00096 [pii] [published Online First: 2011/08/04]
11. Ryu S. Book Review: mHealth: New Horizons for Health through Mobile Technologies: Based on the Findings of the Second Global Survey on eHealth (Global Observatory for eHealth Series, Volume 3). *Healthcare Informatics research* 2012;18(3):231-33. doi: doi:10.4258/hir.2012.18.3.231
12. Free C, Phillips G, Watson L, et al. The effectiveness of mobile-health technologies to improve health care service delivery processes: a systematic review and meta-analysis. *PLoS Med* 2013;10(1):e1001363. doi: 10.1371/journal.pmed.1001363
PMEDICINE-D-12-00641 [pii] [published Online First: 2013/03/06]
13. The World Bank. Maximizing Mobile: Information and Communication Technologies for Development. . Washington DC: International Bank for Reconstruction and Development The World Bank 2012.
14. eMarketer. Smartphone user penetration as percentage of total global population from 2011 to 2018. : Statistica- The Statistics Portal; 2014 [Available from: http://www.statista.com/statistics/203734/global-smartphone-penetration-per-capita-since-2005/ accessed 19 February 2016.

For peer review only - http://bmjopen.bmj.com/site/about/guidelines.xhtml

15. eMarketer. Mobile phone internet user penetration worldwide from 2014 to 2019. : Statistica- The Statistics Potal; 2014 [Available from: http://www.statista.com/statistics/284202/mobile-phone-internet-user-penetration-worldwide/ accessed 19 February 2016.
16. Oulasvirta A, Rattenbury T, Ma L, et al. Habits make smartphone use more pervasive. *Personal and Ubiquitous Computing* 2012;16(1):105-14.
17. Topol EJ. The creative destruction of medicine: How the digital revolution will create better health care. . New York: Basic Books 2012.
18. Akter S, Ray P. mHealth - an Ultimate Platform to Serve the Unserved. *Yearb Med Inform* 2010:94-100. doi: me10010094 [pii] [published Online First: 2010/10/13]
19. Ali EE, Chew L, Yap KYL. Evolution and current status of mhealth research: A systematic review. *BMJ Innov* 2016;2(1):33-40. doi: 10.1136/bmjinnov-2015-000096
20. Lester RT, Ritvo P, Mills EJ, et al. Effects of a mobile phone short message service on antiretroviral treatment adherence in Kenya (WelTel Kenya1): a randomised trial. *Lancet* 2010;376(9755):1838-45. doi: S0140-6736(10)61997-6 [pii]
10.1016/S0140-6736(10)61997-6 [published Online First: 2010/11/13]
21. Ambia J, Mandala J. A systematic review of interventions to improve prevention of mother-to-child HIV transmission service delivery and promote retention. *Journal of the International AIDS Society* 2016;19(1):20309. doi: 10.7448/ias.19.1.20309 [published Online First: 2016/04/09]
22. Conserve DF, Jennings L, Aguiar C, et al. Systematic review of mobile health behavioural interventions to improve uptake of HIV testing for vulnerable and key populations. *J Telemed Telecare* 2017;23(2):347-59. doi: 10.1177/1357633x16639186 [published Online First: 2016/04/09]
23. L'Engle KL, Mangone ER, Parcesepe AM, et al. Mobile Phone Interventions for Adolescent Sexual and Reproductive Health: A Systematic Review. *Pediatrics* 2016;138(3) doi: 10.1542/peds.2016-0884 [published Online First: 2016/08/25]
24. Lima IC, Galvao MT, Alexandre Hde O, et al. Information and communication technologies for adherence to antiretroviral treatment in adults with HIV/AIDS. *Int J Med Inform* 2016;92:54-61. doi: 10.1016/j.ijmedinf.2016.04.013 [published Online First: 2016/06/19]
25. Dunne A, McIntosh J, Mallory D. Adolescents, Sexually Transmitted Infections, and Education Using Social Media: A Review of the Literature. *JNP-J Nurse Pract* 2014;10(6):409-16. doi: 10.1016/j.nurpra.2014.03.020
26. Jones K, Eathington P, Baldwin K, et al. The Impact of Health Education Transmitted Via Social Media or Text Messaging on Adolescent and Young Adult Risky Sexual Behavior: A Systematic Review of the Literature. *Sex Transm Dis* 2014;41(7):413-19. doi: 10.1097/olq.0000000000000146
27. Schnall R, Travers J, Rojas M, et al. eHealth Interventions for HIV Prevention in High-Risk Men Who Have Sex With Men: A Systematic Review. *J Med Internet Res* 2014;16(5):228-37. doi: 10.2196/jmir.3393
28. Moher D, Liberati A, Tetzlaff J, et al. Preferred reporting items for systematic reviews and meta-analyses: the PRISMA statement. *Ann Intern Med* 2009;151(4):264-9, W64. doi: 0000605-200908180-00135 [pii] [published Online First: 2009/07/23]
29. Chiavegatti T, Vijh R, Pant Pai N. Metrics beyond accuracy for HIV point-of-care tests: a systematic review of evidence. *Canadian Journal of Infectious Diseases & Medical Microbiology* 2014;25(Suppl A):90A.
30. Pai M, Filion K. Classification of study designs (Version 8) Montreal: McGill University; 2014 [Available from: http://www.teachepi.org/documents/courses/Classification%20Design.pdf.
31. Harris R, Bradburn M, Deeks J, et al. metan: fixed- and random-effects meta-analysis. *Stata Journal* 2008;8(1):3-28.
32. Pop-Eleches C, Thirumurthy H, Habyarimana JP, et al. Mobile phone technologies improve adherence to antiretroviral treatment in a resource-limited setting: a randomized controlled trial of text

For peer review only - http://bmjopen.bmj.com/site/about/guidelines.xhtml

message reminders. *AIDS* 2011;25(6):825-34. doi: 10.1097/QAD.0b013e32834380c1 [published Online First: 2011/01/22]
33. Dowshen N, Kuhns LM, Johnson A, et al. Improving adherence to antiretroviral therapy for youth living with HIV/AIDS: a pilot study using personalized, interactive, daily text message reminders. *J Med Internet Res* 2012;14(2):e51. doi: v14i2e51 [pii]
10.2196/jmir.2015 [published Online First: 2012/04/07]
34. Menon-Johansson AS, McNaught F, Mandalia S, et al. Texting decreases the time to treatment for genital Chlamydia trachomatis infection. *Sex Transm Infect* 2006;82(1):49-51. doi: 82/1/49 [pii]
10.1136/sti.2004.014381 [published Online First: 2006/02/08]
35. Finocchario-Kessler S, Gautney BJ, Khamadi S, et al. If you text them, they will come: using the HIV infant tracking system to improve early infant diagnosis quality and retention in Kenya. *AIDS* 2014;28 Suppl 3:S313-21. doi: 10.1097/QAD.0000000000000332
00002030-201407001-00011 [pii] [published Online First: 2014/07/06]
36. Lim EJ, Haar J, Morgan J. Can text messaging results reduce time to treatment of Chlamydia trachomatis? *Sex Transm Infect* 2008;84(7):563-4. doi: sti.2008.032441 [pii]
10.1136/sti.2008.032441 [published Online First: 2008/08/30]
37. Bailey S, Scalley B, Gilles M. Text 2 treat - using SMS to recall clients for treatment. *Int J STD AIDS* 2014;25(14):1038-40. doi: 0956462414530108 [pii]
10.1177/0956462414530108 [published Online First: 2014/04/04]
38. Smillie K, Van Borek N, Abaki J, et al. A qualitative study investigating the use of a mobile phone short message service designed to improve HIV adherence and retention in care in Canada (WelTel BC1). *J Assoc Nurses AIDS Care* 2014;25(6):614-25. doi: S1055-3290(14)00040-5 [pii]
10.1016/j.jana.2014.01.008 [published Online First: 2014/04/29]
39. Reback CJ, Fletcher JB, Shoptaw S, et al. Exposure to Theory-Driven Text Messages is Associated with HIV Risk Reduction Among Methamphetamine-Using Men Who have Sex with Men. *AIDS Behav* 2015;19 Suppl 2:130-41. doi: 10.1007/s10461-014-0985-7 [published Online First: 2015/01/08]
40. Evans C, Turner K, Blake H, et al. Text messaging to encourage uptake of HIV testing amongst African communities: Findings from a theory based feasibility study. *HIV Medicine* 2015;16(Suppl 2):54.
41. Dowshen N, Kuhns LM, Gray C, et al. Feasibility of interactive text message response (ITR) as a novel, real-time measure of adherence to antiretroviral therapy for HIV+ youth. *AIDS Behav* 2013;17(6):2237-43. doi: 10.1007/s10461-013-0464-6 [published Online First: 2013/04/03]
42. Cornelius JB, Dmochowski J, Boyer C, et al. Text-messaging-enhanced HIV intervention for African American adolescents: a feasibility study. *J Assoc Nurses AIDS Care* 2013;24(3):256-67. doi: S1055-3290(12)00140-9 [pii]
10.1016/j.jana.2012.06.005 [published Online First: 2012/11/06]
43. Ammassari A, Trotta MP, Mussini C, et al. Mobile phone intervention based on short message system (SMS) improve adherence behavior in HIV-infected non-adherent to antiretroviral therapy. The HI-Vision Study. *Infection* 2010;38(Suppl 52):52.
44. Ammassari A, Trotta MP, Shalev N, et al. Timed short messaging service improves adherence and virological outcomes in HIV-1-infected patients with suboptimal adherence to antiretroviral therapy. *J Acquir Immune Defic Syndr* 2011;58(4):e113-5. doi: 10.1097/QAI.0b013e3182359d2a
00126334-201112010-00015 [pii] [published Online First: 2011/10/29]
45. Segaren N, Lewis T, Desinor O, et al. The distribution and use of cell phones to mothers of HIV-positive infants identified by the Haiti National Early Infant Diagnosis of HIV program (EID): a model for increasing adherence? XIX International AIDS Conference Washington DC, 2012.
46. Sheppard Z, Walsh J, Weston R. The TRxCare™ adherence support system: a pilot study of its acceptability to patients on virologically successful HAART. 18th Annual Conference of British HIV Association Birmingham, 2012.

For peer review only - http://bmjopen.bmj.com/site/about/guidelines.xhtml

47. da Costa TM, Barbosa BJ, Gomes e Costa DA, et al. Results of a randomized controlled trial to assess the effects of a mobile SMS-based intervention on treatment adherence in HIV/AIDS-infected Brazilian women and impressions and satisfaction with respect to incoming messages. *Int J Med Inform* 2012;81(4):257-69. doi: S1386-5056(11)00204-8 [pii]
10.1016/j.ijmedinf.2011.10.002 [published Online First: 2012/02/03]
48. Sabin LL, Bachman DeSilva M, Gill CJ, et al. Improving Adherence to Antiretroviral Therapy With Triggered Real-time Text Message Reminders: The China Adherence Through Technology Study. *J Acquir Immune Defic Syndr* 2015;69(5):551-9. doi: 10.1097/QAI.0000000000000651 [published Online First: 2015/04/19]
49. Brook G, Farmer T, Murphy S, et al. Using SMS text reminders to reduce 'did not attend' (DNA) rates in sexual health and HIV appointment clinics. *Sex Transm Infect* 2013;89(Suppl 1):A1-A428.
50. Kliner M, Knight A, Mamvura C, et al. Using no-cost mobile phone reminders to improve attendance for HIV test results: a pilot study in rural Swaziland. *Infect Dis Poverty* 2013;2(1):12. doi: 2049-9957-2-12 [pii]
10.1186/2049-9957-2-12 [published Online First: 2013/07/16]
51. Farmer T, Brook G, McSorley J, et al. Using short message service text reminders to reduce 'did not attend' rates in sexual health and HIV appointment clinics. *Int J STD AIDS* 2014;25(4):289-93. doi: 0956462413502325 [pii]
10.1177/0956462413502325 [published Online First: 2013/09/04]
52. Brook G, Burton J, McSorley J, et al. The effectiveness of SMS texts for reminding patietns at high risk of STIs and HIV to return for testing. *Sex Transm Infect* 2013;89(Suppl 1):A1-A428.
53. Guy R, Wand H, Knight V, et al. SMS reminders improve re-screening in women and heterosexual men with chlamydia infection at Sydney Sexual Health Centre: a before-and-after study. *Sex Transm Infect* 2013;89(1):11-5. doi: sextrans-2011-050370 [pii]
10.1136/sextrans-2011-050370 [published Online First: 2012/04/21]
54. Burton J, Brook G, McSorley J, et al. The utility of short message service (SMS) texts to remind patients at higher risk of STIs and HIV to reattend for testing: a controlled before and after study. *Sex Transm Infect* 2014;90(1):11-3. doi: sextrans-2013-051228 [pii]
10.1136/sextrans-2013-051228 [published Online First: 2013/09/26]
55. Desai M, Burns F, Mercey D, et al. Active recall of men who have sex with men (MSM) for an HIV/STI testing: a feasible and effective strategy? *HIV Medicine* 2014;15(Suppl 3):17-159.
56. Kharbanda EO, Stockwell MS, Fox HW, et al. Text message reminders to promote human papillomavirus vaccination. *Vaccine* 2011;29(14):2537-41. doi: S0264-410X(11)00125-3 [pii]
10.1016/j.vaccine.2011.01.065 [published Online First: 2011/02/09]
57. Vilella A, Bayas JM, Diaz MT, et al. The role of mobile phones in improving vaccination rates in travelers. *Prev Med* 2004;38(4):503-9. doi: 10.1016/j.ypmed.2003.12.005
S0091743503003475 [pii] [published Online First: 2004/03/17]
58. Downing SG, Cashman C, McNamee H, et al. Increasing chlamydia test of re-infection rates using SMS reminders and incentives. *Sex Transm Infect* 2013;89(1):16-9. doi: sextrans-2011-050454 [pii]
10.1136/sextrans-2011-050454 [published Online First: 2012/06/26]
59. Odeny TA, Bailey RC, Bukusi EA, et al. Text messaging to improve attendance at post-operative clinic visits after adult male circumcision for HIV prevention: a randomized controlled trial. *PLoS One* 2012;7(9):e43832. doi: 10.1371/journal.pone.0043832
PONE-D-12-11992 [pii] [published Online First: 2012/09/08]
60. de Tolly K, Skinner D, Nembaware V, et al. Investigation into the use of short message services to expand uptake of human immunodeficiency virus testing, and whether content and dosage have impact. *Telemed J E Health* 2012;18(1):18-23. doi: 10.1089/tmj.2011.0058 [published Online First: 2011/12/14]

For peer review only - http://bmjopen.bmj.com/site/about/guidelines.xhtml

61. Bigna JJ, Noubiap JJ, Kouanfack C, et al. Effect of mobile phone reminders on follow-up medical care of children exposed to or infected with HIV in Cameroon (MORE CARE): a multicentre, single-blind, factorial, randomised controlled trial. *Lancet Infect Dis* 2014;14(7):600-8. doi: S1473-3099(14)70741-8 [pii]
10.1016/S1473-3099(14)70741-8 [published Online First: 2014/06/17]
62. Rand CM, Brill H, Albertin C, et al. Effectiveness of centralized text message reminders on human papillomavirus immunization coverage for publicly insured adolescents. *J Adolesc Health* 2015;56(5 Suppl):S17-20. doi: S1054-139X(14)00707-1 [pii]
10.1016/j.jadohealth.2014.10.273 [published Online First: 2015/04/13]
63. Matheson EC, Derouin A, Gagliano M, et al. Increasing HPV vaccination series completion rates via text message reminders. *J Pediatr Health Care* 2014;28(4):e35-9. doi: S0891-5245(13)00293-9 [pii]
10.1016/j.pedhc.2013.09.001 [published Online First: 2013/11/10]
64. Schwartz SR, Clouse K, Yende N, et al. Acceptability and Feasibility of a Mobile Phone-Based Case Management Intervention to Retain Mothers and Infants from an Option B+ Program in Postpartum HIV Care. *Matern Child Health J* 2015;19(9):2029-37. doi: 10.1007/s10995-015-1715-0 [published Online First: 2015/02/07]
65. Belzer ME, Naar-King S, Olson J, et al. The use of cell phone support for non-adherent HIV-infected youth and young adults: an initial randomized and controlled intervention trial. *AIDS Behav* 2014;18(4):686-96. doi: 10.1007/s10461-013-0661-3 [published Online First: 2013/11/26]
66. Maduka O, Tobin-West CI. Adherence counseling and reminder text messages improve uptake of antiretroviral therapy in a tertiary hospital in Nigeria. *Niger J Clin Pract* 2013;16(3):302-8. doi: NigerJClinPract_2013_16_3_302_113451 [pii]
10.4103/1119-3077.113451 [published Online First: 2013/06/19]
67. Kebaya L, Nduati R, Bashir A. Efficacy of mobile phone use on adherence to nevirapine prophylaxis and retention in care among the HIV-exposed infants in PMTCT: a randomized controlled trial. *Arch Dis Child* 2014;99(Suppl 2):A1-A620.
68. Hardy H, Kumar V, Doros G, et al. Randomized controlled trial of a personalized cellular phone reminder system to enhance adherence to antiretroviral therapy. *AIDS Patient Care STDS* 2011;25(3):153-61. doi: 10.1089/apc.2010.0006 [published Online First: 2011/02/18]
69. DiClemente RJ, Wingood GM, Sales JM, et al. Efficacy of a telephone-delivered sexually transmitted infection/human immunodeficiency virus prevention maintenance intervention for adolescents: a randomized clinical trial. *JAMA Pediatr* 2014;168(10):938-46. doi: 1899236 [pii]
10.1001/jamapediatrics.2014.1436 [published Online First: 2014/08/27]
70. Cornelius JB, Cato M, Lawrence JS, et al. Development and pretesting multimedia HIV-prevention text messages for mobile cell phone delivery. *J Assoc Nurses AIDS Care* 2011;22(5):407-13. doi: S1055-3290(10)00224-4 [pii]
10.1016/j.jana.2010.11.007 [published Online First: 2011/01/25]
71. Dean AL, Makin JD, Kydd AS, et al. A pilot study using interactive SMS support groups to prevent mother-to-child HIV transmission in South Africa. *J Telemed Telecare* 2012;18(7):399-403. doi: jtt.2012.120118 [pii]
10.1258/jtt.2012.120118 [published Online First: 2012/10/05]
72. Juzang I, Fortune T, Black S, et al. A pilot programme using mobile phones for HIV prevention. *J Telemed Telecare* 2011;17(3):150-3. doi: jtt.2010.091107 [pii]
10.1258/jtt.2010.091107 [published Online First: 2011/01/29]
73. Kalichman SC, Kalichman MO, Cherry C, et al. Brief behavioral self-regulation counseling for HIV treatment adherence delivered by cell phone: an initial test of concept trial. *AIDS Patient Care STDS* 2011;25(5):303-10. doi: 10.1089/apc.2010.0367 [published Online First: 2011/04/05]

For peer review only - http://bmjopen.bmj.com/site/about/guidelines.xhtml

74. Mbuagbaw L, Thabane L, Ongolo-Zogo P, et al. The Cameroon Mobile Phone SMS (CAMPS) trial: a randomized trial of text messaging versus usual care for adherence to antiretroviral therapy. *PLoS One* 2012;7(12):e46909. doi: 10.1371/journal.pone.0046909
PONE-D-12-24092 [pii] [published Online First: 2012/12/14]
75. Moore DJ, Poquette A, Casaletto KB, et al. Individualized texting for adherence building (iTAB): improving antiretroviral dose timing among HIV-infected persons with co-occurring bipolar disorder. *AIDS Behav* 2015;19(3):459-71. doi: 10.1007/s10461-014-0971-0 [published Online First: 2014/12/17]
76. Norton BL, Person AK, Castillo C, et al. Barriers to using text message appointment reminders in an HIV clinic. *Telemed J E Health* 2014;20(1):86-9. doi: 10.1089/tmj.2012.0275 [published Online First: 2013/10/29]
77. Odeny TA, Bailey RC, Bukusi EA, et al. Effect of text messaging to deter early resumption of sexual activity after male circumcision for HIV prevention: a randomized controlled trial. *J Acquir Immune Defic Syndr* 2014;65(2):e50-7. doi: 10.1097/QAI.0b013e3182a0a050 [published Online First: 2013/07/13]
78. Bauermeister J, Carballo-Dieguez A, Giguere R, et al. Interactive voice response system (IVRS): data quality considerations and lessons learned during a microbicide placebo adherence trial with young men who have sex with men. *Journal of Adolescent Health* 2014;54(2):S57-S58.
79. Belzer ME, Kolmodin MacDonell K, Clark LF, et al. Acceptability and Feasibility of a Cell Phone Support Intervention for Youth Living with HIV with Nonadherence to Antiretroviral Therapy. *AIDS Patient Care STDS* 2015;29(6):338-45. doi: 10.1089/apc.2014.0282 [published Online First: 2015/05/01]
80. Shet A, De Costa A, Kumarasamy N, et al. Effect of mobile telephone reminders on treatment outcome in HIV: evidence from a randomised controlled trial in India. *BMJ* 2014;349:g5978. [published Online First: 2014/01/01]
81. Reid MJ, Dhar SI, Cary M, et al. Opinions and attitudes of participants in a randomized controlled trial examining the efficacy of SMS reminders to enhance antiretroviral adherence: a cross-sectional survey. *J Acquir Immune Defic Syndr* 2014;65(2):e86-8. doi: 10.1097/QAI.0b013e3182a9c72b
00126334-201402010-00027 [pii] [published Online First: 2014/01/21]
82. Roth AM, Hensel DJ, Fortenberry JD, et al. Feasibility and acceptability of cell phone diaries to measure HIV risk behavior among female sex workers. *AIDS Behav* 2014;18(12):2314-24. doi: 10.1007/s10461-014-0718-y [published Online First: 2014/03/20]
83. Barnabas RV, van Rooyen H, Tumwesigye E, et al. Uptake of antiretroviral therapy and male circumcision after community-based HIV testing and strategies for linkage to care versus standard clinic referral: a multisite, open-label, randomised controlled trial in South Africa and Uganda. *The lancet HIV* 2016;3(5):e212-20. doi: 10.1016/s2352-3018(16)00020-5 [published Online First: 2016/04/30]
84. Bassett IV, Coleman SM, Giddy J, et al. Sizanani: A Randomized Trial of Health System Navigators to Improve Linkage to HIV and TB Care in South Africa. *J Acquir Immune Defic Syndr* 2016;73(2):154-60. doi: 10.1097/qai.0000000000001025 [published Online First: 2016/09/16]
85. Cantudo-Cuenca MR, Manzano-Garcia M, Jimenez-Galan R, et al. Effect of mobile phone short messages on antiretroviral treatment adherence among HIV infected patients. *International Journal of Clinical Pharmacy* 2016;38 (6):517. doi: http://dx.doi.org/10.1007/s11096-015-0240-y
86. Haberer JE, Musiimenta A, Atukunda EC, et al. Short message service (SMS) reminders and real-time adherence monitoring improve antiretroviral therapy adherence in rural Uganda. *AIDS* 2016;30(8):1295-300. doi: 10.1097/qad.0000000000001021 [published Online First: 2016/01/14]
87. Jeffries C, Ross P, Matoff-Stepp S, et al. Ucare4life: Mobile texting to improve HIV care continuum outcomes for minority youth. *Topics in Antiviral Medicine* 2016;24 (E-1):427.

For peer review only - http://bmjopen.bmj.com/site/about/guidelines.xhtml

88. Joseph Davey D, Nhavoto JA, Augusto O, et al. SMSaude: Evaluating Mobile Phone Text Reminders to Improve Retention in HIV Care for Patients on Antiretroviral Therapy in Mozambique. *J Acquir Immune Defic Syndr* 2016;73(2):e23-30. doi: 10.1097/qai.0000000000001115 [published Online First: 2016/09/16]
89. Kampman C, Koedijk F, Driessen-Hulshof H, et al. Retesting young STI clinic visitors with urogenital Chlamydia trachomatis infection in the Netherlands; response to a text message reminder and reinfection rates: a prospective study with historical controls. *Sex Transm Infect* 2016;92(2):124-9. doi: 10.1136/sextrans-2015-052115 [published Online First: 2015/09/26]
90. Kassaye SG, Ong'ech J, Sirengo M, et al. Cluster-Randomized Controlled Study of SMS Text Messages for Prevention of Mother-to-Child Transmission of HIV in Rural Kenya. *AIDS research and treatment* 2016;2016:1289328. doi: 10.1155/2016/1289328 [published Online First: 2017/01/06]
91. McIver R, Dyda A, McNulty AM, et al. Text message reminders do not improve hepatitis B vaccination rates in an Australian sexual health setting. *Journal of the American Medical Informatics Association : JAMIA* 2016;23(e1):e88-92. doi: 10.1093/jamia/ocv145 [published Online First: 2015/10/27]
92. Njuguna N, Ngure K, Mugo N, et al. The Effect of Human Immunodeficiency Virus Prevention and Reproductive Health Text Messages on Human Immunodeficiency Virus Testing Among Young Women in Rural Kenya: A Pilot Study. *Sex Transm Dis* 2016;43(6):353-9. doi: 10.1097/olq.0000000000000450 [published Online First: 2016/05/21]
93. Nsagha DS, Lange I, Fon PN, et al. A Randomized Controlled Trial on the Usefulness of Mobile Text Phone Messages to Improve the Quality of Care of HIV and AIDS Patients in Cameroon. *Open AIDS J* 2016;10:93-103. doi: 10.2174/1874613601610010093 [published Online First: 2016/09/02]
94. Nyatsanza F, McSorley J, Murphy S, et al. 'It's all in the message': the utility of personalised short message service (SMS) texts to remind patients at higher risk of STIs and HIV to reattend for testing-a repeat before and after study. *Sex Transm Infect* 2016;92(5):393-5. doi: 10.1136/sextrans-2015-052216 [published Online First: 2015/12/17]
95. Rana AI, van den Berg JJ, Lamy E, et al. Using a Mobile Health Intervention to Support HIV Treatment Adherence and Retention Among Patients at Risk for Disengaging with Care. *AIDS Patient Care STDS* 2016;30(4):178-84. doi: 10.1089/apc.2016.0025 [published Online First: 2016/03/31]
96. Rand CM, Vincelli P, Goldstein NP, et al. Effects of Phone and Text Message Reminders on Completion of the Human Papillomavirus Vaccine Series. *J Adolesc Health* 2017;60(1):113-19. doi: 10.1016/j.jadohealth.2016.09.011 [published Online First: 2016/11/12]
97. Coleman J, Bohlin KC, Thorson A, et al. Effectiveness of an SMS-based maternal mHealth intervention to improve clinical outcomes of HIV-positive pregnant women. *AIDS care* 2017:1-8. doi: 10.1080/09540121.2017.1280126 [published Online First: 2017/01/22]
98. Garofalo R, Kuhns LM, Hotton A, et al. A Randomized Controlled Trial of Personalized Text Message Reminders to Promote Medication Adherence Among HIV-Positive Adolescents and Young Adults. *AIDS Behav* 2016;20(5):1049-59. doi: 10.1007/s10461-015-1192-x [published Online First: 2015/09/13]
99. Georgette N, Siedner MJ, Zanoni B, et al. The Acceptability and Perceived Usefulness of a Weekly Clinical SMS Program to Promote HIV Antiretroviral Medication Adherence in KwaZulu-Natal, South Africa. *AIDS Behav* 2016;20(11):2629-38. doi: 10.1007/s10461-016-1287-z [published Online First: 2016/10/21]
100. Dowshen N, Lee S, Matty Lehman B, et al. IknowUshould2: Feasibility of a Youth-Driven Social Media Campaign to Promote STI and HIV Testing Among Adolescents in Philadelphia. *AIDS Behav* 2015;19 Suppl 2:106-11. doi: 10.1007/s10461-014-0991-9 [published Online First: 2015/01/08]

For peer review only - http://bmjopen.bmj.com/site/about/guidelines.xhtml

101. Jones M, Aderogba K, Drunis L. Providing rapid HIV testing in their homes for men who have sex with men, recruited via social media. *HIV Medicine* 2015;16(Suppl 2):50-51.
102. Kurth AE, Spielberg F, Cleland CM, et al. Computerized counseling reduces HIV-1 viral load and sexual transmission risk: findings from a randomized controlled trial. *J Acquir Immune Defic Syndr* 2014;65(5):611-20. doi: 10.1097/QAI.0000000000000100 [published Online First: 2014/01/05]
103. Lelutiu-Weinberger C, Pachankis JE, Gamarel KE, et al. Feasibility, Acceptability, and Preliminary Efficacy of a Live-Chat Social Media Intervention to Reduce HIV Risk Among Young Men Who Have Sex With Men. *AIDS Behav* 2015;19(7):1214-27. doi: 10.1007/s10461-014-0911-z [published Online First: 2014/09/27]
104. Perera AI, Thomas MG, Moore JO, et al. Effect of a smartphone application incorporating personalized health-related imagery on adherence to antiretroviral therapy: a randomized clinical trial. *AIDS Patient Care STDS* 2014;28(11):579-86. doi: 10.1089/apc.2014.0156 [published Online First: 2014/10/08]
105. Jones K, Baldwin KA, Lewis PR. The potential influence of a social media intervention on risky sexual behavior and Chlamydia incidence. *J Community Health Nurs* 2012;29(2):106-20. doi: 10.1080/07370016.2012.670579 [published Online First: 2012/04/28]
106. Jones R, Hoover DR, Lacroix LJ. A randomized controlled trial of soap opera videos streamed to smartphones to reduce risk of sexually transmitted human immunodeficiency virus (HIV) in young urban African American women. *Nurs Outlook* 2013;61(4):205-15 e3. doi: S0029-6554(13)00061-4 [pii]
10.1016/j.outlook.2013.03.006 [published Online First: 2013/06/08]
107. Naar-King S, Outlaw AY, Sarr M, et al. Motivational Enhancement System for Adherence (MESA): pilot randomized trial of a brief computer-delivered prevention intervention for youth initiating antiretroviral treatment. *J Pediatr Psychol* 2013;38(6):638-48. doi: jss132 [pii]
10.1093/jpepsy/jss132 [published Online First: 2013/01/30]
108. Elliot E, Rossi M, McCormack S, et al. Identifying undiagnosed HIV in men who have sex with men (MSM) by offering HIV home sampling via online gay social media: a service evaluation. *Sex Transm Infect* 2016;92(6):470-3. doi: 10.1136/sextrans-2015-052090 [published Online First: 2016/05/26]
109. Garett R, Menacho L, Young SD. Ethical Issues in Using Social Media to Deliver an HIV Prevention Intervention: Results from the HOPE Peru Study. *Prevention science : the official journal of the Society for Prevention Research* 2017;18(2):225-32. doi: 10.1007/s11121-016-0739-z [published Online First: 2016/12/10]
110. Himelhoch S, Kreyenbuhl J, Palmer-Bacon J, et al. Pilot feasibility study of Heart2HAART: a smartphone application to assist with adherence among substance users living with HIV. *AIDS care* 2017:1-7. doi: 10.1080/09540121.2016.1259454 [published Online First: 2017/01/04]
111. Huang E, Marlin RW, Young SD, et al. Using Grindr, a Smartphone Social-Networking Application, to Increase HIV Self-Testing Among Black and Latino Men Who Have Sex With Men in Los Angeles, 2014. *AIDS education and prevention : official publication of the International Society for AIDS Education* 2016;28(4):341-50. doi: 10.1521/aeap.2016.28.4.341 [published Online First: 2016/07/20]
112. Jeon JH. Evaluation of a smartphone application for self-care performance of patients with chronic hepatitis B: A randomized controlled trial. *Applied nursing research : ANR* 2016;32:182-89. doi: 10.1016/j.apnr.2016.07.011 [published Online First: 2016/12/15]
113. Polilli E, Sozio F, Di Stefano P, et al. Web-Based HIV Testing in Abruzzo, Italy: Analysis of 15-Month Activity Results. *AIDS Patient Care STDS* 2016;30(10):471-75. doi: 10.1089/apc.2016.0082
114. Przybyla SM, Eliseo-Arras RK, Krawiec G, et al. Feasibility and Acceptability of a Smartphone App for Daily Reports of Substance Use and Antiretroviral Therapy Adherence among HIV-Infected

For peer review only - http://bmjopen.bmj.com/site/about/guidelines.xhtml

Adults. *AIDS research and treatment* 2016;2016:9510172. doi: 10.1155/2016/9510172 [published Online First: 2016/09/10]

115. Rhodes SD, McCoy TP, Tanner AE, et al. Using Social Media to Increase HIV Testing Among Gay and Bisexual Men, Other Men Who Have Sex With Men, and Transgender Persons: Outcomes From a Randomized Community Trial. *Clinical infectious diseases : an official publication of the Infectious Diseases Society of America* 2016;62(11):1450-3. doi: 10.1093/cid/ciw127 [published Online First: 2016/03/17]
116. Rosengren AL, Huang E, Daniels J, et al. Feasibility of using GrindrTM to distribute HIV self-test kits to men who have sex with men in Los Angeles, California. *Sexual health* 2016 doi: 10.1071/sh15236 [published Online First: 2016/05/23]
117. Solorio R, Norton-Shelpuk P, Forehand M, et al. Tu Amigo Pepe: Evaluation of a Multi-media Marketing Campaign that Targets Young Latino Immigrant MSM with HIV Testing Messages. *AIDS Behav* 2016;20(9):1973-88. doi: 10.1007/s10461-015-1277-6 [published Online First: 2016/02/07]
118. Talal AH, Andrews P, McLeod A, et al. Telemedicine-based Hepatitis C Virus (HCV) management for individuals on opioid agonist treatment (OAT). *Hepatology* 2016;63 (1 Supplement 1):475A.
119. Henwood R, Patten G, Barnett W, et al. Acceptability and use of a virtual support group for HIV-positive youth in Khayelitsha, Cape Town using the MXit social networking platform. *AIDS care* 2016;28(7):898-903. doi: 10.1080/09540121.2016.1173638 [published Online First: 2016/04/22]
120. Brayboy LM, Sepolen A, Mezoian T, et al. Girl Talk: A Smartphone Application to Teach Sexual Health Education to Adolescent Girls. *J Pediatr Adolesc Gynecol* 2017;30(1):23-28. doi: 10.1016/j.jpag.2016.06.011 [published Online First: 2016/07/10]
121. Horvath KJ, Oakes JM, Rosser BR, et al. Feasibility, acceptability and preliminary efficacy of an online peer-to-peer social support ART adherence intervention. *AIDS Behav* 2013;17(6):2031-44. doi: 10.1007/s10461-013-0469-1 [published Online First: 2013/04/05]
122. Gotz HM, van Rooijen MS, Vriens P, et al. Initial evaluation of use of an online partner notification tool for STI, called 'suggest a test': a cross sectional pilot study. *Sex Transm Infect* 2014;90(3):195-200. doi: sextrans-2013-051254 [pii]
10.1136/sextrans-2013-051254 [published Online First: 2014/01/07]
123. Friedman AL, Brookmeyer KA, Kachur RE, et al. An assessment of the GYT: Get Yourself Tested campaign: an integrated approach to sexually transmitted disease prevention communication. *Sex Transm Dis* 2014;41(3):151-7. doi: 10.1097/OLQ.0000000000000100
00007435-201403000-00001 [pii] [published Online First: 2014/02/14]
124. Hightow-Weidman LB, Muessig KE, Pike EC, et al. HealthMpowerment.org: Building Community Through a Mobile-Optimized, Online Health Promotion Intervention. *Health Educ Behav* 2015;42(4):493-9. doi: 1090198114562043 [pii]
10.1177/1090198114562043 [published Online First: 2015/01/16]
125. Dokkum NF, Koekenbier RH, van den Broek IV, et al. Keeping participants on board: increasing uptake by automated respondent reminders in an Internet-based chlamydia screening in the Netherlands. *BMC Public Health* 2012;12:176. doi: 1471-2458-12-176 [pii]
10.1186/1471-2458-12-176 [published Online First: 2012/03/13]
126. Hightow-Weidman L, Beagle S, Pike E, et al. "No one's at home and they won't pick up the phone": using the Internet and text messaging to enhance partner services in North Carolina. *Sex Transm Dis* 2014;41(2):143-8. doi: 10.1097/OLQ.0000000000000087
00007435-201402000-00014 [pii] [published Online First: 2014/01/15]
127. Aronson ID, Cleland CM, Perlman DC, et al. MOBILE SCREENING TO IDENTIFY AND FOLLOW-UP WITH HIGH RISK, HIV NEGATIVE YOUTH. *Journal of mobile technology in medicine* 2016;5(1):9-18. doi: 10.7309/jmtm.5.1.3 [published Online First: 2016/04/26]
128. Hirsch-Moverman Y, Daftary A, Yuengling KA, et al. Using mHealth for HIV/TB Treatment Support in Lesotho: Enhancing Patient-Provider Communication in the START Study. *J Acquir*

For peer review only - http://bmjopen.bmj.com/site/about/guidelines.xhtml

Immune Defic Syndr 2017;74 Suppl 1:S37-S43. doi: 10.1097/qai.0000000000001202 [published Online First: 2016/12/09]
129. John ME, Samson-Akpan PE, Etowa JB, et al. Enhancing self-care, adjustment and engagement through mobile phones in youth with HIV. *International nursing review* 2016;63(4):555-61. doi: 10.1111/inr.12313 [published Online First: 2016/09/23]
130. Guy R, Hocking J, Wand H, et al. How effective are short message service reminders at increasing clinic attendance? A meta-analysis and systematic review. *Health Serv Res* 2012;47(2):614-32. doi: 10.1111/j.1475-6773.2011.01342.x [published Online First: 2011/11/19]
131. Free C, Phillips G, Galli L, et al. The effectiveness of mobile-health technology-based health behaviour change or disease management interventions for health care consumers: a systematic review. *PLoS Med* 2013;10(1):e1001362. doi: 10.1371/journal.pmed.1001362
PMEDICINE-D-12-00520 [pii] [published Online First: 2013/01/26]
132. Ershad Sarabi R, Sadoughi F, Jamshidi Orak R, et al. The Effectiveness of Mobile Phone Text Messaging in Improving Medication Adherence for Patients with Chronic Diseases: A Systematic Review. *Iranian Red Crescent medical journal* 2016;18(5):e25183. doi: 10.5812/ircmj.25183 [published Online First: 2016/07/21]
133. Finitsis DJ, Pellowski JA, Huedo-Medina TB, et al. Visual analogue scale (VAS) measurement of antiretroviral adherence in people living with HIV (PLWH): a meta-analysis. *Journal of behavioral medicine* 2016;39(6):1043-55. doi: 10.1007/s10865-016-9770-6 [published Online First: 2016/08/03]
134. Kanters S, Park JJ, Chan K, et al. Interventions to improve adherence to antiretroviral therapy: a systematic review and network meta-analysis. *The lancet HIV* 2017;4(1):e31-e40. doi: 10.1016/s2352-3018(16)30206-5 [published Online First: 2016/11/20]
135. Mayer JE, Fontelo P. Meta-analysis on the effect of text message reminders for HIV-related compliance. *AIDS care* 2016:1-9. doi: 10.1080/09540121.2016.1214674 [published Online First: 2016/08/02]
136. Thakkar J, Kurup R, Laba TL, et al. Mobile telephone text messaging for medication adherence in chronic disease a meta-analysis. *JAMA Intern Med* 2016;176(3):340-49. doi: 10.1001/jamainternmed.2015.7667
137. Mbuagbaw L, Mursleen S, Lytvyn L, et al. Mobile phone text messaging interventions for HIV and other chronic diseases: an overview of systematic reviews and framework for evidence transfer. *BMC Health Serv Res* 2015;15:33. doi: 10.1186/s12913-014-0654-6
s12913-014-0654-6 [pii] [published Online First: 2015/01/23]
138. Muessig KE, Nekkanti M, Bauermeister J, et al. A Systematic Review of Recent Smartphone, Internet and Web 2.0 Interventions to Address the HIV Continuum of Care. *Curr Hiv/Aids Rep* 2015;12(1):173-90. doi: 10.1007/s11904-014-0239-3
139. Gabarron E, Wynn R. Use of social media for sexual health promotion: a scoping review. *Global health action* 2016;9:32193. doi: 10.3402/gha.v9.32193 [published Online First: 2016/09/22]
140. Chavez NR, Shearer LS, Rosenthal SL. Use of digital media technology for primary prevention of STIs/HIV in youth. *J Pediatr Adolesc Gynecol* 2014;27(5):244-57. doi: 10.1016/j.jpag.2013.07.008 [published Online First: 2013/12/18]
141. Barello S, Triberti S, Graffigna G, et al. eHealth for Patient Engagement: A Systematic Review. *Front Psychol* 2015;6:2013. doi: 10.3389/fpsyg.2015.02013 [published Online First: 2016/01/19]
142. Finitsis DJ, Pellowski JA, Johnson BT. Text Message Intervention Designs to Promote Adherence to Antiretroviral Therapy (ART): A Meta-Analysis of Randomized Controlled Trials. *PLoS ONE* 2014;9(2) doi: e88166
10.1371/journal.pone.0088166
143. Fletcher WR, Fletcher WS. Chapter 8: Treatment. Clinical Epidemiology: The Essentials. 4th ed. Baltimore: Lippincott Williams & Wilkins 2005:141.

For peer review only - http://bmjopen.bmj.com/site/about/guidelines.xhtml

144. Agarwal S, LeFevre AE, Lee J, et al. Guidelines for reporting of health interventions using mobile phones: mobile health (mHealth) evidence reporting and assessment (mERA) checklist. *BMJ* 2016;352:i1174. [published Online First: 2016/03/19]

For peer review only

For peer review only - http://bmjopen.bmj.com/site/about/guidelines.xhtml

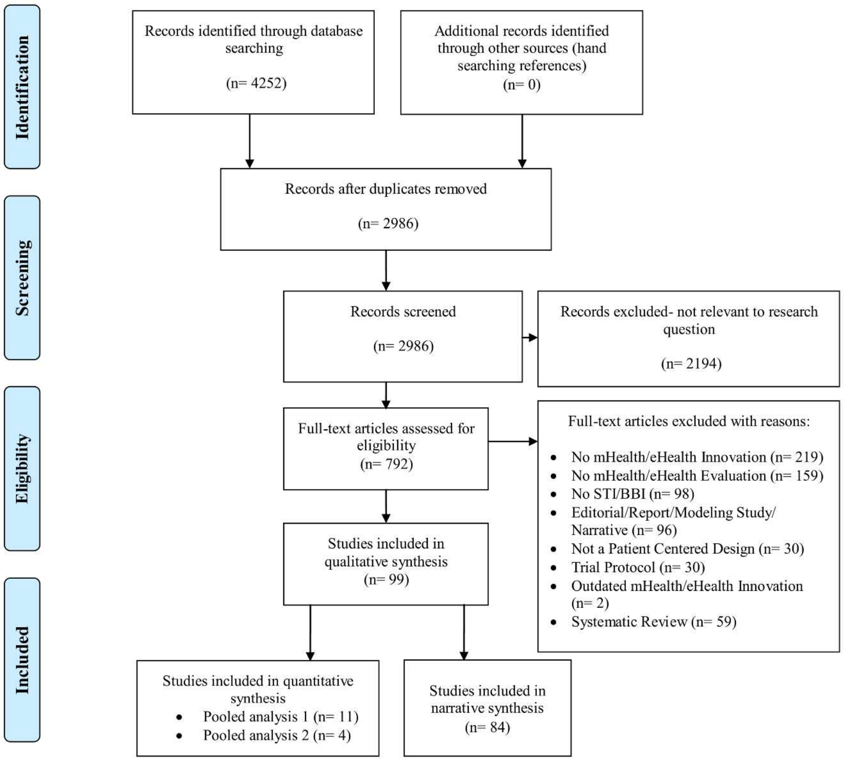

Figure 1. PRISMA Flow Diagram

215x279mm (300 x 300 DPI)

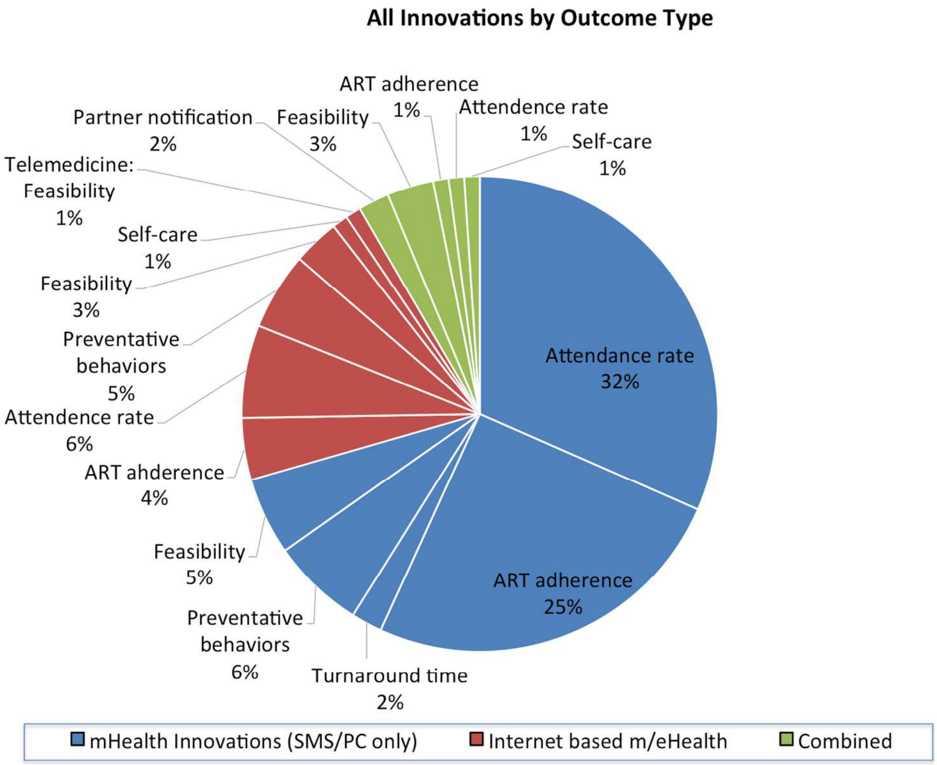

Figure 2. All Innovations by Outcome Type

279x215mm (300 x 300 DPI)

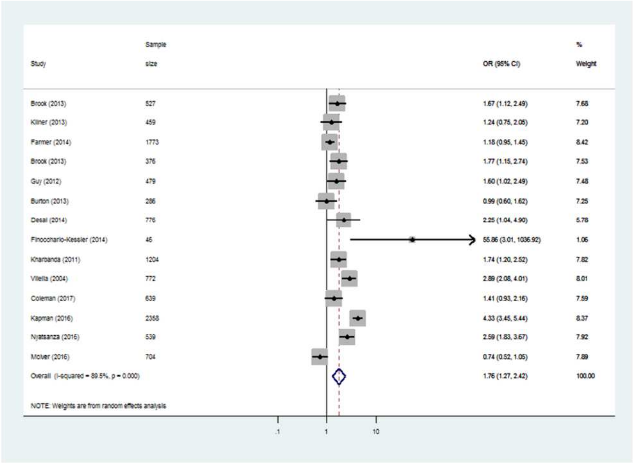

Figure 3A. Sub-Group Analysis Pooled OR for Attendance

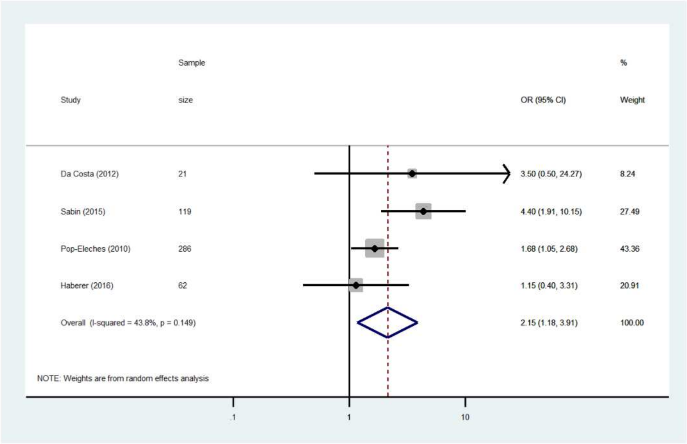

Figure 3B. Sub-Group Analysis Pooled OR for Adherence

Figure 3. Sub-Group Analyses

215x279mm (300 x 300 DPI)

Appendix 1: Search Strategy.

Search #1	"HIV Infections"[Mesh] OR "HIV" [MeSH] OR “human immunodeficiency virus”[tiab] OR “human immuno deficiency virus”[tiab] OR “human immune deficiency virus”[tiab] OR “human immunedeficiency virus”[tiab] OR “aids”[tiab] OR “acquired immunodeficiency syndrome”[tiab] OR “acquired immunodeficiency syndromes”[tiab] OR “acquired immuno deficiency syndrome”[tiab] OR “acquired immuno deficiency syndromes”[tiab] OR “acquired immune deficiency syndrome”[tiab] OR “acquired immune deficiency syndromes”[tiab] OR “acquired immunedeficiency syndrome”[tiab] OR “acquired immunedeficiency syndromes”[tiab]
Search #2	"mHealth" [tiab] OR "telemedicine"[MeSH] OR telemedicine[tiab] OR eHealth[MeSH] OR ehealth[tiab] OR "mobile health" [tiab] OR “mobile technology”[tiab] OR “app”[tiab] OR “apps”[tiab] OR “mobile applications” OR social medi*[tiab] OR cell phone* [tiab] OR cellphone*[tiab] OR “cellular phone”[mesh] OR cellular phone*[tiab] OR smartphone*[tiab] OR smart phone*[tiab] OR mobile phone[tiab] OR mobile device*[tiab] OR cellular telephone*[tiab] OR mobile telephone*[tiab] OR text messag*[tiab] OR texting[tiab] OR texted[tiab] OR SMS[tiab] OR MMS[tiab] OR multimedia messag*[tiab] OR short messag*[tiab] OR “computers, handheld”[mesh] OR personal digital assistant*[tiab]
Search #3 [1,2]<br><br>References<br><br>1.Ferreira A, Young T, Mathews C, Zunza M, Low N. Strategies for partner notification for sexually transmitted infections, including HIV. Cochrane Database of Systematic Reviews 2013, Issue 10. Art. No.: CD002843. DOI: 10.1002/14651858.CD002843.pub2<br><br>2.Obiero J, Mwethera PG, Wiysonge CS. Topical microbicides for prevention of sexually transmitted infections. Cochrane Database of Systematic Reviews 2012, Issue 6. Art. No.: CD007961. DOI: 10.1002/14651858.CD007961.pub2	sexually transmitted infections[mh] OR sexually transmitted disease*[tiab] OR sexually transmissible disease*[tiab] OR sexually transmitted infection*[tiab] OR sexually transmissible infection*[tiab] OR sexually transmitted infectious disease*[tiab] OR sexually transmissible infectious disease*[tiab] OR sexually transmitted disorder*[tiab] OR sexually transmissible disorder*[tiab] OR STI[tiab] OR STIs[tiab] OR STD[tiab] OR STIs[tiab] OR venereal disease*[tiab] OR venereal infection*[tiab] OR venereal disorder*[tiab] OR genital herpes[tiab] OR herpes genitalis[mh] OR herpes genitalis[tiab] OR genital infection*[tiab] OR genital disorder*[tiab] OR herpes simplex[tiab] OR herpes virus[tiab] OR HSV-1[tiab] OR HSV-2[tiab] OR chancroid[mh] OR chancroid* [tiab] OR haemophilus ducreyi[tiab] OR chlamydia infection*[tiab] OR chlamydia trachomatis[mh] OR chlamydia trachomatis[tiab] OR gonorrhea[mh] OR gonorrhoea*[tiab] OR gonorrhea*[tiab] OR syphilis[mh] OR syphilis[tiab] OR cuminat[tiab] OR condylomata lata[tiab] OR chancre*[tiab] OR lymphogranuloma venereum[mh] OR lymphogranuloma venereum[tiab] OR granuloma Inguinale[mh] OR granuloma inguinale[tiab] OR donovania[tiab] OR donovanosis[tiab] OR calymmatobacterium[mh] OR calymmatobacterium granulomatis[tiab] OR klebsiella granulomatis[tiab] OR klebsiella granulomatis[tiab] OR treponema pallidum[mh] OR treponema pallidum[tiab] OR genital wart*[tiab] OR venereal wart*[tiab] OR condylomata cuminate[mh] OR human papillomavirus 6[mh] OR hpv-6[tiab] OR hpv-11[tiab] OR hpv6[tiab] OR human papillomavirus[tiab] OR hepatitis b[mh] OR hepatitis b[tiab] OR trichomonas vaginitis[mh] OR trichomonas vaginitis[tiab] OR genital ulcer*[tiab] OR anogenital ulcer*[tiab] OR anorectal ulcer*[tiab] OR anorectal ulcer*[tiab] OR penile ulcer*[tiab] OR blood-born pathogen*[tiab] OR blood-borne infection*[tiab] OR blood-borne virus*[tiab]
Search #4	#1 OR #3
Search #5	#2 AND #4

For peer review only

Appendix 2: **Abstraction table.**

Combined Innovations	Author	Study Design	Participants/ Country	STBBI	Intervention	Measure/Metric	Results (MD=Mean difference, RR=risk ratio, IRR=incidence rate ratio, HR=hazard ratio, SD= standard deviation, 95% CI when presented. M=months, W=weeks)
**Online campaign + SMS + TV.**	Friedman 2014	Quasi-experimental: HxCtrl w/ population data.	≤25 yrs, USA. n=N/A	HIV, CT, GC	GetYourselfTested: TV campaign w/ website & SMS service for STI info & clinic locator.	ATT testing: Attendance rate.	41.2% more CT tests in 2010 vs. 2008, 90.5% more GC tests, and 190.3% more HIV tests.
						Acceptability: Number of followers.	4477 FB followers and 1994 Twitter followers at yr 2.
						Feasibility: Referral rate.	83,404 referrals using clinic locator in yr1. 61,119 in yr2.
**Interactive website + SMS + cash incentives.**	Horvath 2013	RCT	HIV+ Gay/Bi-sexual men 18+ yrs, USA. n=67(Int) n=57(Ctrl)	HIV	Online self-monitoring system w/ interactive interface + optional SMS reminders +$25 gift card draw.	ART in PVLA: Self-report. [Difference scores: DS = FU-baseline]	No difference. (DS=0.54, SD=25.2 vs. DS=-3.2, SD=24.5; t(107)=1.79, p=0.43) / No impact.
						ART in PVLA: Self-report.	Increased adherence in drug users (DS= 7.1, SD= 22.1 vs. DS= -24, SD= 30.5; t(17)=2.52, p=0.02) / Effective.
						ART in PVLA: Self-report.	Trend to taking meds within 2hrs of scheduled dose. DS=6.6, SD=29.3 vs. DS=-3, SD=29.6; t(105)=1.68, p=0.1 / No impact.
						Acceptability: Self-report.	Mean score = 5.7 on 7-point Likert Scale for satisfaction / Highly acceptable.
						Feasibility: Completion rate.	Completion rate 88% vs. 93% in Ctrl / Highly feasible.
**Website + SMS**	Gotz 2014	Cross-sectional study.	STI index patients at clinic, NLD. n=988	HIV, CT, GC, syph	Suggestatest.nl: online partner notification via SMS/email.	PN: % partners notified.	14% notifications via SAT. 505 notifications sent (84% by SMS, 15% by email). 56% read notification. 20% visited one of 2 STI clinics.
**Social media + SMS.**	Hightow-Weidman 2014	Quasi-experimental: HxCtrl.	HIV+ or syphilis+ patients, USA. n=362(Int) n=133(HxCtrl)	HIV, syphilis	Notification on social networking sites + SMS	PN: % partners notified.	63.5% of contacts notified via internet in 2011 vs. 26% in 2010.
**PC/SMS/MMS + WhatsApp messages**	John 2016	UnCtrlled trial.	HIV+ non-disclosed, 15-29 yrs, NGA. n=19	HIV	Weekly counselling, educational & motivational calls, SMS/MMS and WhatsApp messages over 3M.	Self-care: Self-report.	Significant increase in self-care performance at 6MI (p=0.002)/ Effective.
**Interactive website + SMS**	Hightow-Weidman 2015	Feasibility study.	Black MSM & transwamen 18-30 yrs, USA. n=15	HIV	HealthMpowerment.org: online community networking Int to reduce STI risk + health promotion messages.	Acceptability: Self-report.	86.7%-100% strongly agreed w/ acceptability questions / Highly acceptable.
						Feasibility: Retention rate.	100% retention rate. 7/15 participants used the site 1W after study ended / Highly feasible.
**Mobile app + SMS**	Hirsch-Moverman 2017	Feasibility study.	≥18yrs, HIV+/TB, LSO. n=171	HIV/TB	CommCare application used to automatically send SMS medication reminders over 29M	Acceptability: Self-report.	41.9% think SMS facilitated adherence to TB /ART medication / Less acceptable.

For peer review only

**Mobile app + SMS**	Aronson 2016	Feasibility study	18-24 yrs, USA. n=100	HIV	App assessing risk and sending SMS to encourage re-testing of HIV negatives.	Feasibility: Completion rate	98/100 completed the app process/ Highly feasible<br>30/100 accepted to receive HIV test<br>21/30 accepted to receive SMS<br>1/21 re-tested after 90 days window period.
**Website + SMS**	Dokkum 2012	UnCtrlled trial.	16-29 yrs, NLD. n=52600(Rd 1) n=41700(Rd 2)	CT	At-home CT test + SMS/email to return test for analysis.	Feasibility: Completion rate.	Higher retesting rates (From 10% w/o reminders to 14% in round 1; from 7% to 10% in round 2) / Less feasible.

Note: Int= intervention; Ctrl= control; HxCtrl= historical control; PB= preventative behaviors (i.e. risk reduction); PN= partner notification; TAT= turnaround time; ATT= attendance rate; ART= ART adherence; NAPs= non-adherent patients; AP= adherent patients; PVLA= Patients with various levels of adherence; TNPs= Treatment naive patients; VL= viral load; CD4= CD4 cell count; PC= phone call; FB= Facebook.

For peer review only

Internet-based eHealth Innovation	Author	Study Design	Participants/ Country	STBBI	Intervention	Measure/Metric	Results (MD=Mean difference, RR=risk ratio, IRR=incidence rate ratio, HR=hazard ratio, SD= standard deviation, 95% CI when presented. M=months, W=weeks)
**Online campaign**	Downshen 2015	Quasi-experimental: HxCtrl w/ population data.	13-17 yrs, USA. n=1500	HIV, CT, GC, syphilis	IknowUshould2: social-media campaign w/ website for STI info & clinic locator.	ATT testing: Attendance rate.	More syphilis tests (18.8% vs. 5.4%; p<0.01) and HIV tests (19.0% vs. 5.4%; p<0.01). No change for CT & GC / Effective.
						Acceptability: Number of followers.	1500+ unique website interactions. 128 FB likes; 46 Twitter followers; 390 Youtube views; 42 Instagram followers.
**Social media campaign**	Elliot 2016	Cross-sectional study.	MSM, GBR. n=17361	HIV	Promotion through Gaydar, Grindr, Recon and FB pages to order free postal HIV home sampling kits	ATT testing: Participation rate.	10 323/11 127 (93%) ordered HIV sample kit. 5696/10 323 (55%) returned sample kit within 24M. 82/5696 (1.4%) confirmed new diagnosis and in care.
						Acceptability: Self-report.	59.7% would recommend to someone expected to test positive (93.8% if expected to negative). 64% clicked for more info on test. / Moderately acceptable.
**Social media campaign**	Huang 2016	Cross-sectional	≥18yrs, Black/African American or Hispanic/Latino MSM, USA. n=122	HIV	Promoting of HIV self-testing for 6W on GrindR + study website to order self-test kit	ATT testing: Participation rate.	122 requested tests; 55/57 HIV-, 2/57 HIV+.
						Acceptability: Number of followers	11 939 unique website visitors; 2.8% click-through rate 334 tests requested.
						Feasibility: Completion rate.	122/334 visitors were eligible and completed baseline survey, 81/122 confirmed receiving self test kit, 57/122 completed follow-up survey / Less feasible.
**Social media campaign**	Jones 2015	Cross-sectional study.	MSM, GBR. n=305	HIV	Health promotion and offer of rapid at-home testing via FB, Grindr, and Squirt.	ATT testing: Participation rate.	5/5 high risk sexual behavior but tested HIV negative; 1/5 never tested before; 3/5 not tested in many yrs.
						Acceptability: Number of followers.	103 clicked FB survey; 152 approached on Grindr; 50 Squirt contacts.
						Feasibility: Completion rate.	FB: 6/103 completed survey; 3/6 requested HIV test; 2/3 made appointment. Grindr: 20/152 engaged; 6/20 requests for at home test; 3/6 made appointment. Squirt: 3/50 engaged and 0/3 test requests / Less feasible.
**Social media campaign**	Rhodes 2016	Quasi-experimental.	MSM & transgender, USA n=339 (Int) n=286 (Ctrl)	HIV	Posting info and answering questions on HIV testing on social media sites (Adam4Adam, BlackGayChat, Craigslist, and Gay.com).	ATT testing: Self-report.	63.7% of intervention participants reported past 12M HIV testing compared with 42.0% of control. Adjusted OR= 2.9 (1.8-4.7)/ Effective.
**Social media campaign + website**	Rosengren 2016	Cross-sectional	Black or Hispanic MSM 18+ yrs, USA n=56	HIV	Promotion of free rapid HIV self-testing kits on Grindr and offer of delivery via study website (kit, voucher or pin for smart vending machine)	ATT testing: Self-report.	All 56 reported testing completion (100%); 2/56 reported positive result and linkage to care (confirmatory testing and ART initiation)
						Feasibility: Completion rate.	4389 visited the website; 333 requested test (i.e. 1 in 13 visitors); 56 completed survey 2W after request/ Less feasible.
						ART in TNPs: Self-report.	Higher adherence at 3M & 6M (71.2% vs. 63.9%, d=0.17; 70.3% vs. 66.6%, d=0.09)
**Mobile phone application**	Himelhoch 2016	RCT	18-64yrs, history of drug/alcohol use, HIV+, USA. n=19(Int) n=9(Ctrl)	HIV	Heart2HAART mobile application for ART adherence	ART in NAPs: Pill count	No significant difference in adherence between intervention and control group (p=0.29), but adherence was 100% in both at 3M / No impact
						Acceptability: Self-report.	94.3% strongly agreed/agreed Heart2HAART helped them take their medication / Highly acceptable.
						Feasibility: Response rate.	App was used on avg 21.4, 19.1 and 16.4 times in months 1, 2 and 3. Participants responded to medication prompts on avg 18, 16 and 14 times during months 1,2 and 3 respectively.

For peer review only

For peer review only - http://bmjopen.bmj.com/site/about/guidelines.xhtml

**Avatar-guided computer software**	Kurth 2014	RCT	HIV+ 18+ yrs, USA. n=120(Int) n=120(Ctrl)	HIV	Audio narrated risk assessment, skill building videos, tailored feedback and printouts vs. computer risk assessment only.	ART in PVLA: VL.	Non-significant change. (log10VL= -0.06(-0.4 to -0.3), p=0.74). Significant in subgroup w/ detectable VL at baseline (-0.73(-1.42 to -0.03), p=0.041) / No impact.
						ART in PVLA: Self-report.	Increased adherence. (4.71(0.95- 8.48) increase vs. 1.39(6.03 to 3.24) decrease; p=0.046) / Effective.
						PB: Self-report.	Lower odds of HIV transmission (OR=0.46 (0.25-0.84), p=0.012) / Effective.
						Acceptability: Self-report.	97% reported ease of use and high privacy; 99% satisfied w/ session length; 75% preferred it over human counsellor / Highly acceptable.
						Feasibility: Retention rate.	87.1% retention / Highly feasible.
**Avatar-guided computer program**	Naar-King 2012	RCT	HIV+ 16-24 yrs, USA. n=36(Int) n=40(Ctrl)	HIV	2-D animated character delivering personalized health feedback vs. character giving nutrition info.	ART in TNPs: VL.	Larger suppression rate. (Cohen's d=0.09 at 3M; d= 0.28 at 6M). Larger drop in VL from baseline (d=0.39 at 3M & d=0.19 at 6M).
						ART in TNPs: Self-report.	Higher adherence at 3M & 6M (71.2% vs. 63.9%, d=0.17; 70.3% vs. 66.6%, d=0.09)
						Acceptability: Self-report.	Mean satisfaction ratings 3.7 out of 4 / Highly acceptable.
**Mobile phone application**	Perera 2014	RCT	HIV+, NZ. n=17(Int) n=11(Ctrl)	HIV	ART adherence app w/ medication clock & graphs on disease-state vs. standard app (medication clock only)	ART in PVLA: Self-report.	Increased adherence (F(1,23)=5.37, p=0.03) / Effective.
						ART in PVLA: Pharmacy refills.	No difference. (F(1,25)=1.88, p=0.18) / No impact.
						ART in PVLA: VL.	Lower VL at 3M (F(1,23)=5.62, p=0.023) / Effective.
						ART in PVLA: Composite score (refills, VL, & self-report).	Increased adherence (53% to 13%, X2(1,15)=6, p=0.03). No change in Ctrl (27% to 27%, X2(1,11)=0.00, p>0.99) / Effective.
						Acceptability: Self-report.	More satisfying (on 11 point-scale: 5.88 vs. 3.27, p=0.017) and informative (6 vs. 3, p=0.034) at 3M than standard app / Highly acceptable.
**Mobile app + cash incentive**	Brayboy 2017	UnCtrlled trial.	12-17yrs, USA. n=17	STI	GirlTalk mobile phone app to assess knowledge increase	PB: Self-report.	75.6% to 79% increase in knowledge pre and post app use at 2W. / No impact.
						Acceptability: Self-report.	94.1% would use the app again/recommend it / Highly acceptable
**Social media**	Jones 2012	Quasi-experimental: HxCtrl.	15–24 yrs, USA. n=70/896 FB friends	CT	Educational FB site addressing safe sexual health.	PB: Self-report.	Condom from 57% to 80%. 54% reduction in CT in ages 15-17 from previous yrs (but 42% less tests done).
**Videos vs. SMS**	Jones 2013	RCT	High-risk urban African-American women 18-29 yrs, USA. n=117(Soap opera) n=121(SMS)	HIV	Weekly soap opera episodes (Love, Sex & Choices) vs. HIV prevention SMS.	PB: Self-report.	18% greater reduction in Int. group, p=0.23 / No impact. 78% reduction in risky acts from baseline in Int. group (p<0.001); 72% reduction from baseline in Ctrl (p<0.001)/ Effective
						Acceptability: Self-report.	97.4% liked the videos / Highly acceptable.
**Social media + video chat**	Lelutiu-Weinberger 2014	UnCtrlled trial.	MSM 18-29 yrs, high risk for STI, USA. n=31	HIV	miCHAT: FB chat Int. 8 motivational interviews to reduce HIV risk + CBT training.	PB: Self-report.	Decrease in unprotected anal sex acts (3.11 vs. 8.96; p=0.042). Increased knowledge of sexual risk (p=0.01) / Effective.
						Acceptability: Self-report.	All felt privacy was ensured / Highly acceptable.
						Feasibility: Completion rate.	46% completed baseline assessment + minimum 5 sessions / Less feasible.

For peer review only - http://bmjopen.bmj.com/site/about/guidelines.xhtml

**Social media campaign + website + cash incentive**	Solorio 2016	Feasibility study.	Hispanic MSM, 18-30 yrs, USA n=50	HIV	Radio & social media-based campaign for 16W to encourage testing & condome use + website w/clinic locator to provide free HIV home testing kits and linkage to care	PB: self-report.	No significant change in condom use at 16W (26.1% vs. 15.65, OR=1.9 (0.6-5.9))/ No impact.
						Feasibility: Self-report.	32/50 (64%) requested HIV home testing kit, 28/32 (88%) completed the test/ Moderately feasible.
**Mobile app**	Jeon 2016	RCT.	Chronic HBV+, 19-60 yrs, KOR n=26 (Int) n=27 (Ctrl)	HBV	App to increase disease knowledge, set alarm medication reminders, record lab nutrition & physical activity data, and chat with other users.	Self-care: Self-report.	Significantly higher self-care performance in intervention vs. control (t=3.597, p=0.001)/ Effective.
						Feasibility: Utilisation rate.	Average monthly utilisation rate was 75.1%/ Highly feasible.
**Social media**	Henwood 2016	Feasibility study.	12-25 yrs, HIV+, ZAF n=90	HIV	Use of MXit as support group for HIV+ youth	Acceptability: Self-report.	84% would like chat-room to continue / Highly acceptable.
						Feasibility: Participation rate	33% ever visited MXit chat-room / Less feasible.
**Mobile app + cash incentive**	Przybyla 2016	Feasibility study.	HIV + on ART, 18+ yrs, USA n=27	HIV	DRUM app to report daily on ART adherence and substance abuse.	Acceptability: Self-report.	84% reported the app was easy to use; 96% were satisfied; 92% would use it in the future/ Highly acceptable.
						Feasibility: Completion rate.	Overall completion rate of daily reports after 2W= 95.3%/ Highly feasible.
**Telemedicine**	Talal 2016	Feasibility study.	Individuals on opioid agonist tx, USA n=54	HCV	Telemedicine-based medical tx with hepatologist	Acceptability: Self-report.	88.9% prefer medical tx using telemedicine vs. clinic visit; 100% would recommend it to a friend/ Highly acceptable.
						Feasibility: Completion rate.	54 tested HCV+ over 14M; 81.5% started evaluation/tx; 75% of those given tx have completed it/ Highly feasible.
**Social media**	Garett 2016	Feasibility study.	18+yrs, MSM, PER n=102(Int) n=109(Ctrl)	HIV	12W FB based peer-led intervention to increase HIV testing and prevention behaviour.	Acceptability: Self-report.	Intervention group felt they learned more about; where to receive sexual health services (p-value=0.0061), more likely to have safe sex (p-value=0.034) and more likely to get tested for HIV regularly (p-value=0.021) compared to control group / Highly acceptable.
**Website**	Polilli 2016	Feasibility study.	Residents of Abruzzo Region, ITA n=3500	HIV, syphilis, HBV, HCV	Website with STI info, risk calculator, and appointments booking at testing sites.	Feasibility: Completion rate.	3500 booked an appointment; 3046 (87%) presented for testing within 15M study period/ Highly feasible.

Note: Int= intervention; Ctrl= control; HxCtrl= historical control; PB= preventative behaviors (i.e. risk reduction); PN= partner notification; TAT= turnaround time; ATT= attendance rate; ART= ART adherence; NAPs= non-adherent patients; AP= adherent patients; PVLA= Patients with various levels of adherence; TNPs= Treatment naive patients; VL= viral load; CD4= CD4 cell count; PC= phone call; FB= Facebook.

For peer review only

Basic mHealth Innovation	Author	Study Design	Participants/ Country	STBBI	Intervention	Measure/Metric	Results (MD=Mean difference, RR=risk ratio, IRR=incidence rate ratio, HR=hazard ratio, SD= standard deviation, 95% CI when presented. M=months, W=weeks)
**SMS**	Bailey 2014	UnCtrlled trial.	CT+ at clinic, AUS. n=64	CT	SMS reminders to recall for treatment.	ATT treatment: Attendance rate.	100% treated for CT infection. 72% treated within 1 day of SMS.
						Feasibility: Response rate.	94% replied to SMS, 84% the same day / Highly feasible.
**SMS + PC**	Bassett 2016	RCT	≥18yrs, ZAF. n=543(Int) n=471(Ctrl)	HIV/TB	5 scheduled PC) and 4 SMS, reminders to retrieve test results and attend appointments, over 4M.	ATT treatment: Attendance rate.	No significant difference in reaching outcome at 9M (3M ART treatment or 3+6M of TB treatment) between intervention and control (39% vs 42%, RR=0.93, 95%CI 0.80-1.08) / No Impact
**SMS + PC**	Bigna 2014	RCT	Caregivers of HIV + children 18+ yrs, CMR. n=61(SMS+PC) n=60(PC) n=60(SMS) n=61(Ctrl)	HIV	SMS+PC, SMS, or PC appointment reminders.	ATT FU appointment: Attendance rate.	Improved attendance. (OR=2.9 (1.3-6.3), p=0.012) / Effective.
**SMS**	Brook 2013	Quasi-experimental: HxCtrl.	Sexual health clinic, GBR. n=207(Int) n=169(HxCtrl)	HIV, syphilis, HBV	SMS reminders.	ATT testing: Attendance rate.	Higher retesting rate (41% vs. 28%; p<0.001) / Effective.
**SMS**	Brook 2013	Quasi-experimental: HxCtrl.	Sexual health clinic, GBR.n=699(Int) n=768(HxCtrl)	HIV	SMS reminders 2 days before appointment.	ATT FU appointment: LTFU rate.	35% improvement in overall LTFU rate (26% to 17%; p<0.0001) / Effective.
**SMS**	Burton 2013	Quasi-experimental: HxCtrl.	High risk for STI at clinic, GBR. n=273(Int) n=266(Ctrl)	CT, GC	SMS STI testing reminders.	ATT: testing: Attendance rate.	No change in retesting rates for those w/ recent CT or GC. (CT: 36% vs.33%; p=0.79) (GC: 19% vs. 33%; p=0.48) / No impact.
**SMS**	Coleman 2017	Retrospective Quasi-experimental	>=18 yrs, HIV+ pregnant women, ZAF. n=192(Int) n=447(Ctrl)	HIV	Bi-weekly maternal health info sent throughout pregnancy and for one year after birth to increase HIV PCR testing postpartum and increase ANC visits	ATT testing: Attendance rate.	81.3% vs 75.4% in intervention vs control group likely to attend first PCR 6W postpartum. 40% increase in the likelihood of attending the recommended four ANC visits among individuals within the intervention group (RR: 1.41, CI: 1.15–1.72) / Effective.
						PB: Infection rate	3 infants born with HIV in control group
**SMS**	Desai 2014	Quasi-experimental: Conc. + HxCtrl.	High risk MSM at clinic, GBR. n=31(Int) n=656(Conc. Ctrl) n=745(HxCtrl)	HIV	SMS HIV/STI testing reminders.	ATT testing: Attendance rate.	No significant change in re-testing odds. (32% in SMS vs.30% in Conc. Ctrl; OR=1.1(0.5-2.4) and 17% in HxCtrl; OR=2.3(1.0-4.9) / No impact.
**SMS + cash incentive**	Downing 2013	RCT	CT + or suspected at clinic 16+ yrs, AUS. n=30(Int) n=32(Ctrl)	CT	SMS appointment reminders + $10 if attended.	ATT testing: Attendance rate.	Increased re-testing rate at 10-12W post CT treatment (without cash 26.7% vs. 6.3% in Ctrl; p=0.04); (with cash 28.1% vs. 6.3% in Ctrl; p=0.043) / Effective.
**SMS**	Evans 2015	UnCtrlled trial.	African community, GBR. n=172	HIV	2 weekly Health Belief Model SMS to reduce risky sexual behaviours.	ATT testing: Self-report.	10.5% reported being tested for HIV during/after the 12W Int.
						PB: Self-report.	Non-significant increase in HIV knowledge & attitudes / No impact.
						Acceptability: Self-report.	Acceptable & useful. Majority shared w/ others and want to get tested in future.

For peer review only

**SMS**	Farmer 2014	Quasi-experimental: HxCtrl.	HIV clinic attendees, GBR. n=951(Int) n=822(HxCtrl)	HIV	SMS reminder 2 days before appointment.	ATT FU appointment: LTFU & cancellation rate.	No difference in LTFU (25% vs.28%) or cancellation (62% vs.64%) / No impact.
**SMS**	Finocchario-Kessler 2014	Quasi-experimental: HxCtrl.	HIV+ mother-infant pairs, KEN. n=523(Int) n=320(HxCtrl)	HIV	SMS notification of available test results and appointment reminder.	ATT treatment: Attendance rate.	More infants initiated on ART (Urban: 11/11 vs. 1/7, p<0.001; Peri-urban: 14/14 vs. 9/14, p<0.05) / Effective.
						TAT: Time from test to diagnosis & test to treat.	Shorter median time to diagnosis (5 vs. 6.3W (urban) & 3.4 vs. 8.1W (peri-urban); both p<0.001). Shorter median time to treat (13 vs. 40 days (urban) & 1 vs. 36 days (peri-urban); p<0.001) / Effective.
						Feasibility: Retention rate.	Retention rate double at 9M post-natal (45.1% vs. 93% (urban) and 43.2% vs. 94.1% (peri-urban); p<0.001) / Highly feasible.
**SMS**	Guy 2012	Quasi-experimental: HxCtrl.	STI clinic, AUS. n=141(Int) n=338(HxCtrl)	CT	SMS re-testing reminder 3M after initial infection.	ATT testing: Attendance rate.	Higher retesting rate (30% 1-4M post-infection vs. 21%; p=0.04); AOR= 1.57(1.01-2.46) / Effective.
**SMS**	Joseph Davey 2016	RCT.	HIV+ adults on ART, MOZ n=416 (Int) n=414 (Ctrl)	HIV	SMS reminders 2 and 7 days of appointment and ART drug-pick up + educational SMS every 2M.	ATT treatment: Attendance rate.	Nonsignificant difference in overall retention in care at 12 M (93.8% vs 91%, p=0.139)/ No impact.
**SMS**	Kapman 2016	Quasi-experimental: HxCtrl.	Heterosexual clinic attendees dx & tx for CT, 16-23 yrs, NLD n=828 (Int) n=1530 (Ctrl)	CT	2 SMS reminders at 5.5M & 6M after initial dx with CT for retesting appointment scheduling & attendance.	ATT testing: Attendance rate.	Higher attendance rate between 5-8M after initial dx (30.6% vs. 9.2%).
**SMS**	Kharbanda 2011	Quasi-experimental: Conc. + HxCtrl.	Parents of girls 9-20 yrs at clinics, USA. n=124(Int) n=308(Conc. Ctrl) n=1080(HxCtrl)	HPV	Up to 3 weekly SMS vaccination reminders.	ATT vaccination: Attendance rate.	More likely to get vaccine on time after controlling for insurance and site of care (AOR=1.83(1.23-2.71)) / Effective.
**SMS**	Kliner 2013	Quasi-experimental: HxCtrl.	HIV+ at hospital, SWZ. n=162(Int) n=297(HxCtrl)	HIV	SMS reminders one day before appointment.	ATT FU appointment: Attendance rate.	No difference. SMS 83.3% vs. Ctrl 80.1%; p=0.401. AOR=1.13, p=0.662 / No impact.
**SMS**	Matheson 2014	Quasi-experimental.	11-22 yrs at clinic, USA. n=37(Int) n=232(Ctrl)	HPV	SMS vaccination reminders (3 SMS per dose).	ATT vaccination: Attendance rate.	Higher attendance rate. HPV2 vaccine complete: 73% vs.34%, (p=0.000); on-time HPV2 38% vs. 25%, (p=0.035). HPV3 complete 16% vs.6%, (p=0.018); on-time HPV3 14% vs.3%, (p=0.007) / Effective.
**SMS**	McIver 2016	Quasi-experimental: HxCtrl.	Clinic attendees susceptible to HBV (HIV+, bisexual, CSW, IDUs, Aboriginals), AUS n=241 (Int) n=463 (Ctrl)	HBV	SMS reminders 1 day before appointment for HBV vaccine doses 2&3 reattendance.	ATT vaccination: Attendance rate.	Nonsignificant decrease in attendance rate within 12 M (54% vs 56% for 2 doses, p=0.65/ 24% vs 30% for 3 doses, p=0.07)/ No impact Nonsignificant difference in completion of 3 doses in 12M. aOR= 0.7 (0.48-1.01)/No impact.
**SMS**	Njuguna 2016	RCT.	Rural women, 18-24 yrs, KEN n=300 (Int) n=300 (Ctrl)	HIV	Weekly SMS on HIV and reproductive health.	ATT testing: Self-report.	Significant increase in reported testing at 6M (67% vs 51%, aHR=1.54(1.25-1.90)/ Effective.
**SMS vs. PC**	Norton 2014	RCT	HIV+, 17+ yrs, USA. n=25(Int) n=27(Ctrl)	HIV	SMS appointment reminder vs. message to home phone.	ATT FU appointment: Attendance rate.	No difference (72% vs. 81%, p=0.42) but patients already had high attendance rate / No impact.

For peer review only

For peer review only - http://bmjopen.bmj.com/site/about/guidelines.xhtml

**SMS**	Nyatsanza 2016	Quasi-experimental: HxCtrl.	MSM & CSW at high-risk of STI, GBR n=266 (Int) n=273 (Ctrl)	HIV/STI	Personalised SMS reminders for reattendance.	ATT testing: Attendance rate.	Significantly higher reattendance rate at 6M (56% vs. 33%, p<0.001)/ Effective.
**SMS**	Odeny 2012	RCT	Males circumcised at clinic 18+ yrs, KEN. n=600(Int) n=600(Ctrl)	HIV	Daily SMS for 1W.	ATT FU appointment: Attendance rate.	Improved attendance within 3 days of post-operative clinic appointment: 65.4% vs.59.7% (RR=1.09(1.00–1.20); p=0.04) / Effective.
						PB: Self-report.	Abstention of sexual activity before FU: 28.3% vs. 25.2% (RR=1.13(0.91-1.38), p=0.3) / No impact.
**SMS**	Rand 2015	RCT	11-16 yrs at clinic, USA. n=1893(Int) n=1919(Ctrl)	HPV	SMS appointment reminders.	ATT vaccination: Attendance rate.	Higher HPV1 vaccination rate (16% vs. 13%; HR= 1.3(1.0-1.6); p=0.04) / Effective.
**SMS/PC**	Rand 2016	RCT.	Clinic attendees Parents of youth 11-17 yrs who received 1st HPV vaccine, USA. n=191 (SMS) n=200 (Ctrl); n=178 (PC) n=180 (Ctrl)	HPV	SMS appointment reminders to receive 3 doses of HPV vaccine over 2 yrs.	ATT vaccination: Attendance rate.	SMS: Significant difference in vaccination rates compared to control (49% vs 30%, p=0.001)/ Effective. PC: No difference in vaccination rates compared to control (48% vs 40%, p=0.34)/ No impact.
						TAT: Time from enroll to completion of 3 vaccines.	SMS: Significant difference in time taken to complete 3 HPV doses (71 days earlier than control, p<0.001)/ Effective. PC: No difference in time taken to complete 3 HPV doses compared to control (p=0.08)/ No impact.
**SMS + PC**	Schwartz 2015	Quasi-experimental: HxCtrl.	HIV+ pregnant women on ART, ZAF. n=50	HIV	SMS messages and PCs from a case manager (CM) through 6W postpartum.	ATT testing: Attendance rate.	More infant testing (90.0% vs. 63.3% at 10W; p<0.01) / Effective.
						Acceptability: Self-report.	Helpful to have CM support during pregnancy and postpartum (98%) / Highly acceptable.
						Feasibility: Completion rate.	96% completed postpartum questionnaire / Highly feasible.
**SMS + PC**	Segaren 2012	UnCtrlled trial.	Mothers of HIV+ infants, HTI. n=108	HIV	Cell phones + regular PC for monitoring of mother & child.	ATT treatment: Attendance rate.	All 76 w/ active phones were adherent to treatment (attended 6/6 monthly hospital appointments).
						Acceptability: Self-report.	70% phones active after Int.; good for med reminders (63%) / Moderately acceptable.
**SMS + PC**	Smillie 2014	UnCtrlled trial.	HIV+ in clinic 14+ yrs, CAN. n=20	HIV	Weekly PC or SMS for 6M.	ATT FU appointment: Self-report.	65% said SMS had no effect on attendance.
						Acceptability: Self-report.	Beneficial for appointment scheduling (80%) & reminder (75%). All would recommend to a friend / Highly acceptable.
						Feasibility: Self-report.	75% had no difficulty in receiving and responding to SMS / Highly feasible.
**SMS**	Tolly 2012	RCT	Randomly sampled adults (existing database), ZAF. n=438(in each of 4 Int.) n=801(Ctrl)	HIV	3 or 10 motivational or informational SMS.	ATT testing: Self-report.	Improved attendance in group receiving 10 motivational SMS at 3W: (69% vs. 57%; OR=1.7(1.10–2.390), p=0.0036) / Effective.
						Feasibility: Self-report.	SMS motivated HIV counseling and testing uptake in 89% / Highly feasible.
**SMS**	Vilella 2004	Quasi-experimental: Conc. + HxCtrl.	18+ yrs at travel clinic, ESP. n=738(Int) n=1610(Conc. Ctrl) n=2247(HxCtrl)	HAV/ HBV	SMS reminders for vaccination appointments.	ATT vaccination: Attendance rate.	Improved adherence for 3rd HepA+B dose. (47.1% vs. 26.9%, RR=1.75(1.41–2.17) in Conc. Ctrl and 23.6%(20.1–27.4), RR=2.00(1.63–2.45) in HxCtrl) / Effective.

For peer review only - http://bmjopen.bmj.com/site/about/guidelines.xhtml

**SMS**	Ammassari 2010	UnCtrlled trial.	HIV+, ITA. n=71	HIV	SMS reminders.	ART in NAPs: Self-report.	Increased adherence over 9M. (93.2% vs.79.6%, p=0.003) / Effective.
**SMS**	Ammassari 2011	UnCtrlled trial.	HIV+, 18+ yrs, ITA. n=145	HIV	SMS reminders.	ART in NAPs: Self-report.	Increased adherence at 9M (94.9% vs.78.8%, p<0.001) / Effective.
						ART in NAPs: VL.	More w/ undetectable VL at 9M (76.2% vs. 42.3%, p<0.001) / Effective.
						Acceptability: Self-report.	>90% reporting SMS helpful / Highly acceptable.
**PC + cash incentives**	Belzer 2014	RCT	HIV+ 12-29 yrs, USA. n=19(Int) n=18(Ctrl)	HIV	Daily PC reminders and referrals if necessary+ free phone & plan.	ART in NAPs: Self-report.	Increased adherence for 1M &3 M (OR=3.09(1.20-7.98); OR=2.85(1.02-7.97)) / Effective.
						ART in NAPs: VL.	Lower VL at wk 24 and 48 (2.82 vs. 4.52, p=0.002; 3.23 vs. 4.23, p=0.043) / Effective.
**SMS**	Cantudo-Cuenca 2016	Retrospective quasi-exprimental.	HIV + on ART, ESP n=120 (Int&Ctrl)	HIV	SMS on ART adherence.	ART in PVLA: Pharmacy refills.	Statistically sign relationship bt no SMS and ART adherence(OR= 0.35 (0.14-0.8), p=0.025) [multivariate analysis]/ Effective.
**SMS**	da Costa 2012	RCT	HIV+ women, BRA. n=8(Int) n=13(Ctrl)	HIV	Daily SMS reminders.	ART in APs: Pill count.	Increased adherence over 4M (50% vs. 38.5%; p=0.604) / No impact.
						ART in APs: MEM.	Increased adherence over 4M (75% vs. 46%; p=0.195) / No impact.
						ART in APs: Self-report.	Increased adherence (100% vs. 84.6% in Ctrls; p=0.244) / No impact.
						Acceptability: Self-report.	82% believed SMS were helpful, 77% wanted to keep receiving SMS / Highly acceptable.
**SMS**	Downshen 2011	UnCtrlled trial.	HIV+ 14-29 yrs, AUS. n=25	HIV	Daily SMS ART reminder + FU SMS 1hr later.	ART in NAPs: Self-report.	Increased adherence (Baseline Mean=74.7; 12W Mean=93.3; 24WMean=93.1; p<0.001) / Effective.
						ART in NAPs: VL + CD4 count.	Insignificant change in CD4 cell count & VL (mean VL= 2750, CD4= 502 to VL= 29, CD4= 545 at 24W, p=0.12) / No impact.
						Acceptability: Self-report.	81% want SMS after study end. Helped decrease missed doses in 95% / Highly acceptable.
**SMS**	Downshen 2011	UnCtrlled trial.	HIV+ 14-29 yrs, AUS. n=25	HIV	Daily SMS ART reminder + FU SMS 1hr later.	ART in NAPs: Self-report.	Decreased adherence (58.3% for 0-12W vs. 55.2% for 13-24W, p=0.53) / No impact.
						Feasibility: Completion & response rate.	84% completed all study visits. 61.4% response rate / Highly feasible.
**SMS + cash incentive**	Garofalo 2016	RCT	16-29yrs, HIV+ on ART for ≥1M , USA. n=51(Int) n=54(Ctrl)	HIV	Daily personlised SMS over 6M to remind participants take medications	ART in NAPs: Self-report.	Significant difference in adherence compared to control at 3M OR=2.57 (1.01-6.54). Not significant at 6M OR=1.68 (0.69-4.09). Significant difference from baseline to 6M OR=2.12 (95% CI 1.01-4.45). / Effective.
						ART in NAPs: VL.	No difference in log viral load or viral suppression compared to control at 3 and 6M / No impact.
						Acceptability: Self-report.	100% would recommend intervention to those in need, 81 % wanted to continue getting the text messages after conclusion of the study, 95 % satisfied with the intervention overall / Highly acceptable
						Feasibility: Response rate.	58% average response rate to SMS / Moderately feasible.

For peer review only - http://bmjopen.bmj.com/site/about/guidelines.xhtml

**SMS +PC**	Haberer 2016	RCT	≥18yrs, HIV+ on ART, UGA. n=21(Scheduled SMS) n=20 (Triggered SMS) n=21(Ctrl)	HIV	Scheduled SMS: 1M daily SMS, 2M weekly SMS, 6M SMS sent to patient and support if needed. Triggered SMS; SMS sent to patient and support if no signal received from monitor.	ART: MEM	Significant difference in scheduled SMS intervention compared to control (11.1% increase in adherence, 48-h and more than 96-h lapses were less frequent (IRR=0.6, p value=0.02 and IRR 0.3, P<0.001, respectively). Similar adherence in triggered SMS vs control group. / Effective.
						ART: VL	No significant differences in HIV RNA suppression among study arms (p value = 0.14). 47/62 participants virally suppressed at 3 and 9M / No impact.
**SMS**	Hardy 2011	RCT	HIV+ 18+ yrs, USA. n=12(SMS) n=14(Beeper)	HIV	SMS vs. beeper reminders.	ART in APs: Composite score (MEM+ pill count + self-report).	Higher adherence at 6W. (MD=27.1(7.6-46.6), p =0.009) / Effective.
						ART in APs: MEM.	Increased adherence. (MD=33.4(14.1-52.6), p = 0.002) / Effective.
						ART in APs: Pill count.	No difference. (MD=13.7(-6.7-34.1), p = 0.153) / No impact.
						ART in APs: Self-report.	No difference. (MD=20.2 (-1.8-42.1), p = 0.069) / No impact.
**SMS**	Jeffries 2016	RCT	15-24yrs, HIV+, USA. n=91(Int) n=45(Ctrl)	HIV	UCARE4LIFE daily moblie text messageing intervenetion over 3M to improve HIV care among youth	ART: VL	Significant difference in ART adherence in intervention vs control among non-adherent/new to ART at baseline (6M p=0.03). / Effective. No sig difference in those on ART at baseline (6M p=0.119) /No impact.
						Acceptability: Self-report.	Mean score = 8.44 (SD=2.45) on 10 point Likert Scale for appointment reminder SMS./ Highly acceptable
**PC**	Kalichman 2011	RCT	HIV+ 18+ yrs, USA. n=21(Int) n=19(Ctrl)	HIV	PC counselling.	ART in NAPs: Pill count.	No difference at 4M (F(1,36)=3.32, p<0.07) / No impact.
						Feasibility: Completion rate.	99% completion rate / Highly feasible.
**SMS**	Kassaye 2016	RCT.	HIV+ pregnant women, KEN n=280 (Int) n=270 (Ctrl)	HIV	3 to 6 weekly SMS (ART reminders, motivational, PMTCT, child health & nutrition).	ART in TNPs: Self-report.	Nonsignificant difference in adherence to ART at 34-36W gestation between the 2 groups (97.3% vs 99.6%, aRR= 1.25 (0.43-3.60)./No impact. Nonsignificant difference in adherence to ART at delivery between the 2 groups (94.7% vs 100%, aRR=1.01 (0.88-1.16))./ No impact.
**PC**	Kebaya 2014	RCT	HIV+ mothers in PMTCT, KEN. n=75(Int) n=75(Ctrl)	HIV	Bi-weekly PC.	ART in TNPs: Self-report.	Increased adherence (90.7% vs. 72%, p=0.005) / Effective.
						Feasibility: Retention rate.	More likely to remain in treatment at 10W (69.3% vs 37.3%, p<0.001) / Moderately feasible.
**SMS**	Lester 2010	RCT	HIV+ 18+ yrs, KEN. n=273(Int) n=265(Ctrl)	HIV	Weekly SMS.	ART in TNPs: Self-report.	Improved adherence at 6M and 12M: RR=0.81(0.69-0.94) p=0.006 / Effective.
						ART in TNPs: VL.	Lower virological failure (RR=0.84(0.71-0.99) p=0.04) and improved viral suppression (OR=0.71(0.5-1.01) p=0.058) / Effective.
**SMS + PC + cash incentives**	Maduka 2013	RCT	HIV+ at hospital 20+ yrs, NGA. n=52(Int) n=52(Ctrl)	HIV	2 monthly counselling PCs + 2 weekly SMS+ cash incentives	ART in NAPs: Self-report.	Increased adherence (76.9% vs. 55.8%, X2=5.211,p=0.022; RR=0.725(0.55-0.96)) / Effective.
						ART in NAPs: CD4 count.	Improved CD4+ count (193-->575 cells/mL vs. 131-->361.5 cells/mL; p=0.007) / Effective.
**SMS + PC**	Mbuagbaw 2012	RCT	HIV+ 21+ yrs, CMR. n=101(Int) n=99(Ctrl)	HIV	Weekly motivational SMS. Phone number to call for support.	ART in PVLA: Self-report.	No difference. (RR=1.06(0.89-1.29); p=0.542) / No impact.
						ART in PVLA: Pharmacy Refills.	No difference at 6 months (MD=0.1(-0.23-0.43); p=0.617) / No impact.
						Acceptability: Self-report.	91.1% believed SMS reminders helped; 65% were satisfied; 81.2% would recommend to a friend / Highly acceptable.

For peer review only

**SMS**	Moore 2015	RCT	HIV+ bipolar 18+ yrs, USA. n=25(Int) n=25(Ctrl)	HIV	SMS reminders.	ART in PVLA: MEM.	No difference. (86.2% (SD= 12.7) vs. 84.8% (SD= 18.1); p=0.95; d=0.01) / No impact.
**SMS**	Nsagha 2016	RCT.	HIV+ on ART, 18+yrs, CMR n=45 (Int) n=45 (Ctrl)	HIV	4 weekly educative SMS over 1M.	ART in PVLA: Self-report.	Nonsignificant difference in adherence to ART at 1M between the 2 groups (64.4% vs 44.2%, p=0.056)/ No impact.
						Acceptability: Self-report.	57.8% wished the SMS to continue/ Moderately acceptable
**SMS**	Pop-Eleches 2010	RCT	HIV+ 18+ yrs, rural KEN. n=142(Daily SMS) n=147(Weekly SMS) n=139(Ctrl)	HIV	Daily or weekly SMS.	ART in PVLA: MEM.	Increased adherence in weekly SMS group over 48W (53% vs. 40% p=0.03) / Effective.
						ART in PVLA: MEM.	No difference between daily SMS group and Ctrl (41% vs. 40% p=0.92) / No impact.
**SMS + cash incentive**	Rana 2016	UnCtrlled trial.	HIV+, 18+yrs, USA. n=32	HIV	Bi-directional weekly SMS appointment reminders, daily ART reminder & supportive messages.	ART in PVLA: Undetectable VL	Significant increase in the number of participants with undetectable VL at 6M (25 vs. 18, p=0.002)/ Effective.
						ATT treatment: Attendance rate.	20/32 completed all visits within 6M study period.
**SMS**	Sabin 2015	RCT	HIV+ 18+ yrs, in CHN. n=63(Int) n=56(Ctrl)	HIV	SMS reminders via MEM + adherence counselling.	ART in PVLA: MEM.	Increased adherence over 6M (82% vs. 51.8%; RR=1.59(1.21- 2.10), p<0.001) / Effective.
						ART in PVLA: VL.	No difference in undetectable VL (93.6% vs. 98.2%, p=0.218) / No impact.
						ART in PVLA: CD4 count.	Higher mean change in CD4 count (52 vs 28 cell/μL, p=0.297) / No impact.
**PC + MMS.**	Shet 2014	RCT	HIV+ 18-60 yrs, IND. n=315(Int) n=316(Ctrl)	HIV	Weekly automated motivational voice call, followed by weekly MMS.	ART in TNPs: VL.	No difference. (Number of virological failures: 15.6% vs. 15.5%. Time to virological failure: aHR= 0.96(0.65-1.43), p= 0.85) / No impact.
						ART in TNPs: Pill count.	No difference. (27% vs. 21.7%; aIRR=1.24(0.94-1.63), p=0.13) / No impact.
						Feasibility: PC received.	86% of calls received by patients / Highly feasible.
**SMS**	Walsh 2012	UnCtrlled trial.	HIV+ Adults on ART, GBR. n=14	HIV	Pill-box w/ MEM + weekly SMS wrt med taking + up to 3 late dose SMS reminders.	ART in APs: Self-report + MEM.	99.5% baseline adherence, 98% at 24W. No difference in missed doses (4.8% in 0-12W; 6.3% in 13-24W)
						Acceptability: Self-report.	64% satisfied, 50% found SMS & system useful. 55% found reminders irritating / Moderately acceptable.
**SMS**	Lim 2008	Quasi-experimental: HxCtrl.	STI clinic, NZL. n=293(Int) n=303(HxCtrl)	CT	SMS to contact clinic for CT test result.	TAT: Time from test to treat.	No change in median time to treat (3 days vs. 4 days, t = - 1.3, p<0.1) / No impact.
**SMS**	Menon-Johansson 2006	Quasi-experimental.	At clinic w/untreated CT, GBR. n=28(Int) n=21(Ctrl)	CT	SMS to contact clinic for CT test result.	TAT: Time from test to diagnosis & test to treat.	Shorter mean time to diagnosis. (7.9 days vs. 12.5; p<0.001) Shorter median time to treat. (8.5 days vs. 15; p=0.005) / Effective.
**SMS+PC**	Barnabas 2016	RCT	16-49 yrs,, ZAF & UGA. n=284(Int) n=224(Ctrl)	HIV	SMS promoting male circumcision 3W, 6-7W after tested negative. Follow-up phone call 1M & 2M following SMS reminders.	PB: Self-report.	Significant difference in reaching outcome at 3M (Intervention vs clinic referral); 48% (RR=1.72 95% CI 1.36-2.17, p values < 0.0001) in SMS reminder group and 47% (RR=1.67, 95%CI 1.29-2.14, p value = 0.0001) in lay counsellor follow-up achieved MC at 3M / Effective
**SMS + MMS.**	Cornelius 2013	UnCtrlled trial.	African-Americans age 13-18, USA, n=40	HIV	HIV-prevention SMS + knowledge question for	PB: Self-report.	Improved condom attitudes & HIV knowledge (83% vs.78% correct answers) / No impact.

For peer review only

For peer review only - http://bmjopen.bmj.com/site/about/guidelines.xhtml

					3W.	Acceptability: Self-report.	97% satisfied w/ number of SMS. 86% reported SMS not interfering w/ daily activities/ Highly acceptable.
						Feasibility: Completion rate.	100% at pretest; 90% at 3M FU/ Highly feasible.
**PC**	DiClemente 2014	RCT	High-risk African-American women 14-20 yrs, USA. n=342(Int) n=359(Ctrl)	CT	PC w/ prevention messages every 8W.	PB: % diagnosed w/ CT or GC.	Fewer participants diagnosed w/ CT & GC (90 vs. 104; RR = 0.5 (0.28-0.88), p=0.02. 48 vs. 54; RR = 0.4 (0.15-1.02), p=0.06) / Effective.
						PB: Self-report.	Higher condom use (MD=0.08(0.06 to 0.10) p=0.04) / Effective.
**SMS + cash incentive**	Juzang 2011	Non-randomized Ctrlled trial.	African-American men 16-20 yrs, USA. n=30/group	HIV	3 weekly SMS HIV prevention messages + $40 for completion.	PB: Self-report.	No statistical difference in % of protected sex. Higher awareness of sexual health / No impact.
						Feasibility: Retention rate.	20 (67%) retained in Ctrl & 19 (63%) in SMS group after 2nd FU / Moderately feasible.
**SMS**	Odeny 2014	RCT	Circumcised male at clinic, 18+ yrs, KE. n=600(Int) n=600(Ctrl)	HIV	Daily SMS for 1wk + SMS on days 8, 14, 21, 28, 35, 41, and 42 post-procedure.	PB: Self-report.	Abstention of sexual activity before 42-day follow up: 139/491(28.3%) vs. 124/493(25.2%) in control group (RR=1.13(0.91-1.38), p=0.3)/ No impact.
**SMS**	Reback 2015	UnCtrlled trial.	MSM drug users 18-65 yrs, USA. n=52	HIV	Daily SMS for 2W to reduce risky sexual behaviours.	PB: Self-report.	Reduction in anal sex (6.9 vs. 2.6, t97=2.82, p<0.05) and unprotected anal sex (1.8 vs. 0.5, t97=2.19, p<0.05) in past 2M/ Effective.
**PC**	Belzer 2015	RCT	HIV+ 12-29 yrs, USA. n=19(Int) n=18(Ctrl)	HIV	PC 1hr from time to take medication.	Acceptability: Self-report.	94% satisfied w/ call length and 81% would continue receiving calls / Highly acceptable.
						Feasibility: Retention rate.	63% retention rate / Moderately feasible.
**SMS**	Dean 2012	Feasibility study.	HIV+ at antenatal clinics, ZAF. n=7	HIV	SMS support group+ inquiries answered by physicians.	Acceptability: Self-report.	Overall satisfaction.
						Feasibility: Self-report.	SMS easily kept confidential.
**SMS**	Roth 2014	Feasibility study.	Sex workers 18+ yrs, USA. n=26	HIV	Cell phone diaries to collect info about sexual events.	Acceptability: Self-report.	Cell-phone electronic dairies to collect sensitive information acceptable (84.6%)/ Highly acceptable.
						Feasibility: Completion rate.	90.3% surveys completed / Highly feasible.
**SMS**	Georgette 2016	Feasibility study.	≥18yrs, HIV+, ZAF. n=88	HIV	Weekly SMS reminders to increase ART adherence and appointment reminders	Acceptability: Self-report.	92% would recommend SMS program to a friend, 90.9% said frequency of SMS was just right, 2/88 felt the SMS program slightly violated their privacy. 97.7% reported it helped them remember to take medication. 77.3% agreed that it helped them remember appointments. / Highly acceptable
**SMS**	Reid 2014	Cross-sectional study.	HIV+, BWA. n=42(Int) n=41(Ctrl)	HIV	SMS ARV pick-up reminder.	Acceptability: Self-report.	SMS helpful 93% (Int) vs. 58% (Ctrl) (p<0.001). SMS may lead to serostatus disclosure 10% vs. 56% (p<0.001). 95% satisfied w/ appointment scheduling. 90% would continue receiving SMS / Highly acceptable.
**PC**	Bauermeister 2014	Feasibility study.	MSM 18-30, USA. n=124	HIV	IVRS: microbicide use.	Feasibility: Self-report.	75.5% reported no problems using IVRS / Highly feasible.
**SMS + MMS.**	Cornelius 2011	Feasibility study.	African-Americans age 13-18, USA. n=12	HIV	HIV-prevention SMS+knowledge question for 3W.	Feasibility: Response rate.	80% response rate/ Highly feasible.

Note: Int= intervention; Ctrl= control; HxCtrl= historical control; PB= preventative behaviors (i.e. risk reduction); PN= partner notification; TAT= turnaround time; ATT= attendance rate; ART= ART adherence; NAPs= non-adherent patients; AP= adherent patients; PVLA= Patients with various levels of adherence; TNPs= Treatment naive patients; VL= viral load; CD4= CD4 cell count; PC= phone call; FB= Facebook.

For peer review only - http://bmjopen.bmj.com/site/about/guidelines.xhtml

Appendix 3. Table of studies by innovation (in rows) and by outcomes (in columns)

	Outcome / Digital Innovation	Attendance rate	ART adherence	Risk reduction	Partner notification	Turnaround time	Self-care	Feasibility†	Acceptability†
Number of Studies by Type of Digital Innovation	***mHealth Innovations (SMS/phone call only)***	*30**	*24*	*6*	*0*	*2**	*0*	*5*	*2*
	Internet-based m/eHealth Innovations	*6*	*4*	*5*	*0*	*0*	*1*	*4*	*1*
	Combined innovations	*1*	*1*	*0*	*2*	*0*	*1*	*3*	*1*

*Note: *1 study evaluated both attendance rate and turnaround time and was counted as part of the 30 studies on attendance rate. † studies reporting feasibility and acceptability as secondary outcomes are counted elsewhere in the table depending on primary outcome.*

For peer review only

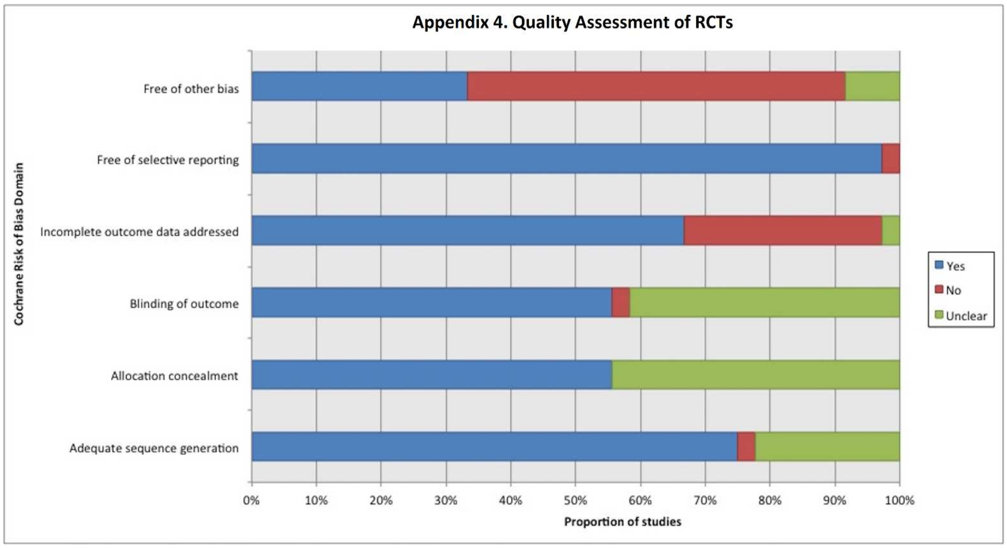

317x171mm (300 x 300 DPI)

review only

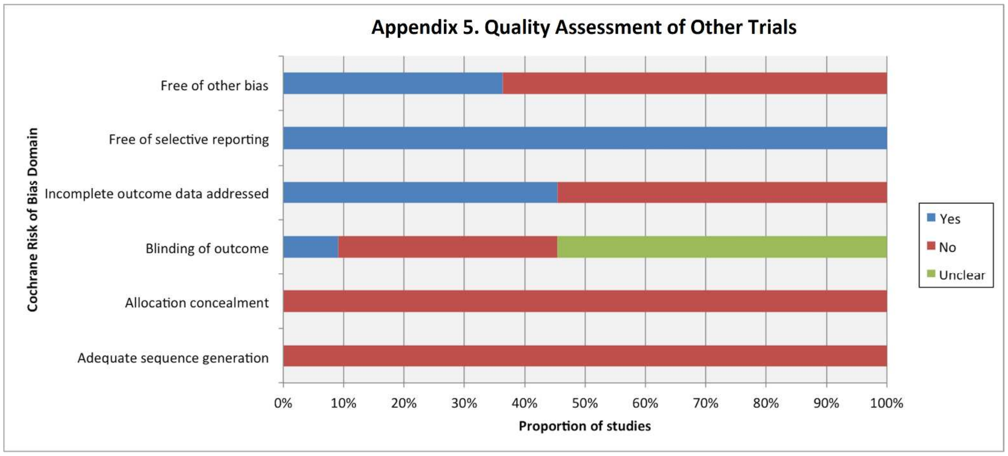

281x126mm (300 x 300 DPI)

For peer review only

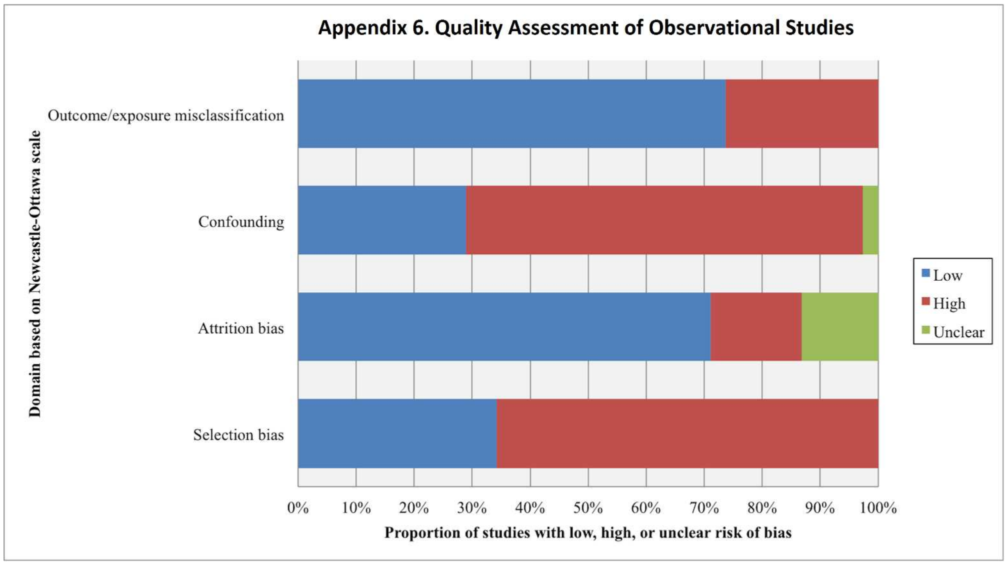

260x145mm (300 x 300 DPI)

review only

PRISMA 2009 Checklist

Section/topic	#	Checklist item	Reported on page #
**TITLE**			
Title	1	Identify the report as a systematic review, meta-analysis, or both.	1
**ABSTRACT**			
Structured summary	2	Provide a structured summary including, as applicable: background; objectives; data sources; study eligibility criteria, participants, and interventions; study appraisal and synthesis methods; results; limitations; conclusions and implications of key findings; systematic review registration number.	2
**INTRODUCTION**			
Rationale	3	Describe the rationale for the review in the context of what is already known.	3
Objectives	4	Provide an explicit statement of questions being addressed with reference to participants, interventions, comparisons, outcomes, and study design (PICOS).	3
**METHODS**			
Protocol and registration	5	Indicate if a review protocol exists, if and where it can be accessed (e.g., Web address), and, if available, provide registration information including registration number.	N/A
Eligibility criteria	6	Specify study characteristics (e.g., PICOS, length of follow-up) and report characteristics (e.g., years considered, language, publication status) used as criteria for eligibility, giving rationale.	4
Information sources	7	Describe all information sources (e.g., databases with dates of coverage, contact with study authors to identify additional studies) in the search and date last searched.	4
Search	8	Present full electronic search strategy for at least one database, including any limits used, such that it could be repeated.	4
Study selection	9	State the process for selecting studies (i.e., screening, eligibility, included in systematic review, and, if applicable, included in the meta-analysis).	4
Data collection process	10	Describe method of data extraction from reports (e.g., piloted forms, independently, in duplicate) and any processes for obtaining and confirming data from investigators.	4
Data items	11	List and define all variables for which data were sought (e.g., PICOS, funding sources) and any assumptions and simplifications made.	4
Risk of bias in individual studies	12	Describe methods used for assessing risk of bias of individual studies (including specification of whether this was done at the study or outcome level), and how this information is to be used in any data synthesis.	5
Summary measures	13	State the principal summary measures (e.g., risk ratio, difference in means).	5
Synthesis of results	14	Describe the methods of handling data and combining results of studies, if done, including measures of consistency (e.g., I^2) for each meta-analysis.	4

For peer review only - http://bmjopen.bmj.com/site/about/guidelines.xhtml

PRISMA 2009 Checklist

Section/topic	#	Checklist item	Reported on page #
Risk of bias across studies	15	Specify any assessment of risk of bias that may affect the cumulative evidence (e.g., publication bias, selective reporting within studies).	5
Additional analyses	16	Describe methods of additional analyses (e.g., sensitivity or subgroup analyses, meta-regression), if done, indicating which were pre-specified.	4
**RESULTS**			
Study selection	17	Give numbers of studies screened, assessed for eligibility, and included in the review, with reasons for exclusions at each stage, ideally with a flow diagram.	5
Study characteristics	18	For each study, present characteristics for which data were extracted (e.g., study size, PICOS, follow-up period) and provide the citations.	Abstraction Table
Risk of bias within studies	19	Present data on risk of bias of each study and, if available, any outcome level assessment (see item 12).	8
Results of individual studies	20	For all outcomes considered (benefits or harms), present, for each study: (a) simple summary data for each intervention group (b) effect estimates and confidence intervals, ideally with a forest plot.	6
Synthesis of results	21	Present results of each meta-analysis done, including confidence intervals and measures of consistency.	6
Risk of bias across studies	22	Present results of any assessment of risk of bias across studies (see Item 15).	8, Supplementary
Additional analysis	23	Give results of additional analyses, if done (e.g., sensitivity or subgroup analyses, meta-regression [see Item 16]).	6
**DISCUSSION**			
Summary of evidence	24	Summarize the main findings including the strength of evidence for each main outcome; consider their relevance to key groups (e.g., healthcare providers, users, and policy makers).	8
Limitations	25	Discuss limitations at study and outcome level (e.g., risk of bias), and at review-level (e.g., incomplete retrieval of identified research, reporting bias).	10
Conclusions	26	Provide a general interpretation of the results in the context of other evidence, and implications for future research.	10
**FUNDING**			
Funding	27	Describe sources of funding for the systematic review and other support (e.g., supply of data); role of funders for the systematic review.	13

From: Moher D, Liberati A, Tetzlaff J, Altman DG, The PRISMA Group (2009). Preferred Reporting Items for Systematic Reviews and Meta-Analyses: The PRISMA Statement. PLoS Med 6(7): e1000097. doi:10.1371/journal.pmed1000097

For more information, visit: www.prisma-statement.org.

For peer review only - http://bmjopen.bmj.com/site/about/guidelines.xhtml

PRISMA 2009 Checklist

For peer review only

For peer review only - http://bmjopen.bmj.com/site/about/guidelines.xhtml
